# Gel-free proteomic analysis of soybean root proteins affected by calcium under flooding stress

**DOI:** 10.3389/fpls.2014.00559

**Published:** 2014-10-20

**Authors:** MyeongWon Oh, Yohei Nanjo, Setsuko Komatsu

**Affiliations:** ^1^Life Sciences and Bioengineering, Graduate School of Life and Environmental Sciences, University of TsukubaTsukuba, Japan; ^2^National Institute of Crop Science, National Agriculture and Food Research OrganizationTsukuba, Japan

**Keywords:** soybean, flooding, calcium, proteomics, root

## Abstract

Soybean is sensitive to flooding stress and exhibits reduced growth under flooding conditions. To better understand the flooding-responsive mechanisms of soybean, the effect of exogenous calcium on flooding-stressed soybeans was analyzed using proteomic technique. An increase in exogenous calcium levels enhanced soybean root elongation and suppressed the cell death of root tip under flooding stress. Proteins were extracted from the roots of 4-day-old soybean seedlings exposed to flooding stress without or with calcium for 2 days and analyzed using gel-free proteomic technique. Proteins involved in protein degradation/synthesis/posttranslational modification, hormone/cell wall metabolisms, and DNA synthesis were decreased by flooding stress; however, their reductions were recovered by calcium treatment. Development, lipid metabolism, and signaling-related proteins were increased in soybean roots when calcium was supplied under flooding stress. Fermentation and glycolysis-related proteins were increased in response to flooding; however, these proteins were not affected by calcium supplementation. Furthermore, urease and copper chaperone proteins exhibited similar profiles in 4-day-old untreated soybeans and 4-day-old soybeans exposed to flooding for 2 days in the presence of calcium. These results suggest that calcium might affect the cell wall/hormone metabolisms, protein degradation/synthesis, and DNA synthesis in soybean roots under flooding stress.

## Introduction

Flooding events caused by heavy rainfall have increased globally over the past six decades as a consequence of climate change (Bailey-Serres et al., 2012). Flooding severely affects the productivity of farmland, because most agriculturally important crops are intolerant to flooding stress (Setter and Waters, 2003). One of the main effects of flooding is a marked reduction in oxygen availability due to the slower diffusion of gas in water as compared to air (Dat et al., 2004). Thus, when plants are exposed to flooding conditions, the roots initially suffer from oxygen deficiency (Sauter, 2013), leading to the inhibition of root respiration and a marked decrease in the energy status of root cells (Ashraf, 2012). In response to flooding, plants activate an alternative fermentation metabolic pathway to produce ATP and regenerate NAD^+^ (Gibbs and Greenway, 2003), as evidenced by the drastic increase in alcohol dehydrogenase activity in soybean under flooding stress (Komatsu et al., 2011). Although the flooding-induced changes in energy metabolism are important processes in adaptation to flooding conditions, metabolism induced by flooding and adaption to flooding in soybean remain unclear.

Calcium is an essential plant nutrient that determines the structure of cell wall and membranes, and plays a role in regulation of growth and development (Hepler, 2005). Calcium also has functions in protecting the integrity of cell membranes, reducing membrane permeability, and preventing ion leakage caused by biotic and abiotic stresses (Lin et al., 2008). Exogenous calcium alleviated the suppression of plant growth and kept plant to maintain and modulate cellular function by relieving gene repression under salt (Henriksson and Nordin Henriksson, 2005), anoxic (Aurisano et al., 1995), and chilling stresses (Gao et al., 2004). Calcium served the regulated optimal amounts of antioxidative enzymes and antioxidants to the antioxidative systems in leaves of sweet potato under waterlogging stress (Lin et al., 2008). Furthermore, improved tolerance to short-term hypoxia by calcium-mediated reduction of polyamine degradation, elevation of nitrate uptake, and accelerated synthesis of heat-stable proteins and polyamines was reported in muskmelon roots (Gao et al., 2011). Calcium was also shown to be effective in soybean for reducing Phytophthora stem rot disease, which is caused by *Phytophthora soja*, in flooded soil (Sugimoto et al., 2005). These observations indicate that study of the roles of calcium in protecting plants against environmental stresses, including flooding, will aids in the understanding of stress tolerance mechanisms in soybean.

Soybean is an important crop worldwide as it serves an abundant source of both protein and oil for animal and human consumption (Hartman et al., 2011). However, soybean is particularly sensitive to flooding (Sullivan et al., 2001), which markedly reduces the growth and productivity of plants (Githiri et al., 2006). Studies on the flooding-responsive mechanisms in soybean using proteomics-based approaches have revealed that the levels of several proteins involved in signal transduction, glucose degradation/sucrose accumulation, alcohol fermentation, and cell wall loosening were changed under flooding conditions (Komatsu et al., 2012a). Specifically, proteins involved in energy production were increased, whereas proteins involved in protein folding and cell structure maintenance were decreased in response to flooding stress in soybean (Nanjo et al., 2012). In addition, these proteomic studies identified a number of calcium-related proteins that may play important roles in flooding stress-responsive mechanisms in soybean.

To further understand calcium-related signaling pathway under flooding condition, Komatsu et al. (2013a) investigated calcium-related proteins in soybean cotyledon under flooding conditions using a proteomic technique and suggested that calcium might play a role in flooding-induced signal transduction through heat shock protein 70. Annexin, a calcium-dependent membrane-binding protein, was identified in soybean under flooding stress with abscisic acid supplementation (Komatsu et al., 2013b). The levels of calreticulin, a calcium-binding protein with chaperone functions (Menegazzi et al., 1993), were also lower in soybean under flooding stress (Komatsu et al., 2009), and a number of annexin proteins, calcium-transporting ATPase 4, calnexin, luminal-binding protein, and calcium ion-binding protein have been identified in flooded soybean root using gel-based and gel-free proteomic techniques (Komatsu et al., 2012b). These changes have effect on the calcium signaling under flooding stress and appear to be contributed flooding-responsive mechanism. Various calcium-related proteins, including annexin, calreticulin, calcium-binding EF-hand family protein, and calcium-dependent lipid-binding family protein, are changed in soybean under flooding stress (Oh et al., 2014a). Taken together, these results indicate that calcium has functions as a key signaling regulator in response to flooding by controlling calcium-related proteins in soybean. However, calcium-induced flooding response metabolism and response in soybean remain to be determined.

Because of the importance of calcium-regulatory mechanisms in plants to adjust to adverse abiotic stresses, including flooding, several studies have examined the involvement of calcium in stress-response mechanisms. Although calcium has been shown to ameliorate stress-induced damage in other crop species (He et al., 2012), calcium-mediated flooding-responsive mechanisms are poorly understood in soybean. In the present study, a gel-free proteomic technique was used to investigate the effect of calcium on soybean under flooding stress.

## Materials and methods

### Plant material and treatment

Soybean (*Glycine max* L. cultivar Enrei) seeds were sterilized with 1% sodium hypochlorite solution, rinsed in water, and sown in a plastic case (180 × 140 × 45 mm) containing 500 mL quartz sand wetted with 125 mL water. Soybeans were grown in a growth chamber under white fluorescent light (160 μmol m^−2^s^−1^, 16 h light period/day) at 25°C and 70% relative humidity. Two-day-old soybeans were transferred to a glass tube (38 × 130 mm) containing 120 mL tap water supplemented without or with 1, 5, 10, and 50 mM CaCl_2_ for flooding stress treatment, and further grown at 25°C under dark conditions. For physiological experiments, 4, 6, and 8-day-old soybeans treated with flooding for 2, 4, and 6 days, respectively, were collected and the length and weight of roots, including the hypocotyl, were measured. At the time of collection, soybeans were also stained with Evans blue dye, and the amount of dye extracted from stained root tips was measured spectroscopically as described below. For proteomic analysis, roots were collected from 4-day-old soybeans flooded without or with 50 mM CaCl_2_ for 2 days. For quantitative reverse transcription polymerase chain reaction (qRT-PCR) analysis, roots, hypocotyls, and cotyledons were collected from 2-, 3-, and 4-day-old soybean flooded without or with 50 mM CaCl_2_ for 0, 1, and 2 days, respectively. For all experiments, non-treated equivalent soybeans were collected as controls, and three independent biological replicates were performed for each experiment (Supplemental Figure 1).

### Evans blue staining for assay of cell death

Root tip cell death was evaluated by Evans blue staining, as described by Baker and Mock (1994) and Delisle et al. (2001). Briefly, soybeans were stained in a 0.25% aqueous solution of Evans blue for 15 min at room temperature. The stained samples were washed with water and immediately photographed. For quantitative assessment of staining, the terminal 5 mm of stained root tips was excised and immersed in 200 μL N,N-dimethylformamide for 24 h at 4°C. After the incubation, the absorbance of Evans blue released from the root tips was measured spectroscopically at 600 nm.

### Protein extraction

A portion (0.5 g) of fresh roots was ground to a powder in liquid nitrogen with a mortar and pestle. The powder was added to an acetone solution containing 10% trichloroacetic acid and 0.07% 2-mercaptoethanol, and the resulting mixture was vortexed and then sonicated for 10 min. The suspension was incubated for 1 h at −20°C with vortexing every 15 min, and was then centrifuged at 9000× g for 20 min at 4°C. The resulting supernatant was discarded and the obtained pellet was washed twice with 0.07% 2-mercaptoethanol in acetone. The pellet was dried using a Speed-Vac concentrator (Savant Instruments, Hicksville, NY, USA) and resuspended in lysis buffer containing 7 M urea, 2 M thiourea, 5% CHAPS, and 2 mM tributylphosphine by vortexing for 1 h at 25°C. The suspension was centrifuged at 20,000× g for 20 min at 25°C, and the supernatant was collected as protein extract. Protein concentrations were determined using the Bradford method (Bradford, 1976) with bovine serum albumin as the standard.

### Protein purification and digestion for mass spectrometry analysis

Extracted proteins (100 μg) were purified with methanol and chloroform to remove any detergent from the sample solutions, as previously described (Nanjo et al., 2012). Briefly, 400 μL methanol was added to 100 μL samples and then mixed. A total of 100 μL chloroform and 300 μL water were added to resulting mixture, mixed, and centrifuged at 20,000× g for 10 min to achieve phase separation. The upper phase was discarded, 300 μL methanol was added to lower phase, and the samples were further centrifuged at 20,000× g for 10 min. The supernatants were discarded, and the obtained pellets were dried. Dried samples were reduced with 50 mM dithiothreitol for 30 min at 56°C and the alkylated with 50 mM idoacetoamide for 30 min at 37°C in the dark. Alkylated proteins were digested with trypsin and lysyl endopeptidase at 1:100 enzyme/protein concentrations at 37°C for 16 h. The resulting tryptic peptides were acidified with formic acid, desalted with a C18-pipette tip (SPE C-TIP, Nikkyo Technos, Tokyo, Japan), and then analyzed by nano-liquid chromatography (LC)-tandem mass spectrometry (MS).

### Nanoliquid chromatography-mass spectrometry analysis

Using an Ultimate 3000 Nano LC system (Dionex, Germering, Germany), peptides in 0.1% formic acid were loaded onto a C18 PepMap trap column (300 μm ID × 5 mm, Dionex). The peptides were eluted from the trap column with a linear acetonitrile gradient (8–30% in 120 min) in 0.1% formic acid at a flow rate of 200 nL/min, and then loaded onto a C18 NANO HPLC NTTC-360/75-3 capillary tip column (75 μm ID × 120 mm, Nikkyo Technos) using a spray voltage of 1.5 kV. A nanospray LTQ Orbitrap mass spectrometer (Thermo Fisher Scientific, San Jose, CA, USA) was operated in data-dependent acquisition mode with the installed Xcalibur software (version 2.0.7, Thermo Fisher Scientific). Full-scan mass spectra were acquired in the Orbitrap MS over 400–1500 m/z with a resolution of 30,000. A lock mass function was used for high mass accuracy (Olsen et al., 2005). The 10 most intense precursor ions were selected for collision-induced fragmentation in the linear ion trap at a normalized collision energy of 35%. Dynamic exclusion was employed within 90 s to prevent the repetitive selection of peptides (Zhang et al., 2009).

### Protein identification

Proteins were identified by Mascot searches (version 2.4.1, Matrix Science, London, UK) of a soybean peptide database (55,787 sequences) constructed from the soybean genome database (Phytozome version 9.1, http://www.phytozome.net/soybean) (Schmutz et al., 2010). The acquired raw data files were processed using Proteome Discover software (version 1.4, Thermo Fisher Scientific). The parameters used in the Mascot searches were as follows: carbamidomethylation of cysteine was set as a fixed modification and oxidation of methionine was set as a variable modification. Trypsin was specified as the proteolytic enzyme and one missed cleavage was allowed. Peptide mass tolerance was set at 5 ppm, fragment mass tolerance was set at 0.5 Da, and peptide charge was set at +2, +3, and +4. An automatic decoy database search was performed as part of the analysis. Mascot results were filtered with the Percolator function to improve the accuracy and sensitivity of peptide identification. False discovery rates for peptide identification in all searches were less than 1.0%. The Mascot results were imported for SIEVE analysis (version 2.1, Thermo Fisher Scientific), which was performed as described in the following section.

### Data analysis of differential abundant proteins acquired using mass spectrometry

For differential analysis of the relative abundance of peptides and proteins between the control and treatment groups, the commercial label-free quantification package SIEVE was used. All SIEVE data analysis was acquired from 3 biological replicates of MS results. The chromatographic peaks detected by MS were aligned, and the peptide peaks were detected as a frame for all parent ions scanned by MS/MS using a frame time width of 5 min and frame m/z width of 10 ppm. Chromatographic peak areas within frames of each sample were compared, and the ratios between two sample groups for each frame were determined. The frames detected in the MS/MS scans were matched with the imported Mascot results. The ratios of peptides between samples were determined from the variance-weighted average of the ratios in frames that matched to the peptides with MS/MS spectrum. The ratios of peptides were further integrated with Ingenuity Pathways Analysis which is a widely-adopted application for 3 biological replications of MS into a ratio of protein to determine the ratio of the corresponding protein. In the differential analysis, total ion current was used for normalization. For the identification of differentially changed proteins, the minimum requirements for the identification of a protein were two matched peptides, and the protein quantities required a greater than two-fold difference with *t*-test significance (*P* < 0.05) between the flooding-treated and control samples.

### Functional categorization analysis

Proteins were categorized based on function using MapMan bin codes (Usadel et al., 2005).

### RNA extraction and quantitative reverse transcription-polymerase chain reaction analysis

A portion (100 mg) of soybean roots, hypocotyls, and cotyledons was ground into powder with a sterilized pestle and mortar in liquid nitrogen. Total RNA was extracted from the powdered tissue using an RNeasy Plant Mini kit (Qiagen, Valencia, CA, USA) and was then reverse-transcribed to cDNA using iScript Reverse Transcription Supermix (Bio-Rad, Hercules, CA, USA) according to the manufacturer's instructions. qRT-PCR was performed using the cDNA as template and SsoAdvanced SYBR Green Supermix (Bio-Rad) on a MyiQ Single-Color Real-Time PCR Detection system (Bio-Rad). The qRT-PCR was performed under the following conditions: 95°C for 30 s, followed by 45 cycles of 95°C for 10 s and 60°C for 30 s. Relative mRNA levels were calculated through normalization using 18S rRNA (X02623.1) abundance. Primer sequences were designed using Primer3 software (http://frodo.wi.mit.edu/primer3/input.htm) (Rozen and Skaletsky, 2000) and are listed in Supplemental Table 1.

### Statistical analysis

The statistical significance of the results was evaluated with one-way ANOVA followed by Duncan's multiple comparisons test, unless otherwise stated. All calculations were performed using SPSS software (version 22.0). A *p*-value of <0.05 was considered to be statistically significant.

## Results and discussion

### Effects of calcium on growth of soybean under flooding stress

**Figure 1:**
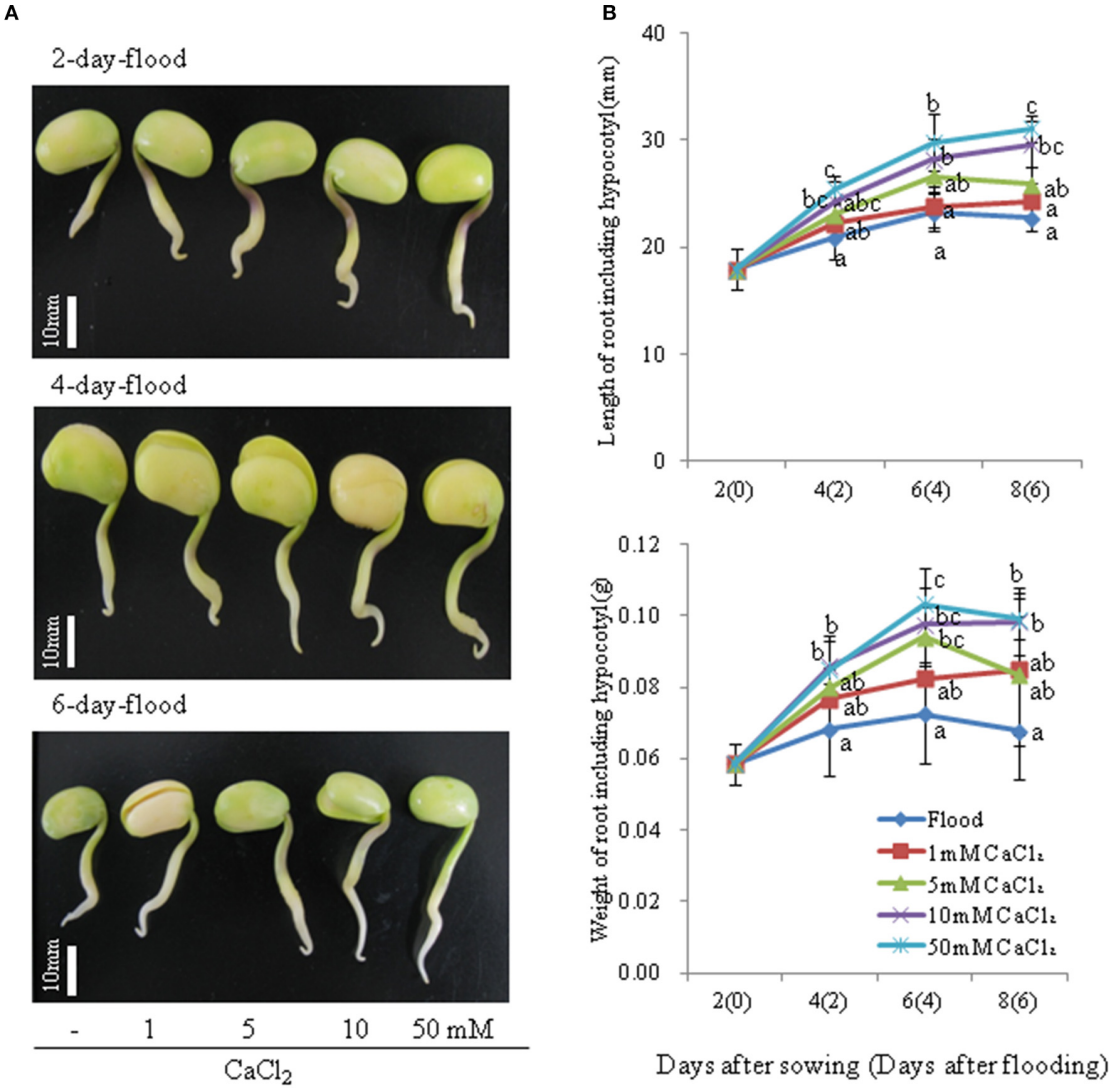
**Effect of calcium on growth of soybean under flooding stress**. Two-day-old soybeans were flooded without (dark blue) or with 1 (red), 5 (light green), 10 (purple), and 50 mM CaCl_2_ (light blue) for 2, 4, and 6 days. **(A)** Photographs show soybean seedlings after 2, 4, and 6 days of flooding. Bars indicate 10 mm. **(B)** Length and weight of roots, including the hypocotyl, were measured at the indicated time points. Data are means ± SE from three independent biological replications. Means with the same letter are not significantly different according to ANOVA (*P* < 0.05).

**Figure 2:**
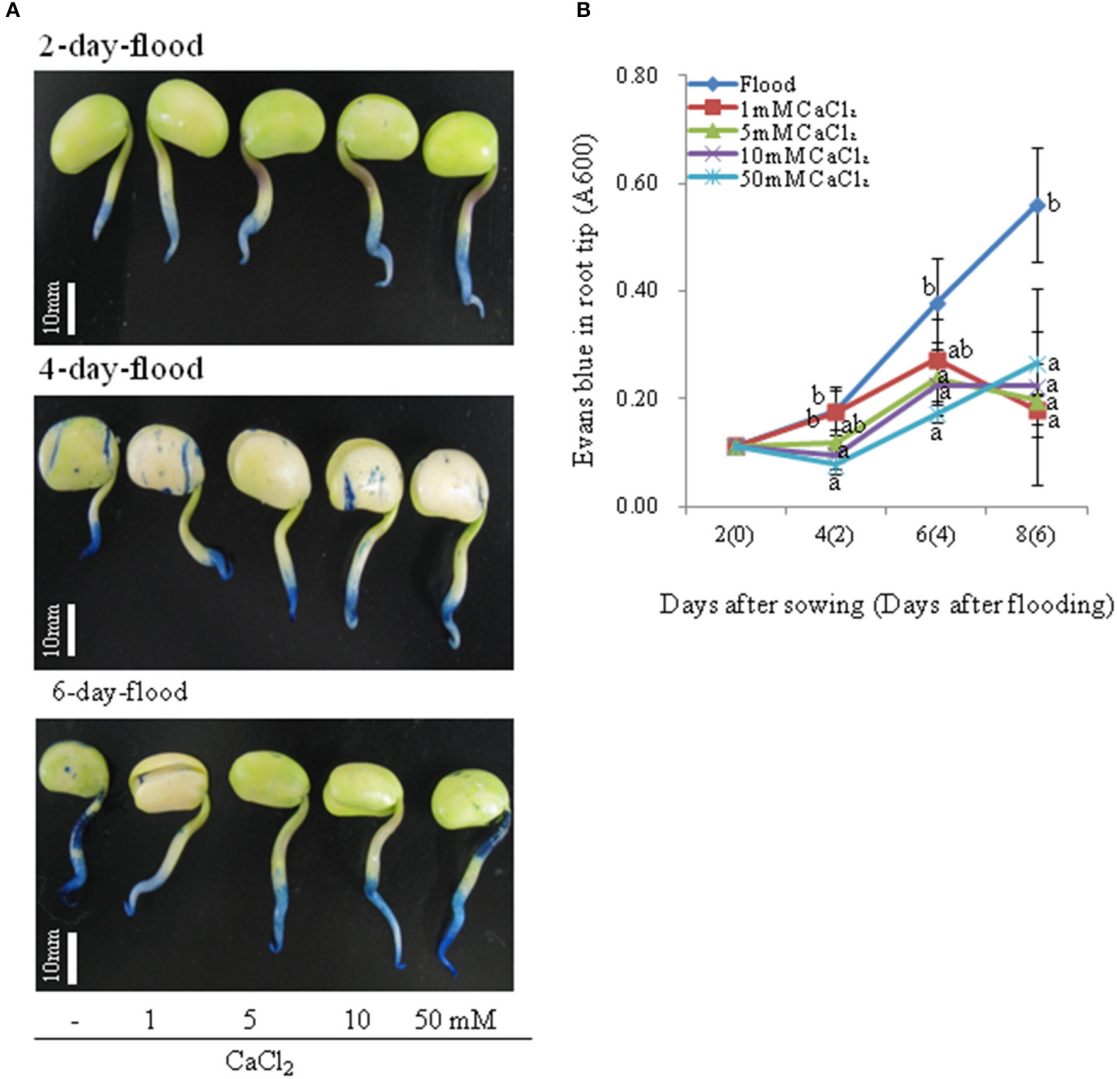
**Evaluation of cell death in flooding-stressed soybean root tips treated with calcium**. Two-day-old soybeans were flooded without (dark blue) or with 1 (red), 5 (light green), 10 (purple), and 50 mM CaCl_2_ (light blue) for 2, 4, and 6 days. The roots were stained with 0.25% Evans blue dye, which was then extracted and measured spectroscopically at 600 nm. **(A)** Photographs show soybeans after 2, 4, and 6 days of flooding. Bars indicate 10 mm. **(B)** Absorbance of Evans blue in root tips at the indicated time points. Data are means ± SE from three independent biological replications. Means with the same letter are not significantly different according to ANOVA (*P* < 0.05).

To investigate the effects of calcium on morphological changes induced by flooding in soybean and evaluate the corresponding degree of root tip damage, 2-day-old soybeans were flooded for 2, 4, and 6 days without or with 1, 5, 10, and 50 mM CaCl_2_. The total length of root including hypocotyl under flooding with various concentrations of CaCl_2_ was clearly longer than that under flooding without CaCl_2_ (Figure 1A). Although no marked differences in root length were detected between flooded soybean in the presence or absence of 1 mM CaCl_2_, the length after treatment with 10 and 50 mM CaCl_2_ was significantly longer than that under flooding without CaCl_2_ (Figure 1B). Consistent with this finding, the root weight of flooding-treated soybeans was also higher for plants exposed to CaCl_2_ during the treatment period (Figure 1B). The length and weight of root including hypocotyl were measured after each treatment in biological triplicates to assess reproducibility (Supplemental Figures 2A–C). The length and weight of root were gradually increased in 2-, 4-, and 6-day flooded soybean with 10 and 50 mM CaCl_2_. In particularly, 2-day-flooded soybean was most affected by CaCl_2_ (Figure 1). The flooding-treated soybeans were also stained with Evans blue dye to evaluate cell death (Figure 2). The degree of staining in root tips was dependent on the CaCl_2_ concentration and treatment period (Figure 2A). Cell death in the root tip was severely induced by flooding without CaCl_2_ compared to that in the presence of 50 mM CaCl_2_ (Figure 2B). The Evans blue staining was also performed in biological triplicate experiments to assess reproducibility (Supplemental Figures 3A–C). Even though cell death in the root tip was significantly suppressed by flooding with CaCl_2_ in 4 and 6 days, the degree of staining in root tips was most affected by CaCl_2_ in 2-day-flooded soybean (Figure 2).

Nanjo et al. (2013) reported that the amount of Evans blue uptake in the root tip region under 3 days flooding, which is evidence of cell death (Delisle et al., 2001), affected by the volume of floodwater and suggested that the loss of root tips in flooded soybean seedlings is due to flood-induced cell death. Consistent with this speculation, Evans blue uptake was markedly induced in soybean seedlings by 4 days flooding (Komatsu et al., 2013c). In the present study, the application of 50 mM CaCl_2_ clearly promoted root elongation and suppressed root tip cell death in soybeans during 6 days flooding stress, in particularly, in 2 days flooding treatment with CaCl_2_. Taken together, these findings indicate that flooding-induced root tip damage might be suppressed by exogenous calcium treatment. Based on the present results, for subsequent proteomic and qRT-PCR analyses, 50 mM CaCl_2_ was selected for the treatment in soybean under flooding.

### Protein profiles in flooding-stressed soybean root treated with calcium

**Figure 3:**
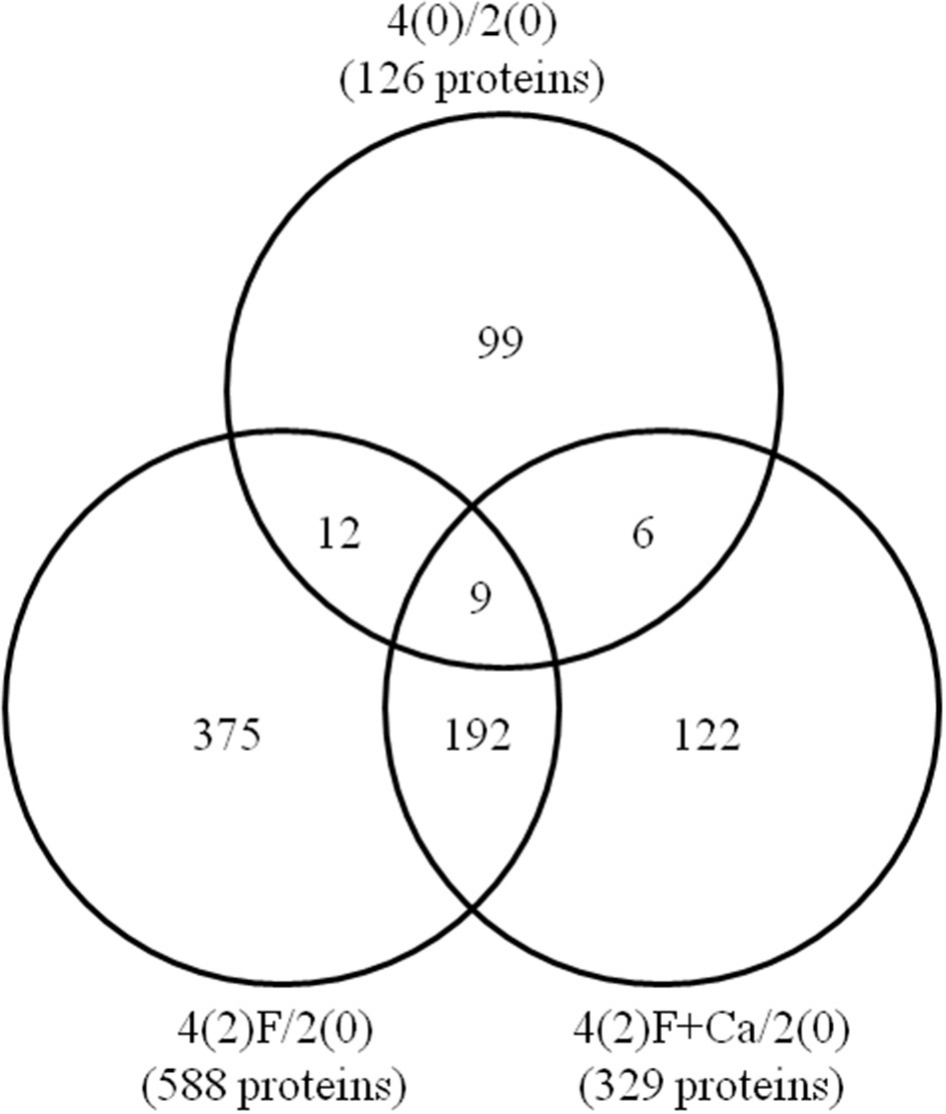
**Venn diagram of differentially changed proteins between control and flooded soybeans treated without or with calcium**. Two-day-old soybeans were flooded in the presence and absence of 50 mM CaCl_2_ for 2 days, and proteins extracted from roots were then analyzed by gel-free proteomics. The Venn diagram shows the number of differentially changed proteins in roots of 4-day-old soybeans without treatment [4(0)/2(0)] or with 2-day-flooding with [4(2)F+Ca/2(0)] and without CaCl_2_ [4(2)F/2(0)]. The overlapping regions denote common proteins among untreated and 2-day-flooded soybeans in the absence or presence of calcium. The numbers represent the number of identified proteins. The identified proteins are listed in Supplementary Tables 2–4.

To investigate the effect of calcium on protein profiles in soybean roots under flooding stress, a gel-free proteomic technique was used. Because 2-day-old soybean was considered as starting point of each treatment, 2-day-old soybean was used for comparison. Proteins were extracted from the roots of 4-day-old soybeans treated with flooded in absence or presence of 50 mM CaCl_2_ for 2 days and analyzed by nanoLC-MS/MS. Based on 3 biological replicated SIEVE analysis (Supplemental Figure 4), a total of 126 differentially changed proteins were identified in 4-day-old untreated soybeans (Figure 3, Supplemental Table 2), 588 such proteins were identified in 2-day-flooded soybeans (Figure 3, Supplemental Table 3), and 329 such proteins were identified in 2-day-flooded soybeans treated with calcium (Figure 3, Supplemental Table 4) compared to 2-day-old soybeans.

To determine whether the altered proteins were different in the absence or presence of exogenous calcium, the root proteins identified under each condition were compared (Figure 3). A total of 99, 375, and 122 differentially changed proteins were unique to 4-day-old soybeans, 2-day-flooded soybeans, and 2-day-flooded soybeans treated with calcium, respectively. In addition, 9 proteins were commonly changed among the three conditions; 12 proteins were common between 4-day-old soybeans and 2-day-flooded soybeans; 192 proteins were common between 2-day-flooded soybeans treated without and with calcium; and only 6 proteins were common between 4-day-old soybeans and 2-day-flooded soybeans treated with calcium. Among the 122 proteins that were specifically changed in response to calcium treatment (Table 1), the predominant functional categories were cell wall (15%), protein degradation/synthesis (13%), DNA (11%), and stress (10%)-related proteins. Notably, the identified proteins included xyloglucan endotransglucosylase/hydrolase (XTH) (11/18), which was categorized within cell-wall proteins, and several ribosomal proteins (9/16), which were grouped within the protein degradation/synthesis functional category.

**Table 1:** **Differentially changed proteins in soybean in response to calcium supplementation under flooding**.

	**Protein ID**	**Description**	**M.P**.	**Ratio**	***P*-value**	**Function**
**DECREASED**						
1	Glyma09g15620.2	Cellulose synthase family protein	4	0.13	0.0165	Cell wall
2	Glyma15g43040.1	Cellulose synthase family protein	4	0.13	0.0165	Cell wall
3	Glyma02g46450.3	Microtubule associated proteins 651	2	0.17	0.0009	Cell
4	Glyma14g02180.1	Microtubule associated proteins 651	2	0.17	0.0009	Cell
5	Glyma14g02200.2	Microtubule associated proteins 651	2	0.17	0.0009	Cell
6	Glyma19g27036.1	Heat shock protein 81-2	3	0.19	0.0079	Stress
7	Glyma03g25621.1	RNA binding (RRM/RBD/RNP motifs) family protein	2	0.22	0.0223	RNA
8	Glyma07g13211.1	RNA binding (RRM/RBD/RNP motifs) family protein	2	0.22	0.0223	RNA
9	Glyma12g31620.1	DNAJ homolog 2	5	0.25	0.0392	Stress
10	Glyma03g37240.4	RNA binding (RRM/RBD/RNP motifs) family protein	2	0.27	0.0185	RNA
11	Glyma03g37280.1	NAD(P) binding Rossmann fold superfamily protein	2	0.29	0.0163	Cell wall
12	Glyma16g04950.1	Xyloglucan endotransglucosylase/hydrolase 5	10	0.30	0.0000	Cell wall
13	Glyma19g28220.1	Xyloglucan endotransglucosylase/hydrolase 5	9	0.32	0.0000	Cell wall
14	Glyma04g01244.1	Thymidylate synthase 1	2	0.33	0.0086	C1
15	Glyma10g33350.2	*ARABIDOPSIS THALIANA* PEROXYGENASE 2	2	0.33	0.0302	Development
16	Glyma02g02170.1	NAD(P) binding Rossmann fold superfamily protein	3	0.33	0.0291	Cell wall
17	Glyma19g39870.1	NAD(P) binding Rossmann fold superfamily protein	3	0.33	0.0291	Cell wall
18	Glyma17g03030.1	NAD(P) binding Rossmann fold superfamily protein	2	0.34	0.0297	Cell wall
19	Glyma14g11760.1	Early nodulin like protein 15	2	0.34	0.0012	Misc
20	Glyma17g34040.1	Early nodulin like protein 15	2	0.34	0.0012	Misc
21	Glyma19g36580.1	Pyridoxal-dependent decarboxylase family protein	5	0.34	0.0009	Amino acid metabolism
22	Glyma18g52860.1	O Glycosyl hydrolases family 17 protein	4	0.34	0.0065	Misc
23	Glyma19g39850.1	RNA binding (RRM/RBD/RNP motifs) family protein	3	0.35	0.0047	RNA
24	Glyma03g02240.1	SIN3 associated polypeptide P18	2	0.36	0.0171	RNA
25	Glyma12g03570.1	Subtilisin like serine protease 2	6	0.38	0.0143	Protein
26	Glyma02g06400.1	Succinate dehydrogenase 11	3	0.39	0.0034	TCA/org transformation
27	Glyma15g03761.1	Leucine rich repeat (LRR) family protein	2	0.39	0.0326	Not assigned
28	Glyma02g07610.1	Xyloglucan endotransglucosylase/hydrolase 5	5	0.39	0.0000	Cell wall
29	Glyma06g10180.1	P450 reductase 1	2	0.39	0.0483	Misc
30	Glyma06g01280.2	Thymidylate synthase 2	2	0.39	0.0075	Nucleotide metabolism
31	Glyma18g53470.1	Rad23 UV excision repair protein family	3	0.40	0.0296	DNA
32	Glyma02g14450.1	Chalcone and stilbene synthase family protein	5	0.41	0.0004	Secondary metabolism
33	Glyma11g07250.1	Succinate dehydrogenase 11	6	0.43	0.0004	TCA/org transformation
34	Glyma01g38200.1	Succinate dehydrogenase 11	5	0.43	0.0008	TCA/org transformation
35	Glyma06g04100.2	RNA binding protein 47C	2	0.43	0.0190	RNA
36	Glyma07g12190.1	Hexokinase 1	4	0.44	0.0313	Major CHO metabolism
37	Glyma17g37270.2	Beta galactosidase 5	4	0.46	0.0023	Misc
38	Glyma14g07700.1	Beta galactosidase 5	2	0.47	0.0090	Misc
39	Glyma08g03730.1	Hexokinase 1	8	0.48	0.0181	Major CHO metabolism
40	Glyma11g33880.1	DegP protease 7	5	0.48	0.0027	Protein
41	Glyma18g04400.1	DegP protease 7	5	0.48	0.0027	Protein
42	Glyma01g22880.1	Chalcone and stilbene synthase family protein	6	0.49	0.0011	Secondary metabolism
43	Glyma05g28610.1	Chalcone and stilbene synthase family protein	6	0.49	0.0011	Secondary metabolism
44	Glyma08g11530.1	Chalcone and stilbene synthase family protein	6	0.49	0.0011	Secondary metabolism
45	Glyma08g11620.1	Chalcone and stilbene synthase family protein	6	0.49	0.0011	Secondary metabolism
46	Glyma09g08780.1	Chalcone and stilbene synthase family protein	6	0.49	0.0011	Secondary metabolism
47	Glyma11g01350.1	Chalcone and stilbene synthase family protein	6	0.49	0.0011	Secondary metabolism
48	Glyma08g11610.1	Chalcone and stilbene synthase family protein	5	0.49	0.0016	Secondary metabolism
49	Glyma02g35640.1	CTC interacting domain 11	2	0.49	0.0052	RNA
**INCREASED**						
50	Glyma19g31590.1	Beta-1,3-glucanase 1	4	2.05	0.0403	Misc
51	Glyma09g00711.1	Glycine cleavage T protein family	3	2.06	0.0390	Photosynthesis
52	Glyma11g14950.1	Heat shock protein 70	25	2.22	0.0017	Stress
53	Glyma08g36700.1	ARF-GAP domain 8	6	2.26	0.0012	Protein
54	Glyma17g14950.1	Lactate/malate dehydrogenase family protein	4	2.28	0.0009	Fermentation
55	Glyma03g02760.1	N.D.^*^	6	2.29	0.0000	Not assigned
56	Glyma15g07850.3	Aluminum induced protein with YGL and LRDR motifs	2	2.30	0.0347	Hormone metabolism
57	Glyma18g52650.2	Heat shock cognate protein 70-1	22	2.31	0.0000	Stress
58	Glyma18g52610.1	Heat shock cognate protein 70-1	25	2.31	0.0046	Stress
59	Glyma13g19331.1	Heat shock cognate protein 70-1	26	2.32	0.0002	Stress
60	Glyma02g41890.3	Type one serine/threonine protein phosphatase 4	3	2.33	0.0039	Protein
61	Glyma14g07080.3	Type one serine/threonine protein phosphatase 4	3	2.33	0.0039	Protein
62	Glyma01g41920.2	Lactate/malate dehydrogenase family protein	3	2.33	0.0019	Fermentation
63	Glyma13g40890.1	Histone H2A 12	2	2.33	0.0002	DNA
64	Glyma13g40900.1	Histone H2A 12	2	2.33	0.0002	DNA
65	Glyma13g40940.1	Histone H2A 12	2	2.33	0.0002	DNA
66	Glyma15g04530.1	Histone H2A 12	2	2.33	0.0002	DNA
67	Glyma15g04540.1	Histone H2A 12	2	2.33	0.0002	DNA
68	Glyma03g34830.1	Enolase	19	2.46	0.0016	Glycolysis
69	Glyma02g04800.1	Calcium dependent phosphotriesterase superfamily protein	2	2.58	0.0371	Secondary metabolism
70	Glyma16g22650.1	Calcium dependent phosphotriesterase superfamily protein	2	2.58	0.0371	Secondary metabolism
71	Glyma17g29320.1	Peroxidase family protein	2	2.63	0.0199	Misc
72	Glyma09g06350.1	Peroxidase superfamily protein	5	2.65	0.0034	Misc
73	Glyma19g42760.1	Gamma histone variant H2AX	3	2.72	0.0000	DNA
74	Glyma20g28460.1	Cupin family protein	2	2.72	0.0006	Development
75	Glyma20g28640.1	Cupin family protein	2	2.72	0.0006	Development
76	Glyma04g38590.1	Beta galactosidase 10	6	2.77	0.0011	Misc
77	Glyma06g16420.2	Beta galactosidase 10	5	2.78	0.0020	Misc
78	Glyma02g00850.3	Type one serine/threonine protein phosphatase 4	2	2.79	0.0012	Protein
79	Glyma05g33410.2	Aldolase-type TIM barrel family protein	2	2.84	0.0077	OPP
80	Glyma19g34780.1	RmlC like cupins superfamily protein	3	2.91	0.0287	Developmen
81	Glyma19g32690.1	Ribosomal protein S10p/S20e family protein	4	2.95	0.0335	Protein
82	Glyma03g30440.1	Histone superfamily protein	2	2.99	0.0000	DNA
83	Glyma12g34360.1	Histone H2A 10	2	2.99	0.0000	DNA
84	Glyma12g34370.2	Histone H2A 2	2	2.99	0.0000	DNA
85	Glyma13g36180.1	Histone H2A 10	2	2.99	0.0000	DNA
86	Glyma13g36190.1	Histone H2A 10	2	2.99	0.0000	DNA
87	Glyma19g33360.1	Gamma histone variant H2AX	2	2.99	0.0000	DNA
88	Glyma17g03360.1	N.D.^*^	7	3.03	0.0000	Not assigned
89	Glyma02g47210.1	HEAT SHOCK PROTEIN 81.4	16	3.11	0.0008	Stress
90	Glyma03g32380.2	Ribosomal protein L14p/L23e family protein	6	3.14	0.0025	Protein
91	Glyma13g18830.1	Ribosomal protein L14p/L23e family protein	6	3.14	0.0025	Protein
92	Glyma14g01530.1	HEAT SHOCK PROTEIN 81.4	17	3.25	0.0010	Stress
93	Glyma14g04840.1	Ribosomal protein S10p/S20e family protein	3	3.34	0.0309	Protein
94	Glyma19g32680.1	Ribosomal protein S10p/S20e family protein	3	3.34	0.0309	Protein
95	Glyma08g44590.1	HEAT SHOCK PROTEIN 81.4	16	3.71	0.0014	Stress
96	Glyma03g28870.1	Beta-1,3-glucanase 1	3	3.87	0.0415	Misc
97	Glyma13g01140.1	Xyloglucan endotransglucosylase/hydrolase family protein	6	3.94	0.0005	Cell wall
98	Glyma18g08220.1	HEAT SHOCK PROTEIN 81.4	16	4.02	0.0001	Stress
99	Glyma08g11300.2	Xyloglucan endotransglucosylase/hydrolase 16	3	4.05	0.0003	Cell wall
100	Glyma11g36730.2	Xyloglucan endotransglucosylase/hydrolase 16	3	4.05	0.0003	Cell wall
101	Glyma18g00630.2	Xyloglucan endotransglucosylase/hydrolase 16	3	4.05	0.0003	Cell wall
102	Glyma15g17620.1	Peroxidase superfamily protein	5	4.14	0.0012	Misc
103	Glyma13g01120.1	Xyloglucan endotransglycosylase 6	3	4.14	0.0027	Cell wall
104	Glyma17g07240.1	Xyloglucan endotransglycosylase 6	3	4.14	0.0027	Cell wall
105	Glyma13g01150.1	Xyloglucan endotransglucosylase/hydrolase family protein	3	4.18	0.0045	Cell wall
106	Glyma17g07270.1	Xyloglucan endotransglycosylase 6	3	4.18	0.0045	Cell wall
107	Glyma19g31580.1	beta-1,3-glucanase 1	2	4.82	0.0205	Misc
108	Glyma13g34540.1	D mannose binding lectin protein with Apple like carbohydrate binding domain	3	5.24	0.0291	Misc
109	Glyma03g32030.1	RmlC like cupins superfamily protein	5	5.35	0.0160	Development
110	Glyma07g02720.1	Ribosomal protein L7Ae/L30e/S12e/Gadd45 family protein	7	6.05	0.0034	Protein
111	Glyma08g23260.3	Ribosomal protein L7Ae/L30e/S12e/Gadd45 family protein	7	6.06	0.0033	Protein
112	Glyma13g44690.1	Ribosomal protein L7Ae/L30e/S12e/Gadd45 family protein	7	6.06	0.0033	Protein
113	Glyma15g00610.1	Ribosomal protein L7Ae/L30e/S12e/Gadd45 family protein	7	6.06	0.0033	Protein
114	Glyma13g32300.1	Quinone reductase family protein	6	6.14	0.0133	Lipid metabolism
115	Glyma15g07040.1	Quinone reductase family protein	6	6.14	0.0133	Lipid metabolism
116	Glyma08g45510.1	Kunitz family trypsin and protease inhibitor protein	3	6.65	0.0161	Stress
117	Glyma04g39930.1	Manganese superoxide dismutase 1	4	7.27	0.0490	Redox
118	Glyma09g30370.1	Glutamine synthase clone R1	8	7.86	0.0038	N-metabolism
119	Glyma03g22260.1	Auxin responsive family protein	2	11.51	0.0081	Hormone metabolism
120	Glyma08g06570.1	Flavodoxin like quinone reductase 1	2	14.54	0.0044	Lipid metabolism
121	Glyma04g37140.1	SNF1 related protein kinase regulatory subunit gamma 1	2	14.59	0.0493	Cell wall
122	Glyma09g29300.1	Kunitz family trypsin and protease inhibitor protein	7	22.58	0.0124	Stress

Protein ID, according to the Phytozome database; M.P., number of matched peptide; Ratio, relative abundance of protein; Function, functional classification by MapMan bin code;

* N.D., No description in Phytozome database; protein, protein synthesis/targeting/degradation/post-translational modification; DNA, DNA synthesis; RNA, RNA processing/binding; C1, one carbon; TCA, tricarboxylic acid; OPP, oxidative pentose phosphate; CHO, carbohydrates; misc, miscellaneous.

In salt-stressed soybean, exogenous calcium treatment restored root growth by maintaining pectin levels and increasing the calcium concentration in the cell wall, suggesting that calcium plays a role in maintaining cell wall composition to protect from salt toxicity (An et al., 2014). XTH, which acts as a cell wall-loosening enzyme (van Sandt et al., 2007), functions in cell-wall elongation and reconstruction through rearranging the bonds between xyloglucan chains (Fry et al., 1992; Nishitani and Tominaga, 1992) and also involves in cell-wall metabolism during flooding-induced aerenchyma development. In *Capsicum annuum*, increased tolerance to drought stress by overexpression of CaXTH3 resulted from the change of cell-wall extensibility of guard cells mediated by the cell-wall remodeling activity of CaXTH3 (Choi et al., 2011), corresponding with increased abundance of XTH in present study. In mungbean, expression of VrXTH1, which is an auxin-inducible gene isolated from mungbean, was closely related to plant growth and modulated by the cytosolic calcium concentration (Yun et al., 2007). Furthermore, changes in calcium ion levels influenced the molecular size of xyloglucans by modifying the expression of VaXTHS4 in azuki bean (Soga et al., 2007). Taken together, these results suggest that the regulation of cell wall-related proteins, such as XTH, by exogenous calcium might promote the elongation of soybean roots under flooding stress. In this study, except for XTH, other cell wall-related proteins such as glycosyl hydrolase, which was decreased in flood-stressed soybean, was exhibited a similar tendency in previous study (Oh et al., 2014a).

A number of ribosomal proteins play roles in cell metabolism/division, and plant growth and fitness (Whittle and Krochko, 2009). Ribosomal proteins are generally decreased in plants in response to abiotic stresses (Rogalski et al., 2008; Falcone Ferreyra et al., 2010), leading to retarded growth and productivity (Kawaguchi et al., 2004). Ribosomal L19 protein, which has 60S ribosomal subunit, is a calcium-calmodulin interacting protein implicated in translational processes (Sonnemann et al., 1991). Mutation of the RPS6A gene, which encodes a component of the small ribosomal 40S subunit (Zhao et al., 2003), led to higher intracellular calcium concentrations in response to exogenous calcium (Zhao et al., 2013). Flooding stress also caused a decrease in the abundance of ribosomal proteins in soybean, indicating that ribosomal proteins resulted in alterations of protein synthesis (Oh et al., 2014b). In present study, ribosomal proteins were increased in soybean roots exposed to exogenous calcium under flooding stress, suggesting that ribosomal proteins in association with calcium-dependent proteins and/or molecules might enhance protein synthesis in response to flooding stress.

### Functional categorization of proteins in calcium-treated soybean root under flooding

**Figure 4:**
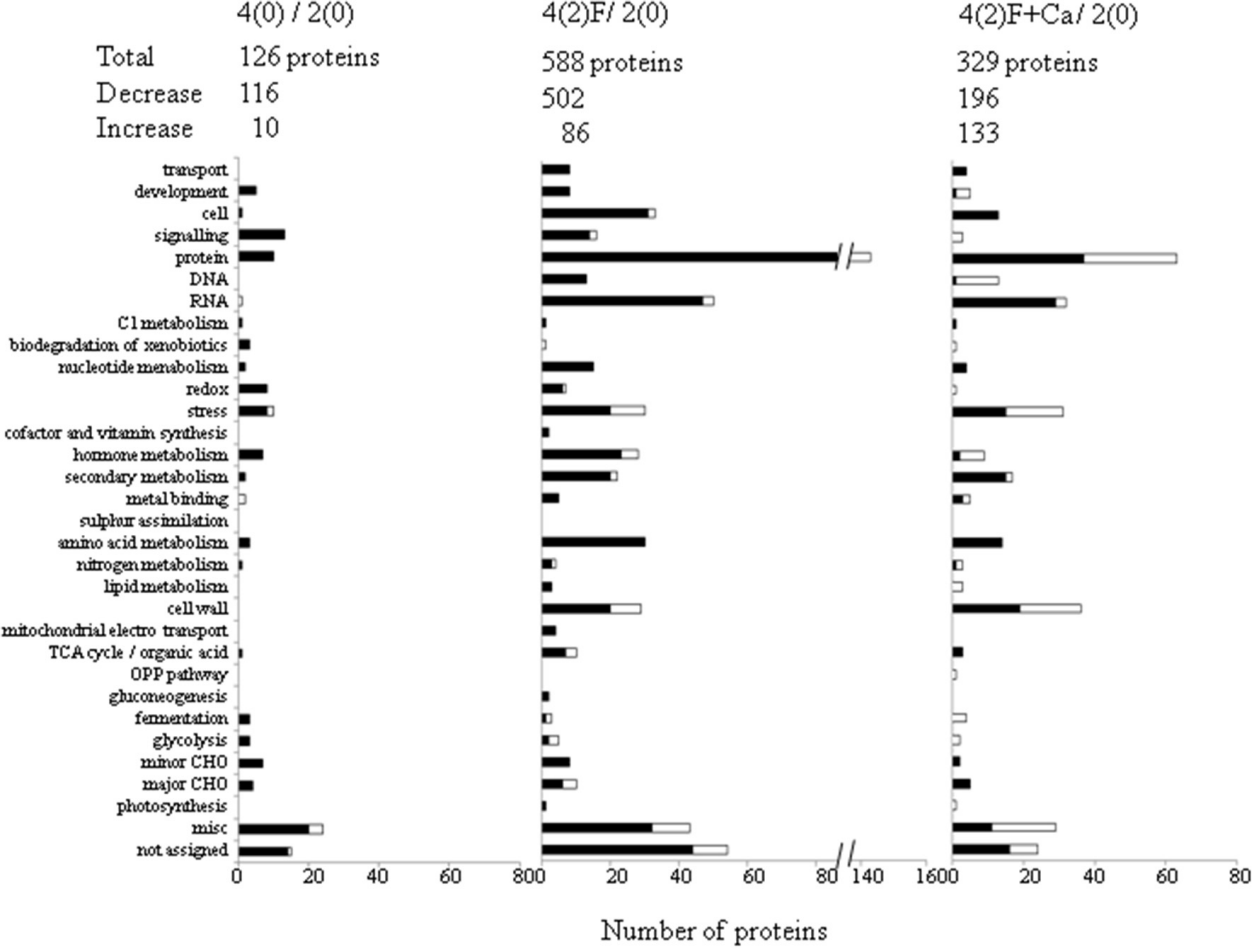
**Functional categorization of flooding-responsive proteins in soybean roots treated with calcium**. Two-day-old soybeans were flooded without or with 50 mM CaCl_2_ for 2 days. Untreated soybeans were used as control. Proteins extracted from roots were analyzed using gel-free proteomics. The following comparisons were made: 4(0)/2(0), 2-day-old soybeans and 4-day-old soybeans; 4(2)F/2(0), 2-day-old soybeans and 2-day-flooded soybeans without calcium; and 4(2)F+Ca/2(0), 2-day-old soybeans and 2-day-flooded soybeans treated with calcium. Identified proteins were categorized using MapMan bin codes: protein, protein synthesis/targeting/degradation/post-translational modification; DNA, DNA synthesis; RNA, RNA processing/binding; C1, one carbon; TCA, tricarboxylic acid; OPP, oxidative pentose phosphate; CHO, carbohydrates; misc, miscellaneous. The number of differentially changed proteins is indicated on the x-axis of the graph. Black and white bars indicate decreased and increased proteins, respectively.

To determine the biological processes of proteins that were altered in flooding-stressed soybean roots by calcium treatment, the identified proteins were functionally characterized using MapMan bin codes (Figure 4). Under flooding stress, the number of proteins related to hormone metabolism, DNA synthesis, cell wall, and protein degradation/synthesis/posttranslational modification was decreased; however, the exposure of plants to calcium under flooding conditions increased the number of these proteins. Similarly, the number of proteins related to development, signaling, and lipid metabolism was increased when exogenous calcium was added to the roots of flooding-stressed plants. In addition, the number of proteins related to fermentation and glycolysis was increased under flooding stress; however, calcium supplementation had little effect on the number of differentially changed proteins (Figure 4). Proteins within the hormone category that were changed in response to flooding in absence or presence of calcium included lipoxygenase and auxin-responsive family protein.

Under flooding stress, most lipoxygenases were decreased in soybean roots and were not induced by calcium supplementation. Lipoxygenases, which are a group of non-heme iron-containing dioxygenases (Brash, 1999), are involved in increasing stress resistance and boosting defense reactions in both *Arabidopsis* and soybean (Melan et al., 1994; Park et al., 1994). Komatsu et al. (2010) reported that two lipoxygenases were decreased in soybean in response to flooding stress and suggested that these enzymes affected cell wall metabolism due to suppression of lignification. The present findings indicate that lipoxygenase may regulate hormone pathways for stress tolerance and involve in defense reaction in soybean roots under flooding stress.

Auxin-responsive family protein was increased under flooding conditions in soybean roots treated with calcium compared to non-treated roots. In *Arabidopsis*, the gene encoding auxin-responsive family protein was expressed during the early stages of lateral root formation (Laskowski et al., 2006), and the corresponding protein interacted with other redox partners within the plasma membrane to form a redox link between the cytoplasm and the apoplast (Preger et al., 2009). Auxin-responsive family protein was also shown to be glycosylated in *Arabidopsis* and involved in the formation of a glycosylphosphatidylinositol anchor to the external side of the plasma membrane (Borner et al., 2003). As calcium ions have an important role in polar auxin transport and gravitropic responses (Toyota et al., 2008), together, these results indicate that calcium supplementation may control hormone metabolic pathways by regulating activation of these proteins in soybean under flooding stress.

In the present study, DNA synthesis-related proteins, include histone, were decreased by flooding stress, but were increased by the treatment of soybean roots with calcium. Histones, which are small and basic proteins associated with DNA to form chromatin (Thuleau et al., 2012), undergo posttranslational modification, such as acetylation/deacetylation. Epigenetic mechanisms, including histone modifications, play a decisive role in regulating plant responses to abiotic stresses (Luo et al., 2012). Servet et al. (2010) reported that histone acetyltransferase AtGCN5/HAG1 in *Arabidopsis* was essential to regulate gene expression during development processes and in response to environmental stresses. In *Nicotiana tabacum*, type-2 histone deacetylases act as negative regulators of programmed cell death induced by the defense elicitor cryptogein (Bourque et al., 2011). The results of an Evans blue assay indicated that flooding-induced root tip cell death of soybean was suppressed by exogenous calcium. It has also been reported that histone modifications were activated by a transient influx of calcium in response to heat (Mach, 2012). Based on these results, calcium-induced stabilization of DNA synthesis via histone modification may be a critical plant defense response to flooding stress in soybean.

### Effects of calcium on mRNA expression level of key proteins in different organs under flooding stress

**Figure 5:**
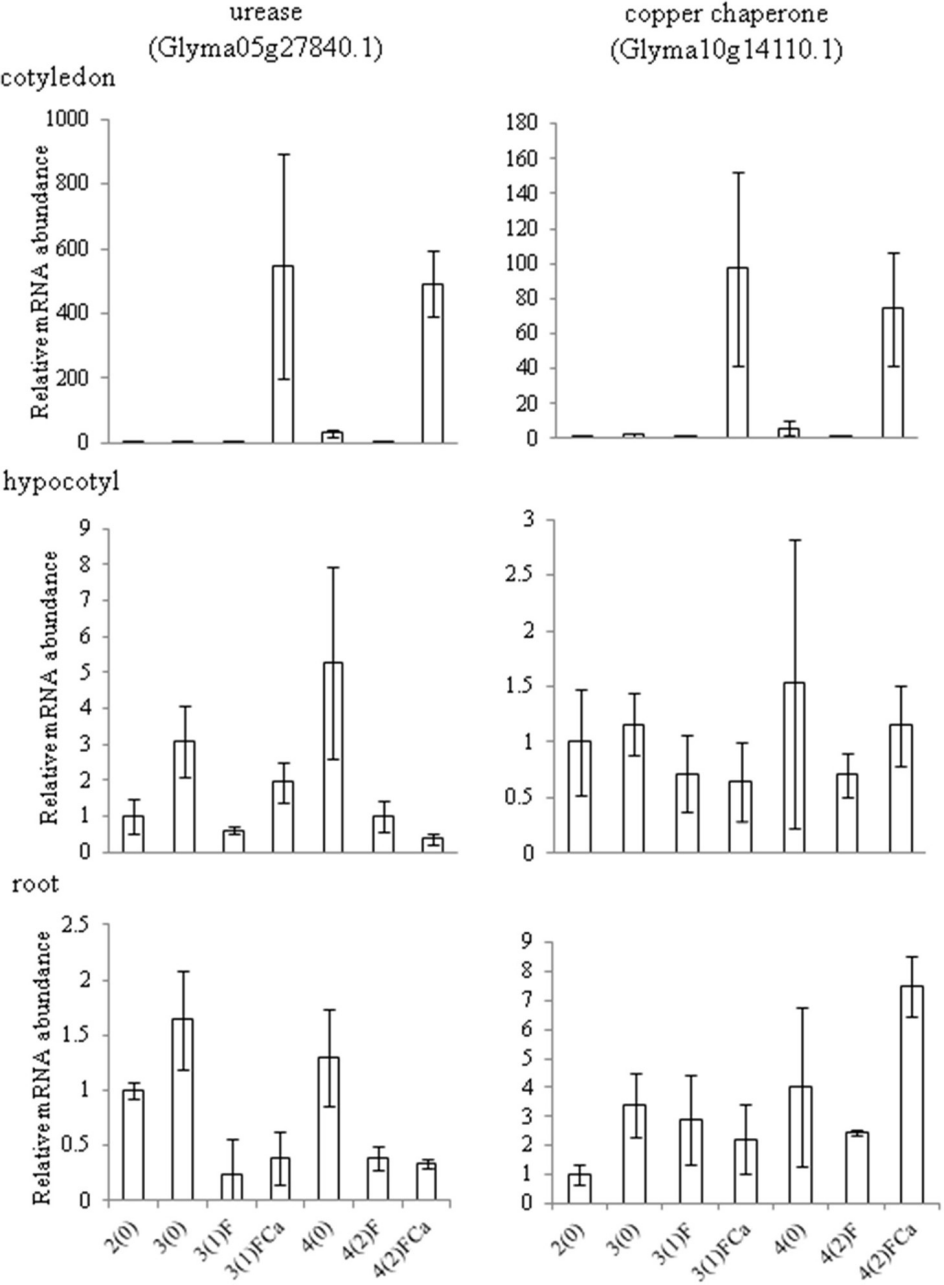
**Effects of calcium on the mRNA expression levels of urease and copper chaperone proteins in different organs of soybean under flooding stress**. Two-day-old soybeans were flooded without or with 50 mM CaCl_2_ for 1 and 2 days. Untreated soybeans were used as a control. RNAs extracted from roots, hypocotyls, and cotyledons of the soybeans were analyzed by qRT-PCR with specific primers for urease and copper chaperone (Supplemental Table 1). Relative mRNA abundance was normalized against that of 18S rRNA. Data are shown as means ± SD from three independent biological replicates. Means with the same letter are not significantly different according to ANOVA (*P* < 0.05).

Six differentially changed proteins were common between 4-day-old soybeans and 2-day-flooded soybeans treated with calcium (Figure 3). The abundance of these proteins was calculated among 4-day-old soybeans, 2-day-flooded soybeans, and 2-day-flooded soybeans treated with 50 mM CaCl_2_ (Supplemental Figure 5). The analysis of protein abundance indicated that urease (Glyma05g27840.1) and two copper chaperones (Glyma10g14110.1 and Glyma02g19380.1) exhibited the same profiles in 4-day-old soybeans and 2-day-flooded soybeans treated with 50 mM CaCl_2_. To determine whether the changes in protein abundance were regulated at the transcriptional level, the mRNA expression levels of these proteins were analyzed in root, hypocotyl, and cotyledon under flooding for 0, 1, and 2 days (Figure 5). Total RNAs extracted from roots, hypocotyls, and cotyledons of soybeans were analyzed using qRT-PCR. In roots, the mRNA level of urease was down-regulated by 1 and 2 days flooding; however, the level was not changed by calcium supplementation. In hypocotyls, the mRNA level of urease was down-regulated by 1 day of flooding and significantly up-regulated by 1 day flooding with calcium; however, there was no significant between 2 days flooding without and with calcium. The mRNA expression level of copper chaperone was similar to their protein level in the root and hypocotyl under 2 days flooding with calcium. In roots and hypocotyls, the level of copper chaperone was down-regulated by 1 day flooding without and with calcium; whereas the level was significantly up-regulated by 2 days flooding with calcium. In cotyledons, the mRNA levels of urease and copper chaperone were significantly up-regulated by 1 and 2 day flooding when calcium was exogenously added (Figure 5).

Urease is a nickel-dependent metalloenzyme that catalyzes the hydrolysis of urea to form ammonia and carbon dioxide (Sirko and Brodzik, 2000). Plants typically have abundant urease (Yata et al., 2014), particularly in seedlings, in which it plays a pivotal role in nitrogen metabolism (Sirko and Brodzik, 2000). It has been reported that plant ureases are associated with plant defense mechanisms against insect predation (Follmer et al., 2004). In soybean, a urease was identified in developing embryos and was designated embryo-specific urease, which was later found to be encoded by the *Eu1* gene (Meyer-Bothling and Polacco, 1987; Torisky et al., 1994; Polacco et al., 2011). The suppression of embryo-specific urease in soybean led to increased susceptibility to fungal infection, demonstrating that urease plays a role in plant defense (Wiebke-strohm et al., 2012). Medeiros-Silva et al. (2014) reported that a urease deficiency in soybean altered the physiology of root nodules and adversely affected nitrogen fixation. Consistent with our present findings, Jack bean urease activity was affected by intra- and extracellular calcium concentrations (Staniscuaski et al., 2009). These results, together with the present findings, suggest that flooding stress decreases the urease activity in soybean, and that exogenously applied calcium appears to increase urease activity under flooding stress.

Copper chaperone has a copper-binding motif and plays a role in the homeostatic regulation of copper within plant cells (Mira et al., 2001). In *Arabidopsis*, copper chaperone was up-regulated during leaf senescence, suggesting that it facilitates the transport of certain metal ions in leaves to other growing parts of the plant (Himelblau et al., 1998; Mira et al., 2001). In tomato, the copper chaperone gene, which encodes a copper chaperone for copper/zinc superoxide dismutase, is involved in defense mechanisms against oxidative stress (Company and Carmen, 2003). In pea, the copper/zinc superoxide dismutase gene was down-regulated when calcium deficiency was induced by heavy metal stress with high-concentration of cadmium (Rodriquez-Serrano et al., 2009). Copper chaperone plays a role in oxidant-responsive posttranslational regulation of superoxide dismutase activity in yeast (Brown et al., 2004). In poplar, copper chaperone specifically responds to certain metals and oxidative damage caused by abiotic stresses (Lee et al., 2005). In the present study, the copper chaperone gene was up-regulated in soybean roots by exogenous calcium under flooding stress, suggesting that this chaperone protein is one of the factors regulating flooding stress responses in a calcium-dependent manner in soybean root.

## Concluding remarks

In the present study, proteins affected by calcium in flooded soybean were analyzed to better understand calcium-mediated flooding stress mechanisms in this agriculturally important crop. These main findings of this study are as follows: (i) cell wall, protein degradation/synthesis, hormone metabolism, and DNA synthesis-related proteins were decreased under flooding stress, but were increased by the addition of calcium; (ii) development, lipid metabolism, and signaling-related proteins were increased by calcium addition; (iii) fermentation- and glycolysis-related proteins were increased under flooding stress and were not affected by calcium addition; and (iv) urease and copper chaperone had similar abundances in untreated soybeans and flooded soybeans treated with calcium. Taken together, these results suggest that calcium might affect the cell wall/hormone metabolisms, protein degradation/synthesis, and DNA synthesis in soybean roots under flooding stress.

### Conflict of interest statement

The authors declare that the research was conducted in the absence of any commercial or financial relationships that could be construed as a potential conflict of interest.

## Supplementary material

Supplemental Figure 1 **Experimental design for this study**. **(A)** For physiological analysis, 2-day-old soybeans were flooded without or with 1, 5, 10, and 50 mM CaCl_2_ for 2, 4, and 6 days. **(B)** For proteomic analysis, 2-day-old soybeans were flooded without or with 50 mM CaCl_2_ for 2 days. Two-day-old and 4-day-old soybeans without flooding were used as controls. **(C)** For transcriptional analysis, 2-day-old soybeans were flooded without or with 50 mM CaCl_2_ for 1 and 2 days. Two-day-old, 3-day-old, and 4-day-old soybeans without flooding were used as controls. Three independent experiments were performed as biological replicates.

Supplemental Figure 2 **Effect of calcium on the growth of soybean under flooding stress in three independent experiments (A–C)**. Two-day-old soybeans were flooded without (dark blue) or with 1 (red), 5 (light green), 10 (purple), and 50 mM CaCl_2_ (light blue) for 2, 4, and 6 days. Photographs show soybean seedlings after 2, 4, and 6 days of flooding. Bars indicate 10 mm. The length and weight of roots, including the hypocotyl, were measured at the indicated time points. Data are means ± SE from three independent biological replications **(A–C)**. Means with the same letter are not significantly different according to ANOVA (*P* < 0.05).

Supplemental Figure 3 **Evaluation of cell death in flooding-stressed soybean roots treated with calcium in three independent experiments (A–C)**. Two-day-old soybeans were flooded without (dark blue) or with 1 (red), 5 (light green), 10 (purple), and 50 mM CaCl_2_ (light blue) for 2, 4, and 6 days. The root tips were stained with 0.25% Evans blue dye, which was then extracted and measured spectroscopically at 600 nm at the indicated time points. Photographs show soybean seedlings after 2, 4, and 6 days of flooding. Bars indicate 10 mm. Data are means ± SE from three independent biological replications **(A–C)**. Means with the same letter are not significantly different according to ANOVA (*P* < 0.05).

Supplemental Figure 4 **Principle component analysis of the peptide profile for identified proteins**. The data sets of soybean proteins from 2-day-old (blue circle), 4-day-old (red square), 4-day-old with 2-day-flood (yellow triangle), and 4-day-old with 2-day-flood with calcium (purple pentagon) were plotted using principle component analysis (PCA). PCA was fully integrated into SIEVE software. PCA loading plot shows the spectral regions responsible for separation of groups in the corresponding score plot.

Supplemental Figure 5 **Abundance of six differentially changed proteins that were common between untreated and flooding-stressed soybean exposed to calcium**. Two-day-old soybeans were flooded without or with 50 mM CaCl_2_ for 2 days. Proteins extracted from roots were purified, digested, and analyzed using nanoLC MS/MS. The protein abundance was determined by differential analysis using SIEVE software. Six differentially changed proteins were commonly identified in 4-day-old soybeans and 2-day-flooded soybeans treated with calcium.

## Abbreviations

LC: liquid chromatography
MS: mass spectrometry
qRT-PCR: quantitative reverse transcription-polymerase chain reaction
XTH: xyloglucan endotransglucosylase/hydrolase.
