# Supplementary material for: Gel-free proteomic analysis of soybean root proteins affected by calcium under flooding stress
Source: Front Plant Sci. 2014 Oct 20;5:559. doi: 10.3389/fpls.2014.00559 (PMC4202786; doi:10.3389/fpls.2014.00559)
Supplement: Supplemental Figure 1 — Experimental design for this study. (A) For physiological analysis, 2-day-old soybeans were flooded without or with 1, 5, 10, and 50 mM CaCl2 for 2, 4, and 6 days. (B) For proteomic analysis, 2-day-old soybeans were flooded without or with 50 mM CaCl2 for 2 days. Two-day-old and 4-day-old soybeans without flooding were used as controls. (C) For transcriptional analysis, 2-day-old soybeans were flooded without or with 50 mM CaCl2 for 1 and 2 days. Two-day-old, 3-day-old, and 4-day-old soybeans without flooding were used as controls. Three independent experiments were performed as biological replicates. [file Image1.PDF]

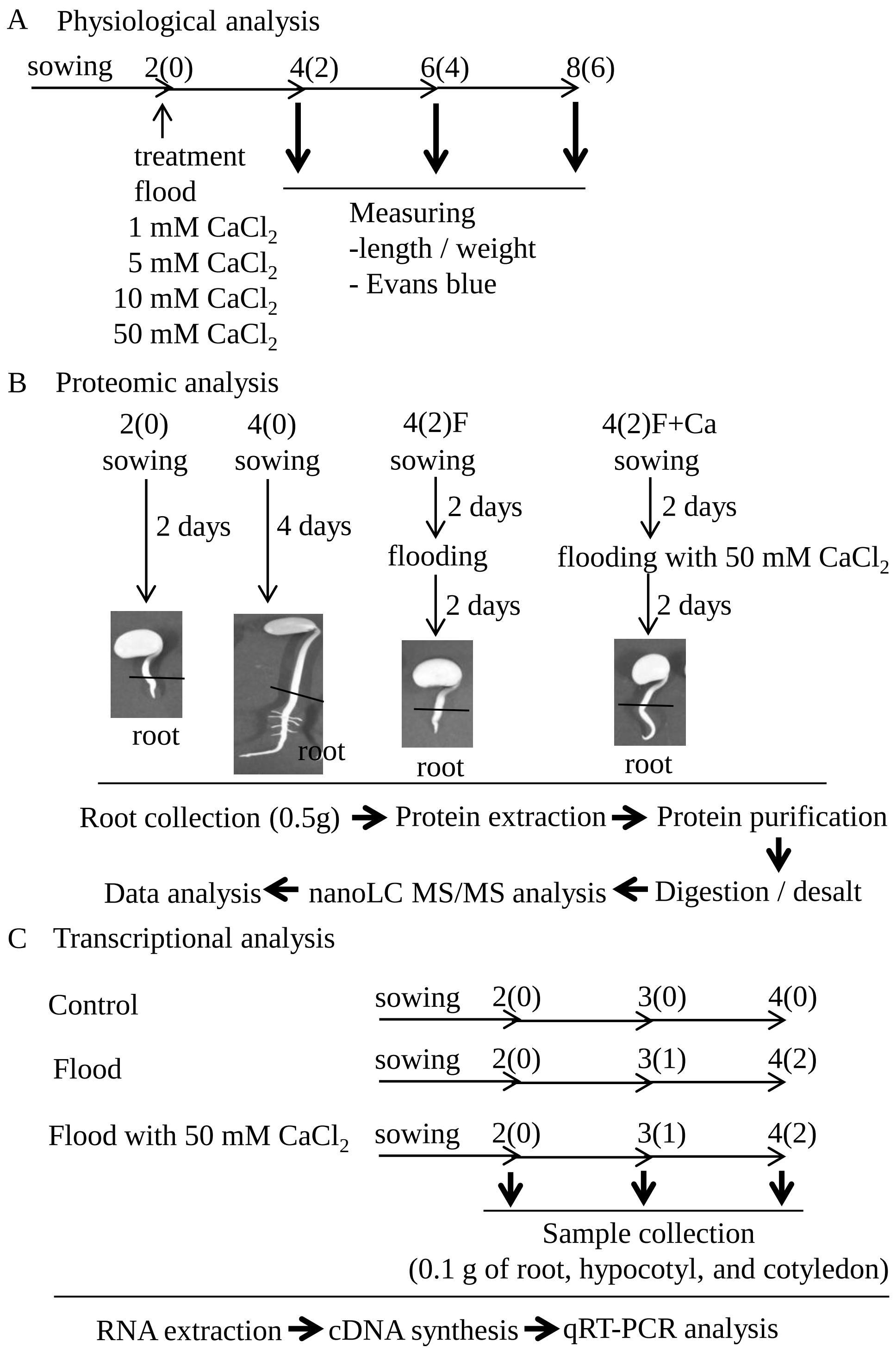


Supplemental Fig. 1

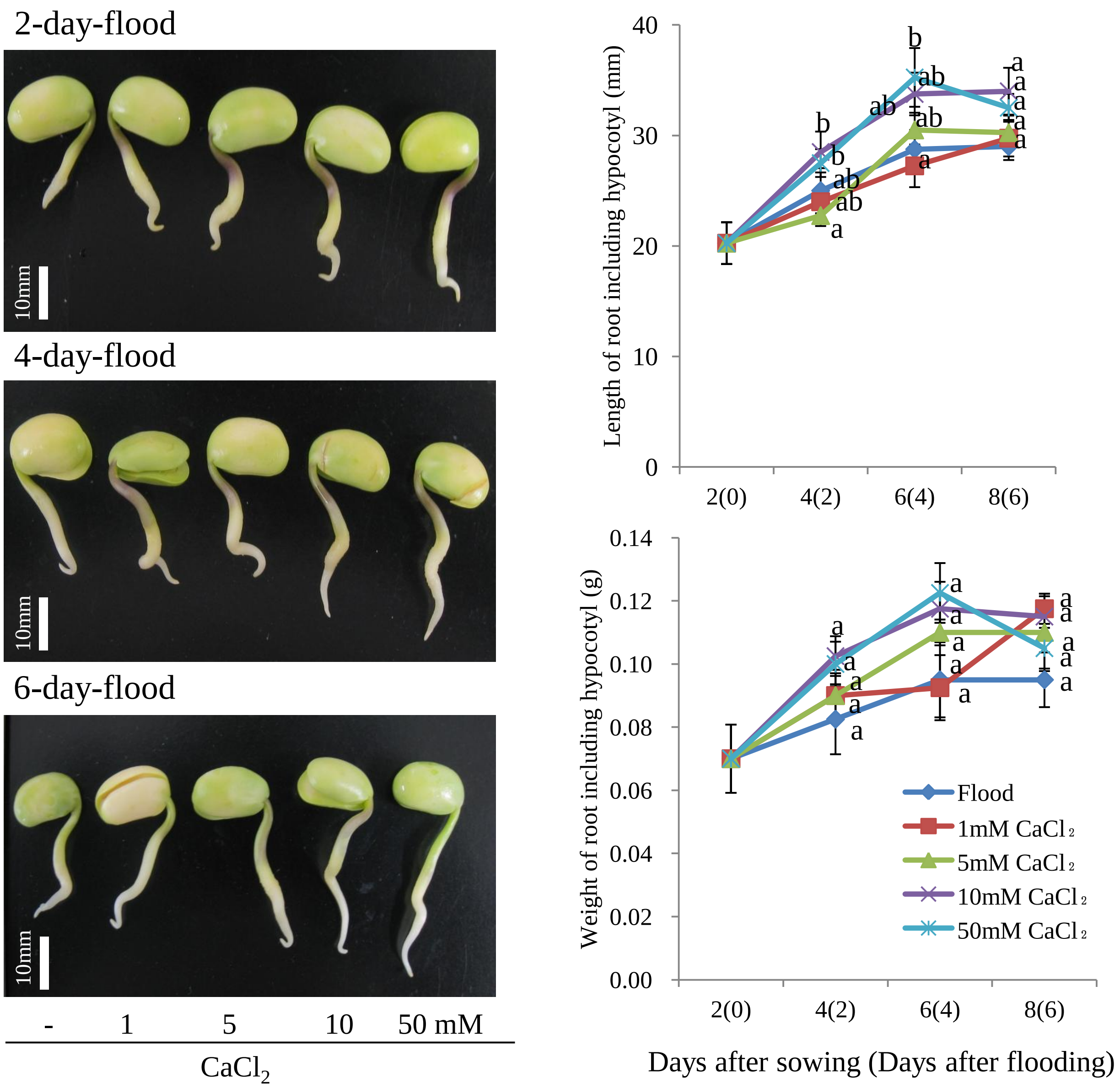


Supplemental Fig. 2A

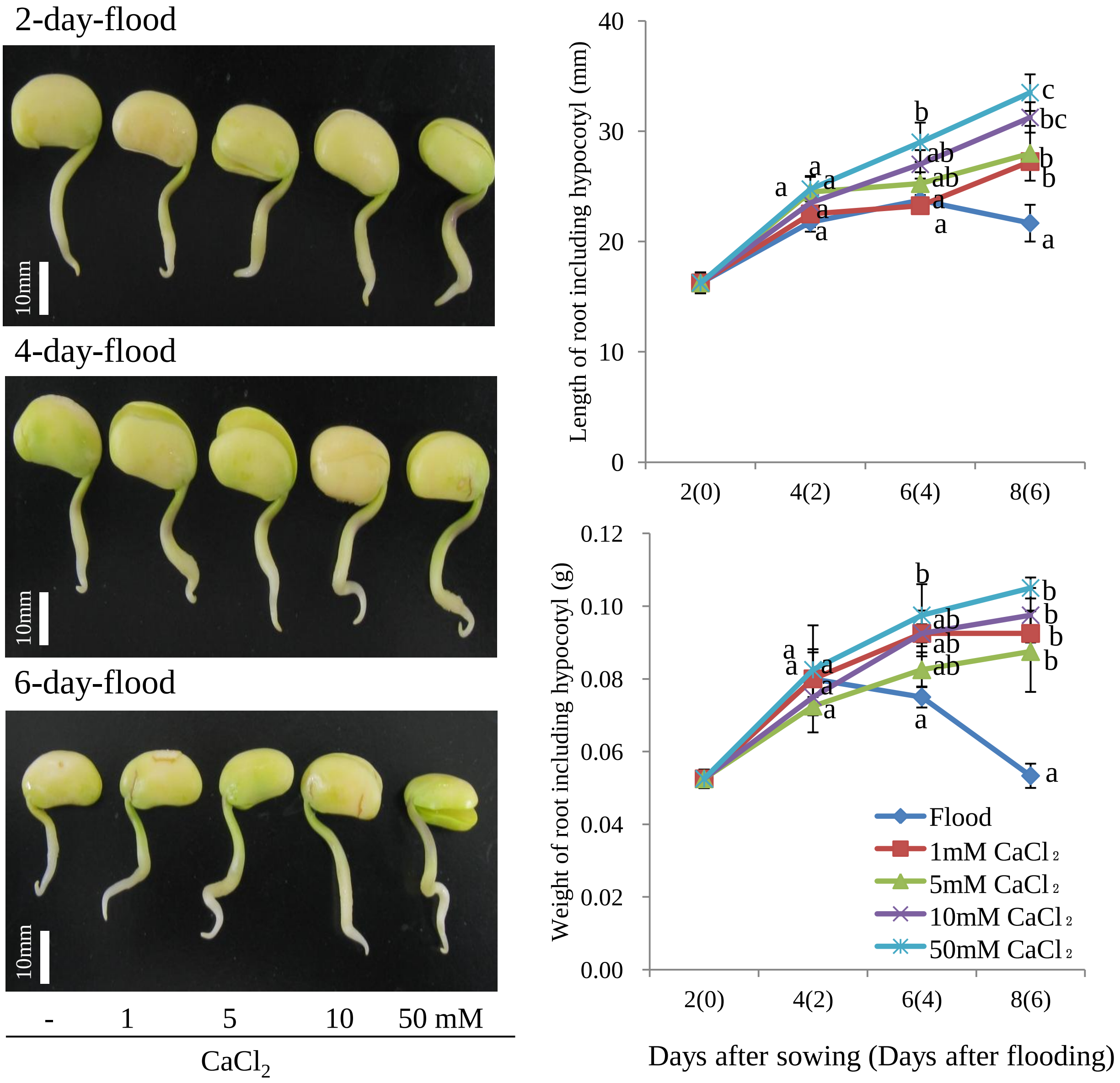


Supplemental Fig. 2B

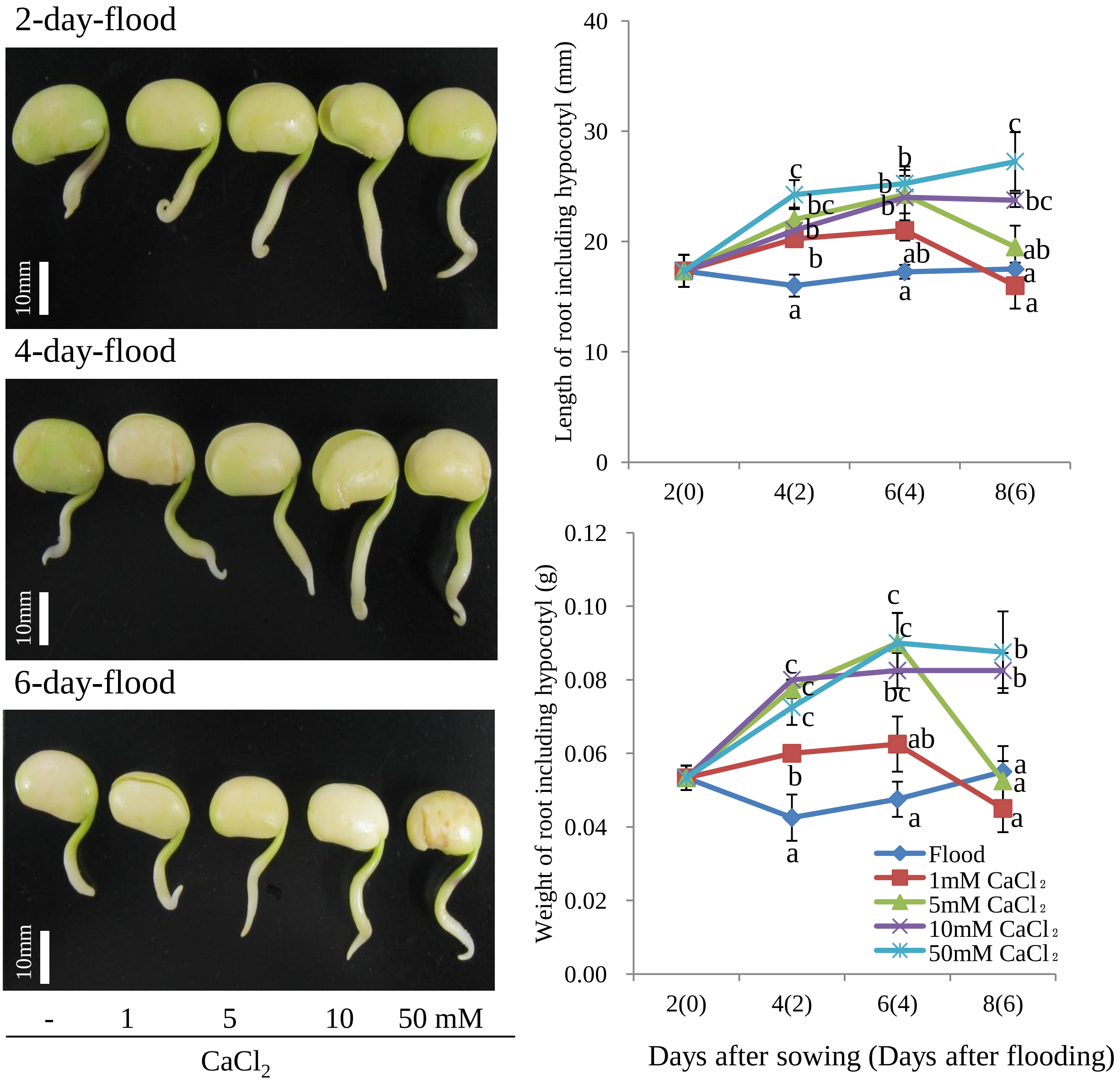


Supplemental Fig. 2C

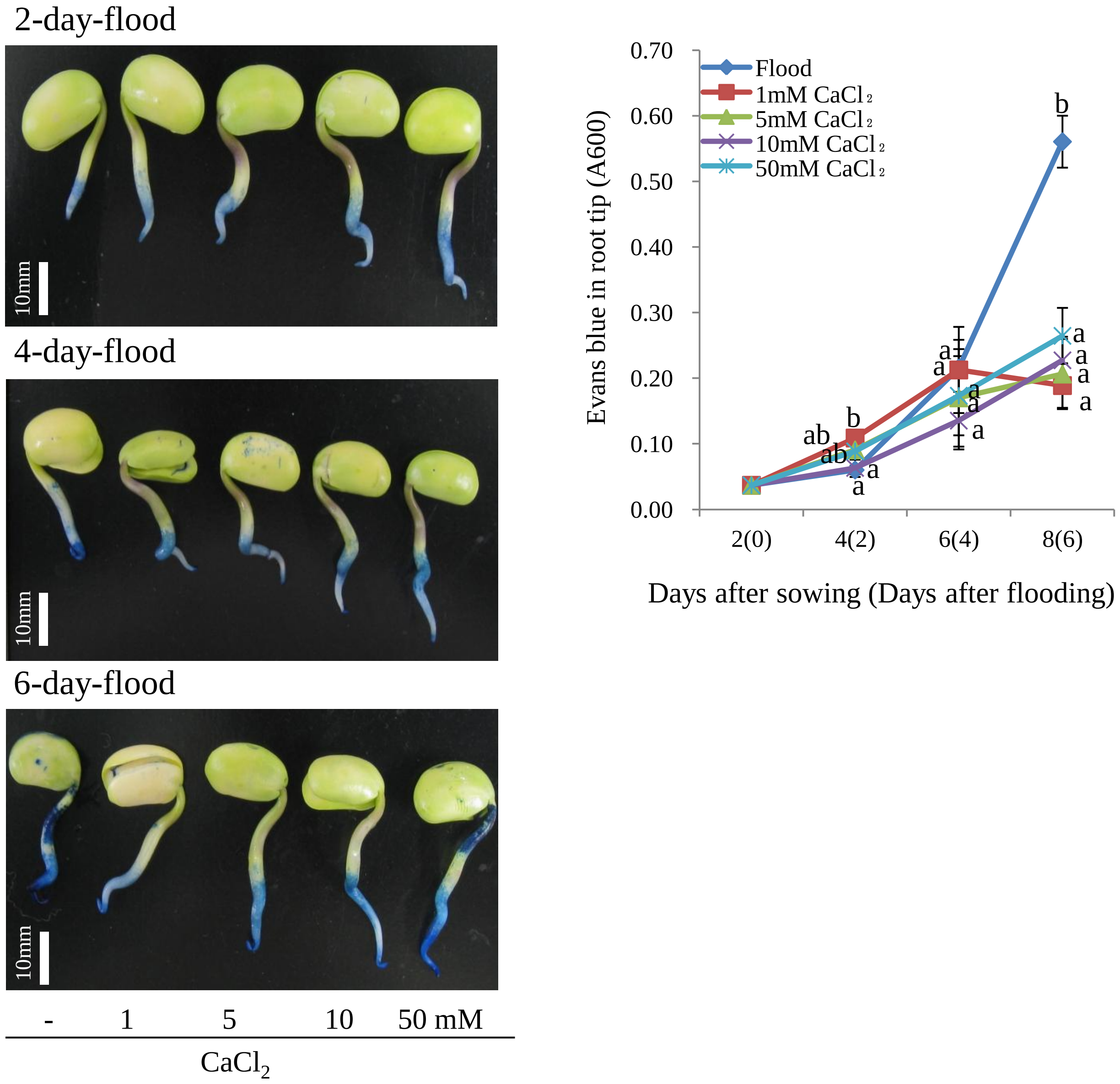


Supplemental Fig. 3A

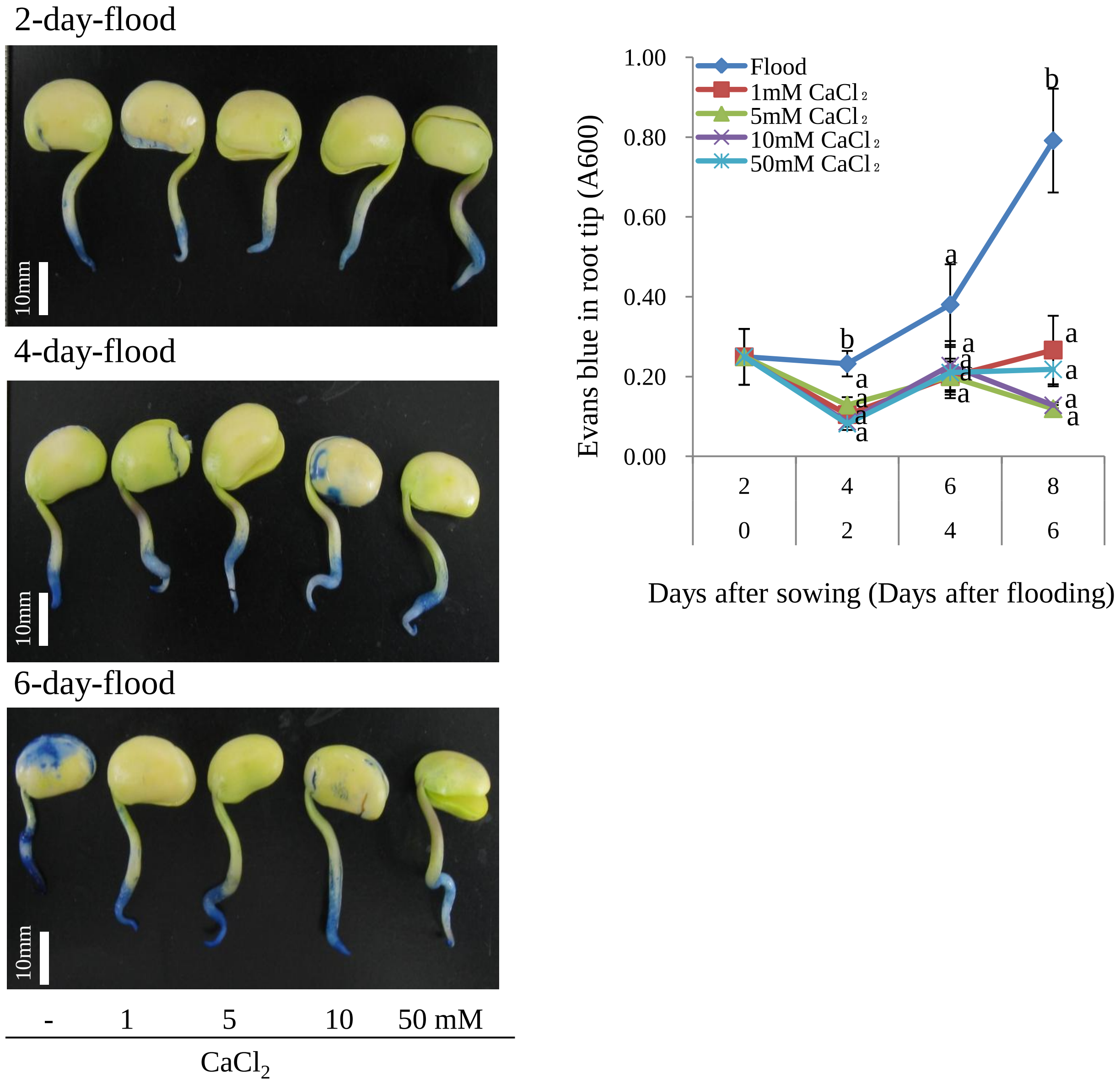


Supplemental Fig. 3B

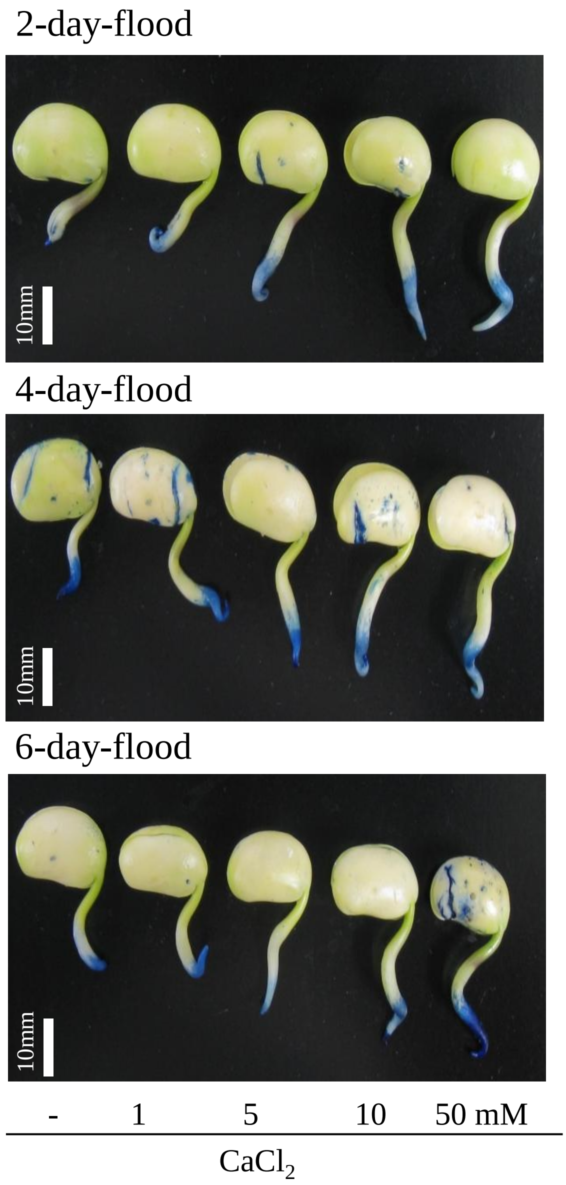


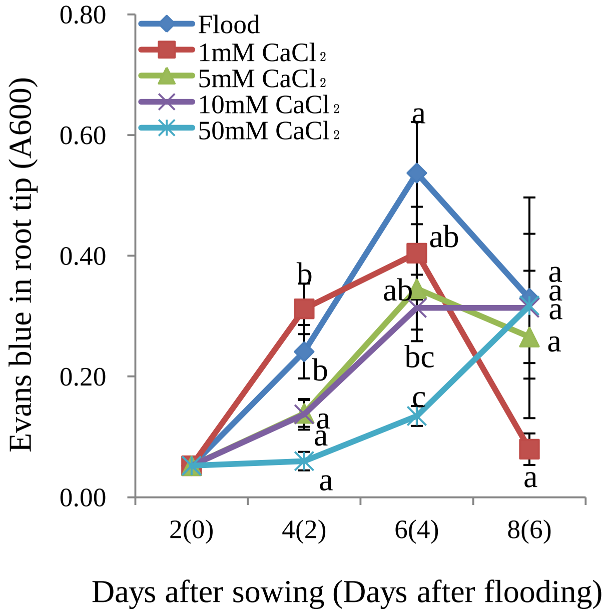


Supplemental Fig. 3C

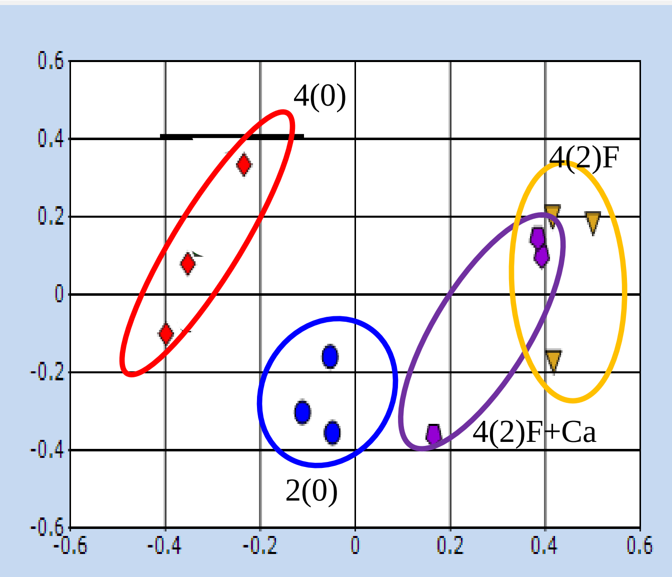


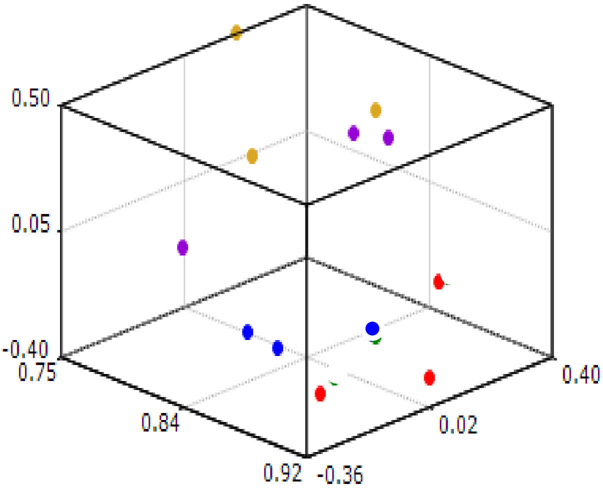


Supplemental Fig. 4

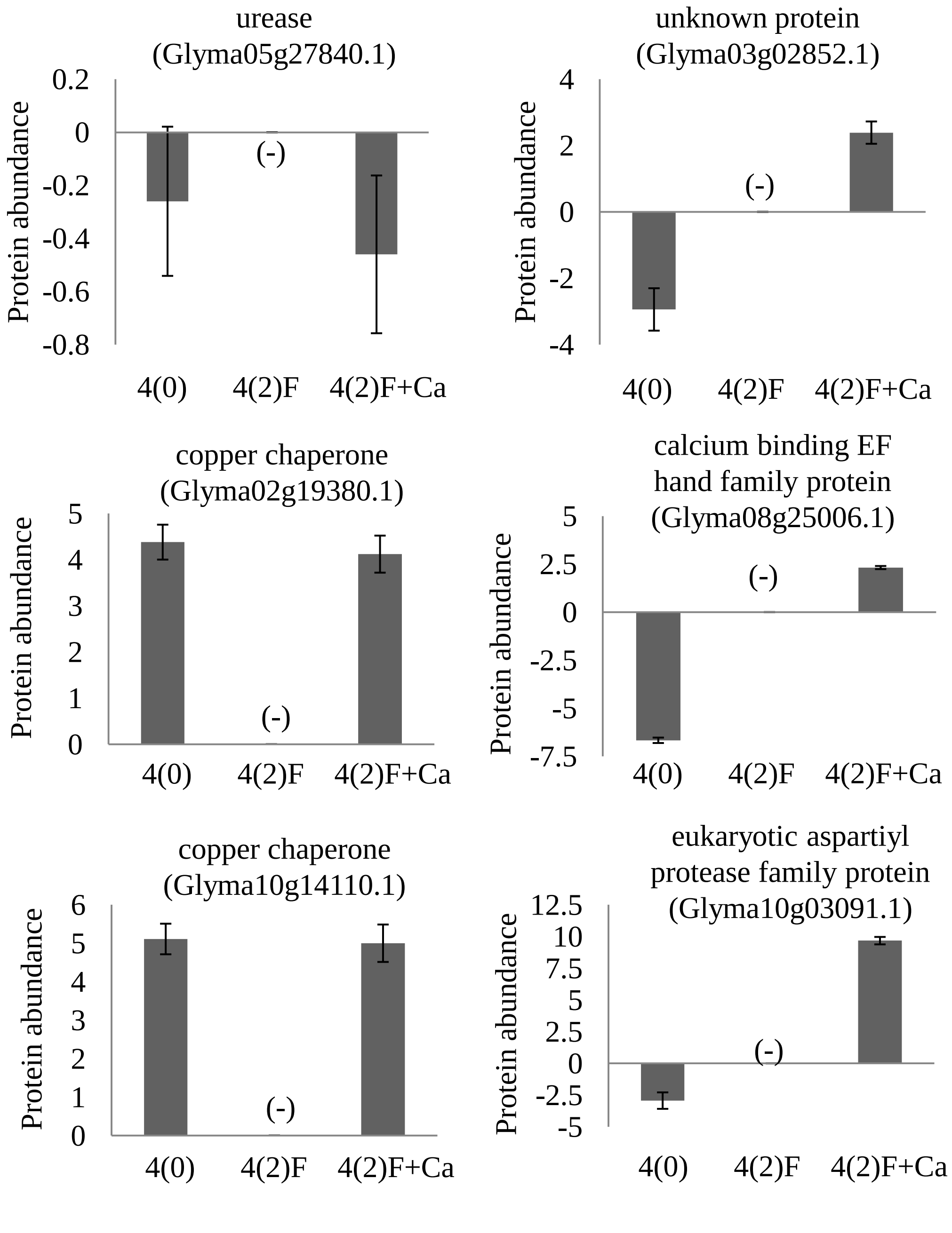


Supplemental Fig. 5
