# Supplementary material for: Gel-free proteomic analysis of soybean root proteins affected by calcium under flooding stress
Source: Front Plant Sci. 2014 Oct 20;5:559. doi: 10.3389/fpls.2014.00559 (PMC4202786; doi:10.3389/fpls.2014.00559)
Supplement: Supplementary file 2 [file DataSheet1.PDF]

Supplemental Table 1. List of qRT-PCR primers used in this study.

Gene name	Protein ID	Sequence	
urease	Glyma05g27840.1	Forward	5'-CGG CAT CAC AAC ATT AGT GG-3'
		Reverse	5'-TAC TTC CCC AGT CCT CAT GC-3'
copper chaperone	Glyma10g14110.1	Forward	5'-GAA AGA AGA CGG CAT TCT GG -3'
		Reverse	5'-TAA GCA ACA GTT TCA ACA GCA G-3'
18S rRNA	X02623.1*	Forward	5'-TGA TTA ACA GGG ACA GTC GG-3'
		Reverse	5'-ACG GTA TCT GAT CGT CTT CG-3'

Protein ID, according to the Phytozome database; *Genbank accession number

Supplemental Table 2. List of identified root proteins that were differentially changed in 4-day-old soybeans compared to 2-day-old untreated soybeans.

Protein ID	Description	M.P.	Ratio 4(0)/2(0)	Pvalue 4(0)/2(0)	Function
Glyma10g29630.1	NAD(P) binding Rossmann fold superfamily protein	8	0.06	0.0000	misc
	Peptide Sequnce	Ratio 4(0)	Pvalue 4(0)		
	DPMAVPADLGYDENCK+Carbamidomethyl(15)	0.03	0.0094		
	DPMAVPADLGYDENCKR+Carbamidomethyl(15)	0.02	0.0001		
	EEETAQFGAQVPMK	0.10	0.0522		
	EGSSIINTTSVNAYK	0.06	0.0003		
	GLALQLVSK	0.05	0.0181		
	IALVTGGDSGIGR	0.07	0.0045		
	LLDYTSTK	0.05	0.0577		
	TSDAKDPMAVPADLGYDENCK+Carbamidomethyl(20)	0.00	1.0000		
Glyma09g29360.2	kunitz family trypsin and protease inhibitor protein	2	0.10	0.0117	stress
	Peptide Sequnce	Ratio 4(0)	Pvalue 4(0)		
	DYVLSFCPSFAQTLCR+Carbamidomethyl(7)Carbamidomethyl(15)	0.08	0.0298		
	YYILPVFR	0.12	0.0528		
Glyma20g37670.1	NAD(P) binding Rossmann fold superfamily protein	6	0.11	0.0000	misc
	Peptide Sequnce	Ratio 4(0)	Pvalue 4(0)		
	EEETAQFGAQVPMK	0.10	0.0522		
	EGSSIINTTSVNAYK	0.06	0.0003		
	EHAMTPVPQFTSPDYKPSNK	0.79	0.0054		
	GLALQLVSK	0.05	0.0181		
	IALVTGGDSGIGR	0.07	0.0045		
	LLDYTSTK	0.05	0.0577		
Glyma06g35680.1	ARM repeat superfamily protein	2	0.12	0.0265	protein
	Peptide Sequnce	Ratio 4(0)	Pvalue 4(0)		
	FQEAVGMGPYLVK	0.38	0.2173		
	VSANPAFAGIAAK	0.07	0.0188		
Glyma01g30670.1	PEBP (phosphatidylethanolamine binding protein) family protein	5	0.14	0.0000	not assigned
	Peptide Sequnce	Ratio 4(0)	Pvalue 4(0)		
	AAEMGGDYATIK	0.04	0.0584		
	AAEMGGDYATIK+Oxidation(4)	0.68	0.7319		
	LVSPAFSNEGK	0.16	0.0304		
	LYALDDELK	0.10	0.0000		
	NISPPLEWYNLPEGTK	0.17	0.0000		
Glyma13g23790.1	formate dehydrogenase	6.00	0.15	0.0160	C1-metabolism

	Peptide Sequnce	Ratio 4(0)	Pvalue 4(0)		
	CDVIVINTPLTEQTR+Carbamidomethyl(1)	0.01	0.0230		
	EGQLASQYR	0.00	1.0000		
	FEEDLDAMLPK	0.29	0.1657		
	GEDFPEQNYIVK	0.25	0.0725		
	HIPDAHVIISTPFHPAYVTAER	0.02	0.1129		
	IVGVFYK	0.33	0.1342		
Glyma08g25006.1	calcium-binding EF-hand family protein	6	0.15	0.0000	signalling
	Peptide Sequnce	Ratio 4(0)	Pvalue 4(0)		
	DFVNDETAFTK	0.14	0.0000		
	GVVVIDGSTVR	0.32	0.0116		
	LIETHFGIDVSTPPEQLAK	0.07	0.0058		
	LYDSIFDK	0.17	0.0001		
	LYDSIFDKFDGDR	1.34	0.8273		
	MVLEDDPNSLLQK	0.01	0.0359		
Glyma03g06780.1	PEBP (phosphatidylethanolamine binding protein) family protein	3	0.17	0.0000	not assigned
	Peptide Sequnce	Ratio 4(0)	Pvalue 4(0)		
	LYALDDELK	0.10	0.0000		
	NISPPLEWYNLPEGTK	0.17	0.0000		
	SAEMGGDYAAIK	0.42	0.1201		
Glyma12g06970.1	dessication-induced 1VOC superfamily protein	4	0.19	0.0002	biodegradation of Xenobiotics
	Peptide Sequnce	Ratio 4(0)	Pvalue 4(0)		
	DVAESVAFYAK	0.19	0.0025		
	ERPPMEVCFVYSDVDAAYK+Carbamidomethyl(8)	0.26	0.0833		
	HAVENGAVAVSEPELK	0.06	0.0374		
	WGELETGNTTIAFTPIHQHETDDLTGAVHNPGSCR+Carbamidomethyl(34)	0.06	0.0396		
Glyma15g30610.1	calcium-binding EF-hand family protein	5	0.21	0.0000	signalling
	Peptide Sequnce	Ratio 4(0)	Pvalue 4(0)		
	GVVVIDGSTVR	0.32	0.0116		
	LYDSIFDK	0.17	0.0001		
	LYDSIFDKFDGDR	1.34	0.8273		
	MVLEDDPNSLLQK	0.01	0.0359		
	SVDEQFGVLDLNNDGVLSR	0.20	0.0712		
Glyma13g44020.1	Late embryogenesis abundant protein (LEA) family protein	6	0.22	0.0326	development
	Peptide Sequnce	Ratio 4(0)	Pvalue 4(0)		
	DNTQGFLQQTGEK	0.15	0.0051		

	ESAQSGKDNTQGFLQQTGEK	0.17	0.1567		
	GAAQGATEAVK	0.87	0.9292		
	QTLGLGEHDQDNR	0.38	0.1433		
	QTLGLGEHDQDNRR	0.23	0.9754		
	TNQTMGNIGEK	1.29	0.1280		
Glyma11g19070.1	NAD ADP ribosyltransferases;NAD ADP ribosyltransferases	9	0.23	0.0204	protein
	Peptide Sequnce	Ratio 4(0)	Pvalue 4(0)		
	CQDLLFYGALDK+Carbamidomethyl(1)	0.00	1.0000		
	EAWLIDSIEK	0.06	0.1129		
	LEPLVANFMK	0.12	0.1220		
	LFDEITGNEFEPWER	0.01	0.1122		
	LGCSISALDK+Carbamidomethyl(3)	0.60	0.2436		
	LPDSVQDSLASDLLK	0.18	0.0616		
	SETGVAEEYDEFCK+Carbamidomethyl(13)	0.05	0.0992		
	SSDDYEMIVK	0.28	0.1739		
	VLLWCGSR+Carbamidomethyl(5)	0.41	0.2461		
Glyma11g16090.1	responsive to abscisic acid 28	2	0.23	0.0005	development
	Peptide Sequnce	Ratio 4(0)	Pvalue 4(0)		
	EDAEAVYTAEVQFPWQR	0.05	0.2775		
	LSDILTDAAEK	0.25	0.0002		
Glyma13g31120.1	Late embryogenesis abundant protein (LEA) family protein	3	0.23	0.0187	not assigned
	Peptide Sequnce	Ratio 4(0)	Pvalue 4(0)		
	QGVQGAWETAK	0.43	0.1626		
	TEEVAASAGEALK	0.17	0.0505		
	TNWTYDSAEAR	0.04	0.0607		
Glyma17g34110.1	glutathione peroxidase 7	2	0.24	0.0003	redox
	Peptide Sequnce	Ratio 4(0)	Pvalue 4(0)		
	AEFPIFDK	0.12	0.0007		
	SSAGGFLGDLIK	0.27	0.0355		
Glyma18g44820.1	alpha galactosidase 1	5	0.24	0.0002	minor CHO metabolism
	Peptide Sequnce	Ratio 4(0)	Pvalue 4(0)		
	APLLLGCDVR+Carbamidomethyl(7)	0.31	0.1003		
	ETADALVSTGLSK	0.26	0.0287		
	EVISVNQDPLGVQGK	0.24	0.0137		
	LGYTYVNIDDCWAELNR+Carbamidomethyl(11)	0.14	0.0336		
	TFASWGIDYLK	0.21	0.0306		

Glyma07g15341.1	caffeoyl CoA 3 O-methyltransferase	2	0.24	0.0037	secondary metabolism
	Peptide Sequnce	Ratio 4(0)	Pvalue 4(0)		
	IGGLLVYDNTLWGGR	0.24	0.0262		
	VEFALTSVGDGLNICR+Carbamidomethyl(15)	0.25	0.0162		
Glyma08g45531.1	kunitz trypsin inhibitor 1	5	0.25	0.0000	stress
	Peptide Sequnce	Ratio 4(0)	Pvalue 4(0)		
	CGDIGISIDHDDGTR+Carbamidomethyl(1)	0.05	0.0000		
	CGDIGISIDHDDGTRR+Carbamidomethyl(1)	0.02	0.0000		
	CPLTVVQSR+Carbamidomethyl(1)	0.50	0.0034		
	NKPLVVQFQK	0.02	0.0025		
	VSDDEFNNYK	0.01	0.0103		
Glyma13g31410.1	Aluminium induced protein with YGL and LRDR motifs	2	0.25	0.0279	hormone metabolism
	Peptide Sequnce	Ratio 4(0)	Pvalue 4(0)		
	DGSLVCSDDPTIIR+Carbamidomethyl(6)	0.11	0.1132		
	FAFIIFDAK	0.31	0.0383		
Glyma15g07930.1	Aluminium induced protein with YGL and LRDR motifs	2	0.25	0.0279	hormone metabolism
	Peptide Sequnce	Ratio 4(0)	Pvalue 4(0)		
	DGSLVCSDDPTIIR+Carbamidomethyl(6)	0.11	0.1132		
	FAFIIFDAK	0.31	0.0383		
Glyma14g03470.1	glutathione S transferase PHI 9	3	0.25	0.0359	misc
	Peptide Sequnce	Ratio 4(0)	Pvalue 4(0)		
	EVEFEVVPVDVTK	0.19	0.0515		
	LQPFGVVPVIK	0.45	0.1612		
	LVQVLNIYEER	0.25	0.1418		
Glyma15g40190.1	glutathione S transferase TAU 19	2	0.25	0.0351	misc
	Peptide Sequnce	Ratio 4(0)	Pvalue 4(0)		
	NPLLPSDPYQR	0.21	0.0483		
	VYEFIMDIR	0.29	0.1179		
Glyma11g15870.1	RmlC like cupins superfamily protein	4	0.25	0.0388	development
	Peptide Sequnce	Ratio 4(0)	Pvalue 4(0)		
	DGPLEFFGFSTSAR	0.40	0.6195		
	LGFIYDDELAER	0.07	0.1030		
	QGDVFVVPR	0.01	0.0081		
	TVVEEIFSK	0.84	0.5724		
Glyma13g29400.1	Protein of unknown function (DUF1000)	2	0.26	0.0377	not assigned
	Peptide Sequnce	Ratio 4(0)	Pvalue 4(0)		

	SISIVGGADGTSPSK	0.23	0.0078		
	VTALNEANPGSVK	0.81	0.7909		
Glyma09g40990.1	alpha galactosidase 1	6	0.26	0.0014	minor CHO metabolism
	Peptide Sequnce	Ratio 4(0)	Pvalue 4(0)		
	APLLLGCDVR+Carbamidomethyl(7)	0.31	0.1003		
	ETADALVSTGLSK	0.26	0.0287		
	EVIAVNQDPLGVQGK	0.36	0.0376		
	LGYTYVNIDDCWAELNR+Carbamidomethyl(11)	0.14	0.0336		
	TFASWGIDYLK	0.21	0.0306		
	TTNDINDSWESMISR	0.00	1.0000		
Glyma20g34960.1	Plastid lipid associated protein PAP	2	0.26	0.0023	cell
	Peptide Sequnce	Ratio 4(0)	Pvalue 4(0)		
	LLPITLGQVFQR	0.46	0.2386		
	LNLLSAVSGLNR	0.05	0.0011		
Glyma14g06900.1	HSP20 like chaperones superfamily protein	2	0.26	0.0034	stress
	Peptide Sequnce	Ratio 4(0)	Pvalue 4(0)		
	AAMENGVLTVTVPK	0.26	0.0251		
	ETPAAHVFNVDLPGLK	0.27	0.0151		
Glyma05g27840.1	urease	8	0.26	0.0000	amino acid metabolism
	Peptide Sequnce	Ratio 4(0)	Pvalue 4(0)		
	DFALYGDECVFGGGK+Carbamidomethyl(9)	0.0061797	0.018536459		
	DGLIVSIGK	0.2150861	0.035114979		
	GPLQPGESDNDNFR	0.1160786	0.006523961		
	GSSSKPDELHDIIK	0.1408257	0.002407979		
	LGLHNAGYLAQK	0.1667243	0.001908171		
	LMLQSTDDLPLNFGFTGK	0.6947982	0.457614167		
	LNIAAGTAVR	0.396115	0.086942111		
	TIHTYHSEGAGGGHAPDIIK	0.066399	0.000150068		
Glyma16g34640.2	alpha galactosidase 1	2	0.27	0.0197	minor CHO metabolism
	Peptide Sequnce	Ratio 4(0)	Pvalue 4(0)		
	APLIIGCDVR+Carbamidomethyl(7)	0.31	0.1003		
	ETADALVSTGLSK	0.26	0.0287		
Glyma06g17860.1	purple acid phosphatase 27	5	0.28	0.0000	misc
	Peptide Sequnce	Ratio 4(0)	Pvalue 4(0)		
	ASPYPGQNSLQR	0.37	0.1014		

	DPGFIHTSFLK	0.36	0.0524		
	FCIADSEHDWR+Carbamidomethyl(2)	0.25	0.0001		
	VCPIYQNQCVNEEK+Carbamidomethyl(2)Carbamidomethyl(9)	0.24	0.0133		
	VIIFGDMGK	0.44	0.1727		
Glyma15g07910.4	allantoinase	3	0.29	0.0026	nucleotide metabolism
	Peptide Sequnce	Ratio 4(0)	Pvalue 4(0)		
	IVTPQGIISGSVEINDGK	0.31	0.0757		
	LMEEGDFLK	0.65	0.4365		
	TEWEGFDTGTR	0.20	0.0013		
Glyma10g07090.1	UDP glucosyl transferase 73C2	4	0.29	0.0002	misc
	Peptide Sequnce	Ratio 4(0)	Pvalue 4(0)		
	AINELMDESR	0.55	0.1233		
	FTSTFSNSQIR	0.25	0.0182		
	LVVQILR	0.22	0.0019		
	VGVEVPVEWGEEDENGLLVK	0.39	0.0762		
Glyma01g42840.1	glutathione peroxidase 6	6	0.29	0.0000	redox
	Peptide Sequnce	Ratio 4(0)	Pvalue 4(0)		
	AEFPIFDK	0.12	0.0007		
	FLVDKDGNVVDR	0.24	0.0936		
	GGLFGDSIK	0.07	0.0058		
	GGLFGDSIKWNFSK	1.84	0.3386		
	VDVNGDNAAPLYK	0.15	0.0552		
	YAPTTSPLSIEK	0.42	0.0118		
Glyma11g02630.1	glutathione peroxidase 6	6	0.2900	0.0000	redox
	Peptide Sequnce	Ratio 4(0)	Pvalue 4(0)		
	AEFPIFDK	0.12	0.0007		
	FLVDKDGNVVDR	0.24	0.0936		
	GGLFGDSIK	0.07	0.0058		
	GGLFGDSIKWNFSK	1.84	0.3386		
	VDVNGDNAAPLYK	0.15	0.0552		
	YAPTTSPLSIEK	0.42	0.0118		
Glyma16g05240.1	Glycosyl hydrolase family 38 protein	4	0.2900	0.0097	misc
	Peptide Sequnce	Ratio 4(0)	Pvalue 4(0)		
	ELNVELAPMEIR	0.64	0.4371		
	IPVIEANVR	0.50	0.1147		
	TDDFFPYADR	0.21	0.0086		

	VNALYSTPSIYTDAK	0.15	0.0971		
Glyma05g38110.1	osmotin 34	4	0.3000	0.0083	stress
	Peptide Sequnce	Ratio 4(0)	Pvalue 4(0)		
	CSYPVWAAAVPGGGR+Carbamidomethyl(1)	0.31	0.0407		
	CTADINGQCPSQLK+Carbamidomethyl(1)Carbamidomethyl(9)	0.30	0.0020		
	LNSGQSWALDVPAGTK	0.57	0.4177		
	TDQYCCNSGSCVPTDYSR+Carbamidomethyl(5)Carbamidomethyl(6)Carbamidomethyl(11)	0.00	1.0000		
Glyma04g37790.1	Glycosyl hydrolase family 38 protein	4	0.3000	0.0048	misc
	Peptide Sequnce	Ratio 4(0)	Pvalue 4(0)		
	FIYVEQAFFQR	0.45	0.0787		
	ITEVSLSANQER	0.92	0.8087		
	TDDFFPYADR	0.21	0.0086		
	TFIISFR	0.31	0.0296		
Glyma06g17270.1	Glycosyl hydrolase family 38 protein	4	0.3000	0.0048	misc
	Peptide Sequnce	Ratio 4(0)	Pvalue 4(0)		
	FIYVEQAFFQR	0.45	0.0787		
	ITEVSLSANQER	0.92	0.8087		
	TDDFFPYADR	0.21	0.0086		
	TFIISFR	0.31	0.0296		
Glyma13g31430.10	allantoinase	4	0.3100	0.0015	nucleotide metabolism
	Peptide Sequnce	Ratio 4(0)	Pvalue 4(0)		
	AAAAGGVTTVVDMPLNCYPTTVSK+Carbamidomethyl(17)	0.41	0.0868		
	IVTPQGIISGSVEINDGK	0.31	0.0757		
	LMEEGDFLK	0.65	0.4365		
	TEWEGFDTGTR	0.20	0.0013		
Glyma03g07470.1	Stress induced protein	4	0.3100	0.0226	hormone metabolism
	Peptide Sequnce	Ratio 4(0)	Pvalue 4(0)		
	AQEEGIGIDESK	0.09	0.0384		
	EQLGTEGYQEMGR	0.05	0.0562		
	KEQLGTEGYQEMGR	0.01	0.2248		
	SLEAQQHLAEGR	0.51	0.2779		
Glyma03g39860.1	Glycoprotein membrane precursor GPI-anchored	3	0.31	0.0002	not assigned
	Peptide Sequnce	Ratio 4(0)	Pvalue 4(0)		
	ATCLADEVAEEIEK+Carbamidomethyl(3)	0.08	0.0085		
	MPCENVNQYYPSSVPGSGNLK+Carbamidomethyl(3)	0.04	0.0357		
	SLVPLSQVSK	0.32	0.0048		

Glyma13g42310.1	lipoxygenase 1	17	0.31	0.0019	hormone metabolism
	Peptide Sequnce	Ratio 4(0)	Pvalue 4(0)		
	DQNSEKPGEVYVPR	0.01	0.0565		
	EGVESTIWLLAK	0.22	0.0181		
	FPPPHVIQVSK	0.12	0.0922		
	FVCNSWVYNTK+Carbamidomethyl(3)	0.05	0.0474		
	GIPNSISI	0.33	0.1162		
	HSVEMSSAVYK	5.14	0.3385		
	IFFANHTYVPSETPAALVGYR	0.00	1.0000		
	ITADALDLDGYTVDEALASR	0.00	1.0000		
	LGPVQLPYTLLHPNSEEGLTCR+Carbamidomethyl(21)	0.05	0.0693		
	NVLDFNSVADLTK	0.09	0.0582		
	NWVFTDQALPADLIK	5.08	0.2102		
	NYMQVEFYLK	0.03	0.1398		
	QSLINADGIIEK	0.60	0.2276		
	SAWMTDEEFAR	0.33	0.3600		
	SLTLEDVPNQGTIR	0.00	1.0000		
	SNLDPTIYGEQTSK	0.11	0.0426		
	SSDFLAYGIK	0.49	0.3066		
Glyma07g11310.1	B-S glucosidase 44	5	0.31	0.0019	misc
	Peptide Sequnce	Ratio 4(0)	Pvalue 4(0)		
	FGIVYVDFK	0.20	0.0332		
	FSISWSR	0.43	0.1002		
	GFLFGTATSAYQVEGMAHK	0.31	0.0158		
	NWMTFNEPR	0.31	0.0946		
	VVAALGYDNGFFAPGR	0.32	0.1765		
Glyma13g06040.1	Glycosyl transferase family 35	14	0.32	0.0000	major CHO metabolism
	Peptide Sequnce	Ratio 4(0)	Pvalue 4(0)		
	AASEEFDLSAFNAGR	0.29	0.0315		
	AVAHDVPIPGYK	0.49	0.0298		
	DAWNITQR	0.72	0.5170		
	DFPSYIECQEK+Carbamidomethyl(8)	0.25	0.0039		
	EEVGADNFFLFGAK	0.28	0.0147		
	FITDVGATVNHDPEIGDLLK	0.22	0.0178		
	ICYILYPGDESIEGK+Carbamidomethyl(2)	0.36	0.0078		
	MSILNTAGSYK	0.05	0.0895		
	QAYYLSMEFLQGR	0.45	0.0772		

	SGTNVNWEEFPEK	0.36	0.1137		
	TGYSVSPDAMFDIQVK	0.15	0.0652		
	TIIAEYGTENSDLLEK	0.23	0.0388		
	VAVQMNDTHPTLCIPELMR+Carbamidomethyl(13)	0.30	0.0950		
	WSLDLMQK	0.26	0.0867		
Glyma08g09310.1	1-cysteine peroxiredoxin 1	12	0.33	0.0000	redox
	Peptide Sequnce	Ratio 4(0)	Pvalue 4(0)		
	ALHIVGPDLK	0.06	0.1391		
	DIEAYTPGAK	0.47	0.2798		
	EMFPQGFESVK	0.30	0.0053		
	IKLSFLYPATTGR	1.13	0.9161		
	LLGLSCDDVQSHNEWIK+Carbamidomethyl(6)	0.81	0.2112		
	LSFLYPATTGR	0.09	0.0138		
	PGLTIGDTIPDLQVETNQGK	0.19	0.0097		
	QLNMVDPDEK	0.04	0.0183		
	QLNMVDPDEKDSTGNLPSR	0.26	0.1207		
	VATPANWKPGDPVVITPDVTNEQAK	0.09	0.0606		
	VNYPIIADPK	0.08	0.0814		
	VNYPIIADPKR	0.33	0.5014		
Glyma19g03490.1	Glycosyl transferase family 35	13	0.33	0.0000	major CHO metabolism
	Peptide Sequnce	Ratio 4(0)	Pvalue 4(0)		
	AASEEFDLSAFNAGR	0.29	0.0315		
	AVAHDVPIPGYK	0.49	0.0298		
	DAWNITQR	0.72	0.5170		
	DFPSYIECQEK+Carbamidomethyl(8)	0.25	0.0039		
	EEVGADNFFLFGAK	0.28	0.0147		
	FITDVGATVNHDPEIGDLLK	0.22	0.0178		
	MSILNTAGSYK	0.05	0.0895		
	QAYYLSMEFLQGR	0.45	0.0772		
	QLMNIFGIVYR	1.03	0.9672		
	TGYSVSPDAMFDIQVK	0.15	0.0652		
	TIIAEYGTENSDLLEK	0.23	0.0388		
	VAVQMNDTHPTLCIPELMR+Carbamidomethyl(13)	0.30	0.0950		
	WSLDLMQK	0.26	0.0867		
Glyma19g36080.1	Glutathione S transferase family protein	8	0.33	0.0000	misc
	Peptide Sequnce	Ratio 4(0)	Pvalue 4(0)		
	EFGEELISHVDTFTK	0.56	0.0885		

	LATWIEEVNK	0.43	0.0349		
	LVPIDLQNRPAWYK	0.41	0.0546		
	LYISYICPYAQR+Carbamidomethyl(7)	1.82	0.2735		
	VLGESLDLVK	0.43	0.0113		
	VPPLTSTSEPPSLFDGTTR	0.24	0.0168		
	YIDDNFEGPSLVPSDPAK	0.27	0.0004		
	YIDDNFEGPSLVPSDPAKK	0.51	0.4491		
Glyma13g22890.1	Raffinose synthase family protein	3	0.33	0.0233	minor CHO metabolism
	Peptide Sequnce	Ratio 4(0)	Pvalue 4(0)		
	DCLFTDPAR+Carbamidomethyl(2)	0.31	0.0825		
	EMEEYGSVMK	0.44	0.1985		
	VSSGVTENEPTWK	0.29	0.0406		
Glyma02g47460.1	ADP glucose pyrophosphorylase 1	3	0.34	0.0126	major CHO metabolism
	Peptide Sequnce	Ratio 4(0)	Pvalue 4(0)		
	LIDIPVSNCLNSNVSK+Carbamidomethyl(9)	0.31	0.0153		
	NVMLDLLR	0.33	0.2436		
	VDTTILGLDDER	0.37	0.0803		
Glyma14g01290.1	ADP glucose pyrophosphorylase 1	3	0.34	0.0126	major CHO metabolism
	Peptide Sequnce	Ratio 4(0)	Pvalue 4(0)		
	LIDIPVSNCLNSNVSK+Carbamidomethyl(9)	0.31	0.0153		
	NVMLDLLR	0.33	0.2436		
	VDTTILGLDDER	0.37	0.0803		
Glyma10g03091.1	Eukaryotic aspartyl protease family protein	2	0.34	0.0034	protein
	Peptide Sequnce	Ratio 4(0)	Pvalue 4(0)		
	LGAAVPTIELVLQNQK	0.02	0.0090		
	LGFSSLLYGSR	0.42	0.0430		
Glyma02g03320.1	Matrixin family protein	4	0.34	0.0085	protein
	Peptide Sequnce	Ratio 4(0)	Pvalue 4(0)		
	FSNLPVTGVPNK	0.13	0.1701		
	LFGPALAK	0.54	0.1691		
	MEVYGGSLIFLQPDSSK	0.74	0.6401		
	QLIQQMLSLR	0.29	0.0019		
Glyma12g06950.1	Pathogenesis related thaumatin superfamily protein	6	0.34	0.0001	stress
	Peptide Sequnce	Ratio 4(0)	Pvalue 4(0)		
	GSIYSSFFK	0.34	0.0042		
	GVCPVVGCR+Carbamidomethyl(3)Carbamidomethyl(8)	0.52	0.3334		

	HACPATFTFAHDTPSLMHQCSSPR+Carbamidomethyl(3)Carbamidomethyl(20)	0.29	0.0115		
	SGCEAFHTDELCCR+Carbamidomethyl(3)Carbamidomethyl(12)Carbamidomethyl(13)	0.31	0.0863		
	SLTHQSIPVPDAHWSGR	0.40	0.2295		
	TGCSYSGTAFSCASGDCGGR+Carbamidomethyl(3)Carbamidomethyl(12)Carbamidomethyl(17)	0.35	0.0085		
Glyma03g02852.1	N.D.*	2	0.34	0.0361	not assigned
	Peptide Sequnce	Ratio 4(0)	Pvalue 4(0)		
	ILLGYSAIK	0.42	0.1466		
	VFVSIGGDDPFK	0.28	0.0401		
Glyma19g42530.1	D aminoacid aminotransferase like PLP dependent enzymes superfamily protein	2	0.35	0.0490	misc
	Peptide Sequnce	Ratio 4(0)	Pvalue 4(0)		
	IIGNGEVGPVTR	0.84	0.8607		
	NPLDILPSFDK	0.23	0.0099		
Glyma18g40690.2	NAD(P) linked oxidoreductase superfamily protein	8	0.35	0.0112	minor CHO metabolism
	Peptide Sequnce	Ratio 4(0)	Pvalue 4(0)		
	AENIVPSDIPNTWK	0.09	0.0809		
	AIGVSNFSTK	0.94	0.8809		
	EDLWITSK	0.47	0.1176		
	FFEIQQER	0.39	0.2017		
	LFEEGVVK	0.32	0.0660		
	LGELLEYAR	0.23	0.1077		
	LWCTDHAPEDVPEALDR+Carbamidomethyl(3)	0.73	0.4742		
	VTPAVNQSECHPAWR+Carbamidomethyl(10)	0.15	0.0240		
Glyma03g40000.1	D aminoacid aminotransferase like PLP dependent enzymes superfamily protein	3	0.35	0.0358	misc
	Peptide Sequnce	Ratio 4(0)	Pvalue 4(0)		
	ALAFENVPTQDEIK	0.37	0.1385		
	IIGNGEVGPVTR	0.84	0.8607		
	NPLDILPSFDK	0.23	0.0099		
Glyma07g39960.1	phosphofructokinase 2	8	0.37	0.0407	glycolysis
	Peptide Sequnce	Ratio 4(0)	Pvalue 4(0)		
	GFYSSVPLPLNPK	0.10	0.0275		
	GGLLEFLGQR	0.20	0.0713		
	NIYFDPSIVR	0.24	0.0727		
	QLQQQQGFYLTESDVLLR	1.28	0.2650		
	STGHIALHATLSSR	0.09	0.8329		
	TVDNDVGIIDR	0.13	0.1772		
	VAIVTCGGLCPGLNTVIR+Carbamidomethyl(6)Carbamidomethyl(10)	0.00	1.0000		

	YIDPTYMIR	0.47	0.2378		
Glyma05g31810.1	RAB GTPase homolog G3A	2	0.37	0.0156	signalling
	Peptide Sequnce	Ratio 4(0)	Pvalue 4(0)		
	GNIPYFETSAK	0.78	0.4547		
	IIVLGDSGVGK	0.32	0.0048		
Glyma08g15080.2	RAB GTPase homolog G3A	2	0.37	0.0156	signalling
	Peptide Sequnce	Ratio 4(0)	Pvalue 4(0)		
	GNIPYFETSAK	0.78	0.4547		
	IIVLGDSGVGK	0.32	0.0048		
Glyma13g34290.1	gamma glutamyl hydrolase 1	8	0.38	0.0000	protein
	Peptide Sequnce	Ratio 4(0)	Pvalue 4(0)		
	DNLIYNYKPTFGGTAGK	0.53	0.2097		
	FPSDLLTQLK	0.36	0.0250		
	KYPVTVNLWQPEK	0.50	0.1384		
	LSDFFEILATSEDRDGK	0.30	0.0417		
	NAFEWATSLK	0.38	0.0327		
	VIPLIYNESPENLNK	0.23	0.0466		
	VTQSTANFFISEAR	0.32	0.0030		
	YPVTVNLWQPEK	0.36	0.0366		
Glyma01g00730.5	S-adenosyl L-methionine dependent methyltransferases superfamily protein	6	0.38	0.0025	secondary metabolism
	Peptide Sequnce	Ratio 4(0)	Pvalue 4(0)		
	IDFIESPALPILDK	0.76	0.9309		
	IGGLLVYDNTLWGGR	0.24	0.0262		
	NPVILQSEDLTK	0.54	0.0618		
	TYEVGLPVIK	0.75	0.3523		
	VEFALTSVGDGLNICR+Carbamidomethyl(15)	0.25	0.0162		
	YILETAVYPR	0.01	0.0299		
Glyma12g01970.1	Polyketide cyclase/dehydrase and lipid transport superfamily protein	3	0.38	0.0290	stress
	Peptide Sequnce	Ratio 4(0)	Pvalue 4(0)		
	LTFAPGVPGPAGYK	0.56	0.2154		
	LVAQELPELFQK	0.30	0.0608		
	VELTEGDGGVGTVLK	0.38	0.0677		
Glyma05g09310.2	Pyruvate kinase family protein	12	0.38	0.0001	glycolysis
	Peptide Sequnce	Ratio 4(0)	Pvalue 4(0)		
	ATDAESTEVILEAALK	0.16	0.1010		
	EGQEVTITTDYDIK	0.32	0.0527		

	FNFSHGTHDYHQETLNNLK	0.62	0.5359		
	GDPEMISMSYK	0.14	0.1643		
	GLIPILGEGSAK	0.45	0.0316		
	ICIEAESSLDYGAIFK+Carbamidomethyl(2)	0.36	0.2204		
	IDMIALSFVR	0.65	0.4035		
	IVCTLGPASR+Carbamidomethyl(3)	0.95	0.9195		
	LIVVLTR	0.32	0.0596		
	NVNLPGVVVDLPTLTEK	0.25	0.0021		
	STPLPMSPLESLASSAVR	0.25	0.1459		
	VENQEGVLNFDEILR	0.38	0.0077		
Glyma15g11530.1	glyoxalase I homolog	7	0.38	0.0000	biodegradation of Xenobiotics
	Peptide Sequnce	Ratio 4(0)	Pvalue 4(0)		
	FLHVVYR	0.58	0.1735		
	FYTECFGMK+Carbamidomethyl(5)	0.34	0.1417		
	GNAYAQVAIGTDDVYK	0.38	0.0850		
	ITAFLDPDGWK	0.33	0.0379		
	QPGPIPGLNTK	0.55	0.0900		
	SAEVVNIVTQELGGK	0.37	0.0003		
	TVLVDNQDFLK	0.34	0.0295		
Glyma08g10850.2	urease	4	0.39	0.0003	amino acid metabolism
	Peptide Sequnce	Ratio 4(0)	Pvalue 4(0)		
	DGLIVSIGK	0.22	0.0351		
	LGLHNAGYLAQK	0.17	0.0019		
	LMLQSTDDLPLNFGFTGK	0.69	0.4576		
	TIHTYHWSAGGGHAPDIIK	0.05	0.0123		
Glyma15g07400.1	oxidoreductase zinc binding dehydrogenase family protein	2	0.39	0.0010	misc
	Peptide Sequnce	Ratio 4(0)	Pvalue 4(0)		
	GASPYLGLECSGTVLSLGK+Carbamidomethyl(10)	0.40	0.0087		
	NLDSLNFDGR	0.35	0.0116		
Glyma01g41310.1	glutamate dehydrogenase 2	2	0.39	0.0117	N-metabolism
	Peptide Sequnce	Ratio 4(0)	Pvalue 4(0)		
	LENSLLIPFR	0.41	0.0199		
	TAVADIPYGGAK	0.34	0.0791		
Glyma10g35230.1	Ras related small GTP binding family protein	2	0.39	0.0095	signalling
	Peptide Sequnce	Ratio 4(0)	Pvalue 4(0)		
	EVAVQDGTDYAEK	9.00	0.2566		

	LVLLGDSGVGK	0.32	0.0048		
Glyma20g32320.1	Ras related small GTP binding family protein	2	0.39	0.0095	signalling
	Peptide Sequnce	Ratio 4(0)	Pvalue 4(0)		
	EVAVQDGTDYAEK	9.00	0.2566		
	LVLLGDSGVGK	0.32	0.0048		
Glyma01g15010.1	Stress responsive A/B Barrel Domain	2	0.39	0.0022	not assigned
	Peptide Sequnce	Ratio 4(0)	Pvalue 4(0)		
	DGAVVDELIQGLEK	0.42	0.0222		
	IVVLDFPSNLVK	0.38	0.0105		
Glyma09g23640.1	Stress responsive A/B Barrel Domain	2	0.39	0.0022	not assigned
	Peptide Sequnce	Ratio 4(0)	Pvalue 4(0)		
	DGVAVDELIQGLEK	0.42	0.0222		
	IVVLDFPSNLVK	0.38	0.0105		
Glyma12g09120.1	Stress responsive A/B Barrel Domain	2	0.39	0.0022	not assigned
	Peptide Sequnce	Ratio 4(0)	Pvalue 4(0)		
	DGVAVDELIQGLEK	0.42	0.0222		
	IVVLDFPSNLVK	0.38	0.0105		
Glyma08g20180.1	lipoxygenase 1	2	0.4	0.0030	hormone metabolism
	Peptide Sequnce	Ratio 4(0)	Pvalue 4(0)		
	DTMNINGLAR	0.29	0.1274		
	HLSVLHPIYK	0.41	0.0027		
Glyma08g17450.1	aldehyde dehydrogenase 5F1	5	0.4	0.0022	TCA / org transformation
	Peptide Sequnce	Ratio 4(0)	Pvalue 4(0)		
	EEAFGPVAPLLR	0.55	0.0583		
	ETNDAISAAYDAYGSWSK	0.15	0.0356		
	IIVQEGIYEK	0.78	0.8124		
	ITFTGSTAVGK	0.42	0.0255		
	YGMDEYLEIK	0.31	0.0259		
Glyma18g49210.1	Galactose mutarotaselike superfamily protein	2	0.4	0.0339	minor CHO metabolism
	Peptide Sequnce	Ratio 4(0)	Pvalue 4(0)		
	GYDINYVIDGEK	0.55	0.2052		
	IGIFELK	0.12	0.0266		
Glyma16g30190.1	tripeptidyl peptidase ii	12	0.4	0.0007	protein
	Peptide Sequnce	Ratio 4(0)	Pvalue 4(0)		
	AALDTQSLEDDPNCGK+Carbamidomethyl(14)	0.30	0.0720		
	ALENTAIPIGDLPEDK	0.31	0.0491		

	FFDAHPEYDGR	0.31	0.0461		
	FVDLVNEVVNK	0.25	0.0645		
	FYVDAVQLCPLR+Carbamidomethyl(9)	0.41	0.1298		
	GALIAIFDSGVDPAADGLQITSDGKPK	1.43	0.6547		
	GLALAEIESLK	0.26	0.1170		
	LANFMPLTNYR	0.31	0.1747		
	MLFQPGHIER	0.63	0.2831		
	SQGDLFEENFK	0.24	0.0928		
	VLDVIDCTGSGDIDTSK+Carbamidomethyl(7)	0.23	0.0517		
	VYSSGDVYPSSSNLPK	0.25	0.1693		
Glyma09g00660.2	glyoxalase I homolog	8	0.41	0.0000	biodegradation of Xenobiotics
	Peptide Sequnce	Ratio 4(0)	Pvalue 4(0)		
	DPDGYAFELIQR	0.38	0.0224		
	FLHVVYR	0.58	0.1735		
	FYTECFGMK+Carbamidomethyl(5)	0.34	0.1417		
	GNAYAQVAIGTDDVYK	0.38	0.0850		
	ITSFLDPDGWK	0.15	0.0565		
	SAEVVNIVTQELGGK	0.37	0.0003		
	SSTPEPLCQVMLR+Carbamidomethyl(8)	0.55	0.0621		
	TVLVDNQDFLK	0.34	0.0295		
Glyma13g44261.1	Cystathionine betasynthase (CBS) protein	3	0.41	0.0026	not assigned
	Peptide Sequnce	Ratio 4(0)	Pvalue 4(0)		
	AGEGDGELQSILSR	0.11	0.0351		
	ELVTCFAESPLSEVIEK+Carbamidomethyl(5)	0.02	0.0071		
	LIGTFSSTDLR	0.65	0.1665		
Glyma12g34000.1	Ras related small GTP binding family protein	2	0.42	0.0230	signalling
	Peptide Sequnce	Ratio 4(0)	Pvalue 4(0)		
	AQIWDTAGQER	0.91	0.7176		
	LVLIGDSGVGK	0.32	0.0048		
Glyma13g36530.1	Ras related small GTP binding family protein	2	0.42	0.0230	signalling
	Peptide Sequnce	Ratio 4(0)	Pvalue 4(0)		
	AQIWDTAGQER	0.91	0.7176		
	LVLIGDSGVGK	0.32	0.0048		
Glyma09g27870.1	Embryo specific protein 3 (ATS3)	2	0.42	0.0004	development
	Peptide Sequnce	Ratio 4(0)	Pvalue 4(0)		
	IGITFGDANGNQVYEPR	0.48	0.0028		

	SGAEEGWEPESVK	0.31	0.0118		
Glyma13g42320.1	lipoxygenase 1	23	0.42	0.0000	hormone metabolism
	Peptide Sequnce	Ratio 4(0)	Pvalue 4(0)		
	EGVESTIWLLAK	0.22	0.0181		
	EMIAGVNPCVIR+Carbamidomethyl(9)	0.11	0.0821		
	FPQPHVVQVSQSAWMTDEEFAR	0.02	0.0039		
	FVCNSWVYNTK+Carbamidomethyl(3)	0.05	0.0474		
	GTVVLMPK	0.49	0.4613		
	HLSVLHPIYK	0.41	0.0027		
	IFFANHTYVPSETPAPLVEYR	0.02	0.0008		
	IFFANHTYVPSETPAPLVEYREEELK	1.18	0.6012		
	ITADSLDLDGYTMDEALGSR	0.02	0.0232		
	IYDYDVYNDLGNPDK	0.11	0.0102		
	NDSELQHWWK	0.05	0.0169		
	NELEVNPDGSAVDNLNAFLGR	0.03	0.0087		
	NNMNINALAR	0.14	0.0933		
	NWVFTDQALPADLIK	5.08	0.2102		
	NYMQVEFFLK	0.16	0.1331		
	QGEVFYVPR	0.01	0.0313		
	QSLINANGIIETTFLPSK	0.00	1.0000		
	SLSQIVQPAFESAFDLK	2.32	0.1380		
	SNLDPAIYGDQSSK	0.03	0.0198		
	STPIEFHSFQDVHDLYEGGIK	0.10	0.0002		
	SVSLQLISATK	0.02	0.0537		
	TWVQEYVPLYYAR	0.02	0.0485		
	YSVEMSSAVYK	0.03	0.0519		
Glyma05g26403.1	1-cysteine peroxiredoxin 1	5	0.43	0.0189	redox
	Peptide Sequnce	Ratio 4(0)	Pvalue 4(0)		
	DIEAYTPGAK	0.47	0.2798		
	LLGLSCDDVQSHNEWIK+Carbamidomethyl(6)	0.81	0.2112		
	PGITIGDTIPDLQVETNQGK	0.19	0.0097		
	VNYPIIADPK	0.08	0.0814		
	VNYPIIADPKR	0.33	0.5014		
Glyma09g25250.1	tripeptidyl peptidase ii	10	0.43	0.0017	protein
	Peptide Sequnce	Ratio 4(0)	Pvalue 4(0)		
	ALENTAIPIGDLPEDK	0.31	0.0491		
	FFDAHPEYDGR	0.31	0.0461		

	FVDLVNEVVNK	0.25	0.0645		
	FYVDAVQLCPLR+Carbamidomethyl(9)	0.41	0.1298		
	GALIAIFDSGVDPAADGLQITSDGKPK	1.43	0.6547		
	GLALAEIESLK	0.26	0.1170		
	MLFQPGHIER	0.63	0.2831		
	SQGDLFEENFK	0.24	0.0928		
	VLDVIDCTGSGDIDTSK+Carbamidomethyl(7)	0.23	0.0517		
	VYSSGDVYPSSSNLPK	0.25	0.1693		
Glyma01g18885.1	RAB GTPase homolog A4D	3	0.43	0.0473	signalling
	Peptide Sequnce	Ratio 4(0)	Pvalue 4(0)		
	AQIWDTAGQER	0.91	0.7176		
	VVLIGDSAVGK	0.32	0.0048		
	WLEELR	0.70	0.4942		
Glyma10g12110.1	RAB GTPase homolog A4D	3	0.43	0.0473	signalling
	Peptide Sequnce	Ratio 4(0)	Pvalue 4(0)		
	AQIWDTAGQER	0.91	0.7176		
	VVLIGDSAVGK	0.32	0.0048		
	WLEELR	0.70	0.4942		
Glyma11g17460.1	RAB GTPase homolog A4D	3	0.43	0.0473	signalling
	Peptide Sequnce	Ratio 4(0)	Pvalue 4(0)		
	AQIWDTAGQER	0.91	0.7176		
	VVLIGDSAVGK	0.32	0.0048		
	WLEELR	0.70	0.4942		
Glyma13g01260.1	xylose isomerase family protein	3	0.43	0.0125	minor CHO metabolism
	Peptide Sequnce	Ratio 4(0)	Pvalue 4(0)		
	ANFEFISK	0.43	0.1608		
	LGVDFWCFHDR+Carbamidomethyl(7)	0.47	0.0390		
	WYNAEEEILGK	0.27	0.0475		
Glyma19g00870.2	Pyruvate kinase family protein	12	0.44	0.0005	glycolysis
	Peptide Sequnce	Ratio 4(0)	Pvalue 4(0)		
	ATDAESTEVILEAALK	0.16	0.1010		
	DKEDILQWGVPNK	0.92	0.8863		
	EGQEITITTDYDIK	0.40	0.1515		
	FNFSHGTHDYHQETLNNLK	0.62	0.5359		
	GLIPILGEGSAK	0.45	0.0316		
	ICIEAESSLDYGAIFK+Carbamidomethyl(2)	0.36	0.2204		

	IDMIALSFVR	0.65	0.4035		
	IVCTLGPASR+Carbamidomethyl(3)	0.95	0.9195		
	LIVVLTR	0.32	0.0596		
	NVNLPGVVVDLPTLTEK	0.25	0.0021		
	STPLPMSPLESLASSAVR	0.25	0.1459		
	VENQEGVLNFDEILR	0.38	0.0077		
Glyma08g13440.1	RmlC like cupins superfamily protein	6	0.44	0.0000	development
	Peptide Sequnce	Ratio 4(0)	Pvalue 4(0)		
	APSYPINPTVQLIYIAR	0.44	0.0000		
	IEIVDFSGK	0.48	0.0109		
	LVLQPQGFAIPHYSDISK	0.64	0.3123		
	LVYNIDVAHPENVVENAGIVK	0.38	0.0018		
	TAEVLFEGDGGGYYTWWSSK	0.76	0.8905		
	VGYVLQGNDGVAGMALR	0.40	0.0481		
Glyma08g05200.3	glutathione peroxidase 3	3	0.45	0.0003	redox
	Peptide Sequnce	Ratio 4(0)	Pvalue 4(0)		
	AEFPIFDK	0.12	0.0007		
	DISGNDVSLNDYSGK	0.37	0.0104		
	GGIFGDGIK	1.33	0.5100		
Glyma11g14360.1	RAB GTPase homolog A2B	4	0.45	0.0001	signalling
	Peptide Sequnce	Ratio 4(0)	Pvalue 4(0)		
	AQIWDTAGQER	0.91	0.7176		
	EGLSFLETSALEATNIEK	0.06	0.0001		
	IVLIGDSGVGK	0.32	0.0048		
	STIGVEFATR	1.20	0.5762		
Glyma12g06280.2	RAB GTPase homolog A2B	4	0.45	0.0001	signalling
	Peptide Sequnce	Ratio 4(0)	Pvalue 4(0)		
	AQIWDTAGQER	0.91	0.7176		
	EGLSFLETSALEATNIEK	0.06	0.0001		
	IVLIGDSGVGK	0.32	0.0048		
	STIGVEFATR	1.20	0.5762		
Glyma05g03320.1	purple acid phosphatase 27	3	0.46	0.0213	misc
	Peptide Sequnce	Ratio 4(0)	Pvalue 4(0)		
	DPGFIHTSFLK	0.36	0.0524		
	TCPIYQNQCVNDER+Carbamidomethyl(2)Carbamidomethyl(9)	0.52	0.0651		
	VIIFGDMGK	0.44	0.1727		

Glyma02g15070.1	ARP protein (REF)	6	0.47	0.0055	stress
	Peptide Sequnce	Ratio 4(0)	Pvalue 4(0)		
	GAFELIMDESK	0.35	0.0251		
	GLALALAEK	0.68	0.3037		
	IIFAGVNASDVNFSSGR	0.47	0.1589		
	LGFPSAIFVK	0.25	0.0532		
	VNVLCPEFVETEMGNK+Carbamidomethyl(5)	0.52	0.2853		
	VVVSVDPSFLPQVAK	0.32	0.0451		
Glyma17g13361.1	RAB GTPase homolog E1B	2	0.47	0.0012	protein
	Peptide Sequnce	Ratio 4(0)	Pvalue 4(0)		
	FSAIVYVLK	0.61	0.1380		
	TVGAGVIQSIIE	0.44	0.0009		
Glyma16g07450.1	GDSL like Lipase/Acylhydrolase superfamily protein	3	0.47	0.0135	misc
	Peptide Sequnce	Ratio 4(0)	Pvalue 4(0)		
	ALYTFDIGQNDLSVGFR	0.18	0.0417		
	DQNVMATEFNK	0.67	0.3965		
	MNFDQIR	0.53	0.0198		
Glyma17g03600.1	Haem oxygenase like multi helical	5	0.47	0.0011	not assigned
	Peptide Sequnce	Ratio 4(0)	Pvalue 4(0)		
	HPLIISIR	0.05	0.0601		
	IGMTETWLK	0.08	0.1053		
	TWLAQDYLFVR	0.42	0.1305		
	WGISLSDVVPQQANK	0.23	0.0280		
	YCQSLQNIANR+Carbamidomethyl(2)	0.58	0.0191		
Glyma08g08970.1	urease accessory protein G	5	0.47	0.0001	amino acid metabolism
	Peptide Sequnce	Ratio 4(0)	Pvalue 4(0)		
	ADILLCESGGDNLAANFSR+Carbamidomethyl(6)	0.15	0.0088		
	AFTVGIGGPVGTGK	0.43	0.0787		
	ENYSLAAVTNDIFTK	0.49	0.1044		
	GGPGITQADLLVINK	0.49	0.0132		
	TDLAPAIGADLAVMQR	0.23	0.0280		
Glyma13g41650.1	Leucine rich repeat (LRR) family protein	13	0.47	0.0001	not assigned
	Peptide Sequnce	Ratio 4(0)	Pvalue 4(0)		
	CITTLPFLR+Carbamidomethyl(1)	0.44	0.0617		
	IVDLIGNR	0.42	0.0598		
	LADLDLSR	0.60	0.1326		

	LSGAIPSSVSQIYR	0.45	0.0030		
	LSGSIPAGIGR	0.36	0.0903		
	LSSITIADWK	0.46	0.0459		
	MAVLSTLNLDMNK	0.62	0.4655		
	NALEGNIPDAFGVR	0.42	0.0979		
	NFGSLSMLSR	1.30	0.5518		
	NNLFSGPIPR	0.31	0.0993		
	NQISGPIPESLGK	0.41	0.0056		
	SYFTALDLSYNNLK	1.06	0.7914		
	TGYMTGYISPAICK+Carbamidomethyl(13)	0.57	0.2785		
Glyma19g44540.1	Eukaryotic aspartyl protease family protein	8	0.48	0.0000	protein
	Peptide Sequnce	Ratio 4(0)	Pvalue 4(0)		
	CYTQADPVFDPTK+Carbamidomethyl(1)	0.47	0.0102		
	FSYCLVDR+Carbamidomethyl(4)	0.48	0.0311		
	GLSASLFR	0.49	0.0269		
	LSFPVQTGR	0.53	0.0643		
	SGSSFSSSIISGLAQGSGEYFTR	0.48	0.0560		
	TYAGIPCGAPLCR+Carbamidomethyl(7)Carbamidomethyl(12)	0.46	0.0002		
	VPTVVLHFR	0.75	0.3843		
	VSFDLAGSR	0.49	0.0357		
Glyma03g41880.1	Eukaryotic aspartyl protease family protein	5	0.48	0.0000	protein
	Peptide Sequnce	Ratio 4(0)	Pvalue 4(0)		
	FSYCLVDR+Carbamidomethyl(4)	0.48	0.0311		
	GLSASLFR	0.49	0.0269		
	LSFPVQTGR	0.53	0.0643		
	TYAGIPCGAPLCR+Carbamidomethyl(7)Carbamidomethyl(12)	0.46	0.0002		
	VPTVVLHFR	0.75	0.3843		
Glyma08g03310.1	ACC oxidase 1	3	0.48	0.0046	hormone metabolism
	Peptide Sequnce	Ratio 4(0)	Pvalue 4(0)		
	LLYPSNFR	0.39	0.0500		
	LSELMSENLGLEK	0.45	0.0139		
	YPQCPRPELVR+Carbamidomethyl(4)	0.64	0.1206		
Glyma08g22380.1	Protein of unknown function DUF642	2	0.48	0.0190	not assigned
	Peptide Sequnce	Ratio 4(0)	Pvalue 4(0)		
	VEIVIHNPGVDEDPACGPLIDSVALK+Carbamidomethyl(16)	0.05	0.0896		

	YIDSDHFAVPEGK	0.51	0.0308		
Glyma18g18910.1	aldehyde dehydrogenase 2B4	14	0.48	0.0000	fermentation
	Peptide Sequnce	Ratio 4(0)	Pvalue 4(0)		
	DEIFGPVQSILK	0.17	0.0585		
	GFYIQPTVFSNVK	0.47	0.0766		
	GGIEQGPQIDSDQFEK	0.36	0.0343		
	IEVPMLVR	0.39	0.2101		
	SGVESGATLETGGDK	8.59	0.2488		
	SGVESGATLETGGDKLGNK	0.00	0.4215		
	SNLKPVTLELGGK	0.39	0.1121		
	TAEQTPLSALYAAK	0.70	0.3243		
	TFPTLDPR	0.29	0.0296		
	TGEVIAHVAEGHSEDVDR	0.35	0.0002		
	VGPALACGNTIVLK+Carbamidomethyl(7)	0.52	0.0097		
	VVGDPFKGGIEQGPQIDSDQFEK	0.01	0.8208		
	VVLELAAK	0.40	0.1145		
	YGLAAGVFTTNMDTAYTLTR	0.38	0.2709		
Glyma07g38330.1	2 oxoglutarate (2OG) and Fe(II)dependent oxygenase superfamily protein	2	0.49	0.0458	misc
	Peptide Sequnce	Ratio 4(0)	Pvalue 4(0)		
	ISSWTFLPK	0.70	0.2170		
	SAVADNLSGESQLSDVR	0.35	0.0361		
Glyma17g02430.1	2 oxoglutarate (2OG) and Fe(II)dependent oxygenase superfamily protein	2	0.49	0.0458	misc
	Peptide Sequnce	Ratio 4(0)	Pvalue 4(0)		
	ISSWTFLPK	0.70	0.2170		
	SAVADNLSGESQLSDVR	0.35	0.0361		
Glyma04g01130.1	cold-regulated 47	3	0.49	0.0076	stress
	Peptide Sequnce	Ratio 4(0)	Pvalue 4(0)		
	GVFDFLGK	0.58	0.1120		
	VEVVETAHAEEK	0.22	0.0386		
	YESSEVEVQDR	0.18	0.0364		
Glyma08g39770.1	aldehyde dehydrogenase 2B4	13	0.49	0.0000	fermentation
	Peptide Sequnce	Ratio 4(0)	Pvalue 4(0)		
	DEIFGPVQSILK	0.17	0.0585		
	GFYIQPTVFSNVK	0.47	0.0766		
	GGIEQGPQIDSDQFEK	0.36	0.0343		
	IEVPMLVR	0.39	0.2101		

	SGVESGATLETGGDK	8.59	0.2488		
	SGVESGATLETGGDKLGNK	0.00	0.4215		
	SNLKPVTLELGGK	0.39	0.1121		
	TAEQTPLSALYAAK	0.70	0.3243		
	TGEVIAHVAEGHSEDVDR	0.35	0.0002		
	VGPALACGNTIVLK+Carbamidomethyl(7)	0.52	0.0097		
	VVGDPFKGGIEQGPQIDSDQFEK	0.01	0.8208		
	VVLELAAK	0.40	0.1145		
	YGLAAGVFTK	0.50	0.1312		
Glyma07g09240.1	thioredoxin dependent peroxidase 1	4	0.49	0.0003	redox
	Peptide Sequnce	Ratio 4(0)	Pvalue 4(0)		
	FALLVEDLK	0.42	0.0084		
	VANVESGGEFTISSAEEIIK	0.48	0.0071		
	VIIFGVPGAFTPTCSLK+Carbamidomethyl(14)	0.59	0.1617		
	YTNALGLELDLTDK	0.54	0.0454		
Glyma09g32540.1	thioredoxin dependent peroxidase 1	4	0.49	0.0003	redox
	Peptide Sequnce	Ratio 4(0)	Pvalue 4(0)		
	FALLVEDLK	0.42	0.0084		
	VANVESGGEFTISSAEEIIK	0.48	0.0071		
	VIIFGVPGAFTPTCSLK+Carbamidomethyl(14)	0.59	0.1617		
	YTNALGLELDLTDK	0.54	0.0454		
Glyma17g09860.1	aldehyde dehydrogenase 2B4	3	0.5	0.0070	fermentation
	Peptide Sequnce	Ratio 4(0)	Pvalue 4(0)		
	SNLKPVTLELGGK	0.39	0.1121		
	VGPALACGNTIVLK+Carbamidomethyl(7)	0.52	0.0097		
	YGLAAGVFTK	0.50	0.1312		
Glyma04g02360.1	Thioredoxin superfamily protein	2	0.5	0.0287	not assigned
	Peptide Sequnce	Ratio 4(0)	Pvalue 4(0)		
	FDGFVYGK	0.73	0.3031		
	YQENFYGAQTR	0.37	0.0148		
Glyma12g01770.2	GroES-like zincbinding dehydrogenase family protein	2	0.5	0.0163	misc
	Peptide Sequnce	Ratio 4(0)	Pvalue 4(0)		
	AISDLSILADK	0.54	0.0569		
	GEAFGITDFINPGDSNK	0.39	0.0406		
Glyma12g01790.1	Zinc binding alcohol dehydrogenase family protein	2	0.5	0.0163	misc
	Peptide Sequnce	Ratio 4(0)	Pvalue 4(0)		

	AISDLSILADK	0.54	0.0569		
	GEAFGITDFINPGDSNK	0.39	0.0406		
Glyma16g27880.1	Peroxidase superfamily protein	9	2.05	0.0000	misc
	Peptide Sequnce	Ratio 4(0)	Pvalue 4(0)		
	DSVFLTGGPDYAVPLGR	1.51	0.1239		
	GLSYSFYSK	2.42	0.0652		
	IFFHDCFVQGCDGSLLLDGSPSER+Carbamidomethyl(6)Carbamidomethyl(11)	13.78	0.0007		
	IVSCADITVLAAR+Carbamidomethyl(4)	3.35	0.1247		
	LSPLDPNMDK	0.60	0.5572		
	LSQLDVLTGNQGEIR	2.04	0.0991		
	NFDVTDVVALSGAHTFGR	1.88	0.0025		
	TEALQTIDDIR	1.26	0.1932		
	YYLDLMNR	1.94	0.1023		
Glyma12g32170.1	Peroxidase superfamily protein	11	2.06	0.0062	misc
	Peptide Sequnce	Ratio 4(0)	Pvalue 4(0)		
	AQIIELLEGSVEK	1.94	0.2690		
	DGVISNLVEAR	2.11	0.3794		
	DLVLLSGAHTIGIAHCSSLSNR+Carbamidomethyl(16)	2.39	0.1476		
	DSIVATGGPYWK	2.63	0.0749		
	FFAEFATSIEK	1.92	0.1381		
	GDQDPSLDSEYAANLK	1.95	0.1615		
	GFDFIDR	2.44	0.1898		
	MHFHDCFVR+Carbamidomethyl(6)	2.49	0.1574		
	MHFHDCFVR+Carbamidomethyl(6)Oxidation(1)	4.66	0.0659		
	SLVEAECPGVVSCADILTLASR+Carbamidomethyl(7)Carbamidomethyl(13)	2.39	0.1261		
	TFDLSYYSHVIK	0.96	0.1211		
Glyma13g38300.1	Peroxidase superfamily protein	8	2.1	0.0164	misc
	Peptide Sequnce	Ratio 4(0)	Pvalue 4(0)		
	DGVISNLVEAR	2.11	0.3794		
	DLVLLSGAHTIGIAHCSSLSNR+Carbamidomethyl(16)	2.39	0.1476		
	GDQDPSLDSEYAANLK	1.95	0.1615		
	GFDFIDR	2.44	0.1898		
	MHFHDCFVR+Carbamidomethyl(6)	2.49	0.1574		
	MHFHDCFVR+Carbamidomethyl(6)Oxidation(1)	4.66	0.0659		
	SLVEAECPGVVSCADILTLAAR+Carbamidomethyl(7)Carbamidomethyl(13)	2.40	0.1201		
	TFDLSYYSHVIK	0.96	0.1211		
Glyma13g38310.2	Peroxidase superfamily protein	8	2.11	0.0000	stress

	Peptide Sequnce	Ratio 4(0)	Pvalue 4(0)		
	DLVLLSGAHTIGIAHCSSLSNR+Carbamidomethyl(16)	2.39	0.1476		
	DTIVATGGPFWK	2.20	0.1497		
	GDQDPSLDSEYAANLK	1.95	0.1615		
	GFDFIDR	2.44	0.1898		
	MHFHDCFVR+Carbamidomethyl(6)	2.49	0.1574		
	MHFHDCFVR+Carbamidomethyl(6)Oxidation(1)	4.66	0.0659		
	SLVEAECPGVVSCADILTLAAR+Carbamidomethyl(7)Carbamidomethyl(13)	2.40	0.1201		
	TFDLSYYSHVIK	0.96	0.1211		
Glyma10g02730.1	Peroxidase superfamily protein	7	2.37	0.0136	stress
	Peptide Sequnce	Ratio 4(0)	Pvalue 4(0)		
	DSCPQAEDIIK+Carbamidomethyl(3)	1.77	0.2271		
	FFTEFAQSMK	1.61	0.0827		
	GLFQSDAALLTQEQSEDIAK	8.17	0.1484		
	MGAIEVLTGSAGEIR	5.05	0.1618		
	MHFHDCFVR+Carbamidomethyl(6)	2.49	0.1574		
	MHFHDCFVR+Carbamidomethyl(6)Oxidation(1)	4.66	0.0659		
	TVSCADILALAAR+Carbamidomethyl(4)	1.98	0.1646		
Glyma07g15610.1	Unknown protein	2	2.56	0.0306	not assigned
	Peptide Sequnce	Ratio 4(0)	Pvalue 4(0)		
	ENLEFNFR	2.84	0.0796		
	FIEDWGTAR	2.24	0.0608		
Glyma09g30910.1	B-S glucosidase 44	8	3.15	0.0023	misc
	Peptide Sequnce	Ratio 4(0)	Pvalue 4(0)		
	ANSYWLYNVPWGMYK	1.38	0.6307		
	DFADYAEFCFK+Carbamidomethyl(9)	0.20	0.0432		
	FGIVYVDFK	0.20	0.0332		
	FSISWSR	0.43	0.1002		
	GFLFGTATSAYQVEGMAHK	0.31	0.0158		
	GPSIWDVFIK	22.89	0.4557		
	NWMTFNEPR	0.31	0.0946		
	VVAALGYDNGFFAPGR	0.32	0.1765		
Glyma06g09830.1	Eukaryotic aspartyl protease family protein	7	3.86	0.0273	RNA
	Peptide Sequnce	Ratio 4(0)	Pvalue 4(0)		
	ASTSYGPLDCSVPQCGQVR+Carbamidomethyl(10)Carbamidomethyl(15)	2.10	0.3476		
	FVEPVYNAVR	2.49	0.1311		

	ILFDIVNNK	2.95	0.1211		
	QVGGTTFTSIGAFDTCFVK+Carbamidomethyl(16)	2.67	0.0645		
	SYYFSGSLK	2.42	0.0652		
	TVSTAPIASGQAFNIGNYVVR	3.72	0.3967		
	TYETLAPPITLHFEGLDLK	16.53	0.2684		
Glyma02g19380.1	copper chaperone	4	4.38	0.0268	metal handling
	Peptide Sequnce	Ratio 4(0)	Pvalue 4(0)		
	GNVEPDEVLQAVSK	2.05	0.1301		
	MEGVESFDIDLK	2.98	0.0345		
	TAFWVDEAPQSK	1.63	0.4110		
	VGMSCQGCAGAVNR+Carbamidomethyl(5)Carbamidomethyl(8)	20.02	0.0931		
Glyma10g14110.1	copper chaperone	3	5.11	0.0061	metal handling
	Peptide Sequnce	Ratio 4(0)	Pvalue 4(0)		
	GNVQPDEVLQAVSK	2.45	0.0375		
	MEGVESFDIDLK	2.98	0.0345		
	VGMSCQGCAGAVNR+Carbamidomethyl(5)Carbamidomethyl(8)	20.02	0.0931		

Protein ID, according to the Phytozome database; M.P., number of matched peptide; Ratio, relative abundance of protein; Function, functional classification by MapMan bin code; *N.D., Not Description in Phytozome database.

Supplemental Table 3. List of identified root proteins that were differentially changed in soybeans exposed to 2-day flooding stress without calcium compared to 2-day-old untreated soybeans.

Protein ID	Description	M.P.	Ratio 4(2)F/2(0)	Pvalue 4(2)F/2(0)	Function
Glyma11g15040.3	RNA binding (RRM/RBD/RNP motifs) family protein	3	0.03	0.0292	RNA
	Peptide Sequnce	Ratio 4(2)F	Pvalue 4(2)F		
	IEIVGTNISTPGVAPAR	0.00	1.0000		
	LYISNLDYGVSSDDIK	0.00	1.0000		
	VSADDLDADLEK	0.09	0.0009		
Glyma19g21200.1	ATPase AAA type CDC48 protein	3	0.03	0.0001	cell
	Peptide Sequnce	Ratio 4(2)F	Pvalue 4(2)F		
	AIANECQANFISVK+Carbamidomethyl(6)	0.03	0.0603		
	EIDIGVPDEVGR	0.06	0.0576		
	ETVVEVPNVSWEDIGGLENVK	0.02	0.0002		
Glyma18g14826.1	ATPase AAA type CDC48 protein	2	0.03	0.0236	cell
	Peptide Sequnce	Ratio 4(2)F	Pvalue 4(2)F		
	DIALAEIEK	0.269094384	0.242956514		
	GVLFYGPPGCGK+Carbamidomethyl(10)	0.041102763	0.065808508		
Glyma20g33270.1	Coatomer alpha subunit	2	0.04	0.0213	cell
	Peptide Sequnce	Ratio 4(2)F	Pvalue 4(2)F		
	GFPEVALHFVK	0.04	0.0201		
	VFIFDLQQR	0.04	0.1574		
Glyma01g00740.3	Ribosomal protein L31e family protein	2	0.05	0.0298	protein
	Peptide Sequnce	Ratio 4(2)F	Pvalue 4(2)F		
	EELYSLVTVVEIPK	0.01	0.1134		
	FVWSQGIR	0.06	0.0414		
Glyma12g08410.2	ATPase AAA type CDC48 protein	8	0.05	0.0000	cell
	Peptide Sequnce	Ratio 4(2)F	Pvalue 4(2)F		
	AIANECQANFISVK+Carbamidomethyl(6)	0.03	0.0603		
	DTHGYVGADLAAICTEAALQCIR+Carbamidomethyl(14)Carbamidomethyl(21)	0.02	0.0541		
	EIDIGVPDEVGR	0.06	0.0576		
	ELQETVQYPVEHPEK	0.07	0.2833		
	ELVELPLR	0.02	0.0982		
	ETVVEVPNVSWEDIGGLENVK	0.02	0.0002		
	GILLYGPPGSGK	0.07	0.0858		
	GVLFYGPPGCGK+Carbamidomethyl(10)	0.04	0.0658		

Glyma11g17930.1	DNAJ homologue 2	3	0.05	0.0091	stress
	Peptide Sequnce	Ratio 4(2)F	Pvalue 4(2)F		
	EIYDQYGEDALK	0.03	0.0688		
	VSLEDLYLGTSK	0.01	0.0344		
	YYEILGVSK	0.09	0.0840		
Glyma12g10150.1	DNAJ homologue 2	3	0.05	0.0091	stress
	Peptide Sequnce	Ratio 4(2)F	Pvalue 4(2)F		
	EIYDQYGEDALK	0.03	0.0688		
	VSLEDLYLGTSK	0.01	0.0344		
	YYEILGVSK	0.09	0.0840		
Glyma10g12760.1	heat shock protein 81 2	3	0.05	0.0212	stress
	Peptide Sequnce	Ratio 4(2)F	Pvalue 4(2)F		
	APFDLFDTR	0.02	0.1254		
	EDQLEYLEER	0.10	0.0539		
	RAPFDLFDTR	0.02	0.0870		
Glyma04g38270.1	Protein kinase superfamily protein	2	0.05	0.0210	protein
	Peptide Sequnce	Ratio 4(2)F	Pvalue 4(2)F		
	LENTLLDGSPAPR	0.03	0.0496		
	STVGTPAYIAPEVLSR	0.06	0.0624		
Glyma05g33170.1	Protein kinase superfamily protein	2	0.05	0.0210	protein
	Peptide Sequnce	Ratio 4(2)F	Pvalue 4(2)F		
	LENTLLDGSPAPR	0.03	0.0496		
	STVGTPAYIAPEVLSR	0.06	0.0624		
Glyma06g16780.1	Protein kinase superfamily protein	2	0.05	0.0210	protein
	Peptide Sequnce	Ratio 4(2)F	Pvalue 4(2)F		
	LENTLLDGSPAPR	0.03	0.0496		
	STVGTPAYIAPEVLSR	0.06	0.0624		
Glyma08g00770.1	Protein kinase superfamily protein	2	0.05	0.0210	protein
	Peptide Sequnce	Ratio 4(2)F	Pvalue 4(2)F		
	LENTLLDGSPAPR	0.03	0.0496		
	STVGTPAYIAPEVLSR	0.06	0.0624		
Glyma08g20090.3	SNF1 related protein kinase 2.10	2	0.05	0.0210	protein
	Peptide Sequnce	Ratio 4(2)F	Pvalue 4(2)F		
	LENTLLDGSPAPR	0.03	0.0496		

	STVGTPAYIAPEVLSR	0.06	0.0624		
Glyma12g29130.1	SNF1 related protein kinase 2.10	2	0.05	0.0210	protein
	Peptide Sequnce	Ratio 4(2)F	Pvalue 4(2)F		
	LENTLLDGSPAPR	0.03	0.0496		
	STVGTPAYIAPEVLSR	0.06	0.0624		
Glyma06g03050.1	Papain family cysteine protease	2	0.05	0.0016	protein
	Peptide Sequnce	Ratio 4(2)F	Pvalue 4(2)F		
	APILPTNDLPTDFDWR	0.01	0.0034		
	NSWGESWGEEGYYK	0.10	0.0495		
Glyma05g04220.1	stress inducible protein putative	3	0.06	0.0024	stress
	Peptide Sequnce	Ratio 4(2)F	Pvalue 4(2)F		
	ALELDDEDISYLTNR	0.06	0.0085		
	ELEQQEYFDPK	0.04	0.0874		
	LGAMPEGLK	0.05	0.0519		
Glyma17g14660.1	stress inducible protein putative	3	0.06	0.0024	stress
	Peptide Sequnce	Ratio 4(2)F	Pvalue 4(2)F		
	ALELDDEDISYLTNR	0.063362928	0.00846776		
	ELEQQEYFDPK	0.042931637	0.087399961		
	LGAMPEGLK	0.050021474	0.051881165		
Glyma20g35120.5	S adenosyl L methionine dependent methyltransferases superfamily protein	3	0.06	0.0053	stress
	Peptide Sequnce	Ratio 4(2)F	Pvalue 4(2)F		
	DGILLLELDR	0.083674143	0.04536283		
	GIPAYLGVLGTK	0.083835436	0.040731282		
	MWLTSESFR	0.027213496	0.05434305		
Glyma07g00620.1	O fucosyltransferase family protein	7	0.06	0.0000	not assigned
	Peptide Sequnce	Ratio 4(2)F	Pvalue 4(2)F		
	NFEDIYDVDVFMK	0.00	1.0000		
	SCFNAQEVAVFLR+Carbamidomethyl(2)	0.08	0.0001		
	SLGATLVIPDIR	0.09	0.0155		
	SQILVPANIPDSSASASSFLSHYVSK	0.06	0.0342		
	VTEDYIAQHVEPIYR	0.17	0.0600		
	WDESLDSLK	0.01	0.0387		
	YLDSEDSELEK	0.01	0.0378		
Glyma01g42010.3	stress inducible protein putative	9	0.06	0.0000	stress

	Peptide Sequnce	Ratio 4(2)F	Pvalue 4(2)F		
	ALELDDEDISYLTNR	0.06	0.0085		
	DYESAIETYQK	0.39	0.2360		
	ELEQQEYFDPK	0.04	0.0874		
	GLEIDPHNEPLK	0.00	1.0000		
	IMQALGVLLNVK	0.02	0.0320		
	LGAMPEGLK	0.05	0.0519		
	LVSAGIVQMK	0.02	0.0341		
	QVLIDFQENPK	0.09	0.0036		
	YDSNNQELLEGIR	0.07	0.0001		
Glyma01g21660.1	Transducin/WD40 repeat like superfamily protein	2	0.07	0.0211	not assigned
	Peptide Sequnce	Ratio 4(2)F	Pvalue 4(2)F		
	FFLTGCYDGLGR+Carbamidomethyl(6)	0.18	0.0931		
	MSLEQFLLAK	0.05	0.0334		
Glyma05g01170.1	Transducin/WD40 repeat like superfamily protein	2	0.07	0.0211	not assigned
	Peptide Sequnce	Ratio 4(2)F	Pvalue 4(2)F		
	FFLTGCYDGLGR+Carbamidomethyl(6)	0.18	0.0931		
	MSLEQFLLAK	0.05	0.0334		
Glyma13g06140.1	Transducin/WD40 repeat like superfamily protein	2	0.07	0.0211	not assigned
	Peptide Sequnce	Ratio 4(2)F	Pvalue 4(2)F		
	FFLTGCYDGLGR+Carbamidomethyl(6)	0.18	0.0931		
	MSLEQFLLAK	0.05	0.0334		
Glyma19g09960.1	O fucosyltransferase family protein	3	0.07	0.0000	not assigned
	Peptide Sequnce	Ratio 4(2)F	Pvalue 4(2)F		
	SCFNAQEVAVFLR+Carbamidomethyl(2)	0.083058222	5.88863E-05		
	VTEDYIAQHVEPIYR	0.172962199	0.060028504		
	WDESLDSLK	0.01004556	0.03870514		
Glyma02g08690.1	S18 ribosomal protein	3	0.07	0.0279	protein
	Peptide Sequnce	Ratio 4(2)F	Pvalue 4(2)F		
	IMFALTSIK	0.00	1.0000		
	IPDWFLNR	0.05	0.0371		
	YSQVVSNALDMK	0.17	0.0227		
Glyma16g06171.1	regulatory particle triple A 1A	3	0.08	0.0184	protein
	Peptide Sequnce	Ratio 4(2)F	Pvalue 4(2)F		

	IDPSVTMMTVEEKPDVTYNDVGGCK+Carbamidomethyl(24)	0.06	0.2711		
	SVCTEAGMYAIR+Carbamidomethyl(3)	0.11	0.0234		
	VIGSELVQK	0.02	0.0771		
Glyma05g36930.1	glycosyl hydrolase 9C2	3	0.08	0.0251	misc
	Peptide Sequnce	Ratio 4(2)F	Pvalue 4(2)F		
	SLLFFEAQR	0.20	0.2219		
	TPGGLIFR	0.11	0.3088		
	WGTDYFIK	0.04	0.0107		
Glyma08g02610.1	glycosyl hydrolase 9C2	3	0.08	0.0251	misc
	Peptide Sequnce	Ratio 4(2)F	Pvalue 4(2)F		
	SLLFFEAQR	0.20	0.2219		
	TPGGLIFR	0.11	0.3088		
	WGTDYFIK	0.04	0.0107		
Glyma11g32827.1	Ribosomal S17 family protein	2	0.09	0.0332	protein
	Peptide Sequnce	Ratio 4(2)F	Pvalue 4(2)F		
	LLEEVAIIPSK	0.09	0.0271		
	LLEEVAIIPSKR	0.07	0.1964		
Glyma14g02395.1	Ribosomal S17 family protein	2	0.09	0.0332	protein
	Peptide Sequnce	Ratio 4(2)F	Pvalue 4(2)F		
	LLEEVAIIPSK	0.09	0.0271		
	LLEEVAIIPSKR	0.07	0.1964		
Glyma08g45425.1	eukaryotic translation initiation factor 4G	2	0.09	0.0032	protein
	Peptide Sequnce	Ratio 4(2)F	Pvalue 4(2)F		
	DTSVADQSNTGETYTGTR	0.06	0.1301		
	VNPTPVNSTESNSTYAAR	0.10	0.0028		
Glyma02g47330.1	seed imbibition 1	3	0.09	0.0017	minor CHO metabolism
	Peptide Sequnce	Ratio 4(2)F	Pvalue 4(2)F		
	DCLFTDPAR+Carbamidomethyl(2)	0.07	0.0512		
	LALPDGSILR	0.11	0.0564		
	VDVQNILETLGAGHGGR	0.09	0.0086		
Glyma08g25950.1	cytochrome P450 family 72 subfamily A polypeptide 15	4	0.09	0.0287	misc
	Peptide Sequnce	Ratio 4(2)F	Pvalue 4(2)F		
	LCIGQNFGLLEAK+Carbamidomethyl(2)	0.04	0.2346		
	LLASGFANYDGDK	0.23	0.0069		

	LSYLPFGWGPR	0.02	0.1173		
	SSFMWLGPTPR	0.00	1.0000		
Glyma02g10470.1	2 oxoglutarate dehydrogenase E1 component	7	0.09	0.0225	TCA / org transformation
	Peptide Sequnce	Ratio 4(2)F	Pvalue 4(2)F		
	APSAATATGFLK	0.29	0.1383		
	ASDLGVESIVIGMAHR	0.19	0.1546		
	FGLEGGETLIPGMK	0.04	0.1255		
	LDPLNLEPR	0.04	0.1686		
	LNVLGNVVR	0.05	0.1966		
	NLLESGELTQEEIDR	0.02	0.0939		
	SNLSEFDDVQGHPGFDK	0.08	0.2127		
Glyma18g52430.2	2 oxoglutarate dehydrogenase E1 component	7	0.1	0.0416	TCA / org transformation
	Peptide Sequnce	Ratio 4(2)F	Pvalue 4(2)F		
	APSAATATGFLK	0.29	0.1383		
	ASDLGVESIVIGMAHR	0.19	0.1546		
	FGLEGGETLIPGMK	0.04	0.1255		
	LDPLNLEPR	0.04	0.1686		
	LLESGELTQEEIDR	0.06	0.2731		
	LNVLGNVVR	0.05	0.1966		
	SNLSEFDDVQGHPGFDK	0.08	0.2127		
Glyma06g02330.1	sterol methyltransferase 2	5	0.1	0.0336	hormone metabolism
	Peptide Sequnce	Ratio 4(2)F	Pvalue 4(2)F		
	ANVVGITINEYQVNR	0.15	0.0565		
	LEEVYAEIFR	0.04	0.0700		
	MPFVDNSFDGAYSIEATCHAPK+Carbamidomethyl(18)	0.00	1.0000		
	QYWSFFR	0.04	0.0476		
	YSGDDPEHVEVIQGIER	0.42	0.2989		
Glyma09g08120.1	Subtilase family protein	2	0.1	0.0000	protein
	Peptide Sequnce	Ratio 4(2)F	Pvalue 4(2)F		
	ALSPGLVYDATPSDYIK	0.10	0.0007		
	DFPAYASLGNK	0.09	0.0007		
Glyma08g05570.1	Translation elongation factor EF1A	6	0.1	0.0139	signalling
	Peptide Sequnce	Ratio 4(2)F	Pvalue 4(2)F		
	EGDSLLVMPNK	0.25	0.0940		
	EIPSVQDEEDVPEMTK	0.00	1.0000		

	FSDFPQLGR	0.22	0.0469		
	MDEPTVQWSK	0.11	0.1820		
	STTGGQILFLSGQVDER	0.15	0.1004		
	VQVNNSICIEK+Carbamidomethyl(8)	0.08	0.0420		
Glyma05g31830.1	copper ion binding	5	0.1	0.0000	not assigned
	Peptide Sequnce	Ratio 4(2)F	Pvalue 4(2)F		
	DALEAMETQK	0.15	0.0502		
	FETAIGILK	0.16	0.0096		
	GMDLLLAEFDK	0.05	0.0415		
	TYLLTLK	0.01	0.0162		
	YEEELELK	0.05	0.0226		
Glyma08g15100.1	copper ion binding	5	0.1	0.0000	not assigned
	Peptide Sequnce	Ratio 4(2)F	Pvalue 4(2)F		
	DALEAMETQK	0.15	0.0502		
	FETAIGILK	0.16	0.0096		
	GMDLLLAEFDK	0.05	0.0415		
	TYLLTLK	0.01	0.0162		
	YEEELELK	0.05	0.0226		
Glyma14g34990.1	copper ion binding	5	0.1	0.0000	not assigned
	Peptide Sequnce	Ratio 4(2)F	Pvalue 4(2)F		
	DALEAMETQK	0.153489803	0.050169026		
	FETAIGILK	0.16283938	0.009577506		
	GMDLLLAEFDK	0.052274714	0.041548732		
	TYLLTLK	0.00785282	0.016180355		
	YEEELELK	0.047595691	0.022631365		
Glyma05g37150.1	vacuolar H+ ATPase subunit E isoform 3	2	0.1	0.0164	transport
	Peptide Sequnce	Ratio 4(2)F	Pvalue 4(2)F		
	DLIVQCLLR+Carbamidomethyl(6)	0.11	0.0186		
	IVCENTLDAR+Carbamidomethyl(3)	0.08	0.1243		
Glyma05g37160.1	vacuolar H+ ATPase subunit E isoform 3	2	0.1	0.0164	transport
	Peptide Sequnce	Ratio 4(2)F	Pvalue 4(2)F		
	DLIVQCLLR+Carbamidomethyl(6)	0.11	0.0186		
	IVCENTLDAR+Carbamidomethyl(3)	0.08	0.1243		
Glyma05g37190.1	vacuolar H+ ATPase subunit E isoform 3	2	0.1	0.0164	transport
	Peptide Sequnce	Ratio 4(2)F	Pvalue 4(2)F		

	DLIVQCLLR+Carbamidomethyl(6)	0.11	0.0186		
	IVCENTLDAR+Carbamidomethyl(3)	0.08	0.1243		
Glyma08g02390.1	vacuolar ATP synthase subunit E1	2	0.1	0.0164	transport
	Peptide Sequnce	Ratio 4(2)F	Pvalue 4(2)F		
	DLIVQCLLR+Carbamidomethyl(6)	0.11	0.0186		
	IVCENTLDAR+Carbamidomethyl(3)	0.08	0.1243		
Glyma17g09120.1	tRNA synthetase beta subunit family protein	3	0.1	0.0018	protein
	Peptide Sequnce	Ratio 4(2)F	Pvalue 4(2)F		
	FLHIIEDSPVFPVIYDSK	0.034400584	0.018761945		
	FVIEPVEVISSDGK	0.141614858	0.065940942		
	NVFIECTATDLTK+Carbamidomethyl(6)	0.12725336	0.02118786		
Glyma14g01430.1	seed imbibition 1	4	0.1	0.0001	minor CHO metabolism
	Peptide Sequnce	Ratio 4(2)F	Pvalue 4(2)F		
	DCLFTDPAR+Carbamidomethyl(2)	0.074182162	0.051196116		
	LALPDGSILR	0.107713567	0.056372546		
	VDVQNILETLGAGHGGR	0.08588351	0.008567828		
	VEDPALGLR	0.198328692	0.00664198		
Glyma04g02271.1	sterol methyltransferase 2	3	0.1	0.0087	hormone metabolism
	Peptide Sequnce	Ratio 4(2)F	Pvalue 4(2)F		
	ANVVGITINEYQVNR	0.15	0.0565		
	LEEVYAEIFR	0.04	0.0700		
	QYWSFFR	0.04	0.0476		
Glyma10g36610.2	Ribosomal protein S13/S18 family	2	0.11	0.0068	protein
	Peptide Sequnce	Ratio 4(2)F	Pvalue 4(2)F		
	IPDWFLNR	0.05	0.0371		
	YSQVVSNALDMK	0.17	0.0227		
Glyma04g07220.1	cellulose synthase 1	3	0.11	0.0048	cell wall
	Peptide Sequnce	Ratio 4(2)F	Pvalue 4(2)F		
	APEFYFAQK	0.03	0.0120		
	DLNSYGLGNVDWK	0.04	0.0322		
	NIVFFDINMK	0.37	0.2341		
Glyma06g07320.1	cellulose synthase 1	3	0.11	0.0048	cell wall
	Peptide Sequnce	Ratio 4(2)F	Pvalue 4(2)F		
	APEFYFAQK	0.03	0.0120		

	DLNSYGLGNVDWK	0.04	0.0322		
	NIVFFDINMK	0.37	0.2341		
Glyma08g46520.1	cytochrome P450 family 93 subfamily D polypeptide 1	2	0.11	0.0444	misc
	Peptide Sequnce	Ratio 4(2)F	Pvalue 4(2)F		
	GQYYQLLPFGSGR	0.16	0.0075		
	LPPGPPISIPLLGHAPYLR	0.00	1.0000		
Glyma03g29810.1	Ribosomal protein S10p/S20e family protein	2	0.11	0.0176	protein
	Peptide Sequnce	Ratio 4(2)F	Pvalue 4(2)F		
	SPCGEGTNTWDR+Carbamidomethyl(3)	0.01	0.3588		
	VIDLYSSPDVVK	0.15	0.0070		
Glyma12g00615.1	Ribosomal protein S10p/S20e family protein	2	0.11	0.0176	protein
	Peptide Sequnce	Ratio 4(2)F	Pvalue 4(2)F		
	SPCGEGTNTWDR+Carbamidomethyl(3)	0.01	0.3588		
	VIDLYSSPDVVK	0.15	0.0070		
Glyma15g13830.1	GTP binding	10	0.11	0.0003	not assigned
	Peptide Sequnce	Ratio 4(2)F	Pvalue 4(2)F		
	AFEDPDIIHVDDTVDPVR	0.08	0.0201		
	APQAAGAIHTDFER	0.19	0.0620		
	AVDGIFHVLR	0.33	0.1796		
	DLEVITEELR	0.16	0.0596		
	FEWLCQLFKPK+Carbamidomethyl(5)	0.00	1.0000		
	GFICAEVMK+Carbamidomethyl(4)	0.12	0.1875		
	IGIVGLPNVGK	0.06	0.0885		
	LAIPAENFPFCTIEPNEAR+Carbamidomethyl(11)	0.11	0.0432		
	NLADMPPDEAAK	0.25	0.0209		
	STLFNTLTK	0.14	0.1195		
Glyma17g20610.1	sucrose nonfermenting 1(SNF1) related protein kinase 2.3	2	0.12	0.0234	protein
	Peptide Sequnce	Ratio 4(2)F	Pvalue 4(2)F		
	IFVFDPAER	0.15	0.0711		
	LENTLLDGSPAPR	0.03	0.0496		
Glyma18g48620.1	Hyaluronan	6	0.12	0.0000	RNA
	Peptide Sequnce	Ratio 4(2)F	Pvalue 4(2)F		
	APYNQGPFEEGDAGK	0.07412282	0.000167216		
	EFESMQALSSK	0.137883393	0.015167556		
	EMTLEEYEK	0.138137627	0.014217909		

	GSSTNNAPAPSIEDPGHFPNLGAK	0.106197765	0.148515138		
	KAPAQQNKPAQLPTKPPPPAQAVR	0.062127675	0.098048428		
	NLGDEKPAVEEDVADGNK	0.115427836	0.004968965		
Glyma05g34120.2	Translation elongation factor EF1A	5	0.12	0.0050	signalling
	Peptide Sequnce	Ratio 4(2)F	Pvalue 4(2)F		
	EGDSLLVMPNK	0.25	0.0940		
	FSDFPQLGR	0.22	0.0469		
	MDEPTVQWSK	0.11	0.1820		
	STTGGQILFLSGQVDER	0.15	0.1004		
	VQVNNSICIEK+Carbamidomethyl(8)	0.08	0.0420		
Glyma04g01130.1	cold regulated 47	3	0.12	0.0011	stress
	Peptide Sequnce	Ratio 4(2)F	Pvalue 4(2)F		
	GVFDFLGK	0.07	0.0100		
	VEVVETAHAEEK	0.24	0.0427		
	YESSEVEVQDR	0.06	0.0359		
Glyma18g12660.1	rhamnose biosynthesis 1	10	0.12	0.0043	cell wall
	Peptide Sequnce	Ratio 4(2)F	Pvalue 4(2)F		
	AYSTDAFYFR	0.00	1.0000		
	EYENVCTLR+Carbamidomethyl(6)	0.13	0.0765		
	FLLLAMK	0.00	1.0000		
	FVENRPFNDQR	0.15	0.0046		
	GNNVYGPNQFPEK	0.85	0.7744		
	NPDWWGDVSGALLPHPR	0.03	0.0056		
	SQILFDFR	0.02	0.0225		
	SYGLPVITTR	0.09	0.0462		
	TMDWYVK	0.00	1.0000		
	YYDGSDNVTGTASNGDVNHSNQNR	0.00	1.0000		
Glyma03g07420.4	metallopeptidase M24 family protein	5	0.13	0.0013	protein
	Peptide Sequnce	Ratio 4(2)F	Pvalue 4(2)F		
	AWLALGTK	0.02	0.0775		
	ELDLTSPEVVTK	0.07	0.0598		
	FIFSEISQK	0.29	0.0284		
	IVEGVLSHQMK	0.10	0.0040		
	KGAEAEPMDATNDATPQEQD	0.00	1.0000		
Glyma05g07640.3	tRNA synthetase beta subunit family protein	4	0.13	0.0007	protein

	Peptide Sequnce	Ratio 4(2)F	Pvalue 4(2)F		
	FLHIIEDSPVFPVIYDSK	0.03	0.0188		
	FVIEPVEVISSDGK	0.14	0.0659		
	NVFIECTATDLTK+Carbamidomethyl(6)	0.13	0.0212		
	YNSFIDLQDK	0.18	0.0505		
Glyma09g37850.1	Hyaluronan	4	0.13	0.0002	RNA
	Peptide Sequnce	Ratio 4(2)F	Pvalue 4(2)F		
	EFESMQALSSK	0.14	0.0152		
	EMTLEEYEK	0.14	0.0142		
	GNWGAQTDELAQVTDEVANETEK	0.08	0.2194		
	NLGDEKPAVEEDVADGNK	0.12	0.0050		
Glyma08g23770.1	O fucosyltransferase family protein	5	0.13	0.0000	not assigned
	Peptide Sequnce	Ratio 4(2)F	Pvalue 4(2)F		
	SCFNAQEVAVFLR+Carbamidomethyl(2)	0.08	0.0001		
	SLGATLVIPDIR	0.09	0.0155		
	SQILVPANIPDSSASASSFLSHYVSK	0.06	0.0342		
	VTEEYIAQHVEPIYR	0.38	0.0651		
	WDESLDSLK	0.01	0.0387		
Glyma17g05340.2	histone deacetylase 2C	3	0.13	0.0001	RNA
	Peptide Sequnce	Ratio 4(2)F	Pvalue 4(2)F		
	FVLGTLSR	0.10	0.0143		
	IPQISLELVLEK	0.01	0.0468		
	VDPAEFEACIHLSQAALGEAK+Carbamidomethyl(9)	0.15	0.0016		
Glyma06g18800.1	Ribosomal protein L18e/L15 superfamily protein	2	0.13	0.0403	protein
	Peptide Sequnce	Ratio 4(2)F	Pvalue 4(2)F		
	APLIDVTQFGYFK	0.16	0.0425		
	GVLPQNQPVVVK	0.05	0.1583		
Glyma08g46090.2	rotamase FKBP 1	2	0.13	0.0065	protein
	Peptide Sequnce	Ratio 4(2)F	Pvalue 4(2)F		
	EGEGYERPNEGAIVK	0.15	0.0071		
	VLDLESTNVK	0.10	0.1140		
Glyma18g32830.1	rotamase FKBP 1	2	0.13	0.0065	protein
	Peptide Sequnce	Ratio 4(2)F	Pvalue 4(2)F		
	EGEGYERPNEGAIVK	0.15	0.0071		

	VLDLESTNVK	0.10	0.1140		
Glyma02g47460.1	ADP glucose pyrophosphorylase 1	3	0.13	0.0031	major CHO metabolism
	Peptide Sequnce	Ratio 4(2)F	Pvalue 4(2)F		
	LIDIPVSNCLNSNVSK+Carbamidomethyl(9)	0.09	0.0070		
	NVMLDLLR	0.16	0.1728		
	VDTTILGLDDER	0.17	0.0436		
Glyma14g01290.1	ADP glucose pyrophosphorylase 1	3	0.13	0.0031	major CHO metabolism
	Peptide Sequnce	Ratio 4(2)F	Pvalue 4(2)F		
	LIDIPVSNCLNSNVSK+Carbamidomethyl(9)	0.09	0.0070		
	NVMLDLLR	0.16	0.1728		
	VDTTILGLDDER	0.17	0.0436		
Glyma10g32471.1	S adenosyl L methionine dependent methyltransferases superfamily protein	4	0.14	0.0052	stress
	Peptide Sequnce	Ratio 4(2)F	Pvalue 4(2)F		
	DGLLLLELDR	0.08	0.0454		
	GIPAYLGVLGTK	0.08	0.0407		
	LADFGYSNDMFEK	0.37	0.1793		
	MWLTSESFR	0.03	0.0543		
Glyma09g16606.1	Ribosomal L22e protein family	2	0.14	0.0347	protein
	Peptide Sequnce	Ratio 4(2)F	Pvalue 4(2)F		
	GASFVIDCAKPVEDK+Carbamidomethyl(8)	0.07	0.1149		
	IMDIASLEK	0.17	0.0489		
Glyma02g02140.1	Ribosomal L22e protein family	5	0.14	0.0000	protein
	Peptide Sequnce	Ratio 4(2)F	Pvalue 4(2)F		
	AGALGDSITVTR	0.16	0.0181		
	GASFVIDCAKPVEDK+Carbamidomethyl(8)	0.07	0.1149		
	IMDIASLEK	0.17	0.0489		
	ITVTSDSNFSK	0.13	0.0052		
	YFNIAENEGEEED	0.13	0.0003		
Glyma10g02270.1	Ribosomal L22e protein family	5	0.14	0.0000	protein
	Peptide Sequnce	Ratio 4(2)F	Pvalue 4(2)F		
	AGALGDSITVTR	0.16	0.0181		
	GASFVIDCAKPVEDK+Carbamidomethyl(8)	0.07	0.1149		
	IMDIASLEK	0.17	0.0489		
	ITVTSDSNFSK	0.13	0.0052		
	YFNIAENEGEEED	0.13	0.0003		

Glyma02g04120.1	ATP citrate lyase A 1	4	0.14	0.0046	TCA / org transformation
	Peptide Sequnce	Ratio 4(2)F	Pvalue 4(2)F		
	ALGEEIGIPIEVYGPEATMTGICK+Carbamidomethyl(23)	0.01	0.0246		
	LVVKPDMLFGK	0.27	0.0432		
	SAQITASTDFTELQEK	0.17	0.1214		
	VMSATESFIHGLDEK	0.12	0.1200		
Glyma19g42090.2	Ribosomal L22e protein family	4	0.14	0.0000	protein
	Peptide Sequnce	Ratio 4(2)F	Pvalue 4(2)F		
	AGALGDSITVTR	0.162672796	0.01806001		
	IIVTSDSNFSK	0.127548583	0.013960888		
	IMDIASLEK	0.172167362	0.048852088		
	YFNIAENEGEEED	0.1290895	0.000303581		
Glyma01g29420.1	metallopeptidase M24 family protein	5	0.14	0.0001	protein
	Peptide Sequnce	Ratio 4(2)F	Pvalue 4(2)F		
	AWLALGTK	0.02	0.0775		
	ELDLTSPEVVTK	0.07	0.0598		
	FIFSEISQK	0.29	0.0284		
	IVEGVLSHQMK	0.10	0.0040		
	VVLSVSNPDTR	0.03	0.0590		
Glyma08g17010.3	ATP citrate lyase A 1	5	0.15	0.0080	TCA / org transformation
	Peptide Sequnce	Ratio 4(2)F	Pvalue 4(2)F		
	ALGEEIGIPIEVYGPEATMTGICK+Carbamidomethyl(23)	0.01	0.0246		
	EDEVLQYAR	0.31	0.0567		
	FTVLNPMGR	0.03	0.1091		
	LVVKPDMLFGK	0.27	0.0432		
	VVIDCATSNPDGQK+Carbamidomethyl(5)	0.00	1.0000		
Glyma09g06390.3	villin 4	3	0.15	0.0043	cell
	Peptide Sequnce	Ratio 4(2)F	Pvalue 4(2)F		
	DLDPAFQGAGQK	0.11	0.0301		
	FFTGDSYVILK	0.22	0.0633		
	FLEHDFLLEK	0.13	0.0408		
Glyma15g17640.2	villin 4	3	0.15	0.0043	cell
	Peptide Sequnce	Ratio 4(2)F	Pvalue 4(2)F		
	DLDPAFQGAGQK	0.11	0.0301		
	FFTGDSYVILK	0.22	0.0633		

	FLEHDFLLEK	0.13	0.0408		
Glyma02g40310.1	eukaryotic translation initiation factor 2 beta subunit	5	0.15	0.0001	protein
	Peptide Sequnce	Ratio 4(2)F	Pvalue 4(2)F		
	EEVPEIVPFDPTK	0.35	0.1057		
	ITIVDPADDPVEK	0.10	0.0010		
	NFEGILR	0.06	0.0500		
	TENLSVSEGVETAFAGLK	0.17	0.0317		
	TVFVNFMDLCK+Carbamidomethyl(10)	0.06	0.0961		
Glyma09g16553.1	Ribosomal L22e protein family	2	0.15	0.0023	protein
	Peptide Sequnce	Ratio 4(2)F	Pvalue 4(2)F		
	AGALGDSITVTR	0.16	0.0181		
	IIVTSDSNFSK	0.13	0.0140		
Glyma20g38080.1	Ribosomal L22e protein family	4	0.15	0.0000	protein
	Peptide Sequnce	Ratio 4(2)F	Pvalue 4(2)F		
	AGALADTITVTR	0.256007421	0.063561652		
	IIVTSDSNFSK	0.127548583	0.013960888		
	IMDIASLEK	0.172167362	0.048852088		
	YFNIAENEGEEED	0.1290895	0.000303581		
Glyma08g15960.3	beta glucosidase 31	6	0.16	0.0022	misc
	Peptide Sequnce	Ratio 4(2)F	Pvalue 4(2)F		
	FGLIYVDYK	0.12	0.0254		
	FLLQAIK	0.11	0.0541		
	FSAFWLQK	0.11	0.1183		
	FSISWSR	0.36	0.1739		
	GPSIWDTYTR	0.12	0.0421		
	GYANFCFK+Carbamidomethyl(6)	0.21	0.1785		
Glyma12g30600.1	histone deacetylase 2C	3	0.16	0.0000	RNA
	Peptide Sequnce	Ratio 4(2)F	Pvalue 4(2)F		
	FVLGTLSR	0.10	0.0143		
	SNEPVVLYLK	0.22	0.0020		
	VDPAEFEACIHLSQAALGEAK+Carbamidomethyl(9)	0.15	0.0016		
Glyma18g43040.1	SGNH hydrolase type esterase superfamily protein	2	0.16	0.0293	misc
	Peptide Sequnce	Ratio 4(2)F	Pvalue 4(2)F		
	AIDLWSAIK	0.13	0.1370		
	STINISNWAFPGNDKWE	0.19	0.0335		

Glyma16g19311.1	metallopeptidase M24 family protein	3	0.16	0.0025	protein
	Peptide Sequnce	Ratio 4(2)F	Pvalue 4(2)F		
	FIFSEISQK	0.293901063	0.02838005		
	IVEGVLSHQMK	0.102179377	0.003987128		
	VDEAEFEENEVYAIDIVTSTGDGKPK	0.057819962	0.356071521		
Glyma14g01600.1	Uncharacterised protein family (UPF0172)	2	0.16	0.0006	development
	Peptide Sequnce	Ratio 4(2)F	Pvalue 4(2)F		
	EPSANLVLLDYIASEK	0.36	0.0537		
	YFPEAAVLLLDNK	0.11	0.0010		
Glyma09g07120.1	Transducin family protein	2	0.17	0.0040	development
	Peptide Sequnce	Ratio 4(2)F	Pvalue 4(2)F		
	MSDLIYRPEDEVLAELEK	0.17	0.0335		
	VAAAEHISQFNEEAR	0.17	0.0138		
Glyma15g18450.1	Transducin family protein	2	0.17	0.0040	development
	Peptide Sequnce	Ratio 4(2)F	Pvalue 4(2)F		
	MSDLIYRPEDEVLAELEK	0.17	0.0335		
	VAAAEHISQFNEEAR	0.17	0.0138		
Glyma03g32130.1	S adenosyl L methionine dependent methyltransferases superfamily protein	2	0.17	0.0468	stress
	Peptide Sequnce	Ratio 4(2)F	Pvalue 4(2)F		
	DGLLLLELDR	0.08	0.0454		
	LDLSLMEHYER	0.27	0.1772		
Glyma19g34890.1	S adenosyl L methionine dependent methyltransferases superfamily protein	2	0.17	0.0468	stress
	Peptide Sequnce	Ratio 4(2)F	Pvalue 4(2)F		
	DGILLLELDR	0.08	0.0454		
	LDLSLMEHYER	0.27	0.1772		
Glyma14g38600.1	eukaryotic translation initiation factor 2 beta subunit	5	0.17	0.0000	protein
	Peptide Sequnce	Ratio 4(2)F	Pvalue 4(2)F		
	EEVPEIVPFDPTK	0.347580302	0.105673863		
	ITIVDPADEPVEK	0.084741422	0.000136064		
	NFEGILR	0.062385435	0.050027659		
	NYEYEELLGR	0.066171135	0.037558971		
	TVFVNFMDLCK+Carbamidomethyl(10)	0.062108264	0.096101083		
Glyma03g32800.1	regulatory particle AAA ATPase 2A	7	0.17	0.0018	protein
	Peptide Sequnce	Ratio 4(2)F	Pvalue 4(2)F		

	AICTEAGLLALR+Carbamidomethyl(3)	0.02	0.0460		
	APLESYADIGGLDAQIQEIK	0.11	0.0049		
	AVANSTSATFLR	0.18	0.0621		
	DYLLMEEEFVANQER	0.10	0.2169		
	GVILYGEPGTGK	0.57	0.2860		
	MTLADDVNLEEFVMTK	0.50	0.4968		
	VVGSELIQK	0.02	0.0771		
Glyma10g04920.1	regulatory particle AAA ATPase 2A	7	0.17	0.0018	protein
	Peptide Sequnce	Ratio 4(2)F	Pvalue 4(2)F		
	AICTEAGLLALR+Carbamidomethyl(3)	0.02	0.0460		
	APLESYADIGGLDAQIQEIK	0.11	0.0049		
	AVANSTSATFLR	0.18	0.0621		
	DYLLMEEEFVANQER	0.10	0.2169		
	GVILYGEPGTGK	0.57	0.2860		
	MTLADDVNLEEFVMTK	0.50	0.4968		
	VVGSELIQK	0.02	0.0771		
Glyma13g19280.1	regulatory particle AAA ATPase 2A	7	0.17	0.0018	protein
	Peptide Sequnce	Ratio 4(2)F	Pvalue 4(2)F		
	AICTEAGLLALR+Carbamidomethyl(3)	0.02	0.0460		
	APLESYADIGGLDAQIQEIK	0.11	0.0049		
	AVANSTSATFLR	0.18	0.0621		
	DYLLMEEEFVANQER	0.10	0.2169		
	GVILYGEPGTGK	0.57	0.2860		
	MTLADDVNLEEFVMTK	0.50	0.4968		
	VVGSELIQK	0.02	0.0771		
Glyma19g35510.1	regulatory particle AAA ATPase 2A	7	0.17	0.0018	protein
	Peptide Sequnce	Ratio 4(2)F	Pvalue 4(2)F		
	AICTEAGLLALR+Carbamidomethyl(3)	0.02	0.0460		
	APLESYADIGGLDAQIQEIK	0.11	0.0049		
	AVANSTSATFLR	0.18	0.0621		
	DYLLMEEEFVANQER	0.10	0.2169		
	GVILYGEPGTGK	0.57	0.2860		
	MTLADDVNLEEFVMTK	0.50	0.4968		
	VVGSELIQK	0.02	0.0771		
Glyma03g29440.1	seed imbibition 2	2	0.17	0.0276	minor CHO metabolism

	Peptide Sequnce	Ratio 4(2)F	Pvalue 4(2)F		
	FLIIDDGWQQIENK	0.72	0.4994		
	VDVQNIIETLGAGHGGR	0.09	0.0086		
Glyma03g01550.1	Remorin family protein	2	0.17	0.0222	RNA
	Peptide Sequnce	Ratio 4(2)F	Pvalue 4(2)F		
	DIALAEIEK	0.27	0.2430		
	IAALEAQLR	0.14	0.0136		
Glyma05g20930.1	Granulin repeat cysteine protease family protein	2	0.17	0.0025	protein
	Peptide Sequnce	Ratio 4(2)F	Pvalue 4(2)F		
	FVSLSEQELVDCDR+Carbamidomethyl(12)	0.18	0.0060		
	NSWGTGWGEDGYFK	0.15	0.0458		
Glyma16g16290.1	Granulin repeat cysteine protease family protein	2	0.17	0.0025	protein
	Peptide Sequnce	Ratio 4(2)F	Pvalue 4(2)F		
	FVSLSEQELVDCDR+Carbamidomethyl(12)	0.184814431	0.00602178		
	NSWGTGWGEDGYFK	0.145416936	0.045781431		
Glyma17g18440.1	Granulin repeat cysteine protease family protein	2	0.17	0.0025	protein
	Peptide Sequnce	Ratio 4(2)F	Pvalue 4(2)F		
	FVSLSEQELVDCDR+Carbamidomethyl(12)	0.184814431	0.00602178		
	NSWGTGWGEDGYFK	0.145416936	0.045781431		
Glyma03g40300.1	Trimeric LpxA like enzyme	2	0.17	0.0016	protein
	Peptide Sequnce	Ratio 4(2)F	Pvalue 4(2)F		
	GLLLDDIMGYK	0.07	0.0101		
	SGAVLEPGVILSFK	0.22	0.0165		
Glyma08g46270.1	UDP glycosyltransferase 73B4	2	0.18	0.0269	misc
	Peptide Sequnce	Ratio 4(2)F	Pvalue 4(2)F		
	FLWVLPK	0.16	0.1085		
	GWVPQGLILK	0.19	0.0383		
Glyma02g00550.1	S adenosyl L methionine dependent methyltransferases superfamily protein	5	0.18	0.0072	stress
	Peptide Sequnce	Ratio 4(2)F	Pvalue 4(2)F		
	DGILLLELDR	0.08	0.0454		
	GIPAYLGVLGTK	0.08	0.0407		
	LDLSLMEHYER	0.27	0.1772		
	SDQNWMIVK	0.37	0.2231		
	VENYWNLLGPK	0.12	0.0779		

Glyma10g29200.1	Ribosomal L22e protein family	4	0.18	0.0000	protein
	Peptide Sequnce	Ratio 4(2)F	Pvalue 4(2)F		
	AGALGDSITVVR	0.33	0.1482		
	IIVTSDSNFSK	0.13	0.0140		
	IMDIASLEK	0.17	0.0489		
	YFNIAENEGEEED	0.13	0.0003		
Glyma04g03020.1	Papain family cysteine protease	2	0.18	0.0130	protein
	Peptide Sequnce	Ratio 4(2)F	Pvalue 4(2)F		
	APILPTSDLPTDFDWR	0.22	0.0359		
	NSWGESWGEEGYYK	0.10	0.0495		
Glyma10g06480.1	ATPase AAA type CDC48 protein	20	0.18	0.0000	cell
	Peptide Sequnce	Ratio 4(2)F	Pvalue 4(2)F		
	AIANECQANFISVK+Carbamidomethyl(6)	0.03	0.0603		
	DFSTAILER	0.07	0.0870		
	DTHGYVGADLAALCTEAALQCIR+Carbamidomethyl(14)Carbamidomethyl(21)	0.02	0.0541		
	DTICIALADETCEEPK+Carbamidomethyl(4)Carbamidomethyl(12)	0.16	0.1129		
	EDENRLDEVGYDDVGGVR	0.03	0.0118		
	EIDIGVPDEVGR	0.06	0.0576		
	ELQETVQYPVEHPEK	0.07	0.2833		
	ELVELPLR	0.02	0.0982		
	ETVVEVPNVSWEDIGGLENVK	0.02	0.0002		
	GILLYGPPGSGK	0.07	0.0858		
	GVLFYGPPGCGK+Carbamidomethyl(10)	0.04	0.0658		
	LAEDVDLER	0.03	0.0887		
	LAGESESNLR	2.10	0.1645		
	LDEVGYDDVGGVR	0.33	0.0383		
	LDQLIYIPLPDEDSR	0.06	0.0284		
	LVVDEAVNDDNSVVALHPNTMEK	0.00	1.0000		
	TVFIIGATNRPDIIDPALLRPGR	0.01	0.3223		
	VLNQLLTEMDGMSAK	0.08	0.1267		
	YQAFAQTLQQSR	0.02	0.0478		
	YTQGFSGADITEICQR+Carbamidomethyl(14)	0.01	0.0314		
Glyma13g20680.1	ATPase AAA type CDC48 protein	20	0.18	0.0000	cell
	Peptide Sequnce	Ratio 4(2)F	Pvalue 4(2)F		
	AIANECQANFISVK+Carbamidomethyl(6)	0.03	0.0603		

	DFSTAILER	0.07	0.0870		
	DTHGYVGADLAALCTEAALQCIR+Carbamidomethyl(14)Carbamidomethyl(21)	0.02	0.0541		
	DTICIALADETCEEPK+Carbamidomethyl(4)Carbamidomethyl(12)	0.16	0.1129		
	EDENRLDEVGYDDVGGVR	0.03	0.0118		
	EIDIGVPDEVGR	0.06	0.0576		
	ELQETVQYPVEHPEK	0.07	0.2833		
	ELVELPLR	0.02	0.0982		
	ETVVEVPNVSWEDIGGLENVK	0.02	0.0002		
	GILLYGPPGSGK	0.07	0.0858		
	GVLFYGPPGCGK+Carbamidomethyl(10)	0.04	0.0658		
	LAEDVDLER	0.03	0.0887		
	LAGESESNLR	2.10	0.1645		
	LDEVGYDDVGGVR	0.33	0.0383		
	LDQLIYIPLPDEDSR	0.06	0.0284		
	LVVDEAVNDDNSVVVLHPDTMEK	0.00	1.0000		
	TVFIIGATNRPDIIDPALLRPGR	0.01	0.3223		
	VLNQLLTEMDGMSAK	0.08	0.1267		
	YQAFAQTLQQSR	0.02	0.0478		
	YTQGFSGADITEICQR+Carbamidomethyl(14)	0.01	0.0314		
Glyma19g27250.1	translocon associated protein beta (TRAPB) family protein	3	0.19	0.0000	not assigned
	Peptide Sequnce	Ratio 4(2)F	Pvalue 4(2)F		
	AALQEAYSTPILPLDVLSDRPPEK	0.15	0.0094		
	GVFAGEPAVIK	0.21	0.0390		
	LDAGGIISHTFELEAK	0.22	0.0010		
Glyma10g41880.4	Cysteinyl tRNA synthetase class Ia family protein	2	0.19	0.0280	protein
	Peptide Sequnce	Ratio 4(2)F	Pvalue 4(2)F		
	DAVSPLLQGETEK	0.27	0.1003		
	SLGNFFTIR	0.13	0.0433		
Glyma20g25160.3	Cysteinyl tRNA synthetase class Ia family protein	2	0.19	0.0280	protein
	Peptide Sequnce	Ratio 4(2)F	Pvalue 4(2)F		
	DAVSPLLQGETEK	0.27	0.1003		
	SLGNFFTIR	0.13	0.0433		
Glyma03g04880.1	Peroxidase superfamily protein	2	0.19	0.0048	misc
	Peptide Sequnce	Ratio 4(2)F	Pvalue 4(2)F		
	GFEVIDNIK	0.54	0.1263		

	YYATYPSLFFK	0.11	0.0045		
Glyma09g02160.1	glycosyl hydrolase 9A1	5	0.19	0.0003	cell wall
	Peptide Sequnce	Ratio 4(2)F	Pvalue 4(2)F		
	DLVGGYYDAGDAIK	0.06	0.0072		
	LFLSPGYPYEEILR	0.02	0.0144		
	SQIDYILGNNPR	0.93	0.9180		
	WGTDYFLK	0.04	0.0107		
	YVDLGCIIVSR+Carbamidomethyl(6)	0.21	0.0824		
Glyma13g05520.4	RNA binding KH domain containing protein	2	0.19	0.0018	RNA
	Peptide Sequnce	Ratio 4(2)F	Pvalue 4(2)F		
	LLVPSDQIGCVIGK+Carbamidomethyl(10)	0.24	0.0128		
	LVCPTGNIGGVIGK+Carbamidomethyl(3)	0.13	0.0147		
Glyma19g02840.2	RNA binding KH domain containing protein	2	0.19	0.0018	RNA
	Peptide Sequnce	Ratio 4(2)F	Pvalue 4(2)F		
	LLVPSDQIGCVIGK+Carbamidomethyl(10)	0.24	0.0128		
	LVCPTGNIGGVIGK+Carbamidomethyl(3)	0.13	0.0147		
Glyma08g20190.1	lipoxygenase 1	6	0.19	0.0303	hormone metabolism
	Peptide Sequnce	Ratio 4(2)F	Pvalue 4(2)F		
	EMIAGVNPCVIR+Carbamidomethyl(9)	0.35	0.1558		
	GIPNSISI	0.11	0.0765		
	LLFPHYR	0.29	0.1725		
	LPTDILSK	0.07	0.1551		
	SAWMTDEEFAR	0.05	0.1749		
	SSDFLTYGLK	0.39	0.2106		
Glyma08g20210.1	lipoxygenase 1	4	0.19	0.041	hormone metabolism
	Peptide Sequnce	Ratio 4(2)F	Pvalue 4(2)F		
	EMIAGVNPCVIR+Carbamidomethyl(9)	0.35	0.1558		
	GIPNSISI	0.11	0.0765		
	LLFPHYR	0.29	0.1725		
	LPTDILSK	0.07	0.1551		
Glyma11g10860.1	Alanyl tRNA synthetase	5	0.2	0.0106	protein
	Peptide Sequnce	Ratio 4(2)F	Pvalue 4(2)F		
	AFALLSEEGIAK	0.03	0.0867		
	IYATYFGGDDK	0.08	0.0994		
	LVGTLLEEK	0.27	0.0570		

	NIIEEEEESFGR	0.02	0.0638		
	SGLAPDLEAR	0.56	0.3189		
Glyma07g06410.1	adenine nucleotide transporter 1	2	0.2	0.0113	transport
	Peptide Sequnce	Ratio 4(2)F	Pvalue 4(2)F		
	ITVQTEASPYQYR	0.05	0.1175		
	SLVAGGVAGGVSR	0.21	0.0128		
Glyma16g03020.1	adenine nucleotide transporter 1	2	0.2	0.0113	transport
	Peptide Sequnce	Ratio 4(2)F	Pvalue 4(2)F		
	ITVQTEASPYQYR	0.05	0.1175		
	SLVAGGVAGGVSR	0.21	0.0128		
Glyma19g03590.1	Transducin/WD40 repeat like superfamily protein	3	0.2	0.027	not assigned
	Peptide Sequnce	Ratio 4(2)F	Pvalue 4(2)F		
	FFLTGCYDGLGR+Carbamidomethyl(6)	0.18	0.0931		
	LNAEGPVNNPMR	0.54	0.2589		
	MSLEQFLLAK	0.05	0.0334		
Glyma19g30110.1	N.D.*	2	0.2	0.0196	not assigned
	Peptide Sequnce	Ratio 4(2)F	Pvalue 4(2)F		
	LDEVAISQLR	0.22	0.0560		
	WITEEVLCQPK+Carbamidomethyl(8)	0.11	0.0511		
Glyma04g08540.1	La protein 1	2	0.2	0.0012	RNA
	Peptide Sequnce	Ratio 4(2)F	Pvalue 4(2)F		
	NYIAILDPVTGEAEK	0.12	0.0645		
	TLAVSPFEYDLK	0.23	0.0018		
Glyma10g31590.1	methionine gamma lyase	6	0.2	0.0004	amino acid metabolism
	Peptide Sequnce	Ratio 4(2)F	Pvalue 4(2)F		
	ELAGISPGLVR	0.19	0.0223		
	FISGGADIIAGAVCGPAR+Carbamidomethyl(14)	0.14	0.0682		
	LGADVVVHSISK	0.01	0.0374		
	LLLQQGEADPAAALASAR	0.05	0.0349		
	MFAGELGPDR	0.29	0.1789		
	MFAGELGPDRDFFIYSR	0.28	0.0603		
Glyma04g16660.1	Ribosomal protein S19e family protein	3	0.2	0.0113	protein
	Peptide Sequnce	Ratio 4(2)F	Pvalue 4(2)F		
	ELAPYDPDWYYVR	0.17	0.0421		

	GGLGVGAFQR	0.19	0.0548		
	MELPEWTDIVK	0.24	0.1136		
Glyma11g25910.1	Ribosomal protein S19e family protein	3	0.2	0.0113	protein
	Peptide Sequnce	Ratio 4(2)F	Pvalue 4(2)F		
	ELAPYDPDWYYVR	0.17	0.0421		
	GGLGVGAFQR	0.19	0.0548		
	MELPEWTDIVK	0.24	0.1136		
Glyma13g06070.1	Ribosomal protein S19e family protein	3	0.2	0.0113	protein
	Peptide Sequnce	Ratio 4(2)F	Pvalue 4(2)F		
	ELAPYDPDWYYVR	0.17	0.0421		
	GGLGVGAFQR	0.19	0.0548		
	MELPEWTDIVK	0.24	0.1136		
Glyma09g37070.3	RNA binding KH domain containing protein	3	0.2	0.0012	RNA
	Peptide Sequnce	Ratio 4(2)F	Pvalue 4(2)F		
	ALFQIAAQIR	0.24	0.0341		
	IGDALPGCDER+Carbamidomethyl(8)	0.09	0.0372		
	LLVPSDQIGCVIGK+Carbamidomethyl(10)	0.24	0.0128		
Glyma18g49600.2	RNA binding KH domain containing protein	3	0.2	0.0012	RNA
	Peptide Sequnce	Ratio 4(2)F	Pvalue 4(2)F		
	ALFQIAAQIR	0.24	0.0341		
	IGDALPGCDER+Carbamidomethyl(8)	0.09	0.0372		
	LLVPSDQIGCVIGK+Carbamidomethyl(10)	0.24	0.0128		
Glyma08g15420.1	cinnamyl alcohol dehydrogenase 9	3	0.21	0.0013	secondary metabolism
	Peptide Sequnce	Ratio 4(2)F	Pvalue 4(2)F		
	AFGWAASDTSGTLAPFHFSR	0.17	0.0035		
	LGADFFLVSSDPAK	0.16	0.0287		
	LIGGSNFGGLK	0.37	0.1847		
Glyma13g17200.3	oligouridylate binding protein 1B	3	0.21	0.0033	RNA
	Peptide Sequnce	Ratio 4(2)F	Pvalue 4(2)F		
	GFGFVSFR	0.28	0.0069		
	NQQDAQSAINDLTGK	0.32	0.4130		
	SSYGFVDYFDR	0.11	0.0195		
Glyma17g05530.3	oligouridylate binding protein 1B	3	0.21	0.0033	RNA
	Peptide Sequnce	Ratio 4(2)F	Pvalue 4(2)F		

	GFGFVSFR	0.28	0.0069		
	NQQDAQSAINDLTGK	0.32	0.4130		
	SSYGFVDYFDR	0.11	0.0195		
Glyma15g01120.2	phospholipase D alpha 1	2	0.21	0.0337	lipid metabolism
	Peptide Sequnce	Ratio 4(2)F	Pvalue 4(2)F		
	LYATIDLEK	0.21	0.1080		
	VSLYQDAHVPDNFVPK	0.22	0.0501		
Glyma05g03100.1	malate synthase	3	0.21	0.0263	gluconeogenesis / glyoxylate cycle
	Peptide Sequnce	Ratio 4(2)F	Pvalue 4(2)F		
	IWNSVFEK	0.07	0.0543		
	MVINALNSAAK	0.24	0.1701		
	TQGAGFGPFFYLPK	0.27	0.0844		
Glyma17g13740.1	malate synthase	3	0.21	0.0263	gluconeogenesis / glyoxylate cycle
	Peptide Sequnce	Ratio 4(2)F	Pvalue 4(2)F		
	IWNSVFEK	0.07	0.0543		
	MVINALNSAAK	0.24	0.1701		
	TQGAGFGPFFYLPK	0.27	0.0844		
Glyma19g36740.1	ATPase AAA type CDC48 protein	21	0.21	0	cell
	Peptide Sequnce	Ratio 4(2)F	Pvalue 4(2)F		
	AIANECQANFISVK+Carbamidomethyl(6)	0.03	0.0603		
	DFSTAILER	0.07	0.0870		
	DNPEAMEEDDVEDEIAEIK	0.08	0.0796		
	DTHGYVGADLAALCTEAALQCIR+Carbamidomethyl(14)Carbamidomethyl(21)	0.02	0.0541		
	EDENRLDEVGYDDVGGVR	0.03	0.0118		
	EIDIGVPDEVGR	0.06	0.0576		
	ELQETVQYPVEHPEK	0.07	0.2833		
	ELVELPLR	0.02	0.0982		
	ETVVEVPNVSWEDIGGLENVK	0.02	0.0002		
	GILLYGPPGSGK	0.07	0.0858		
	GVLFYGPPGCGK+Carbamidomethyl(10)	0.04	0.0658		
	LAEDVDLER	0.03	0.0887		
	LAGESESNLR	2.10	0.1645		
	LDEVGYDDVGGVR	0.33	0.0383		
	LDQLIYIPLPDEDSR	0.06	0.0284		
	LGDVVSVHQCPDVK+Carbamidomethyl(10)	0.55	0.1658		

	LVVDEAVNDDNSVVALHPDTMEK	0.02	0.3980		
	TVFIIGATNRPDIIDPALLRPGR	0.01	0.3223		
	VLNQLLTEMDGMSAK	0.08	0.1267		
	YQAFAQTLQQSR	0.02	0.0478		
	YTQGFSGADITEICQR+Carbamidomethyl(14)	0.01	0.0314		
Glyma06g02290.1	Pyridoxal dependent decarboxylase family protein	11	0.22	0.0013	amino acid metabolism
	Peptide Sequnce	Ratio 4(2)F	Pvalue 4(2)F		
	AEIESVLSLGVSPDR	0.06	0.0187		
	IIYANPCK+Carbamidomethyl(7)	0.12	0.2086		
	LPTVQPFYAVK	0.23	0.1165		
	NCHPTCELLLR+Carbamidomethyl(2)Carbamidomethyl(6)	0.00	1.0000		
	NVFEMASGLGLPR	0.11	0.0205		
	PSLVAEAFEAK	0.14	0.0944		
	STFLAYSSPEHSMF	0.02	0.0547		
	TYPSTVFGPTCDSIDTVLR+Carbamidomethyl(11)	0.56	0.8247		
	VLDIGGGFTSGPPFEAAALK	0.01	0.0233		
	VTGVSFHIGSGGADTR	0.01	0.8145		
	YFAETAFTLATR	0.06	0.0689		
Glyma04g34300.1	N.D. *	2	0.22	0.0397	not assigned
	Peptide Sequnce	Ratio 4(2)F	Pvalue 4(2)F		
	LIIIANNCPPLR+Carbamidomethyl(8)	0.19	0.0418		
	SEIEYYAMLAK	0.31	0.1575		
Glyma19g38600.1	NDR1/HIN1 like 1	2	0.22	0.0034	stress
	Peptide Sequnce	Ratio 4(2)F	Pvalue 4(2)F		
	IGIYYDR	0.26	0.0254		
	LNTYVTYR	0.17	0.0152		
Glyma07g03920.2	lipoxygenase 1	2	0.22	0.0497	hormone metabolism
	Peptide Sequnce	Ratio 4(2)F	Pvalue 4(2)F		
	GIPNSISI	0.11	0.0765		
	SSDFLTYAIK	0.35	0.1128		
Glyma03g33990.1	ATPase AAA type CDC48 protein	21	0.22	0	cell
	Peptide Sequnce	Ratio 4(2)F	Pvalue 4(2)F		
	AIANECQANFISVK+Carbamidomethyl(6)	0.03	0.0603		
	DFSTAILER	0.07	0.0870		

	DNPEAMEEDDVEDEIAEIK	0.08	0.0796		
	DTHGYVGADLAALCTEAALQCIR+Carbamidomethyl(14)Carbamidomethyl(21)	0.02	0.0541		
	DTICIALADETCEEPK+Carbamidomethyl(4)Carbamidomethyl(12)	0.16	0.1129		
	EDENRLDEVGYDDVGGVR	0.03	0.0118		
	EIDIGVPDEVGR	0.06	0.0576		
	ELQETVQYPVEHPEK	0.07	0.2833		
	ELVELPLR	0.02	0.0982		
	ETVVEVPNVSWEDIGGLENVK	0.02	0.0002		
	GILLYGPPGSGK	0.07	0.0858		
	GVLFYGPPGCGK+Carbamidomethyl(10)	0.04	0.0658		
	LAGESESNLR	2.10	0.1645		
	LDEVGYDDVGGVR	0.33	0.0383		
	LDQLIYIPLPDEDSR	0.06	0.0284		
	LGDVVSVHQCPDVK+Carbamidomethyl(10)	0.55	0.1658		
	LVVDEAVNDDNSVVALHPDTMEK	0.02	0.3980		
	TVFIIGATNRPDIIDPALLRPGR	0.01	0.3223		
	VLNQLLTEMDGMSAK	0.08	0.1267		
	YQAFAQTLQQSR	0.02	0.0478		
	YTQGFSGADITEICQR+Carbamidomethyl(14)	0.01	0.0314		
Glyma08g08970.1	urease accessory protein G	5	0.22	0	amino acid metabolism
	Peptide Sequnce	Ratio 4(2)F	Pvalue 4(2)F		
	ADILLCESGGDNLAANFSR+Carbamidomethyl(6)	0.10	0.0011		
	AFTVGIGGPVGTGK	0.08	0.0265		
	ENYSLAAVTNDIFTK	0.14	0.0070		
	GGPGITQADLLVINK	0.26	0.0044		
	TDLAPAIGADLAVMQR	0.45	0.2601		
Glyma11g09630.1	RNAse l inhibitor protein 2	3	0.23	0.0437	RNA
	Peptide Sequnce	Ratio 4(2)F	Pvalue 4(2)F		
	AVQGNVGQVLDQK	0.26	0.0967		
	GSELQNYFTR	0.06	0.2447		
	NVGDLSGGELQR	0.09	0.0649		
Glyma16g03760.1	UDP glycosyltransferase 73B4	3	0.23	0.0126	misc
	Peptide Sequnce	Ratio 4(2)F	Pvalue 4(2)F		
	GQHVTIITTPANAQLFDQNIDK	0.09	0.0362		
	HDCLTWLDSK+Carbamidomethyl(3)	0.52	0.2679		

	VWHVGPSSLMVQK	0.06	0.0308		
Glyma16g05530.1	translocon associated protein beta (TRAPB) family protein	3	0.23	0.0001	not assigned
	Peptide Sequnce	Ratio 4(2)F	Pvalue 4(2)F		
	GVFAGEPAVIK	0.21	0.0390		
	LDAGGILSHTFELEAK	0.22	0.0010		
	SALQEAYSTPILPLDVLSDRPPEK	0.26	0.0253		
Glyma04g01690.1	Ribophorin I	4	0.23	0.0016	protein
	Peptide Sequnce	Ratio 4(2)F	Pvalue 4(2)F		
	DISVSVPFPVK	0.18	0.0246		
	IDLTSQIVR	0.26	0.0454		
	TAFTIGYGLPLR	0.20	0.0331		
	YPMFGGWK	0.28	0.1084		
Glyma17g01970.1	Aluminium induced protein with YGL and LRDR motifs	3	0.23	0.0169	metal handling
	Peptide Sequnce	Ratio 4(2)F	Pvalue 4(2)F		
	ITAVPANEEEIWGATFK	0.26	0.0171		
	STNEVLLVIEAYK	0.10	0.0851		
	STSTLFVASDQYGK	0.25	0.3005		
Glyma06g14360.1	Ribosomal protein L6 family	6	0.23	0.0008	protein
	Peptide Sequnce	Ratio 4(2)F	Pvalue 4(2)F		
	FLDGIYVSEK	0.18	0.0376		
	FVYAHFPINASIGNNSK	0.08	0.0474		
	IDSWFGSR	0.14	0.0508		
	TALSHVENLITGVTK	0.28	0.1513		
	TILSSETMNIPDGVSIK	0.10	0.0490		
	VKDELVLDGNDIELVSR	0.10	0.0793		
Glyma04g16340.1	dynamin related protein 3A	4	0.24	0.0048	misc
	Peptide Sequnce	Ratio 4(2)F	Pvalue 4(2)F		
	LGYVGVVNR	0.17	0.1078		
	SLEEVDPCEDLTDDDIR+Carbamidomethyl(8)	0.10	0.0044		
	SSVLEALVGR	0.23	0.2424		
	VPVGDQPSDIEAR	0.34	0.1383		
Glyma07g18500.1	metallopeptidase M24 family protein	7	0.24	0.0019	protein
	Peptide Sequnce	Ratio 4(2)F	Pvalue 4(2)F		
	AWLALGTK	0.02	0.0775		
	ELDLSSAEVVTK	0.60	0.1667		

	FIFSEISQK	0.29	0.0284		
	ITTHPLQELQPTK	0.00	1.0000		
	IVEGVLSHQMK	0.10	0.0040		
	VDEAEFEENEVYAIDIVTSTGDGKPK	0.06	0.3561		
	VVLSLSNPDTR	0.05	0.0705		
Glyma18g43390.1	metallopeptidase M24 family protein	7	0.24	0.0019	protein
	Peptide Sequnce	Ratio 4(2)F	Pvalue 4(2)F		
	AWLALGTK	0.02	0.0775		
	ELDLSSAEVVTK	0.60	0.1667		
	FIFSEISQK	0.29	0.0284		
	ITTHPLQELQPTK	0.00	1.0000		
	IVEGVLSHQMK	0.10	0.0040		
	VDEAEFEENEVYAIDIVTSTGDGKPK	0.06	0.3561		
	VVLSLSNPDTR	0.05	0.0705		
Glyma13g39830.1	ATPase AAA type CDC48 protein	16	0.24	0.0000	cell
	Peptide Sequnce	Ratio 4(2)F	Pvalue 4(2)F		
	AIANECQANFISVK+Carbamidomethyl(6)	0.03	0.0603		
	DFSTAILER	0.07	0.0870		
	DTHGYVGADLAALCTEAALQCIR+Carbamidomethyl(14)Carbamidomethyl(21)	0.02	0.0541		
	EIDIGVPDEVGR	0.06	0.0576		
	ELQETVQYPVEHPEK	0.07	0.2833		
	ELVELPLR	0.02	0.0982		
	ETVVEVPNVSWEDIGGLENVK	0.02	0.0002		
	GILLYGPPGSGK	0.07	0.0858		
	GVLFYGPPGCGK+Carbamidomethyl(10)	0.04	0.0658		
	LAGESESNLR	2.10	0.1645		
	LDEVGYDDVGGVR	0.33	0.0383		
	LDQLIYIPLPDEDSR	0.06	0.0284		
	LGDVVSVHQCPDVK+Carbamidomethyl(10)	0.55	0.1658		
	TVFIIGATNRPDIIDPALLRPGR	0.01	0.3223		
	VLNQLLTEMDGMSAK	0.08	0.1267		
	YQAFAQTLQQSR	0.02	0.0478		
Glyma06g01790.1	Ribophorin I	3	0.24	0.0061	protein
	Peptide Sequnce	Ratio 4(2)F	Pvalue 4(2)F		
	DISVSVPFPVK	0.18	0.0246		

	IDLTSQIVR	0.26	0.0454		
	YPMFGGWK	0.28	0.1084		
Glyma15g12780.1	farnesyl diphosphate synthase 1	4	0.24	0.0472	secondary metabolism
	Peptide Sequnce	Ratio 4(2)F	Pvalue 4(2)F		
	GLSVIDSYR	0.14	0.2869		
	MLDYNVPGGK	0.26	0.2208		
	STFLNVYSVLK	0.33	0.0296		
	VGMIAANDGVLLR	0.06	0.2100		
Glyma04g02230.1	Pyridoxal dependent decarboxylase family protein	10	0.24	0.0035	amino acid metabolism
	Peptide Sequnce	Ratio 4(2)F	Pvalue 4(2)F		
	AEIESVLSLGVSPDR	0.06	0.0187		
	IIYANPCK+Carbamidomethyl(7)	0.12	0.2086		
	INAAIEGSFGK	0.09	0.0373		
	LPTVQPFYAVK	0.23	0.1165		
	MGVLDIGGGFTSGPSFEAAALK	0.28	0.2949		
	NGLTDFIQR	0.15	0.0049		
	SIFLACSSPEHTMF+Carbamidomethyl(6)	0.00	1.0000		
	TYPSTVFGPTCDSIDTVLR+Carbamidomethyl(11)	0.56	0.8247		
	VTGVSFHIGSGGADTR	0.01	0.8145		
	YFAETAFTLATR	0.06	0.0689		
Glyma14g35370.2	Class II DAHP synthetase family protein	5	0.24	0.0000	amino acid metabolism
	Peptide Sequnce	Ratio 4(2)F	Pvalue 4(2)F		
	AFATGGYAAMQR	0.07	0.0763		
	GVANPLGIK	0.34	0.0051		
	TRPFDFIR	0.34	0.0337		
	TVTFDDLSSR	0.32	0.0553		
	VTQWNLDFTDHSEQGDR	0.08	0.0063		
Glyma02g09650.1	methionine gamma lyase	3	0.25	0.0159	amino acid metabolism
	Peptide Sequnce	Ratio 4(2)F	Pvalue 4(2)F		
	LGADVVVHSISK	0.01	0.0374		
	MFAGELGPDR	0.29	0.1789		
	MFAGELGPDRDFFIYSR	0.28	0.0603		
Glyma02g01170.1	S adenosylmethionine synthetase 2	4	0.25	0.0000	amino acid metabolism
	Peptide Sequnce	Ratio 4(2)F	Pvalue 4(2)F		
	EILQLVK	0.11	0.0239		

	FVIGGPHGDAGLTGR	0.28	0.0001		
	TIFHLNPSGR	0.30	0.0106		
	VLVNIEQQSPDIAQGVHGHFTK	0.16	0.0000		
Glyma15g42570.1	beta glucosidase 17	2	0.25	0.0284	misc
	Peptide Sequnce	Ratio 4(2)F	Pvalue 4(2)F		
	FGIIYVDYK	0.12	0.0254		
	FSISWSR	0.36	0.1739		
Glyma15g42590.1	beta glucosidase 17	2	0.25	0.0284	misc
	Peptide Sequnce	Ratio 4(2)F	Pvalue 4(2)F		
	FGIIYVDYK	0.12	0.0254		
	FSISWSR	0.36	0.1739		
Glyma04g10100.1	L Aspartase like family protein	2	0.25	0.0283	amino acid metabolism
	Peptide Sequnce	Ratio 4(2)F	Pvalue 4(2)F		
	FMTSEALGFTAPLR	0.31	0.0576		
	NCQLLDLSLDELR+Carbamidomethyl(2)	0.13	0.0764		
Glyma06g10130.1	L Aspartase like family protein	2	0.25	0.0283	amino acid metabolism
	Peptide Sequnce	Ratio 4(2)F	Pvalue 4(2)F		
	FMTSEALGFTAPLR	0.31	0.0576		
	NCQLLDLSLDELR+Carbamidomethyl(2)	0.13	0.0764		
Glyma15g03040.1	lipoxygenase 1	28	0.25	0.0009	hormone metabolism
	Peptide Sequnce	Ratio 4(2)F	Pvalue 4(2)F		
	DEAFGHLK	5.96	0.4941		
	DGGDYWTSDAGPLEAFK	0.05	0.1130		
	DGGDYWTSDAGPLEAFKR	0.24	0.0743		
	DTMNINSLAR	0.05	0.2120		
	DWVFTDQALPNDLVK	0.04	0.1444		
	ETIAGVNPNVIK	0.01	0.1459		
	FGIIGGNK	0.00	1.0000		
	GIPNSISI	0.11	0.0765		
	IAPIPVVK	0.00	1.0000		
	IFFANNTYLPSETPAPLVK	0.42	0.3124		
	ILEEFPPR	0.01	0.2002		
	IYDYDVYNDLGDPDK	0.03	0.1003		
	LDSQAYGDHTSIITK	0.13	0.1815		
	LFILDHHDYLIPYLR	0.01	0.4093		

	LLFPHYR	0.29	0.1725		
	LYEGGVTLPTNFLSK	0.06	0.1284		
	MPYTLLYPSSEEGLTFR	0.06	0.1356		
	MPYTLLYPSSEEGLTFR+Oxidation(1)	2.04	0.3557		
	NFMQVEFYLK	0.24	0.1464		
	NFMQVEFYLK+Oxidation(3)	0.09	0.1495		
	NVLDINSITSVK	0.08	0.1231		
	QHLEPNLGGLTVEQAIQSK	0.08	0.1131		
	SAWMTDEEFAR	0.05	0.1749		
	SDAAIQQDPELQAWWK	0.22	0.1204		
	SKLDSQAYGDHTSIITK	0.08	0.0562		
	SLVNADGIIEK	0.08	0.1401		
	SSDFLVYGIK	0.02	0.1453		
	YARPVLGGSALPYPR	0.02	0.4751		
Glyma04g06080.2	translation initiation factor 3B1	7	0.25	0.0009	protein
	Peptide Sequnce	Ratio 4(2)F	Pvalue 4(2)F		
	AGSDTEVLWNDAR	0.17	0.0337		
	DIPIEVLELENK	0.08	0.0725		
	IIAFAWEPK	0.30	0.0519		
	IYSQIGVIK	0.11	0.0856		
	QANALFWSPAGR	0.38	0.1172		
	VSLIQIPSK	0.09	0.1141		
	VVINIFDVR	0.13	0.0898		
Glyma07g36150.1	S adenosylmethionine synthetase family protein	12	0.25	0.0000	metal handling
	Peptide Sequnce	Ratio 4(2)F	Pvalue 4(2)F		
	EHVIKPVIPEK	0.83	0.8002		
	ENFDFRPGMISINLDLK	0.22	0.4198		
	FVIGGPHGDAGLTGR	0.28	0.0001		
	IIIDTYGGWGAHGGGAFSGK	0.36	0.0023		
	LCDQISDAVLDACLEQDPDSK+Carbamidomethyl(2)Carbamidomethyl(13)	0.19	0.0004		
	NGTCPWLRPDGK+Carbamidomethyl(4)	0.94	0.9532		
	NIGFVSNDVGLDADNCK+Carbamidomethyl(16)	0.21	0.0000		
	TIFHLNPSGR	0.30	0.0106		
	TNLVMVFGEITTK	0.28	0.1015		
	TQVTVEYYNDNGAR	0.17	0.0770		

	VHTVLISTQHDETVTNDEIAADLK	0.19	0.0001		
	VLVNIEQQSPDIAQGVHGHLTK	0.23	0.0000		
Glyma17g04330.1	S adenosylmethionine synthetase family protein	12	0.25	0.0000	metal handling
	Peptide Sequnce	Ratio 4(2)F	Pvalue 4(2)F		
	EHVIKPVIPEK	0.83	0.8002		
	ENFDFRPGMISINLDLK	0.22	0.4198		
	FVIGGPHGDAGLTGR	0.28	0.0001		
	IIIDTYGGWGAHGGGAFSGK	0.36	0.0023		
	LCDQISDAVLDACLEQDPDSK+Carbamidomethyl(2)Carbamidomethyl(13)	0.19	0.0004		
	NGTCPWLRPDGK+Carbamidomethyl(4)	0.94	0.9532		
	NIGFVSNDVGLDADNCK+Carbamidomethyl(16)	0.21	0.0000		
	TIFHLNPSGR	0.30	0.0106		
	TNLVMVFGEITTK	0.28	0.1015		
	TQVTVEYYNDNGAR	0.17	0.0770		
	VHTVLISTQHDETVTNDEIAADLK	0.19	0.0001		
	VLVNIEQQSPDIAQGVHGHLTK	0.23	0.0000		
Glyma17g04340.1	S adenosylmethionine synthetase family protein	12	0.25	0.0000	metal handling
	Peptide Sequnce	Ratio 4(2)F	Pvalue 4(2)F		
	EHVIKPVIPEK	0.83	0.8002		
	ENFDFRPGMISINLDLK	0.22	0.4198		
	FVIGGPHGDAGLTGR	0.28	0.0001		
	IIIDTYGGWGAHGGGAFSGK	0.36	0.0023		
	LCDQISDAVLDACLEQDPDSK+Carbamidomethyl(2)Carbamidomethyl(13)	0.19	0.0004		
	NGTCPWLRPDGK+Carbamidomethyl(4)	0.94	0.9532		
	NIGFVSNDVGLDADNCK+Carbamidomethyl(16)	0.21	0.0000		
	TIFHLNPSGR	0.30	0.0106		
	TNLVMVFGEITTK	0.28	0.1015		
	TQVTVEYYNDNGAR	0.17	0.0770		
	VHTVLISTQHDETVTNDEIAADLK	0.19	0.0001		
	VLVNIEQQSPDIAQGVHGHLTK	0.23	0.0000		
Glyma11g20060.2	ATPase AAA type CDC48 protein	12	0.25	0.0000	cell
	Peptide Sequnce	Ratio 4(2)F	Pvalue 4(2)F		
	AIANECQANFISVK+Carbamidomethyl(6)	0.03	0.0603		
	DFSTAILER	0.07	0.0870		
	DTHGYVGADLAALCTEAALQCIR+Carbamidomethyl(14)Carbamidomethyl(21)	0.02	0.0541		

	EIDIGVPDEVGR	0.06	0.0576		
	ELQETVQYPVEHPEK	0.07	0.2833		
	ELVELPLR	0.02	0.0982		
	ETVVEVPNVSWEDIGGLENVK	0.02	0.0002		
	GILLYGPPGSGK	0.07	0.0858		
	GVLFYGPPGCGK+Carbamidomethyl(10)	0.04	0.0658		
	LAGESESNLR	2.10	0.1645		
	LDEVGYDDVGGVR	0.33	0.0383		
	YQAFAQTLQQSR	0.02	0.0478		
Glyma16g26630.1	xyloglucan endotransglucosylase/hydrolase 5	4	0.25	0.0000	cell wall
	Peptide Sequnce	Ratio 4(2)F	Pvalue 4(2)F		
	GFHIDGCEASVNAK+Carbamidomethyl(7)	0.11	0.0010		
	IYNSLWNADDWATR	0.15	0.0062		
	TGQPYILQTNVFTGGK	0.35	0.0049		
	WWDQPEFR	0.12	0.0714		
Glyma08g42500.1	spermidine hydroxycinnamoyl transferase	3	0.25	0.0169	secondary metabolism
	Peptide Sequnce	Ratio 4(2)F	Pvalue 4(2)F		
	FEAIAAHIWR	0.37	0.2793		
	GVTLLEAETTK	0.17	0.0196		
	ILVYYYPVAGR	0.32	0.0801		
Glyma08g28580.1	TRICHOME BIREFRINGENCE LIKE 19	2	0.25	0.0284	not assigned
	Peptide Sequnce	Ratio 4(2)F	Pvalue 4(2)F		
	SMAFVGDSVGR	0.36	0.1226		
	VEWPIDVSYK	0.17	0.0361		
Glyma18g51480.1	TRICHOME BIREFRINGENCE LIKE 19	2	0.25	0.0284	not assigned
	Peptide Sequnce	Ratio 4(2)F	Pvalue 4(2)F		
	SMAFVGDSVGR	0.36	0.1226		
	VEWPIDVSYK	0.17	0.0361		
Glyma0776s50.1	NADH dehydrogenase subunit 7	4	0.26	0.0378	mitochondrial electron transport
	Peptide Sequnce	Ratio 4(2)F	Pvalue 4(2)F		
	DWGFSGVMLR	0.24	0.2266		
	LLEFYER	0.37	0.1327		
	LLNCEVPLR+Carbamidomethyl(4)	0.31	0.1205		
	LVDIGTVTAQQAK	0.08	0.0781		
Glyma07g38740.1	Aluminium induced protein with YGL and LRDR motifs	2	0.26	0.0323	metal handling

	Peptide Sequnce	Ratio 4(2)F	Pvalue 4(2)F		
	ITAVPANEEEIWGATFK	0.26	0.0171		
	STSTLFVASDQYGK	0.25	0.3005		
Glyma01g38320.1	Tyrosyl tRNA synthetase class Ib bacterial/mitochondrial	2	0.26	0.0017	protein
	Peptide Sequnce	Ratio 4(2)F	Pvalue 4(2)F		
	TVEGNPCLEYIK+Carbamidomethyl(7)	0.27	0.0076		
	YLIEIWK	0.21	0.0228		
Glyma12g30060.1	ATPase AAA type CDC48 protein	15	0.26	0.0000	cell
	Peptide Sequnce	Ratio 4(2)F	Pvalue 4(2)F		
	AIANECQANFISVK+Carbamidomethyl(6)	0.03	0.0603		
	DFSTAILER	0.07	0.0870		
	DTHGYVGADLAALCTEAALQCIR+Carbamidomethyl(14)Carbamidomethyl(21)	0.02	0.0541		
	EIDIGVPDEVGR	0.06	0.0576		
	ELQETVQYPVEHPEK	0.07	0.2833		
	ELVELPLR	0.02	0.0982		
	GILLYGPPGSGK	0.07	0.0858		
	GVLFYGPPGCGK+Carbamidomethyl(10)	0.04	0.0658		
	LAGESESNLR	2.10	0.1645		
	LDEVGYDDVGGVR	0.33	0.0383		
	LDQLIYIPLPDEDSR	0.06	0.0284		
	LGDVVSVHQCPDVK+Carbamidomethyl(10)	0.55	0.1658		
	TVFIIGATNRPDIIDPALLRPGR	0.01	0.3223		
	VLNQLLTEMDGMSAK	0.08	0.1267		
	YQAFAQTLQQSR	0.02	0.0478		
Glyma1337s00200.1	S adenosylmethionine synthetase 2	11	0.26	0.0000	amino acid metabolism
	Peptide Sequnce	Ratio 4(2)F	Pvalue 4(2)F		
	EHVIKPVIPEK	0.83	0.8002		
	ENFDFRPGMISINLDLK	0.22	0.4198		
	FVIGGPHGDAGLTGR	0.28	0.0001		
	IIIDTYGGWGAHGGGAFSGK	0.36	0.0023		
	LCDQISDAVLDACLEQDPDSK+Carbamidomethyl(2)Carbamidomethyl(13)	0.19	0.0004		
	NGTCPWLRPDGK+Carbamidomethyl(4)	0.94	0.9532		
	TIFHLNPSGR	0.30	0.0106		
	TNLVMVFGEITTK	0.28	0.1015		
	TQVTVEYYNDNGAR	0.17	0.0770		

	VHTVLISTQHDETVTNDEIAADLK	0.19	0.0001		
	VLVNIEQQSPDIAQGVHGHLTK	0.23	0.0000		
Glyma06g48260.1	cellulose synthase like G1	7	0.26	0.0020	cell wall
	Peptide Sequnce	Ratio 4(2)F	Pvalue 4(2)F		
	EWVPFCNIYGVK+Carbamidomethyl(6)	0.13	0.0624		
	GGALNALLR	0.04	0.1497		
	GPGLSGSGNYLSR	0.27	0.0066		
	IEIINDQPGMPLVVYVSR	0.08	0.2304		
	SALLFGSPNQK	0.29	0.0720		
	TMWQGMDGLR	0.47	0.2579		
	TPCFLGCAPTDIK+Carbamidomethyl(3)Carbamidomethyl(7)	0.16	0.1527		
Glyma12g02320.1	Radical SAM superfamily protein	2	0.26	0.0157	Co-factor and vitamine metabolism
	Peptide Sequnce	Ratio 4(2)F	Pvalue 4(2)F		
	LLTTPNNDFDADQLMFK	0.31	0.1222		
	TNFNQILEYVK	0.22	0.0181		
Glyma06g47200.1	Plant invertase/pectin methylesterase inhibitor superfamily	2	0.26	0.0268	cell wall
	Peptide Sequnce	Ratio 4(2)F	Pvalue 4(2)F		
	EGLYEEYVVIPK	0.24	0.0270		
	SPSLNHAETASLGDCR+Carbamidomethyl(15)	0.28	0.1530		
Glyma17g00710.1	glycosyl hydrolase 9A1	4	0.26	0.0004	cell wall
	Peptide Sequnce	Ratio 4(2)F	Pvalue 4(2)F		
	ALLFFNAQK	0.21	0.0650		
	DLVGGYYDAGDAIK	0.06	0.0072		
	WGTDYLLK	0.35	0.0152		
	YVDLGCIIVSR+Carbamidomethyl(6)	0.21	0.0824		
Glyma02g39630.1	NAD(P) binding Rossmann fold superfamily protein	2	0.26	0.0115	secondary metabolism
	Peptide Sequnce	Ratio 4(2)F	Pvalue 4(2)F		
	LFQMDLLR	0.52	0.3651		
	LMDLGLQFIPMEK	0.23	0.0042		
Glyma14g37680.1	NAD(P) binding Rossmann fold superfamily protein	2	0.26	0.0115	secondary metabolism
	Peptide Sequnce	Ratio 4(2)F	Pvalue 4(2)F		
	LFQMDLLR	0.52	0.3651		
	LMDLGLQFIPMEK	0.23	0.0042		
Glyma13g33960.1	rhamnose biosynthesis 1	14	0.26	0.0000	cell wall

	Peptide Sequnce	Ratio 4(2)F	Pvalue 4(2)F		
	EYDNVCTLR+Carbamidomethyl(6)	0.07	0.0898		
	FVENRPFNDQR	0.15	0.0046		
	GIWNFTNPGAVSHNEILEMYR	0.04	0.0269		
	GNNVYGPNQFPEK	0.85	0.7744		
	ILGWSER	0.09	0.0738		
	LKNEFPELLSIK	0.31	0.3364		
	MPISSDLSNPR	0.20	0.1561		
	NEFPELLSIK	0.11	0.0492		
	NILITGAAGFIASHVANR	0.11	0.0299		
	QGIPYEYGK	0.30	0.0336		
	SYGLPVITTR	0.09	0.0462		
	TGWIGGLLGK	0.27	0.0272		
	TNVAGTLTLADVSR	0.11	0.0615		
	WANFNLEEQAK	0.13	0.0334		
Glyma13g22890.1	Raffinose synthase family protein	3	0.27	0.0163	minor CHO metabolism
	Peptide Sequnce	Ratio 4(2)F	Pvalue 4(2)F		
	DCLFTDPAR+Carbamidomethyl(2)	0.07	0.0512		
	EMEEYGSVMK	0.52	0.3448		
	VSSGVTENEPTWK	0.15	0.0237		
Glyma04g12320.2	Ribosomal L5P family protein	4	0.27	0.0102	protein
	Peptide Sequnce	Ratio 4(2)F	Pvalue 4(2)F		
	AMQLLESGLK	0.15	0.1335		
	IACYVTVR+Carbamidomethyl(3)	0.44	0.1727		
	LVLNISVGESGDR	0.15	0.0469		
	VLEQLSGQTPVFSK	0.12	0.0410		
Glyma06g47510.1	Ribosomal L5P family protein	4	0.27	0.0102	protein
	Peptide Sequnce	Ratio 4(2)F	Pvalue 4(2)F		
	AMQLLESGLK	0.15	0.1335		
	IACYVTVR+Carbamidomethyl(3)	0.44	0.1727		
	LVLNISVGESGDR	0.15	0.0469		
	VLEQLSGQTPVFSK	0.12	0.0410		
Glyma06g48130.1	Ribosomal L5P family protein	4	0.27	0.0102	protein
	Peptide Sequnce	Ratio 4(2)F	Pvalue 4(2)F		
	AMQLLESGLK	0.15	0.1335		

	IACYVTVR+Carbamidomethyl(3)	0.44	0.1727		
	LVLNISVGESGDR	0.15	0.0469		
	VLEQLSGQTPVFSK	0.12	0.0410		
Glyma0985s00200.1	Ribosomal L5P family protein	4	0.27	0.0102	protein
	Peptide Sequnce	Ratio 4(2)F	Pvalue 4(2)F		
	AMQLLESGLK	0.15	0.1335		
	IACYVTVR+Carbamidomethyl(3)	0.44	0.1727		
	LVLNISVGESGDR	0.15	0.0469		
	VLEQLSGQTPVFSK	0.12	0.0410		
Glyma12g15800.1	Ribosomal L5P family protein	4	0.27	0.0102	protein
	Peptide Sequnce	Ratio 4(2)F	Pvalue 4(2)F		
	AMQLLESGLK	0.15	0.1335		
	IACYVTVR+Carbamidomethyl(3)	0.44	0.1727		
	LVLNISVGESGDR	0.15	0.0469		
	VLEQLSGQTPVFSK	0.12	0.0410		
Glyma15g36190.2	Ribosomal L5P family protein	4	0.27	0.0102	protein
	Peptide Sequnce	Ratio 4(2)F	Pvalue 4(2)F		
	AMQLLESGLK	0.15	0.1335		
	IACYVTVR+Carbamidomethyl(3)	0.44	0.1727		
	LVLNISVGESGDR	0.15	0.0469		
	VLEQLSGQTPVFSK	0.12	0.0410		
Glyma07g40090.1	glycosyl hydrolase 9A1	3	0.27	0.0006	cell wall
	Peptide Sequnce	Ratio 4(2)F	Pvalue 4(2)F		
	ALLFFNAQK	0.21	0.0650		
	DLVGGYYDAGDAIK	0.06	0.0072		
	WGTDYLLK	0.35	0.0152		
Glyma12g09250.1	TCP 1/cpn60 chaperonin family protein	12	0.27	0.0002	protein
	Peptide Sequnce	Ratio 4(2)F	Pvalue 4(2)F		
	AIDNIFK	0.27	0.0574		
	EGCTAGIDVISGSVGDMAER+Carbamidomethyl(3)	0.24	0.1470		
	EVTVTNDGATILK	0.27	0.0218		
	GSTNLESIQIIK	0.29	0.0755		
	IIGHGINCFVNR+Carbamidomethyl(8)	0.31	0.0901		
	ILVANTAMDTDK	0.28	0.1453		
	LAVDAVMR	0.31	0.1787		

	LGHCDLIEEIMIGEDK+Carbamidomethyl(4)	0.33	0.1423		
	SDLLNIAMTTLSSK	0.25	0.0744		
	SLAIEAFSR	0.23	0.0841		
	VDEIITCAPR+Carbamidomethyl(7)	0.44	0.1283		
	VLLGGGWPEMVMAK	0.21	0.1049		
Glyma10g16790.1	UDP Glycosyltransferase superfamily protein	8	0.28	0.0002	secondary metabolism
	Peptide Sequnce	Ratio 4(2)F	Pvalue 4(2)F		
	AYSSCDMFLLR+Carbamidomethyl(5)	0.09	0.0464		
	DVEEEDNNPDWVK	0.04	0.1229		
	FFWALR	0.12	0.0648		
	IPETLQPSIK	0.20	0.0977		
	LAIVDEEGSDYRK	0.34	0.0966		
	LAYEALQGPVSELLK	0.16	0.0278		
	LSQQDVTELAHGIELSGLR	0.32	0.0130		
	VPVVPVGLVPPSIQIR	0.12	0.2922		
Glyma06g42460.1	Ribosomal L5P family protein	4	0.28	0.0187	protein
	Peptide Sequnce	Ratio 4(2)F	Pvalue 4(2)F		
	AMQLLESGLK	0.15	0.1335		
	IACYVTVR+Carbamidomethyl(3)	0.44	0.1727		
	LVLNISVGESGDR	0.15	0.0469		
	VLEQLSGQAPVFSK	0.24	0.0953		
Glyma11g19220.1	TCP 1/cpn60 chaperonin family protein	11	0.28	0.0002	protein
	Peptide Sequnce	Ratio 4(2)F	Pvalue 4(2)F		
	AIDNIFK	0.27	0.0574		
	EVTVTNDGATILK	0.27	0.0218		
	GSTNLESIQIIK	0.29	0.0755		
	IIGHGINCFVNR+Carbamidomethyl(8)	0.31	0.0901		
	ILVANTAMDTDK	0.28	0.1453		
	LAVDAVMR	0.31	0.1787		
	LGHCDLIEEIMIGEDK+Carbamidomethyl(4)	0.33	0.1423		
	SDLLNIAMTTLSSK	0.25	0.0744		
	SLAIEAFSR	0.23	0.0841		
	VDEIITCAPR+Carbamidomethyl(7)	0.44	0.1283		
	VLLGGGWPEMVMAK	0.21	0.1049		
Glyma09g02310.1	vesicle associated membrane protein 726	2	0.28	0.0324	cell

	Peptide Sequnce	Ratio 4(2)F	Pvalue 4(2)F		
	IELLVDK	0.26	0.0780		
	SLIYAFVSR	0.30	0.0662		
Glyma15g13220.1	vesicle associated membrane protein 726	2	0.28	0.0324	cell
	Peptide Sequnce	Ratio 4(2)F	Pvalue 4(2)F		
	IELLVDK	0.26	0.0780		
	SLIYAFVSR	0.30	0.0662		
Glyma03g33000.12	pyrophosphorylase 4	2	0.28	0.0402	nucleotide metabolism
	Peptide Sequnce	Ratio 4(2)F	Pvalue 4(2)F		
	IIAVCADDPEYR+Carbamidomethyl(5)	0.23	0.0290		
	VLYSSVVYPHNYGFIPR	0.41	0.2312		
Glyma19g35710.1	pyrophosphorylase 4	2	0.28	0.0402	nucleotide metabolism
	Peptide Sequnce	Ratio 4(2)F	Pvalue 4(2)F		
	IIAVCADDPEYR+Carbamidomethyl(5)	0.23	0.0290		
	VLYSSVVYPHNYGFIPR	0.41	0.2312		
Glyma09g04290.1	secretion associated RAS super family 2	3	0.28	0.0007	signalling
	Peptide Sequnce	Ratio 4(2)F	Pvalue 4(2)F		
	IDIPYAASEDELR	0.32	0.1035		
	ILFLGLDNAGK	0.30	0.1098		
	LVQHQPTQHPTSEELSIGK	0.27	0.0008		
Glyma15g15330.1	secretion associated RAS super family 2	3	0.28	0.0007	signalling
	Peptide Sequnce	Ratio 4(2)F	Pvalue 4(2)F		
	IDIPYAASEDELR	0.32	0.1035		
	ILFLGLDNAGK	0.30	0.1098		
	LVQHQPTQHPTSEELSIGK	0.27	0.0008		
Glyma07g13900.1	Hyaluronan / mRNA binding family	4	0.28	0.0002	RNA
	Peptide Sequnce	Ratio 4(2)F	Pvalue 4(2)F		
	DFSNDDNSSAPANQGSFEGDSGNHSER	0.06	0.0033		
	EFASMQPLSNK	0.21	0.0413		
	EMTLEEYEK	0.14	0.0142		
	ENDEIFIK	0.60	0.1096		
Glyma08g20900.2	ubiquitin specific protease 7	4	0.28	0.0012	protein
	Peptide Sequnce	Ratio 4(2)F	Pvalue 4(2)F		
	ALFGIELISR	0.12	0.0499		

	LMMMGTADEVVK	0.15	0.0333		
	SLGISENPDAVK	0.42	0.0646		
	YLTVQFVR	0.13	0.0252		
Glyma09g23330.1	UDP glucosyl transferase 88A1	2	0.28	0.0340	misc
	Peptide Sequnce	Ratio 4(2)F	Pvalue 4(2)F		
	FLWVVR	0.10	0.0481		
	ILNSISQTSNLK	0.36	0.1139		
Glyma09g23600.1	UDP glucosyl transferase 88A1	2	0.28	0.0340	misc
	Peptide Sequnce	Ratio 4(2)F	Pvalue 4(2)F		
	FLWVVR	0.10	0.0481		
	ILNSISQTSNLK	0.36	0.1139		
Glyma16g29340.1	UDP glucosyl transferase 88A1	2	0.28	0.0340	misc
	Peptide Sequnce	Ratio 4(2)F	Pvalue 4(2)F		
	FLWVVR	0.10	0.0481		
	ILNSISQTSNLK	0.36	0.1139		
Glyma16g29370.1	UDP glucosyl transferase 88A1	2	0.28	0.0340	misc
	Peptide Sequnce	Ratio 4(2)F	Pvalue 4(2)F		
	FLWVVR	0.10	0.0481		
	ILNSISQTSNLK	0.36	0.1139		
Glyma04g01920.1	catalase 2	4	0.28	0.0193	redox
	Peptide Sequnce	Ratio 4(2)F	Pvalue 4(2)F		
	DEEVNYFPSR	0.28	0.0031		
	GFFEVTHDISHLTCADFLR+Carbamidomethyl(14)	0.00	1.0000		
	GPILLEDYHLVEK	0.57	0.2129		
	LGPNYLQLPANAPK	0.11	0.1656		
Glyma06g02040.1	catalase 2	4	0.28	0.0193	redox
	Peptide Sequnce	Ratio 4(2)F	Pvalue 4(2)F		
	DEEVNYFPSR	0.28	0.0031		
	GFFEVTHDISHLTCADFLR+Carbamidomethyl(14)	0.00	1.0000		
	GPILLEDYHLVEK	0.57	0.2129		
	LGPNYLQLPANAPK	0.11	0.1656		
Glyma17g38140.1	catalase 2	4	0.28	0.0193	redox
	Peptide Sequnce	Ratio 4(2)F	Pvalue 4(2)F		
	DEEVNYFPSR	0.28	0.0031		

	GFFEVTHDISHLTCADFLR+Carbamidomethyl(14)	0.00	1.0000		
	GPILLEDYHLVEK	0.57	0.2129		
	LGPNYLQLPANAPK	0.11	0.1656		
Glyma02g00590.1	51 kDa subunit of complex I	5	0.28	0.0157	mitochondrial electron transport
	Peptide Sequnce	Ratio 4(2)F	Pvalue 4(2)F		
	AVGTGLGTAAVIVMDK	0.28	0.0367		
	IFTNLYGLHDPFLK	0.12	0.0577		
	LEEIDMLQELTK	0.38	0.1830		
	LLEGCLIAGVGMR+Carbamidomethyl(5)	0.29	0.3050		
	STDVVDAIAR	0.07	0.1494		
Glyma10g00820.1	51 kDa subunit of complex I	5	0.28	0.0157	mitochondrial electron transport
	Peptide Sequnce	Ratio 4(2)F	Pvalue 4(2)F		
	AVGTGLGTAAVIVMDK	0.28	0.0367		
	IFTNLYGLHDPFLK	0.12	0.0577		
	LEEIDMLQELTK	0.38	0.1830		
	LLEGCLIAGVGMR+Carbamidomethyl(5)	0.29	0.3050		
	STDVVDAIAR	0.07	0.1494		
Glyma05g03140.1	adenylate kinase 1	6	0.28	0.0000	nucleotide metabolisminase
	Peptide Sequnce	Ratio 4(2)F	Pvalue 4(2)F		
	GFILDGFPR	0.31	0.0173		
	GLVANLHAEKPPK	0.67	0.7517		
	LILIGPPGSGK	0.25	0.0081		
	QTEPVIDYYSK	0.49	0.1250		
	VLGVDDVTGEPLIQR	0.25	0.0003		
	VLNFAIDDAILEER	0.28	0.0000		
Glyma15g09800.1	ubiquitin like protein 5	2	0.28	0.0497	protein
	Peptide Sequnce	Ratio 4(2)F	Pvalue 4(2)F		
	IEVVLNDR	0.27	0.0976		
	MIEVVLNDR	0.30	0.0885		
Glyma07g37070.1	secretion associated RAS super family 2	3	0.29	0.0011	signalling
	Peptide Sequnce	Ratio 4(2)F	Pvalue 4(2)F		
	IDIPYAASEEELR	0.39	0.1681		
	ILFLGLDNAGK	0.30	0.1098		
	LVQHQPTQHPTSEELSIGK	0.27	0.0008		
Glyma17g03540.1	secretion associated RAS super family 2	3	0.29	0.0011	signalling

	Peptide Sequnce	Ratio 4(2)F	Pvalue 4(2)F		
	IDIPYAASEEELR	0.39	0.1681		
	ILFLGLDNAGK	0.30	0.1098		
	LVQHQPTQHPTSEELSIGK	0.27	0.0008		
Glyma09g29900.1	glutamate decarboxylase 5	3	0.29	0.0238	amino acid metabolism
	Peptide Sequnce	Ratio 4(2)F	Pvalue 4(2)F		
	FGWIVPAYTMPPDAQHIAVLR+Oxidation(10)	0.03	0.0610		
	NYVDMDEYPVTTELQNR	0.37	0.0610		
	YFEVELK	0.36	0.1831		
Glyma16g34450.1	glutamate decarboxylase 5	3	0.29	0.0238	amino acid metabolism
	Peptide Sequnce	Ratio 4(2)F	Pvalue 4(2)F		
	FGWIVPAYTMPPDAQHIAVLR+Oxidation(10)	0.03	0.0610		
	NYVDMDEYPVTTELQNR	0.37	0.0610		
	YFEVELK	0.36	0.1831		
Glyma08g47161.1	Pectin lyase like superfamily protein	2	0.29	0.0345	cell wall
	Peptide Sequnce	Ratio 4(2)F	Pvalue 4(2)F		
	LANCGIGFGR+Carbamidomethyl(4)	0.38	0.0993		
	YLAPLNPFAK	0.13	0.0562		
Glyma18g38430.1	Pectin lyase like superfamily protein	2	0.29	0.0345	cell wall
	Peptide Sequnce	Ratio 4(2)F	Pvalue 4(2)F		
	LANCGIGFGR+Carbamidomethyl(4)	0.38	0.0993		
	YLAPLNPFAK	0.13	0.0562		
Glyma04g43470.1	cellulose synthase like G2	4	0.29	0.0054	cell wall
	Peptide Sequnce	Ratio 4(2)F	Pvalue 4(2)F		
	GPGLSGSGNYLSR	0.27	0.0066		
	SALLFGSPNQK	0.29	0.0720		
	TMWQGMDGLR	0.47	0.2579		
	TPCFLGCAPTDIK+Carbamidomethyl(3)Carbamidomethyl(7)	0.16	0.1527		
Glyma05g25320.1	cell division control 2	3	0.29	0.0025	cell
	Peptide Sequnce	Ratio 4(2)F	Pvalue 4(2)F		
	IGEGTYGVVYK	0.25	0.0016		
	LADFGLAR	0.32	0.0482		
	LEQEDEGVPSTAIR	0.62	0.5116		
Glyma08g08330.1	cell division control 2	3	0.29	0.0025	cell

	Peptide Sequnce	Ratio 4(2)F	Pvalue 4(2)F		
	IGEGTYGVVYK	0.25	0.0016		
	LADFGLAR	0.32	0.0482		
	LEQEDEGVPSTAIR	0.62	0.5116		
Glyma09g03470.1	cell division control 2	3	0.29	0.0025	cell
	Peptide Sequnce	Ratio 4(2)F	Pvalue 4(2)F		
	IGEGTYGVVYK	0.25	0.0016		
	LADFGLAR	0.32	0.0482		
	LEQEDEGVPSTAIR	0.62	0.5116		
Glyma15g14390.1	cell division control 2	3	0.29	0.0025	cell
	Peptide Sequnce	Ratio 4(2)F	Pvalue 4(2)F		
	IGEGTYGVVYK	0.25	0.0016		
	LADFGLAR	0.32	0.0482		
	LEQEDEGVPSTAIR	0.62	0.5116		
Glyma13g20480.1	methionine adenosyltransferase 3	5	0.29	0.0000	amino acid metabolism
	Peptide Sequnce	Ratio 4(2)F	Pvalue 4(2)F		
	ETFLFTSESVNEGHPDK	0.07	0.1438		
	FVIGGPHGDAGLTGR	0.28	0.0001		
	ICDQVSDAILDACLEQDPESK+Carbamidomethyl(2)Carbamidomethyl(13)	0.07	0.1224		
	IIIDTYGGWGAHGGGAFSGK	0.36	0.0023		
	TNMVMVFGEITTK	0.28	0.1659		
Glyma14g17880.1	rhamnose biosynthesis 1	5	0.29	0.0305	cell wall
	Peptide Sequnce	Ratio 4(2)F	Pvalue 4(2)F		
	AMVEDLLK	0.46	0.2360		
	FILLAMK	0.00	1.0000		
	MPISSDLSNPR	0.20	0.1561		
	NYDNVCTLR+Carbamidomethyl(6)	0.28	0.0485		
	TGWIGGLLGK	0.27	0.0272		
Glyma17g29120.1	rhamnose biosynthesis 1	5	0.29	0.0305	cell wall
	Peptide Sequnce	Ratio 4(2)F	Pvalue 4(2)F		
	AMVEDLLK	0.46	0.2360		
	FILLAMK	0.00	1.0000		
	MPISSDLSNPR	0.20	0.1561		
	NYDNVCTLR+Carbamidomethyl(6)	0.28	0.0485		

	TGWIGGLLGK	0.27	0.0272		
Glyma17g13760.1	adenylate kinase 1	7	0.29	0.0000	nucleotide metabolism
	Peptide Sequnce	Ratio 4(2)F	Pvalue 4(2)F		
	GFILDGFPR	0.31	0.017		
	GLVANLHAEKPPK	0.67	0.752		
	LDEMLQNQGVK	0.46	0.071		
	LILIGPPGSGK	0.25	0.008		
	QTEPVIDYYSK	0.49	0.125		
	VLGVDDVTGEPLIQR	0.25	0.000		
	VLNFAIDDAILEER	0.28	0.000		
Glyma03g00470.1	translation initiation factor 3B1	12	0.29	0.0003	protein
	Peptide Sequnce	Ratio 4(2)F	Pvalue 4(2)F		
	AGSDTEVLWNDAR	0.17	0.0337		
	DIPIEVLELENK	0.08	0.0725		
	EDGLWMPVDPETEK	0.13	0.1503		
	GSADDFAVGGAGGVTGVSWPVFK	0.57	0.3989		
	IIAFAWEPK	0.30	0.0519		
	IYSQIGVIK	0.11	0.0856		
	LGVDLSTLDLDAIR	0.06	0.0729		
	NLFSVSDCK+Carbamidomethyl(8)	0.23	0.1221		
	QANALFWSPAGR	0.38	0.1172		
	STYTGFELFR	0.08	0.1323		
	VSLIQIPSK	0.09	0.1141		
	VVINLFDVR	0.13	0.0898		
Glyma14g39810.1	catalase 2	3	0.29	0.0230	redox
	Peptide Sequnce	Ratio 4(2)F	Pvalue 4(2)F		
	DEEVNYFPSR	0.28	0.0031		
	GFFEVTHDISHLTCADFLR+Carbamidomethyl(14)	0.00	1.0000		
	GPILLEDYHLVEK	0.57	0.2129		
Glyma08g00920.1	calnexin 1	6	0.29	0.0003	signalling
	Peptide Sequnce	Ratio 4(2)F	Pvalue 4(2)F		
	EFDNESPYSIMFGPDK	0.38	0.0829		
	IADIPFLSAYK	0.13	0.0547		
	LQNGLECGGAYLK+Carbamidomethyl(7)	0.32	0.0340		
	VFDLLYK	0.25	0.0371		

	VNFLSADDFEPPLIPPK	0.32	0.0085		
	YLRPQEAGWK	0.35	0.2456		
Glyma05g31950.2	Small nuclear ribonucleoprotein family protein	3	0.3	0.0149	RNA
	Peptide Sequnce	Ratio 4(2)F	Pvalue 4(2)F		
	FMVIPDMLK	0.26	0.0837		
	LLHEASGHVVTVELK	0.30	0.1179		
	TSQLEHVFIR	0.32	0.0375		
Glyma08g15260.1	Small nuclear ribonucleoprotein family protein	3	0.3	0.0149	RNA
	FMVIPDMLK	0.26	0.0837		
	LLHEASGHVVTVELK	0.30	0.1179		
	TSQLEHVFIR	0.32	0.0375		
Glyma10g06170.1	methionine adenosyltransferase 3	5	0.3	0.0000	amino acid metabolism
	Peptide Sequnce	Ratio 4(2)F	Pvalue 4(2)F		
	ETFLFTSESVNEGHPDK	0.07	0.1438		
	FVIGGPHGDAGLTGR	0.28	0.0001		
	IIIDTYGGWGAHGGGAFSGK	0.36	0.0023		
	TIFHLNPSGR	0.30	0.0106		
	TNMVMVFGEITTK	0.28	0.1659		
Glyma17g34480.1	glycine decarboxylase P protein 2	2	0.3	0.0263	photosynthesis
	Peptide Sequnce	Ratio 4(2)F	Pvalue 4(2)F		
	EYAAFPAPWLR	0.32	0.0505		
	FCDALISIR+Carbamidomethyl(2)	0.25	0.0799		
Glyma13g42340.1	lipoxygenase 1	24	0.3	0.0077	hormone metabolism
	Peptide Sequnce	Ratio 4(2)F	Pvalue 4(2)F		
	DEAFGHLK	5.96	0.4941		
	DGGDFWTSDAGPLEAFK	0.09	0.4162		
	DTMNINSLAR	0.05	0.2120		
	DWVFTDQALPNDLVK	0.04	0.1444		
	ETIAGVNPNVIK	0.01	0.1459		
	FGIIGGNK	0.00	1.0000		
	IAPIPVIK	0.05	0.1572		
	IFFANNTYLPSETPAPLLK	0.11	0.1055		
	ILEEFPPR	0.01	0.2002		
	IYDYDVYNDLGNPDSGDK	0.65	0.0966		
	LDTQAYGDHTCIITK+Carbamidomethyl(11)	0.00	1.0000		

	LFILDHHDYLIPYLR	0.01	0.4093		
	LLFPHYR	0.29	0.1725		
	LYEGGVTLPTNFLSK	0.06	0.1284		
	NFMQVEFYLK	0.24	0.1464		
	NFMQVEFYLK+Oxidation(3)	0.09	0.1495		
	NVLDINSITSVK	0.08	0.1231		
	QHLEPNLGGLTVEQAIQSK	0.08	0.1131		
	SAWMTDEEFAR	0.05	0.1749		
	SDAAIQQDPELQAWWK	0.22	0.1204		
	SLVNADGIIEK	0.08	0.1401		
	SSDFLAFGIK	0.63	0.5093		
	SWVQEYVSFYYK	0.04	0.0920		
	YARPVLGGSALPYPR	0.02	0.4751		
Glyma15g02610.1	Ribosomal L28e protein family	2	0.3	0.0289	protein
	Peptide Sequnce	Ratio 4(2)F	Pvalue 4(2)F		
	AVQNQVADNYYRPDLK	0.34	0.0319		
	EPNNLYNLNTFK	0.20	0.1415		
Glyma07g30150.2	dynamin related protein 3A	5	0.3	0.0107	misc
	Peptide Sequnce	Ratio 4(2)F	Pvalue 4(2)F		
	AGQGALLLNILSK	0.09	0.1724		
	LGYVGVVNR	0.17	0.1078		
	LLDPSLQCAR+Carbamidomethyl(8)	0.65	0.5034		
	SLEEVDPCEDLTDDDIR+Carbamidomethyl(8)	0.10	0.0044		
	SSVLEALVGR	0.23	0.2424		
Glyma16g01230.1	oligouridylate binding protein 1B	3	0.3	0.0183	RNA
	Peptide Sequnce	Ratio 4(2)F	Pvalue 4(2)F		
	GFGFVSFR	0.28	0.0069		
	NQQDAQSAINDLTGK	0.32	0.4130		
	VNWAYASGQR	0.36	0.1700		
Glyma12g36810.1	2 oxoglutarate (2OG) and Fe(II) dependent oxygenase superfamily protein	2	0.3	0.0458	not assigned
	Peptide Sequnce	Ratio 4(2)F	Pvalue 4(2)F		
	FPGLNIWLR	0.30	0.0791		
	VPIGCLLIQTGK+Carbamidomethyl(5)	0.31	0.0990		
Glyma10g28500.1	S adenosylmethionine synthetase 2	7	0.31	0.0000	amino acid metabolism
	Peptide Sequnce	Ratio 4(2)F	Pvalue 4(2)F		

	DDPDFTWEVVKPLK	0.45	0.0651		
	EHVIKPVIPEK	0.83	0.8002		
	FVIGGPHGDAGLTGR	0.28	0.0001		
	LCDQISDAVLDACLEQDPESK+Carbamidomethyl(2)Carbamidomethyl(13)	0.07	0.1224		
	RPEDIGAGDQGHMFGYATDETPELMPLSHVLATK+Oxidation(13)	0.56	0.2977		
	TNLVMVFGEITTK	0.28	0.1015		
	VHTVLISTQHDETVTNDEIAADLK	0.19	0.0001		
Glyma03g32140.3	ribophorin II (RPN2) family protein	3	0.31	0.0012	development
	Peptide Sequnce	Ratio 4(2)F	Pvalue 4(2)F		
	LPPLPVDPYSR	0.35	0.0245		
	VNTVLGSAAPPLTVK	0.39	0.1497		
	YDDGTFYFDEK	0.23	0.0047		
Glyma20g21431.1	Ribosomal protein L14p/L23e family protein	2	0.31	0.0052	protein
	Peptide Sequnce	Ratio 4(2)F	Pvalue 4(2)F		
	ECADLWPR+Carbamidomethyl(2)	0.3384494	0.022108701		
	VLPAVIVR	0.238925497	0.028196559		
Glyma08g42270.1	rhamnose biosynthesis 1	7	0.31	0.0349	cell wall
	Peptide Sequnce	Ratio 4(2)F	Pvalue 4(2)F		
	EYENVCTLR+Carbamidomethyl(6)	0.13	0.0765		
	FLILAMK	0.00	1.0000		
	GNNVYGPNQFPEK	0.85	0.7744		
	LFNLDPDK	0.60	0.2483		
	NPDWWGDVSGALLPHPR	0.03	0.0056		
	SYGLPVITTR	0.09	0.0462		
	TMDWYVK	0.00	1.0000		
Glyma20g03060.1	Nuclear transport factor 2 (NTF2) family protein	7	0.31	0.0000	protein
	Peptide Sequnce	Ratio 4(2)F	Pvalue 4(2)F		
	EVSQPLENGNVSVTEK	0.30	0.0604		
	FTQSFFLAPQDK	0.33	0.0074		
	GYFVLNDVFR	0.40	0.0243		
	ILSLDYTSFR	0.20	0.0022		
	SFASIVNALK	0.30	0.0002		
	VEILSADAQPSFK	0.12	0.0018		
	VSSIPAPEAPAPSIESPPEK	0.14	0.0002		
Glyma09g00550.1	beta glucosidase 13	2	0.31	0.0058	misc

	Peptide Sequnce	Ratio 4(2)F	Pvalue 4(2)F		
	FGLVYVDYK	0.32	0.0167		
	FSISWPR	0.24	0.0423		
Glyma12g36870.1	beta glucosidase 13	2	0.31	0.0058	misc
	Peptide Sequnce	Ratio 4(2)F	Pvalue 4(2)F		
	FGLVYVDYK	0.32	0.0167		
	FSISWPR	0.24	0.0423		
Glyma01g41990.1	Glycosyl hydrolases family 32 protein	8	0.31	0.0000	major CHO metabolism
	Peptide Sequnce	Ratio 4(2)F	Pvalue 4(2)F		
	DFRDPTTAWITSEGK	0.71	0.6666		
	DPTTAWITSEGK	0.18	0.0048		
	IFGSFVPVLK	0.12	0.0006		
	ILVDHSIVESFAQGGR	0.40	0.0175		
	NWMNDPNGPMYYK	0.27	0.1644		
	VLWGWIGESDSEYADVAK	0.16	0.0085		
	VSLDDDRHDYYALGTYDEK	0.55	0.7208		
	YDYGIFYASK	0.40	0.0395		
Glyma13g44261.1	Cystathionine beta synthase (CBS) protein	3	0.31	0.0012	not assigned
	Peptide Sequnce	Ratio 4(2)F	Pvalue 4(2)F		
	AGEGDGELQSILSR	0.63	0.3947		
	ELVTCFAESPLSEVIEK+Carbamidomethyl(5)	0.09	0.0015		
	LIGTFSSTDLR	0.19	0.0281		
Glyma08g07160.1	dynamin related protein 3A	6	0.31	0.0078	misc
	Peptide Sequnce	Ratio 4(2)F	Pvalue 4(2)F		
	AGQGALLLNILSK	0.09	0.1724		
	LGYVGVVNR	0.17	0.1078		
	LLDPSLQCAR+Carbamidomethyl(8)	0.65	0.5034		
	SLEEVDPCEDLTDDDIR+Carbamidomethyl(8)	0.10	0.0044		
	SSVLEALVGR	0.23	0.2424		
	VPVGDQPSDIEAR	0.34	0.1383		
Glyma13g32940.1	dynamin related protein 3A	6	0.31	0.0078	misc
	Peptide Sequnce	Ratio 4(2)F	Pvalue 4(2)F		
	AGQGALLLNILSK	0.09	0.1724		
	LGYVGVVNR	0.17	0.1078		
	LLDPSLQCAR+Carbamidomethyl(8)	0.65	0.5034		

	SLEEVDPCEDLTDDDIR+Carbamidomethyl(8)	0.10	0.0044		
	SSVLEALVGR	0.23	0.2424		
	VPVGDQPSDIEAR	0.34	0.1383		
Glyma15g06380.1	dynamin related protein 3A	6	0.31	0.0078	misc
	Peptide Sequnce	Ratio 4(2)F	Pvalue 4(2)F		
	AGQGALLLNILSK	0.09	0.1724		
	LGYVGVVNR	0.17	0.1078		
	LLDPSLQCAR+Carbamidomethyl(8)	0.65	0.5034		
	SLEEVDPCEDLTDDDIR+Carbamidomethyl(8)	0.10	0.0044		
	SSVLEALVGR	0.23	0.2424		
	VPVGDQPSDIEAR	0.34	0.1383		
Glyma05g28730.1	Protein of unknown function DUF538	2	0.31	0.0201	not assigned
	Peptide Sequnce	Ratio 4(2)F	Pvalue 4(2)F		
	LSYGSITNLK	0.31	0.0348		
	YGLPSGLLPDTVTDYK	0.33	0.0845		
Glyma01g38410.1	aminopeptidase P1	3	0.32	0.0321	protein
	Peptide Sequnce	Ratio 4(2)F	Pvalue 4(2)F		
	IGEDPAVDIWMADNLPK	0.06	0.0656		
	IYLFDSGAQYLDGTTDITR	0.40	0.1453		
	LTEVTVSDQLEGFR	0.38	0.1061		
Glyma15g06020.1	3 deoxy d arabino heptulosonate 7 phosphate synthase	2	0.32	0.0034	amino acid metabolism
	Peptide Sequnce	Ratio 4(2)F	Pvalue 4(2)F		
	AFATGGYAAMQR	0.07	0.0763		
	GVANPLGIK	0.34	0.0051		
Glyma15g27510.3	rhamnose biosynthesis 1	9	0.32	0.0000	cell wall
	Peptide Sequnce	Ratio 4(2)F	Pvalue 4(2)F		
	EYDNVCTLR+Carbamidomethyl(6)	0.07	0.0898		
	FILLAMQGK	0.11	0.1120		
	FVENRPFNDQR	0.15	0.0046		
	GNNVYGPNQFPEK	0.85	0.7744		
	MPISSDLSNPR	0.20	0.1561		
	NILITGAAGFIASHVANR	0.11	0.0299		
	QGIPYEYGK	0.30	0.0336		
	SYGLPVITTR	0.09	0.0462		
	TGWIGGLLGK	0.27	0.0272		

Glyma07g14870.1	translation initiation factor 3B1	13	0.32	0.0002	protein
	Peptide Sequnce	Ratio 4(2)F	Pvalue 4(2)F		
	AGSDTEVLWNDAR	0.17	0.0337		
	DIPIEVLELENK	0.08	0.0725		
	EDGLWMPVDPETEK	0.13	0.1503		
	GSADDFAVGGAGGVTGVSWPVFK	0.57	0.3989		
	IIAFAWEPK	0.30	0.0519		
	IYSQIGVIK	0.11	0.0856		
	LGVDLSTLDLDAIR	0.06	0.0729		
	NLFSVSDCK+Carbamidomethyl(8)	0.23	0.1221		
	QANALFWSPAGR	0.38	0.1172		
	QGAAVWGGAASFNR	0.45	0.1050		
	STYTGFELFR	0.08	0.1323		
	VSLIQIPSK	0.09	0.1141		
	VVINIFDVR	0.13	0.0898		
Glyma10g35381.1	phenylalanine ammonia lyase 2	4	0.32	0.0033	secondary metabolism
	Peptide Sequnce	Ratio 4(2)F	Pvalue 4(2)F		
	ALHGGNFQGTPIGVSMDNTR	0.39	0.1447		
	EINSVNDNPLIDVSR	0.25	0.0002		
	INTLLQGYSGIR	0.00	1.0000		
	NPSLDYGFK	0.65	0.3185		
Glyma20g32135.1	phenylalanine ammonia lyase 2	4	0.32	0.0033	secondary metabolism
	Peptide Sequnce	Ratio 4(2)F	Pvalue 4(2)F		
	ALHGGNFQGTPIGVSMDNTR	0.385717657	0.144668758		
	EINSVNDNPLIDVSR	0.254223359	0.000217044		
	INTLLQGYSGIR	0	1		
	NPSLDYGFK	0.649421382	0.318474932		
Glyma05g34060.1	transducin family protein / WD 40 repeat family protein	2	0.32	0.0500	development
	Peptide Sequnce	Ratio 4(2)F	Pvalue 4(2)F		
	IDDLQWSPDGLR	0.22	0.0657		
	VLLSSSYDGLIVK	0.36	0.1324		
Glyma08g05640.1	transducin family protein / WD 40 repeat family protein	2	0.32	0.0500	development
	Peptide Sequnce	Ratio 4(2)F	Pvalue 4(2)F		
	IDDLQWSPDGLR	0.22	0.0657		
	VLLSSSYDGLIVK	0.36	0.1324		

Glyma08g20230.2	lipoxygenase 1	7	0.32	0.0428	hormone metabolism
	Peptide Sequnce	Ratio 4(2)F	Pvalue 4(2)F		
	DWVFPEQALPADLVK	1.02	0.9778		
	FMPEIGSPEYDELAK	0.03	0.1395		
	GIPNSISI	0.11	0.0765		
	IYDYDVYNDLGTPEK	0.35	0.0863		
	LDSQLYGDNTSTITK	0.41	0.4062		
	LQFNQPEFTSFDDVR	0.06	0.1615		
	SWVQEYVSFYYK	0.04	0.0920		
Glyma02g25300.1	tryptophan synthase beta subunit 2	3	0.32	0.0077	amino acid metabolism
	Peptide Sequnce	Ratio 4(2)F	Pvalue 4(2)F		
	IIAETGAGQHGVATATVCAR+Carbamidomethyl(18)	0.05	0.1332		
	INNAVAQALLAK	0.02	0.0667		
	LIGVEAAGFGLDSGK	0.36	0.0180		
Glyma18g12090.1	tryptophan synthase beta subunit 2	3	0.32	0.0077	amino acid metabolismse
	Peptide Sequnce	Ratio 4(2)F	Pvalue 4(2)F		
	IIAETGAGQHGVATATVCAR+Carbamidomethyl(18)	0.05	0.1332		
	INNAVAQALLAK	0.02	0.0667		
	LIGVEAAGFGLDSGK	0.36	0.0180		
Glyma16g34120.1	Translation initiation factor eIF3 subunit	5	0.33	0.0007	protein
	Peptide Sequnce	Ratio 4(2)F	Pvalue 4(2)F		
	DIASSVTAIANEK	0.34	0.0222		
	ESWEDEDEPAPAPAAPAVK	0.22	0.0384		
	SESDFLEYAELISHK	0.45	0.1972		
	SNWDDEDVDENDVK	0.06	0.0195		
	TVEPIKEEPLDPVAEK	0.33	0.0674		
Glyma11g09980.1	Radical SAM superfamily protein	3	0.33	0.0111	Co-factor and vitamine
	Peptide Sequnce	Ratio 4(2)F	Pvalue 4(2)F		
	APSLHEGETSVTEDYK	0.38	0.1157		
	LLTTPNNDFDADQLMFK	0.31	0.1222		
	TNFNQILEYVK	0.22	0.0181		
Glyma07g35200.1	Nuclear transport factor 2 (NTF2) family protein	4	0.33	0.0000	protein
	Peptide Sequnce	Ratio 4(2)F	Pvalue 4(2)F		
	FTQSFFLAPQDK	0.33	0.0074		
	GYFVLNDVFR	0.40	0.0243		

	ILSLDYTSFR	0.20	0.0022		
	SFASIVNALK	0.30	0.0002		
Glyma11g16220.1	beta glucosidase 42	2	0.33	0.0199	misc
	Peptide Sequnce	Ratio 4(2)F	Pvalue 4(2)F		
	FGLVYVDYK	0.32	0.0167		
	FSISWSR	0.36	0.1739		
Glyma05g33330.1	calnexin 1	8	0.33	0.0000	signalling
	Peptide Sequnce	Ratio 4(2)F	Pvalue 4(2)F		
	EFDNESPYSIMFGPDK	0.38	0.0829		
	GIWKPQDIPNPDYFEIK	0.44	0.0046		
	IADIPFLSAYK	0.13	0.0547		
	LQNGLECGGAYLK+Carbamidomethyl(7)	0.32	0.0340		
	TIPDPDDKKPEDWDER	0.23	0.0184		
	VFDLLYK	0.25	0.0371		
	VNFLSADDFEPPLIPPK	0.32	0.0085		
	YLRPQEAGWK	0.35	0.2456		
Glyma08g15680.1	rhamnose biosynthesis 1	8	0.33	0.0001	cell wall
	Peptide Sequnce	Ratio 4(2)F	Pvalue 4(2)F		
	FILLAMQGK	0.11	0.1120		
	FVENRPFNDQR	0.15	0.0046		
	GNNVYGPNQFPEK	0.85	0.7744		
	MPISSDLSNPR	0.20	0.1561		
	NILITGAAGFIASHVANR	0.11	0.0299		
	QGIPYEYGK	0.30	0.0336		
	SYGLPVITTR	0.09	0.0462		
	TGWIGGLLGK	0.27	0.0272		
Glyma08g03690.2	heat shock protein 81 2	6	0.33	0.0062	stress
	Peptide Sequnce	Ratio 4(2)F	Pvalue 4(2)F		
	APFDLFDTR	0.02	0.1254		
	AVENSPFLEK	0.86	0.7238		
	EGQNDIYYITGESK	0.00	1.0000		
	HSEFISYPISLWIEK	0.35	0.0012		
	RAPFDLFDTR	0.02	0.0870		
	SLTNDWEEHLAVK	0.28	0.1058		
Glyma09g29540.1	Translation initiation factor eIF3 subunit	5	0.33	0.0063	protein

	Peptide Sequnce	Ratio 4(2)F	Pvalue 4(2)F		
	DIASSVTAIANEK	0.34	0.0222		
	ESWEDEDEPAPAPAAPAVK	0.22	0.0384		
	SESDFLEYAELISHK	0.45	0.1972		
	TNWDDEDVDENDVK	0.26	0.4173		
	TVEPIKEEPLDPVAEK	0.33	0.0674		
Glyma15g03030.1	lipoxygenase 1	21	0.33	0.0011	hormone metabolism
	Peptide Sequnce	Ratio 4(2)F	Pvalue 4(2)F		
	CGPVQMPYTLLLPSSK+Carbamidomethyl(1)	0.45	0.0491		
	DEAFGHLK	5.96	0.4941		
	DNPNWTSDTR	0.00	1.0000		
	DTMNINGLAR	0.43	0.1887		
	EHLEPNLEGLTVDEAIQNK	0.14	0.0706		
	EMLAGVNPNLIR	0.13	0.1147		
	GIPNSISI	0.11	0.0765		
	GMAIEDPSCPHGIR+Carbamidomethyl(9)	0.49	0.1237		
	HASDEVYLGER	0.56	0.1758		
	IFFANQTYLPSETPAPLVK	0.12	0.1982		
	IYDYDVYNDLGDPDK	0.03	0.1003		
	LDSQVYGDHTSQITK	0.07	0.0422		
	LFLLDHHDPIMPYLR	0.00	1.0000		
	LPTDIISK	0.07	0.1551		
	SAWMTDEEFAR	0.05	0.1749		
	SDDTLREDPELQACWK+Carbamidomethyl(14)	0.04	0.1622		
	SKLDSQVYGDHTSQITK	0.30	0.0292		
	SSDFLTYGLK	0.39	0.2106		
	SVSLQLISATK	0.33	0.1204		
	YREEELHNLR	0.94	0.9482		
	YSVEMSAVVYK	0.33	0.2380		
Glyma17g32630.1	uridine kinase like 3	2	0.33	0.0118	nucleotide metabolism
	Peptide Sequnce	Ratio 4(2)F	Pvalue 4(2)F		
	DIGAVLDQYSK	0.37	0.0361		
	YADIIIPR	0.26	0.0439		
Glyma07g04640.1	oligouridylate binding protein 1B	3	0.34	0.0048	RNA
	Peptide Sequnce	Ratio 4(2)F	Pvalue 4(2)F		

	GFGFVSFR	0.28	0.0069		
	NQQDAQSSINDLTGK	0.36	0.0762		
	VNWAYASGQR	0.36	0.1700		
Glyma18g14670.1	putative mitochondrial RNA helicase 2	6	0.34	0.0097	RNA
	Peptide Sequnce	Ratio 4(2)F	Pvalue 4(2)F		
	IIDLLNR	0.10	0.0335		
	ILEGLSPNR	0.17	0.1455		
	LGIAPEIVDALAR	0.03	0.1512		
	NYLNNPLTIDLVGDSDQK	0.29	0.0117		
	QTLMFSATMPSWIK	0.97	0.8264		
	TLAFGIPILDR	0.11	0.2712		
Glyma11g03360.1	Glycosyl hydrolases family 32 protein	6	0.34	0.0003	major CHO metabolism
	Peptide Sequnce	Ratio 4(2)F	Pvalue 4(2)F		
	DFRDPTTAWLTSEGK	0.71	0.6666		
	DPTTAWLTSEGK	0.18	0.0048		
	ILVDHSIVESFAQGGR	0.40	0.0175		
	VLWGWIGESDSEYADVAK	0.16	0.0085		
	VSLDDDRHDYYALGTYDEK	0.55	0.7208		
	YDYGIFYASK	0.40	0.0395		
Glyma05g05540.1	Protein kinase superfamily protein	2	0.34	0.0337	protein
	Peptide Sequnce	Ratio 4(2)F	Pvalue 4(2)F		
	ITIPEIK	0.53	0.0869		
	STVGTPAYIAPEVLSR	0.06	0.0624		
Glyma05g31000.1	Protein kinase superfamily protein	2	0.34	0.0337	protein
	Peptide Sequnce	Ratio 4(2)F	Pvalue 4(2)F		
	ITIPEIK	0.53	0.0869		
	STVGTPAYIAPEVLSR	0.06	0.0624		
Glyma08g14210.1	Protein kinase superfamily protein	2	0.34	0.0337	protein
	Peptide Sequnce	Ratio 4(2)F	Pvalue 4(2)F		
	ITIPEIK	0.53	0.0869		
	STVGTPAYIAPEVLSR	0.06	0.0624		
Glyma17g15860.3	Protein kinase superfamily protein	2	0.34	0.0337	protein
	Peptide Sequnce	Ratio 4(2)F	Pvalue 4(2)F		
	ITIPEIK	0.53	0.0869		
	STVGTPAYIAPEVLSR	0.06	0.0624		

Glyma05g27360.1	ornithine delta aminotransferase	3	0.34	0.0012	amino acid metabolism
	Peptide Sequnce	Ratio 4(2)F	Pvalue 4(2)F		
	GFGPLLPGNLK	0.49	0.0945		
	IQQQYPDYVK	0.42	0.1070		
	VDFGDAEALER	0.27	0.0016		
Glyma02g45030.1	putative mitochondrial RNA helicase 2	5	0.34	0.0007	RNA
	Peptide Sequnce	Ratio 4(2)F	Pvalue 4(2)F		
	AEYAVDDFPYEEGSK	0.79	0.7069		
	IIDLLNR	0.10	0.0335		
	NYLNNPLTIDLVGDSDQK	0.29	0.0117		
	QTLMFSATMPSWIK	0.97	0.8264		
	TLAFGIPIMDK	0.27	0.0009		
Glyma09g36870.1	Transducin/WD40 repeat like superfamily protein	4	0.34	0.0045	protein
	Peptide Sequnce	Ratio 4(2)F	Pvalue 4(2)F		
	DGDLLFSCAK+Carbamidomethyl(8)	0.39	0.0505		
	IADDPTEQSGESLLLIK	0.24	0.0152		
	ILQEEIGGVK	0.31	0.0578		
	LHHFDPDYFNIK	0.44	0.3300		
Glyma05g32670.3	S adenosyl L methionine dependent methyltransferases superfamily protein	2	0.34	0.0440	stress
	Peptide Sequnce	Ratio 4(2)F	Pvalue 4(2)F		
	LPEDVEIWNEMK	0.37	0.0682		
	VTGEYLTFPGGGTQFK	0.06	0.1093		
Glyma08g00320.1	S adenosyl L methionine dependent methyltransferases superfamily protein	2	0.34	0.0440	stress
	Peptide Sequnce	Ratio 4(2)F	Pvalue 4(2)F		
	LPEDVEIWNEMK	0.37	0.0682		
	VTGEYLTFPGGGTQFK	0.06	0.1093		
Glyma11g06900.1	aminopeptidase P1	4	0.34	0.0308	protein
	Peptide Sequnce	Ratio 4(2)F	Pvalue 4(2)F		
	EAGTNFNFGDR	0.44	0.2077		
	IGEDPAVDIWMADNLPK	0.06	0.0656		
	IYLFDSGAQYLDGTTDITR	0.40	0.1453		
	LTEVTVSDQLEGFR	0.38	0.1061		
Glyma03g21690.1	rotamase FKBP 1	5	0.34	0.0181	protein
	Peptide Sequnce	Ratio 4(2)F	Pvalue 4(2)F		
	EGEGYERPNEGAIVK	0.15	0.0071		

	GTPFSFTLGQGQVIK	0.81	0.7698		
	GWDQGIITMK	0.09	0.1065		
	SDGVEFTVNDGYFCPALSK+Carbamidomethyl(14)	0.45	0.3316		
	VLDLESTNVK	0.10	0.1140		
Glyma05g33930.1	eukaryotic translation initiation factor 3C	3	0.34	0.0450	protein
	Peptide Sequnce	Ratio 4(2)F	Pvalue 4(2)F		
	IDVEFFK	0.30	0.1432		
	INDWVSLQESFDK	0.31	0.3070		
	QTFTGPPENVR	0.37	0.0363		
Glyma08g05740.1	eukaryotic translation initiation factor 3C	3	0.34	0.0450	protein
	Peptide Sequnce	Ratio 4(2)F	Pvalue 4(2)F		
	IDVEFFK	0.30	0.1432		
	INDWVSLQESFDK	0.31	0.3070		
	QTFTGPPENVR	0.37	0.0363		
Glyma08g41510.1	putative mitochondrial RNA helicase 2	5	0.34	0.0167	RNA
	Peptide Sequnce	Ratio 4(2)F	Pvalue 4(2)F		
	IDAPAGSAEMFTGMGGGR	0.18	0.4112		
	IIDLLNR	0.10	0.0335		
	ILEGLSPNR	0.17	0.1455		
	NYLNNPLTIDLVGDSDQK	0.29	0.0117		
	QTLMFSATMPSWIK	0.97	0.8264		
Glyma02g47270.1	uridine kinase like 4	4	0.34	0.0017	nucleotide metabolism
	Peptide Sequnce	Ratio 4(2)F	Pvalue 4(2)F		
	DIGAVLDQYSK	0.37	0.0361		
	IFVDTDADVR	0.38	0.0453		
	VIPGMGEFGDR	0.29	0.0585		
	YADIIIPR	0.26	0.0439		
Glyma06g10670.1	Class II DAHP synthetase family protein	2	0.35	0.0410	amino acid metabolism
	Peptide Sequnce	Ratio 4(2)F	Pvalue 4(2)F		
	AFATGGYAAMQR	0.07	0.0763		
	SLEAFPPIVFAGEAR	0.37	0.0898		
Glyma17g23720.2	RNAhelicase like 8	2	0.35	0.0044	DNA
	Peptide Sequnce	Ratio 4(2)F	Pvalue 4(2)F		
	ELALQTSQVCK+Carbamidomethyl(10)	0.39	0.0293		
	GFERPSPIQEESIPIALTGSDILAR	0.21	0.0177		

Glyma15g03050.1	lipoxygenase 1	26	0.35	0.0006	hormone metabolism
	Peptide Sequnce	Ratio 4(2)F	Pvalue 4(2)F		
	DEAFGHLK	5.96	0.4941		
	DGGDYWTSDAGPLEAFK	0.05	0.1130		
	DGGDYWTSDAGPLEAFKR	0.24	0.0743		
	DPELQAWWK	0.05	0.1426		
	DTMNINSLAR	0.05	0.2120		
	DWVFTDQALPNDLVK	0.04	0.1444		
	EHLEPNLGGLTVEQAIQNK	0.01	0.4920		
	ELVEVGHGDLK	0.07	0.2031		
	ETIAGLNPNVIK	0.06	0.0736		
	GIPNSISI	0.11	0.0765		
	GSPEYDALAK	0.02	0.1981		
	HASDEFYLGQR	0.43	0.0187		
	IFFANNTYLPSETPAPLVK	0.42	0.3124		
	IIEEFPLSSK	0.05	0.1464		
	IYDYDVYNDLGDPDK	0.03	0.1003		
	LDTQAYGDHTCIITK+Carbamidomethyl(11)	0.00	1.0000		
	LFILDHHDYLIPYLR	0.01	0.4093		
	LLFPHYR	0.29	0.1725		
	LYEGGVTLPTNFLSK	0.06	0.1284		
	MPYTLLYPSSEEGLTFR	0.06	0.1356		
	MPYTLLYPSSEEGLTFR+Oxidation(1)	2.04	0.3557		
	NFMQNEFYLK	0.07	0.1587		
	SAWMTDEEFAR	0.05	0.1749		
	SLVNADGIIEK	0.08	0.1401		
	SSDFLAYGIK	0.07	0.1157		
	YARPVLGGSALPYPR	0.02	0.4751		
Glyma11g13970.1	Leucine rich repeat (LRR) family protein	4	0.35	0.0475	not assigned
	Peptide Sequnce	Ratio 4(2)F	Pvalue 4(2)F		
	IAGTLPSNIGR	0.25	0.1001		
	IFGPIPR	0.07	0.0735		
	LSGSIPEALGR	0.43	0.1615		
	LVDLDLSNNR	0.45	0.3345		
Glyma03g01500.1	RNAhelicase like 8	8	0.35	0.0006	DNA

	Peptide Sequnce	Ratio 4(2)F	Pvalue 4(2)F		
	DCAMLVMDEADK+Carbamidomethyl(2)	0.09	0.3313		
	ELALQTSQVCK+Carbamidomethyl(10)	0.39	0.0293		
	GFERPSPIQEESIPIALTGSDILAR	0.21	0.0177		
	GITQFYAFVEER	0.32	0.0267		
	GNEFEDYFLK	0.20	0.0744		
	IQVMVTTGGTSLK	0.52	0.3020		
	LYQPVHLLVGTPGR	0.50	0.3659		
	TVQSEAVDSSSQDWK	0.26	0.0408		
Glyma07g07920.1	RNAhelicase like 8	8	0.35	0.0006	DNA
	Peptide Sequnce	Ratio 4(2)F	Pvalue 4(2)F		
	DCAMLVMDEADK+Carbamidomethyl(2)	0.09	0.3313		
	ELALQTSQVCK+Carbamidomethyl(10)	0.39	0.0293		
	GFERPSPIQEESIPIALTGSDILAR	0.21	0.0177		
	GITQFYAFVEER	0.32	0.0267		
	GNEFEDYFLK	0.20	0.0744		
	IQVMVTTGGTSLK	0.52	0.3020		
	LYQPVHLLVGTPGR	0.50	0.3659		
	TVQSEAVDSSSQDWK	0.26	0.0408		
Glyma07g07950.1	RNAhelicase like 8	8	0.35	0.0006	DNA
	Peptide Sequnce	Ratio 4(2)F	Pvalue 4(2)F		
	DCAMLVMDEADK+Carbamidomethyl(2)	0.09	0.3313		
	ELALQTSQVCK+Carbamidomethyl(10)	0.39	0.0293		
	GFERPSPIQEESIPIALTGSDILAR	0.21	0.0177		
	GITQFYAFVEER	0.32	0.0267		
	GNEFEDYFLK	0.20	0.0744		
	IQVMVTTGGTSLK	0.52	0.3020		
	LYQPVHLLVGTPGR	0.50	0.3659		
	TVQSEAVDSSSQDWK	0.26	0.0408		
Glyma14g03760.1	putative mitochondrial RNA helicase 2	6	0.35	0.0019	RNA
	Peptide Sequnce	Ratio 4(2)F	Pvalue 4(2)F		
	AEYAVDDFPYEEGSK	0.79	0.7069		
	IIDLLNR	0.10	0.0335		
	LGISEDIVSALAK	1.28	0.8072		
	NYLNNPLTIDLVGDSDQK	0.29	0.0117		

	QTLMFSATMPSWIK	0.97	0.8264		
	TLAFGIPIMDK	0.27	0.0009		
Glyma19g34900.2	ribophorin II (RPN2) family protein	4	0.35	0.0028	development
	Peptide Sequnce	Ratio 4(2)F	Pvalue 4(2)F		
	LPPLPVDPYSR	0.35	0.0245		
	VAENLELSSPVK	0.63	0.4592		
	VNTVLGSAAPPLTVK	0.39	0.1497		
	YDDGTFYFDEK	0.23	0.0047		
Glyma05g34850.1	glycosyl hydrolase 9B1	2	0.35	0.0134	misc
	Peptide Sequnce	Ratio 4(2)F	Pvalue 4(2)F		
	SILFFEGQR	0.39	0.1210		
	WGTDYLLK	0.35	0.0152		
Glyma08g04840.1	glycosyl hydrolase 9B1	2	0.35	0.0134	misc
	Peptide Sequnce	Ratio 4(2)F	Pvalue 4(2)F		
	SILFFEGQR	0.39	0.1210		
	WGTDYLLK	0.35	0.0152		
Glyma01g42450.1	hydroxymethylglutaryl CoA synthase	5	0.35	0.0011	secondary metabolism
	Peptide Sequnce	Ratio 4(2)F	Pvalue 4(2)F		
	LEVGSETVIDK	0.46	0.0702		
	LFFNDFLMNSSSADEVAK	0.23	0.8948		
	LGPFATLSGDESYQSR	0.32	0.0035		
	TFLMQIFEK	0.20	0.0722		
	VQPTTLIPK	0.25	0.0276		
Glyma09g05230.1	plasma membrane associated cation binding protein 1	2	0.35	0.0002	signalling
	Peptide Sequnce	Ratio 4(2)F	Pvalue 4(2)F		
	FLEELVK	0.28	0.0007		
	VSTFIVTEEK	0.48	0.0253		
Glyma15g16560.1	plasma membrane associated cation binding protein 1	2	0.35	0.0002	signalling
	Peptide Sequnce	Ratio 4(2)F	Pvalue 4(2)F		
	FLEELVK	0.28	0.0007		
	VSTFIVTEEK	0.48	0.0253		
Glyma06g17060.1	calnexin 1	14	0.35	0.0000	signalling
	Peptide Sequnce	Ratio 4(2)F	Pvalue 4(2)F		
	AEEEAATGSDGISGFQK	0.25	0.0423		

	ANFLSSEDFEPPLIPSK	0.43	0.0020		
	DGTVVLQFETR	0.36	0.0117		
	EFDNESPYSIMFGPDK	0.38	0.0829		
	IADIPFLSEHK	0.36	0.1467		
	IFDLIEK	0.18	0.0503		
	LQNGLECGGAYIK+Carbamidomethyl(7)	0.32	0.0340		
	LTHVYTAILKPDNELQILIDGEEK	0.23	0.0258		
	SDGHDDYGLLVSEQAR	0.39	0.0115		
	TIPDPDDKKPEDWDER	0.23	0.0184		
	VFDLLYK	0.25	0.0371		
	WIVSDKEDYNGVWK	0.70	0.5394		
	WSAPYIDNPSYK	0.34	0.0031		
	YLRPQESGWKPK	0.53	0.4879		
Glyma20g04830.1	Ribonuclease T2 family protein	3	0.35	0.0350	RNA
	Peptide Sequnce	Ratio 4(2)F	Pvalue 4(2)F		
	ITDLIPR	0.092261202	0.033837679		
	QLNEIYLCADK+Carbamidomethyl(8)	0.078526978	0.079496828		
	YASSFIECPILPSR+Carbamidomethyl(8)	0.716744257	0.42309521		
Glyma03g39480.3	Ribosomal L22e protein family	4	0.35	0.0000	protein
	Peptide Sequnce	Ratio 4(2)F	Pvalue 4(2)F		
	AGALGDSVTVTR	1.23	0.9343		
	IILTSDCNFSK+Carbamidomethyl(7)	0.12	0.0019		
	IMDIASLEK	0.17	0.0489		
	YFNIAENEGEEED	0.13	0.0003		
Glyma03g42370.1	regulatory particle triple A 1A	13	0.35	0.0000	protein
	Peptide Sequnce	Ratio 4(2)F	Pvalue 4(2)F		
	DFLDAVNK	0.21	0.0775		
	ESDTGLAAPSQWDLVSDK	0.15	0.0015		
	FDDGVGGDNEVQR	0.21	0.0110		
	IDPSVTMMTVEEKPDVTYNDVGGCK+Carbamidomethyl(24)	0.06	0.2711		
	KVEFGLPDLESR	0.28	0.1428		
	NPRPLDEDDIALLK	0.27	0.0030		
	QMMQEEQPLQVAR	0.36	0.2420		
	SVCTEAGMYAIR+Carbamidomethyl(3)	0.11	0.0234		
	TMLEIVNQLDGFDAR	1.64	0.6630		

	TYGLGPYSTSIK	0.40	0.0005		
	VEFGLPDLESR	0.11	0.0819		
	VIGSELVQK	0.02	0.0771		
	VSPTDIEEGMR	0.25	0.0895		
Glyma07g05220.1	regulatory particle triple A 1A	13	0.35	0.0000	protein
	Peptide Sequnce	Ratio 4(2)F	Pvalue 4(2)F		
	DFLDAVNK	0.21	0.0775		
	ESDTGLAAPSQWDLVSDK	0.15	0.0015		
	FDDGVGGDNEVQR	0.21	0.0110		
	IDPSVTMMTVEEKPDVTYNDVGGCK+Carbamidomethyl(24)	0.06	0.2711		
	KVEFGLPDLESR	0.28	0.1428		
	NPRPLDEDDIALLK	0.27	0.0030		
	QMMQEEQPLQVAR	0.36	0.2420		
	SVCTEAGMYAIR+Carbamidomethyl(3)	0.11	0.0234		
	TMLEIVNQLDGFDAR	1.64	0.6630		
	TYGLGPYSTSIK	0.40	0.0005		
	VEFGLPDLESR	0.11	0.0819		
	VIGSELVQK	0.02	0.0771		
	VSPTDIEEGMR	0.25	0.0895		
Glyma19g45140.1	regulatory particle triple A 1A	13	0.35	0.0000	protein
	Peptide Sequnce	Ratio 4(2)F	Pvalue 4(2)F		
	DFLDAVNK	0.21	0.0775		
	ESDTGLAAPSQWDLVSDK	0.15	0.0015		
	FDDGVGGDNEVQR	0.21	0.0110		
	IDPSVTMMTVEEKPDVTYNDVGGCK+Carbamidomethyl(24)	0.06	0.2711		
	KVEFGLPDLESR	0.28	0.1428		
	NPRPLDEDDIALLK	0.27	0.0030		
	QMMQEEQPLQVAR	0.36	0.2420		
	SVCTEAGMYAIR+Carbamidomethyl(3)	0.11	0.0234		
	TMLEIVNQLDGFDAR	1.64	0.6630		
	TYGLGPYSTSIK	0.40	0.0005		
	VEFGLPDLESR	0.11	0.0819		
	VIGSELVQK	0.02	0.0771		
	VSPTDIEEGMR	0.25	0.0895		
Glyma02g40840.1	glutamate decarboxylase	4	0.35	0.0307	amino acid metabolism

	Peptide Sequnce	Ratio 4(2)F	Pvalue 4(2)F		
	GSSQVIAQYYQLIR	0.36	0.1397		
	LIMAAINK	0.28	0.1343		
	NYVDMDEYPVTTELQNR	0.37	0.0610		
	YFEVELK	0.36	0.1831		
Glyma06g46350.1	SKU5 similar 5	7	0.35	0.0000	not assigned
	Peptide Sequnce	Ratio 4(2)F	Pvalue 4(2)F		
	DEYPIPSNAIR	0.27	0.0051		
	DQIGSYFYYPSLAFHK	0.48	0.0125		
	GSNAYTFTVDQGK	0.26	0.0017		
	ISNVGLTTSINFR	0.33	0.0935		
	SENWVHQYLGQQFYLR	0.28	0.0813		
	VYSPANSWR	0.34	0.0546		
	YAVNSVSFIPADTPLK	0.28	0.0067		
Glyma12g10420.1	SKU5 similar 5	6	0.36	0.0000	not assigned
	Peptide Sequnce	Ratio 4(2)F	Pvalue 4(2)F		
	DEYPIPSNAIR	0.27	0.0051		
	DQIGSYFYYPSLAFHK	0.48	0.0125		
	GSNAYTFTVDQGK	0.26	0.0017		
	SENWVHQYLGQQFYLR	0.28	0.0813		
	VYSPANSWR	0.34	0.0546		
	YAVNSVSFIPADTPLK	0.28	0.0067		
Glyma18g02210.1	myo inositol 1 phosphate synthase 2	11	0.36	0.0010	minor CHO metabolism
	Peptide Sequnce	Ratio 4(2)F	Pvalue 4(2)F		
	ACVGLAPENNMILEYK+Carbamidomethyl(2)	0.72	0.6006		
	AMDEYTSEIFMGGK	0.27	0.2949		
	AMLENIMR	0.28	0.0831		
	APLVPPGTPVVNALSK	0.64	0.5622		
	IQQANYFGSLTQASAIR	0.20	0.0507		
	MFIENFK	0.25	0.1690		
	NTLIGGDDFK	0.36	0.0111		
	SNVVDDMVNSNAILYEPGEHPDHVVVIK	0.45	0.3881		
	VGSFQGEEIYAPFK	0.28	0.0331		
	VVVLWTANTER	0.12	0.0136		
	YTETEIQSVYNYETTELVHENR	0.08	0.2421		

Glyma13g28840.1	Ribosomal protein S11 family protein	5	0.36	0.0006	protein
	Peptide Sequnce	Ratio 4(2)F	Pvalue 4(2)F		
	EENVTLGPAVR	0.09	0.0307		
	ELGITALHIK	0.08	0.0046		
	IEDVTPIPSDSTR	0.29	0.0377		
	IFASFNDTFIHVTDLSGR	0.74	0.1676		
	TPGPGAQSALR	2.16	0.1939		
Glyma02g24818.1	Adenylate kinase family protein	2	0.36	0.0095	nucleotide metabolism
	Peptide Sequnce	Ratio 4(2)F	Pvalue 4(2)F		
	GFILDGFPR	0.31	0.0173		
	LDEMLQNQGVK	0.46	0.0709		
Glyma08g14940.1	N.D. *	2	0.36	0.0388	not assigned
	Peptide Sequnce	Ratio 4(2)F	Pvalue 4(2)F		
	MDWVPYIPLEGR	0.39	0.1351		
	VYLFGCTEPQLVMFK+Carbamidomethyl(6)	0.33	0.0475		
Glyma03g33880.1	PHE ammonia lyase 1	5	0.36	0.0067	secondary metabolism
	Peptide Sequnce	Ratio 4(2)F	Pvalue 4(2)F		
	ALHGGNFQGTPIGVSMDNTR	0.39	0.1447		
	EINSVNDNPLIDVSR	0.25	0.0002		
	FEILEAITK	0.72	0.5169		
	INTLLQGYSGIR	0.00	1.0000		
	NPSLDYGFK	0.65	0.3185		
Glyma03g33890.1	PHE ammonia lyase 1	5	0.36	0.0067	secondary metabolism
	Peptide Sequnce	Ratio 4(2)F	Pvalue 4(2)F		
	ALHGGNFQGTPIGVSMDNTR	0.39	0.1447		
	EINSVNDNPLIDVSR	0.25	0.0002		
	FEILEAITK	0.72	0.5169		
	INTLLQGYSGIR	0.00	1.0000		
	NPSLDYGFK	0.65	0.3185		
Glyma03g01530.1	RNAhelicase like 8	7	0.36	0.0019	DNA
	Peptide Sequnce	Ratio 4(2)F	Pvalue 4(2)F		
	DCAMLVMDEADK+Carbamidomethyl(2)	0.09	0.3313		
	ELALQTSQVCK+Carbamidomethyl(10)	0.39	0.0293		
	GFERPSPIQEESIPIALTGSDILAR	0.21	0.0177		
	GITQFYAFVEER	0.32	0.0267		

	GNEFEDYFLK	0.20	0.0744		
	IQVMVTTGGTSLK	0.52	0.3020		
	LYQPVHLLVGTPGR	0.50	0.3659		
Glyma10g35024.1	Cyclophilin like peptidyl prolyl cis trans isomerase family protein	2	0.36	0.0110	cell
	Peptide Sequnce	Ratio 4(2)F	Pvalue 4(2)F		
	IIVFGQVIK	0.17	0.0190		
	VSIGILDVTLK	0.54	0.0770		
Glyma20g32530.1	Cyclophilin like peptidyl prolyl cis trans isomerase family protein	2	0.36	0.0110	cell
	Peptide Sequnce	Ratio 4(2)F	Pvalue 4(2)F		
	IIVFGQVIK	0.17	0.0190		
	VSIGILDVTLK	0.54	0.0770		
Glyma19g03730.1	HXXXD type acyl transferase family protein	4	0.36	0.0001	secondary metabolism
	Peptide Sequnce	Ratio 4(2)F	Pvalue 4(2)F		
	GLFELTPLDIK	0.08	0.0123		
	LFSVAGSPR	0.73	0.2860		
	SLNVWDSLGGSQTDLVK	0.12	0.0002		
	VPFIFSVDCR+Carbamidomethyl(9)	0.39	0.1339		
Glyma08g22380.1	Protein of unknown function DUF642	2	0.37	0.0322	not assigned
	Peptide Sequnce	Ratio 4(2)F	Pvalue 4(2)F		
	VEIVIHNPGVDEDPACGPLIDSVALK+Carbamidomethyl(16)	0.48	0.2482		
	YIDSDHFAVPEGK	0.36	0.0207		
Glyma16g21111.1	TUDOR SN protein 2	2	0.37	0.0214	RNA
	Peptide Sequnce	Ratio 4(2)F	Pvalue 4(2)F		
	IPDFHLLEQAEQSAK	0.25	0.0623		
	SNYYDALLTAESR	0.40	0.0508		
Glyma05g04290.1	Glycosyl hydrolases family 32 protein	13	0.37	0.0000	major CHO metabolism
	Peptide Sequnce	Ratio 4(2)F	Pvalue 4(2)F		
	AFPWDNSMLSWQR	0.52	0.1656		
	DFRDPTTAWLTSEGK	0.71	0.6666		
	DPTTAWLTSEGK	0.18	0.0048		
	ENENGLDTSINGAEVK	0.36	0.5229		
	GWASVQSIPR	0.42	0.0234		
	ILVDHSIVESFAQGGR	0.40	0.0175		
	NVLFTPDDAK	0.32	0.0389		
	NWMNDPNGPMYYK	0.27	0.1644		

	QIVGSAVPVLK	0.30	0.0457		
	TGIALVYDTEDFK	0.38	0.0145		
	VLWGWIGESDSEYADVAK	0.16	0.0085		
	YDYGIFYASK	0.40	0.0395		
	YPGNPVLVPPPGIGAK	0.16	0.0010		
Glyma13g04590.1	Peroxidase superfamily protein	4	0.37	0.0041	misc
	Peptide Sequnce	Ratio 4(2)F	Pvalue 4(2)F		
	DTCPQFSQIIR+Carbamidomethyl(3)	0.37	0.0827		
	FDNAYFQNLPK	0.30	0.0301		
	FFQVFAR	0.32	0.1679		
	LSLLNVQTGR	0.43	0.0312		
Glyma15g01370.1	Protein of unknown function DUF642	7	0.38	0.0000	not assigned
	Peptide Sequnce	Ratio 4(2)F	Pvalue 4(2)F		
	EEDPACGPLIDSVALK+Carbamidomethyl(6)	0.26	0.0034		
	ESALAQVVITTIGK	0.51	0.0299		
	FLSTFYTMK	0.17	0.1056		
	GSFYSITFSAAR	0.26	0.0117		
	QGDMLLVVPEGDYAVR	0.30	0.0761		
	SDNSGSLCGPVIDDVK+Carbamidomethyl(8)	0.46	0.0169		
	YIDSDHFAVPEGK	0.36	0.0207		
Glyma19g01620.1	Peroxidase superfamily protein	3	0.38	0.0076	misc
	Peptide Sequnce	Ratio 4(2)F	Pvalue 4(2)F		
	FDNAYFQNLPK	0.30	0.0301		
	FFQVFAR	0.32	0.1679		
	LSLLNVQTGR	0.43	0.0312		
Glyma16g10730.1	rotamase FKBP 1	5	0.38	0.0288	protein
	Peptide Sequnce	Ratio 4(2)F	Pvalue 4(2)F		
	EGEGYEHPNEGAIVK	0.47	0.5828		
	GTPFSFTLGQGQVIK	0.81	0.7698		
	GWDQGIITMK	0.09	0.1065		
	VLDLESTNVK	0.10	0.1140		
	YEAHLEDGTLVAK	0.02	0.0081		
Glyma13g01260.1	xylose isomerase family protein	3	0.38	0.0158	minor CHO metabolism
	Peptide Sequnce	Ratio 4(2)F	Pvalue 4(2)F		
	ANFEFISK	0.17	0.0744		

	LGVDFWCFHDR+Carbamidomethyl(7)	0.54	0.1740		
	WYNAEEEILGK	0.16	0.0309		
Glyma13g42330.1	lipoxygenase 1	31	0.38	0.0000	hormone metabolism
	Peptide Sequnce	Ratio 4(2)F	Pvalue 4(2)F		
	ALVNADGIIEK	0.08	0.1396		
	DEAFGHLK	5.96	0.4941		
	DGGDYWTSDAGPLEAFK	0.05	0.1130		
	DGGDYWTSDAGPLEAFKR	0.24	0.0743		
	DPELQAWWK	0.05	0.1426		
	DTMNINSLAR	0.05	0.2120		
	DWVFTDQALPNDLVK	0.04	0.1444		
	EHLEPNLGGLTVEQAIQNK	0.01	0.4920		
	ELVEVGHGDLK	0.07	0.2031		
	ETIAGLNPNVIK	0.06	0.0736		
	GIPNSISI	0.11	0.0765		
	GSPEYDALAK	0.02	0.1981		
	HASDEFYLGQR	0.43	0.0187		
	IAPIPVIK	0.05	0.1572		
	IFFANNTYLPSETPAPLLK	0.11	0.1055		
	IIEEFPLSSK	0.05	0.1464		
	ISIQLISATK	0.07	0.1370		
	IYDYDVYNDLGNPDSGDK	0.65	0.0966		
	LDTQAYGDHTCIIAK+Carbamidomethyl(11)	0.13	0.0076		
	LFILDHHDYLIPYLR	0.01	0.4093		
	LLFPHYR	0.29	0.1725		
	LYEGGVTLPTNFLSK	0.06	0.1284		
	MPYTLLYPSSEEGLTFR	0.06	0.1356		
	MPYTLLYPSSEEGLTFR+Oxidation(1)	2.04	0.3557		
	NFMQNEFYLK	0.07	0.1587		
	QLSVVHPIYK	0.08	0.0770		
	SAWMTDEEFAR	0.05	0.1749		
	SLILEDIPNHGTIHFVCNSWVYNSK+Carbamidomethyl(17)	0.09	0.1867		
	SSDFLAYGIK	0.07	0.1157		
	SWVQEYVSFYYK	0.04	0.0920		
	YARPVLGGSALPYPR	0.02	0.4751		

Glyma05g22060.1	Subtilase family protein	7	0.38	0.0000	protein
	Peptide Sequnce	Ratio 4(2)F	Pvalue 4(2)F		
	AVGPTGLPVDNR	0.21	0.0255		
	GGCFSSDILAAIER+Carbamidomethyl(3)	0.29	0.0296		
	HVVGTPISINWG	0.17	0.1144		
	ISVEPQVLSFK	0.23	0.0264		
	SALMTTAYTVYK	0.52	0.0064		
	SFDDTGLGPVPSTWK	0.31	0.0009		
	VDFNIISGTSMSCPHVSGLAALIK+Carbamidomethyl(13)	0.90	0.5889		
Glyma09g41240.4	Protein kinase superfamily protein	2	0.38	0.0278	protein
	Peptide Sequnce	Ratio 4(2)F	Pvalue 4(2)F		
	LADFGLAR	0.32	0.0482		
	LEKELMGK+Oxidation(6)	0.42	0.0895		
Glyma20g37700.1	seryl tRNA synthetase / serine tRNA ligase	3	0.38	0.0316	protein
	Peptide Sequnce	Ratio 4(2)F	Pvalue 4(2)F		
	CAQLAQFDEELYK+Carbamidomethyl(1)	0.93	0.9124		
	ELVSCSNCTDYQAR+Carbamidomethyl(5)Carbamidomethyl(8)	0.09	0.0083		
	MLDINLFR	0.09	0.1318		
Glyma19g25360.1	uridine kinase/uracil phosphoribosyltransferase 1	2	0.38	0.0108	nucleotide metabolism
	Peptide Sequnce	Ratio 4(2)F	Pvalue 4(2)F		
	IFVDTDADVR	0.38	0.0453		
	LCGVSIVR+Carbamidomethyl(2)	0.39	0.0316		
Glyma19g03520.1	Ribosomal protein S19e family protein	3	0.38	0.0114	protein
	Peptide Sequnce	Ratio 4(2)F	Pvalue 4(2)F		
	ELAPYDPDWYYIR	0.47	0.0423		
	GGLGVGAFQR	0.19	0.0548		
	MELPEWTDIVK	0.24	0.1136		
Glyma18g49189.1	Hyaluronan / mRNA binding family	3	0.38	0.0037	RNA
	Peptide Sequnce	Ratio 4(2)F	Pvalue 4(2)F		
	EFASMQPLSNK	0.21	0.0413		
	EMTLEEYEK	0.14	0.0142		
	ENDEIFIK	0.60	0.1096		
Glyma10g11670.3	sterol methyltransferase 1	4	0.39	0.0014	hormone metabolism
	Peptide Sequnce	Ratio 4(2)F	Pvalue 4(2)F		
	AEIEIGDGLPDIR	0.36	0.0292		

	FSSTSITGLNNNEYQITR	0.37	0.0238		
	QAGFEVIWEK	0.34	0.0863		
	VLDVGCGIGGPLR+Carbamidomethyl(6)	0.45	0.0529		
Glyma15g10210.1	Ribosomal protein S11 family protein	4	0.39	0.0090	protein
	Peptide Sequnce	Ratio 4(2)F	Pvalue 4(2)F		
	EENVTLGPAVR	0.09	0.0307		
	IEDVTPIPSDSTR	0.29	0.0377		
	IFASFNDTFIHVTDLSGR	0.74	0.1676		
	TPGPGAQSALR	2.16	0.1939		
Glyma01g01070.1	hexokinase 2	3	0.39	0.0026	major CHO metabolism
	Peptide Sequnce	Ratio 4(2)F	Pvalue 4(2)F		
	ELGFTFSFPVR	0.55	0.0388		
	LSAAGIFGILK	0.18	0.0027		
	MLITYVDNLPSGDEK	0.51	0.3983		
Glyma11g11410.1	subtilisin like serine protease 2	4	0.39	0.0446	protein
	Peptide Sequnce	Ratio 4(2)F	Pvalue 4(2)F		
	MYQLVYPGK	0.49	0.1800		
	SAMMTTATVLDNR	0.16	0.1507		
	SFSDLNLGPIPR	0.42	0.0609		
	SPQFLGLR	0.06	0.2194		
Glyma20g21440.2	HAD superfamily subfamily IIIB acid phosphatase	2	0.39	0.0296	misc
	Peptide Sequnce	Ratio 4(2)F	Pvalue 4(2)F		
	DFQVVPEECIEYIGK+Carbamidomethyl(9)	0.35	0.0352		
	VGYYGWTK	0.45	0.1322		
Glyma13g43970.1	Protein of unknown function DUF642	8	0.39	0.0000	not assigned
	Peptide Sequnce	Ratio 4(2)F	Pvalue 4(2)F		
	ADFPEAEIVIHNPGKEEDPACGPLIDSVALK+Carbamidomethyl(21)	0.42	0.0054		
	EEDPACGPLIDSVALK+Carbamidomethyl(6)	0.26	0.0034		
	ESALAQVVITTIGK	0.51	0.0299		
	FLSTFYTMK	0.17	0.1056		
	GSFYSITFSAAR	0.26	0.0117		
	QGDMLLVVPEGDYAVR	0.30	0.0761		
	SDNSGSLCGPVIDDVK+Carbamidomethyl(8)	0.46	0.0169		
	YIDSDHFAVPEGK	0.36	0.0207		
Glyma02g42210.1	eukaryotic translation initiation factor 2	2	0.39	0.0366	protein

	Peptide Sequnce	Ratio 4(2)F	Pvalue 4(2)F		
	FISDAVGSIPK	0.43	0.2425		
	QIAAQFQEIPLDLR	0.37	0.0246		
Glyma01g24650.1	Metal dependent protein hydrolase	3	0.39	0.0009	not assigned
	Peptide Sequnce	Ratio 4(2)F	Pvalue 4(2)F		
	AMALAGSEFLDSVR	0.35	0.1207		
	LNLDWTDPDQSPEK	0.44	0.0118		
	VQAVAVSPDSFQSR	0.33	0.0085		
Glyma08g18240.1	eukaryotic translation initiation factor 2 gamma subunit	10	0.39	0.0153	protein
	Peptide Sequnce	Ratio 4(2)F	Pvalue 4(2)F		
	EDSPMCDVPGFENSK+Carbamidomethyl(6)	0.50	0.4041		
	FIQGTVADGAPVVPISAQLK	0.24	0.0661		
	GEMLMLNIGSMSTGAR	0.73	0.7702		
	LHPLSPEVISR	0.42	0.0915		
	LQHIIILQNK	0.38	0.2156		
	LQLTSPVCTSK+Carbamidomethyl(8)	0.30	0.0366		
	NFVSPPNMIVIR	0.33	0.1813		
	SFDVNKPGYEVDEIK	1.02	0.9845		
	VNQFIEVRPGIVVK	0.43	0.2170		
	YNIDVVCEYIVK+Carbamidomethyl(7)	0.21	0.0263		
Glyma15g40750.1	eukaryotic translation initiation factor 2 gamma subunit	10	0.39	0.0153	protein
	Peptide Sequnce	Ratio 4(2)F	Pvalue 4(2)F		
	EDSPMCDVPGFENSK+Carbamidomethyl(6)	0.50	0.4041		
	FIQGTVADGAPVVPISAQLK	0.24	0.0661		
	GEMLMLNIGSMSTGAR	0.73	0.7702		
	LHPLSPEVISR	0.42	0.0915		
	LQHIIILQNK	0.38	0.2156		
	LQLTSPVCTSK+Carbamidomethyl(8)	0.30	0.0366		
	NFVSPPNMIVIR	0.33	0.1813		
	SFDVNKPGYEVDEIK	1.02	0.9845		
	VNQFIEVRPGIVVK	0.43	0.2170		
	YNIDVVCEYIVK+Carbamidomethyl(7)	0.21	0.0263		
Glyma09g40580.1	NAD(P) binding Rossmann fold superfamily protein	4	0.39	0.0299	secondary metabolism
	Peptide Sequnce	Ratio 4(2)F	Pvalue 4(2)F		
	AIDGALGILK	0.19	0.1595		

	DVSFLTNLPGASEK	0.30	0.0258		
	LVDAGFEFK	0.55	0.0730		
	YHMVHVDDVAR	0.65	0.6695		
Glyma18g45260.2	NAD(P) binding Rossmann fold superfamily protein	4	0.39	0.0299	secondary metabolism
	Peptide Sequnce	Ratio 4(2)F	Pvalue 4(2)F		
	AIDGALGILK	0.19	0.1595		
	DVSFLTNLPGASEK	0.30	0.0258		
	LVDAGFEFK	0.55	0.0730		
	YHMVHVDDVAR	0.65	0.6695		
Glyma10g07340.1	L O methylthreonine resistant 1	6	0.39	0.0003	amino acid metabolism
	Peptide Sequnce	Ratio 4(2)F	Pvalue 4(2)F		
	FLDPFSPR	0.26	0.1102		
	GVICSSAGNHAQGVALAAK+Carbamidomethyl(4)	0.39	0.0118		
	VILDQVGGFADGVAVK	0.28	0.0054		
	VVNNDDDFQLLMH	0.56	0.3854		
	VVTELANVGR	0.39	0.0834		
	VYDVAIESPLQLAPK	0.41	0.0557		
Glyma01g37810.1	NmrA like negative transcriptional regulator family protein	6	0.39	0.0355	secondary metabolism
	Peptide Sequnce	Ratio 4(2)F	Pvalue 4(2)F		
	AGNPTFILVR	0.41	0.1560		
	FFPSEFGLDVDR	0.46	0.1677		
	GDAVYEIDPAK	0.34	0.2091		
	ILVLGPTGAIGR	0.46	0.2032		
	IYVSEEEVLK	0.31	0.0589		
	LLIEDQVK	0.47	0.2321		
Glyma12g01160.1	sterol methyltransferase 1	3	0.39	0.0056	hormone metabolism
	Peptide Sequnce	Ratio 4(2)F	Pvalue 4(2)F		
	FSSTSITGLNNNEYQITR	0.37	0.0238		
	QAGFEVIWEK	0.34	0.0863		
	VLDVGCGIGGPLR+Carbamidomethyl(6)	0.45	0.0529		
Glyma19g36620.1	PHE ammonia lyase 1	6	0.39	0.0066	secondary metabolism
	Peptide Sequnce	Ratio 4(2)F	Pvalue 4(2)F		
	ALHGGNFQGTPIGVSMDNTR	0.39	0.1447		
	EINSVNDNPLIDVSR	0.25	0.0002		
	FEILEAITK	0.72	0.5169		

	GSDPLNWGAAAEAMK	0.59	0.2085		
	INTLLQGYSGIR	0.00	1.0000		
	NPSLDYGFK	0.65	0.3185		
Glyma04g42460.1	2 oxoglutarate (2OG) and Fe(II) dependent oxygenase superfamily protein	4	0.39	0.0008	hormone metabolism
	Peptide Sequnce	Ratio 4(2)F	Pvalue 4(2)F		
	ATICPAPQLVEK+Carbamidomethyl(4)	0.27	0.0805		
	FVFGDYMSVYAEQK	0.47	0.0408		
	SIASFYNPSFK	0.34	0.0033		
	VGGLQMLK	0.29	0.1364		
Glyma05g33860.1	Pathogenesis related thaumatin superfamily protein	2	0.4	0.0163	stress
	Peptide Sequnce	Ratio 4(2)F	Pvalue 4(2)F		
	AYSLQLPALWSGR	0.35	0.0037		
	AYSYAYDDPTSIATCTK+Carbamidomethyl(15)	0.70	0.6149		
Glyma08g05820.1	Pathogenesis related thaumatin superfamily protein	2	0.4	0.0163	stress
	Peptide Sequnce	Ratio 4(2)F	Pvalue 4(2)F		
	AYSLQLPALWSGR	0.35	0.0037		
	AYSYAYDDPTSIATCTK+Carbamidomethyl(15)	0.70	0.6149		
Glyma01g41310.1	glutamate dehydrogenase 2	2	0.4	0.0303	N-metabolism
	Peptide Sequnce	Ratio 4(2)F	Pvalue 4(2)F		
	LENSLLIPFR	0.30	0.0401		
	TAVADIPYGGAK	0.46	0.1190		
Glyma05g10100.1	peptidyl prolyl cis trans isomerase / cyclophilin 40 (CYP40) / rotamase	2	0.4	0.0017	cell
	Peptide Sequnce	Ratio 4(2)F	Pvalue 4(2)F		
	IVVELFHDVVPK	0.08	0.0007		
	LGDLQGALLDSDFAMHDGDNAK	0.47	0.2463		
Glyma17g20430.1	peptidyl prolyl cis trans isomerase / cyclophilin 40 (CYP40) / rotamase	2	0.4	0.0017	cell
	Peptide Sequnce	Ratio 4(2)F	Pvalue 4(2)F		
	IVVELFHDVVPK	0.08	0.0007		
	LGDLQGALLDSDFAMHDGDNAK	0.47	0.2463		
Glyma13g21230.1	L O methylthreonine resistant 1	5	0.4	0.0004	amino acid metabolism
	Peptide Sequnce	Ratio 4(2)F	Pvalue 4(2)F		
	GVICSSAGNHAQGVALAAK+Carbamidomethyl(4)	0.39	0.0118		
	VILDQVGGFADGVAVK	0.28	0.0054		
	VVNNDDDFQLLMH	0.56	0.3854		
	VVTELANVGR	0.39	0.0834		

	VYDVAIESPLQLAPK	0.41	0.0557		
Glyma05g21260.1	lipoxygenase 1	5	0.4	0.0061	hormone metabolism
	Peptide Sequnce	Ratio 4(2)F	Pvalue 4(2)F		
	DGGDYWTSDAGPLEAFK	0.05	0.1130		
	DGGDYWTSDAGPLEAFKR	0.24	0.0743		
	DPELQAWWK	0.05	0.1426		
	ELVEVGHGDLK	0.07	0.2031		
	HASDEFYLGQR	0.43	0.0187		
Glyma09g06750.1	RNA binding KH domain containing protein	2	0.4	0.0286	RNA
	Peptide Sequnce	Ratio 4(2)F	Pvalue 4(2)F		
	LLVASTQAINLIGK	0.31	0.0890		
	WPGWPGDCVFR+Carbamidomethyl(8)	0.41	0.0502		
Glyma15g18002.1	RNA binding KH domain containing protein	2	0.4	0.0286	RNA
	Peptide Sequnce	Ratio 4(2)F	Pvalue 4(2)F		
	LLVASTQAINLIGK	0.31	0.0890		
	WPGWPGDCVFR+Carbamidomethyl(8)	0.41	0.0502		
Glyma14g39170.1	glutamate decarboxylase	5	0.4	0.0316	amino acid metabolism
	Peptide Sequnce	Ratio 4(2)F	Pvalue 4(2)F		
	EAAYQIINDELMLDGNPR	0.49	0.2437		
	GSSQVIAQYYQLIR	0.36	0.1397		
	LIMAAINK	0.28	0.1343		
	NYVDMDEYPVTTELQNR	0.37	0.0610		
	YFEVELK	0.36	0.1831		
Glyma04g38630.1	Glycosyl hydrolases family 31 protein	11	0.4	0.0001	misc
	Peptide Sequnce	Ratio 4(2)F	Pvalue 4(2)F		
	APGSSSLIATDVTISHGDLTAK	0.43	0.1015		
	EPWLFGER	0.15	0.0189		
	FEVPDVIVSEFPSTK	0.40	0.0298		
	HDPFELFIR	0.43	0.2082		
	HMVTIVDPHIK	0.34	0.1289		
	IDEDPSLSPPK	0.13	0.0181		
	LEVTEESIPAFQR	0.57	0.4212		
	LTSIDLAPASSSK	0.09	0.0207		
	SEDDNWEEQFR	0.31	0.1150		
	VISLNSHDLFDFEQLK	0.66	0.1903		

	YGAVWTGDNTADWDHLR	0.41	0.1659		
Glyma02g41220.1	high mobility group A	2	0.4	0.0003	DNA
	Peptide Sequnce	Ratio 4(2)F	Pvalue 4(2)F		
	VKPQLTEVSVES	0.42	0.0063		
	YIETTYGELPDATVLGSHLNK	0.38	0.0045		
Glyma02g41230.2	high mobility group A	2	0.4	0.0003	DNA
	Peptide Sequnce	Ratio 4(2)F	Pvalue 4(2)F		
	VKPQLTEVSVES	0.42	0.0063		
	YIETTYGELPDATVLGSHLNK	0.38	0.0045		
Glyma09g40590.1	NAD(P) binding Rossmann fold superfamily protein	3	0.4	0.0378	secondary metabolism
	Peptide Sequnce	Ratio 4(2)F	Pvalue 4(2)F		
	DVSFLTNLPGASEK	0.30	0.0258		
	LVDAGFEFK	0.55	0.0730		
	YHMVHVDDVAR	0.65	0.6695		
Glyma05g04822.1	rhamnose biosynthesis 1	5	0.41	0.0053	cell wall
	Peptide Sequnce	Ratio 4(2)F	Pvalue 4(2)F		
	FILLAMQGK	0.11	0.1120		
	GNNVYGPNQFPEK	0.85	0.7744		
	QGIPYEYGK	0.30	0.0336		
	SYGLPVITTR	0.09	0.0462		
	TGWIGGLLGK	0.27	0.0272		
Glyma12g36350.1	Nucleoporin autopeptidase	2	0.41	0.0143	protein
	Peptide Sequnce	Ratio 4(2)F	Pvalue 4(2)F		
	LESISAMPIYK	0.77	0.4807		
	WEDYQLGDK	0.28	0.0041		
Glyma13g33910.1	Nucleoporin autopeptidase	2	0.41	0.0143	protein
	Peptide Sequnce	Ratio 4(2)F	Pvalue 4(2)F		
	LESISAMPIYK	0.77	0.4807		
	WEDYQLGDK	0.28	0.0041		
Glyma20g36060.1	Nucleoporin autopeptidase	2	0.41	0.0143	protein
	Peptide Sequnce	Ratio 4(2)F	Pvalue 4(2)F		
	LESISAMPIYK	0.77	0.4807		
	WEDYQLGDK	0.28	0.0041		
Glyma02g36680.1	MIF4G domain containing protein / MA3 domain containing protein	16	0.41	0.0000	protein

	Peptide Sequnce	Ratio 4(2)F	Pvalue 4(2)F		
	APAYYPEFVK	0.27	0.0871		
	APNNFGEIIGK	0.28	0.1486		
	AQISSNQGGGPTPTLVK	0.46	0.0420		
	AVSAIPSEKPQPPAAK	0.28	0.0258		
	EGVEILDDVLK	0.26	0.0484		
	ELSTNPQLAPR	0.26	0.0040		
	GQLIDSGITSADILK	0.77	0.1342		
	GVISLIFDK	0.43	0.1178		
	INDMYFIR	0.24	0.1337		
	LLDEALQCVEELK+Carbamidomethyl(8)	0.50	0.3852		
	LLPQGSGGIISGR	0.21	0.0137		
	LPPFPSDEPGGK	0.26	0.0928		
	LVLAGGLDFK	0.30	0.0796		
	NLGLRPGATASMR	0.53	0.1231		
	TVSLLEEYFSVR	0.24	0.2418		
	VLLNICQEAFEGADK+Carbamidomethyl(6)	0.43	0.0502		
Glyma10g05130.1	pyrophosphorylase 4	4	0.41	0.0053	nucleotide metabolism
	Peptide Sequnce	Ratio 4(2)F	Pvalue 4(2)F		
	EVAVNDFLPASAAYEAIK	0.27	0.0542		
	IIAVCADDPEYR+Carbamidomethyl(5)	0.23	0.0290		
	ILYSSVVYPHNYGFIPR	0.55	0.2037		
	TGLIMVDR	0.38	0.0572		
Glyma13g19500.1	pyrophosphorylase 4	4	0.41	0.0053	nucleotide metabolism
	Peptide Sequnce	Ratio 4(2)F	Pvalue 4(2)F		
	EVAVNDFLPASAAYEAIK	0.27	0.0542		
	IIAVCADDPEYR+Carbamidomethyl(5)	0.23	0.0290		
	ILYSSVVYPHNYGFIPR	0.55	0.2037		
	TGLIMVDR	0.38	0.0572		
Glyma07g00860.2	lipoxygenase 1	3	0.41	0.0190	hormone metabolism
	Peptide Sequnce	Ratio 4(2)F	Pvalue 4(2)F		
	EMIAGVNPCVIR+Carbamidomethyl(9)	0.35	0.1558		
	HASDEFYLGQR	0.43	0.0187		
	SAWMTDEEFAR	0.05	0.1749		
Glyma17g17850.1	Subtilase family protein	9	0.41	0.0000	protein

	Peptide Sequnce	Ratio 4(2)F	Pvalue 4(2)F		
	AVGPTGLPVDNR	0.21	0.0255		
	GGCFSSDILAAIER+Carbamidomethyl(3)	0.29	0.0296		
	HLVGSPISVNWG	0.43	0.0061		
	ISVEPQVLSFK	0.23	0.0264		
	SALMTTAYTVYK	0.52	0.0064		
	SFDDTGLGPVPSTWK	0.31	0.0009		
	TFTVTFSSSGSPQHTENAFGR	0.87	0.7293		
	TPLFLGLDK	0.36	0.0083		
	VDFNIISGTSMSCPHVSGLAALIK+Carbamidomethyl(13)	0.90	0.5889		
Glyma04g05460.1	N.D. *	2	0.41	0.0312	not assigned
	Peptide Sequnce	Ratio 4(2)F	Pvalue 4(2)F		
	AVLSGSQDLDTDSLTSLR	0.51	0.1728		
	GFPVEIQMDTK	0.03	0.0287		
Glyma06g05490.1	N.D. *	2	0.41	0.0312	not assigned
	Peptide Sequnce	Ratio 4(2)F	Pvalue 4(2)F		
	AVLSGSQDLDTDSLTSLR	0.51	0.1728		
	GFPVEIQMDTK	0.03	0.0287		
Glyma12g01170.1	sterol methyltransferase 1	2	0.41	0.0116	hormone metabolism
	Peptide Sequnce	Ratio 4(2)F	Pvalue 4(2)F		
	AEIEIGDGLPDIR	0.36	0.0292		
	VLDVGCGIGGPLR+Carbamidomethyl(6)	0.45	0.0529		
Glyma05g03150.1	homolog of nucleolar protein NOP56	2	0.41	0.0490	RNA
	Peptide Sequnce	Ratio 4(2)F	Pvalue 4(2)F		
	DTEMETEAPAEVSGK	0.42	0.0416		
	IVNDNYLYAK	0.04	0.2046		
Glyma13g44170.3	phospholipase D alpha 1	8	0.41	0.0010	lipid metabolism
	Peptide Sequnce	Ratio 4(2)F	Pvalue 4(2)F		
	FPGVPYTFFSQR	0.12	0.0610		
	IVSFVGGIDLCDGR+Carbamidomethyl(11)	0.13	0.0406		
	LYATIDLEK	0.21	0.1080		
	NYLTFFCLGNR+Carbamidomethyl(7)	0.58	0.2749		
	SIDGGAAFGFPETPEDAAR	0.13	0.0280		
	VLMLVWDDR	0.14	0.1651		
	VSLYQDAHVPDNFVPK	0.22	0.0501		

	YWDIYSSESLEHDLPGHLLR	0.26	0.1777		
Glyma03g37320.1	Protein of unknown function DUF538	2	0.41	0.0080	not assigned
	Peptide Sequnce	Ratio 4(2)F	Pvalue 4(2)F		
	IEVLWLK	0.15	0.0252		
	YDLPVGLLPK	0.44	0.0404		
Glyma19g39930.1	Protein of unknown function DUF538	2	0.41	0.0080	not assigned
	Peptide Sequnce	Ratio 4(2)F	Pvalue 4(2)F		
	IEVLWLK	0.15	0.0252		
	YDLPVGLLPK	0.44	0.0404		
Glyma03g33390.1	adenosine kinase	4	0.41	0.0018	nucleotide metabolism
	Peptide Sequnce	Ratio 4(2)F	Pvalue 4(2)F		
	GESGFILDGFPR	0.18	0.0310		
	LCNLLGVPHIATGDLVR+Carbamidomethyl(2)	0.13	0.0422		
	LLQALNLDDYEEK	0.49	0.0736		
	NVQWVFLGCPGVGK+Carbamidomethyl(9)	0.24	0.0454		
Glyma19g36120.1	adenosine kinase	4	0.41	0.0018	nucleotide metabolism
	Peptide Sequnce	Ratio 4(2)F	Pvalue 4(2)F		
	GESGFILDGFPR	0.18	0.0310		
	LCNLLGVPHIATGDLVR+Carbamidomethyl(2)	0.13	0.0422		
	LLQALNLDDYEEK	0.49	0.0736		
	NVQWVFLGCPGVGK+Carbamidomethyl(9)	0.24	0.0454		
Glyma01g02300.1	Mitochondrial substrate carrier family protein	4	0.41	0.0351	transport
	Peptide Sequnce	Ratio 4(2)F	Pvalue 4(2)F		
	DLTAGTVGGAAQLIVGHPFDTIK	0.05	0.0061		
	EVPGNAAMFGVYEALK	0.75	0.7467		
	FSGSIDAFR	0.62	0.1201		
	LLAGGTDTSGLGR	0.67	0.4654		
Glyma15g15010.1	ATP citrate lyase subunit B 2	6	0.41	0.0075	TCA / org transformation
	Peptide Sequnce	Ratio 4(2)F	Pvalue 4(2)F		
	FGGAIDDAAR	0.05	0.1337		
	LYRPGSVGFVSK	0.33	0.0176		
	MLDFDFLCGR+Carbamidomethyl(8)	0.03	0.1770		
	MMVVLGELGGR	0.27	0.1919		
	SGGMSNELYNTIAR	0.28	0.0895		
	VVAIIAEGVPESDTK	0.86	0.1838		

Glyma09g39710.2	RNAhelicase like 8	4	0.42	0.0339	DNA
	Peptide Sequnce	Ratio 4(2)F	Pvalue 4(2)F		
	AIAGIITER	0.99	0.9894		
	GADGSWLWCTTDDTVYDAVK+Carbamidomethyl(9)	0.94	0.9212		
	IEEHGFESTTISDILNDK	1.76	0.0987		
	SMTQNNVGALVVVK	0.89	0.7394		
Glyma04g38000.1	calnexin 1	13	0.42	0.0000	signalling
	Peptide Sequnce	Ratio 4(2)F	Pvalue 4(2)F		
	AEDEAATGSDGIAGFQK	0.11	0.1506		
	ANFLSSEDFEPPLIPSK	0.43	0.0020		
	DGTVVLQFETR	0.36	0.0117		
	EFDNESPYSIMFGPDK	0.38	0.0829		
	IADIPFLSDHK	1.44	0.5977		
	LQNGLECGGAYIK+Carbamidomethyl(7)	0.32	0.0340		
	LTHVYTAILKPDNELQILIDGEEK	0.23	0.0258		
	SDGHDDYGLLVSEQAR	0.39	0.0115		
	TIPDPDDKKPEDWDER	0.23	0.0184		
	VFDLLYK	0.25	0.0371		
	WIVSDKEDYNGVWK	0.70	0.5394		
	WSAPYIDNPNYK	0.54	0.0368		
	YLRPQESGWKPK	0.53	0.4879		
Glyma04g11870.1	lipoxygenase 1	3	0.42	0.0195	hormone metabolism
	Peptide Sequnce	Ratio 4(2)F	Pvalue 4(2)F		
	DPELQAWWK	0.05	0.1426		
	GSPEYDALAK	0.02	0.1981		
	HASDEFYLGQR	0.43	0.0187		
Glyma04g11640.1	lipoxygenase 1	3	0.42	0.0199	hormone metabolism
	Peptide Sequnce	Ratio 4(2)F	Pvalue 4(2)F		
	DPELQAWWK	0.05	0.1426		
	ELVEVGHGDLK	0.07	0.2031		
	HASDEFYLGQR	0.43	0.0187		
Glyma14g01480.2	uridine kinase like 4	4	0.42	0.0190	nucleotide metabolism
	Peptide Sequnce	Ratio 4(2)F	Pvalue 4(2)F		
	AGTEQPTTSATDMYK	0.93	0.9075		
	IFVDTDADVR	0.38	0.0453		

	VIPGMGEFGDR	0.29	0.0585		
	YADIIIPR	0.26	0.0439		
Glyma07g01480.1	ubiquitin specific protease 6	4	0.42	0.0024	protein
	Peptide Sequnce	Ratio 4(2)F	Pvalue 4(2)F		
	ALFGIELISR	0.12	0.0499		
	DVELDTTQSPYVFK	0.57	0.1593		
	LMMMGTADEVVK	0.15	0.0333		
	YLTVQFVR	0.13	0.0252		
Glyma02g10760.1	translation initiation factor 3 subunit H1	6	0.42	0.0085	protein
	Peptide Sequnce	Ratio 4(2)F	Pvalue 4(2)F		
	AAGEEPLPEEDPSNPIFKPLPEPSR	0.36	0.0042		
	FQFYYR	0.54	0.3085		
	LQLSTSSLMER	0.36	0.0117		
	LSDSFMELYR	0.71	0.5965		
	SFLQVAATEEAAPPLR	0.41	0.2301		
	VVQIEGLVILK	0.82	0.7633		
Glyma18g52060.1	translation initiation factor 3 subunit H1	6	0.42	0.0085	protein
	Peptide Sequnce	Ratio 4(2)F	Pvalue 4(2)F		
	AAGEEPLPEEDPSNPIFKPLPEPSR	0.36	0.0042		
	FQFYYR	0.54	0.3085		
	LQLSTSSLMER	0.36	0.0117		
	LSDSFMELYR	0.71	0.5965		
	SFLQVAATEEAAPPLR	0.41	0.2301		
	VVQIEGLVILK	0.82	0.7633		
Glyma03g12130.1	Metal dependent protein hydrolase	4	0.42	0.0017	not assigned
	Peptide Sequnce	Ratio 4(2)F	Pvalue 4(2)F		
	AMALAGSEFLDSVR	0.35	0.1207		
	LNLDWTDPDQSPEK	0.44	0.0118		
	SIVMETLEAR	0.63	0.3515		
	VQAVAVSPDSFQSR	0.33	0.0085		
Glyma06g12340.1	2 oxoglutarate (2OG) and Fe(II) dependent oxygenase superfamily protein	7	0.42	0.0004	hormone metabolism
	Peptide Sequnce	Ratio 4(2)F	Pvalue 4(2)F		
	ALNGGDGENAFFGTK	0.54	0.1734		
	ATICPAPQLVEK+Carbamidomethyl(4)	0.27	0.0805		
	FVFGDYMSVYAEQK	0.47	0.0408		

	LMEVMDENLGLTK	0.70	0.5240		
	SIASFYNPSFK	0.34	0.0033		
	VGGLQMLK	0.29	0.1364		
	VSHYPPCPHPELVK+Carbamidomethyl(7)	0.40	0.0289		
Glyma17g13770.1	homolog of nucleolar protein NOP56	3	0.43	0.0426	RNA
	Peptide Sequnce	Ratio 4(2)F	Pvalue 4(2)F		
	DTEMETEAPAEVSGK	0.42	0.0416		
	IPALTDIVGDEDK	0.53	0.1743		
	IVNDNYLYAK	0.04	0.2046		
Glyma08g47740.1	ENTH/VHS family protein	4	0.43	0.0005	not assigned
	Peptide Sequnce	Ratio 4(2)F	Pvalue 4(2)F		
	GLVNLNISGPK	0.52	0.0377		
	STVWADTLSR	0.39	0.0377		
	TNPLADIGVDFEAINR	0.26	0.0016		
	VLDATSNEPWGPHGSLLADIAQATR	2.14	0.4174		
Glyma18g53750.1	ENTH/VHS family protein	4	0.43	0.0005	not assigned
	Peptide Sequnce	Ratio 4(2)F	Pvalue 4(2)F		
	GLVNLNISGPK	0.52	0.0377		
	STVWADTLSR	0.39	0.0377		
	TNPLADIGVDFEAINR	0.26	0.0016		
	VLDATSNEPWGPHGSLLADIAQATR	2.14	0.4174		
Glyma15g37370.1	PLAT/LH2 domain containing lipoxygenase family protein	2	0.43	0.0184	hormone metabolism
	Peptide Sequnce	Ratio 4(2)F	Pvalue 4(2)F		
	DPELQAWWK	0.05	0.1426		
	HASDEFYLGQR	0.43	0.0187		
Glyma08g14670.2	myo inositol 1 phosphate synthase 3	5	0.43	0.0069	minor CHO metabolism
	Peptide Sequnce	Ratio 4(2)F	Pvalue 4(2)F		
	ACVGLAPENNMILEYK+Carbamidomethyl(2)	0.72	0.6006		
	IQQANYFGSLTQASAIR	0.20	0.0507		
	SNVVDDMVNSNAILYEPGEHPDHVVVIK	0.45	0.3881		
	VGSFQGEEIYAPFK	0.28	0.0331		
	VVVLWTANTER	0.12	0.0136		
Glyma02g00270.1	PENTATRICOPEPTIDE REPEAT 596	5	0.43	0.0201	RNA
	Peptide Sequnce	Ratio 4(2)F	Pvalue 4(2)F		
	AIANAPGLSVDSALNK	0.00	1.0000		

	IGPLTWDALVK	0.02	0.0703		
	TLANQLALVDAFR	0.41	0.0373		
	VEDCLAAVEAWGK+Carbamidomethyl(4)	0.14	0.1213		
	YIESVPESFR	0.58	0.0803		
Glyma18g33150.1	Nuclear transport factor 2 (NTF2) family protein	3	0.43	0.0452	protein
	Peptide Sequnce	Ratio 4(2)F	Pvalue 4(2)F		
	EGAAPSSTVTPVSVK	0.540340417	0.130600497		
	GLPPTATPAVLENEFK	0.589312056	0.505872479		
	GYFVLNDVFR	0.395790812	0.024292171		
Glyma11g00370.1	vaculolar sorting receptor 3	3	0.43	0.0100	protein
	Peptide Sequnce	Ratio 4(2)F	Pvalue 4(2)F		
	GDVTILPTLVVNNR	0.22	0.0023		
	VCECPLVDGVQFK+Carbamidomethyl(2)Carbamidomethyl(4)	0.57	0.0975		
	YCAPDPEQDFSTGYDGK+Carbamidomethyl(2)	0.00	1.0000		
Glyma04g02180.2	Prolyl oligopeptidase family protein	6	0.43	0.0224	protein
	Peptide Sequnce	Ratio 4(2)F	Pvalue 4(2)F		
	EPTAWADVLQESEK	0.40	0.0551		
	IPMFIVAK	0.45	0.3023		
	NVEPDTLSWVK	0.42	0.1484		
	VLEVHWIE	0.45	0.1016		
	WLEDPDAEEVK	0.41	0.0929		
	YTFGGSVTDDGK	0.47	0.3072		
Glyma16g04890.1	eukaryotic release factor 1 3	6	0.43	0.0007	protein
	Peptide Sequnce	Ratio 4(2)F	Pvalue 4(2)F		
	DSATSAELEVQEK	0.47	0.1079		
	FTVDLPK	0.38	0.0290		
	TELSQSDMFDPR	0.46	0.0539		
	YFEEISQDTGK	0.33	0.0220		
	YQLDIR	0.70	0.3356		
	YVFGVEDTLK	0.18	0.0376		
Glyma06g33940.2	RNA binding (RRM/RBD/RNP motifs) family protein	5	0.43	0.0000	RNA
	Peptide Sequnce	Ratio 4(2)F	Pvalue 4(2)F		
	GFALFVYK	0.51	0.0834		
	GLGWDTTTDGLR	0.53	0.0424		
	GYGFVTFR	0.45	0.0235		

	HLDVLAAVR	0.42	0.0810		
	SPEGAQAALIDPVK	0.41	0.0006		
Glyma15g11890.1	Phosphofructokinase family protein	12	0.44	0.0008	glycolysis
	Peptide Sequnce	Ratio 4(2)F	Pvalue 4(2)F		
	AMVELEGAPFK	0.55	0.3012		
	APTTTVPIGTLPSGSVTGR	0.19	0.0021		
	EVPTSFGFDTACK+Carbamidomethyl(12)	0.31	0.0799		
	IYSEMIGNVMIDAR	0.54	0.1158		
	KLDLDGLVVIGGDDSNTNACLLAENFR+Carbamidomethyl(20)	0.33	0.1019		
	LASVYSEVQK	0.51	0.1213		
	MLIQMVETELEK	0.58	0.6000		
	MLIQMVETELEKR	1.48	0.6975		
	NQGGFDMICSGR+Carbamidomethyl(9)	0.28	0.0723		
	NVTDYIVDVVCK+Carbamidomethyl(11)	0.36	0.0739		
	VDHALPLPSVLK	0.63	0.3973		
	YVELTSEYIYPYR	0.33	0.0785		
Glyma12g03070.1	nucleolin like 2	11	0.44	0.0000	protein
	Peptide Sequnce	Ratio 4(2)F	Pvalue 4(2)F		
	ADVEDFFK	0.38	0.0104		
	ALGLNGQQLFNR	0.60	0.2197		
	DAGEVVDVR	0.43	0.0096		
	GAYTPNSSNWNNSSQK	0.81	0.9856		
	GFAYVDFSDVDSMGK	0.52	0.1127		
	GFDTSLGEDEIR	0.31	0.1104		
	GFGHVEFATAAAAQK	0.07	0.0476		
	GSLQEHFGSCGDITR+Carbamidomethyl(10)	0.46	0.0269		
	LPALSVAPALK	0.32	0.0161		
	TLFVGNLPFSVER	0.52	0.0141		
	VDAVPAVVPPSK	0.29	0.0048		
Glyma08g11650.1	Chalcone and stilbene synthase family protein	4	0.44	0.0191	secondary metabolism
	Peptide Sequnce	Ratio 4(2)F	Pvalue 4(2)F		
	ITHLIFCTTSGVDMPGADYQLTK+Carbamidomethyl(7)	0.46	0.1027		
	VLVVCSEITAVTFR+Carbamidomethyl(5)	0.39	0.0939		
	YMMYQQGCFAGGTVLR+Carbamidomethyl(8)	0.41	0.0889		
	YMYLNEEILK	0.48	0.1236		

Glyma11g19070.1	NAD ADP ribosyltransferases;NAD ADP ribosyltransferases	9	0.44	0.0020	protein
	Peptide Sequnce	Ratio 4(2)F	Pvalue 4(2)F		
	CQDLLFYGALDK+Carbamidomethyl(1)	0.01	0.1079		
	EAWLIDSIEK	0.03	0.1069		
	LEPLVANFMK	0.21	0.1347		
	LFDEITGNEFEPWER	0.10	0.1292		
	LGCSISALDK+Carbamidomethyl(3)	0.31	0.0562		
	LPDSVQDSLASDLLK	0.14	0.0545		
	SETGVAEEYDEFCK+Carbamidomethyl(13)	0.03	0.0967		
	SSDDYEMIVK	0.06	0.1208		
	VLLWCGSR+Carbamidomethyl(5)	3.09	0.2616		
Glyma13g31500.1	Clathrin light chain protein	4	0.44	0.0022	not assigned
	Peptide Sequnce	Ratio 4(2)F	Pvalue 4(2)F		
	HTPPPHMIPPPPAPAK	0.46	0.1489		
	IIEEAEEYK	0.35	0.1832		
	KPSITVIQGPKPGKPTDLSR	0.55	0.0741		
	TIAELIPR	0.37	0.0029		
Glyma15g07810.1	Clathrin light chain protein	4	0.44	0.0022	not assigned
	Peptide Sequnce	Ratio 4(2)F	Pvalue 4(2)F		
	HTPPPHMIPPPPAPAK	0.46	0.1489		
	IIEEAEEYK	0.35	0.1832		
	KPSITVIQGPKPGKPTDLSR	0.55	0.0741		
	TIAELIPR	0.37	0.0029		
Glyma11g10120.1	Adaptin family protein	4	0.45	0.0251	cell
	Peptide Sequnce	Ratio 4(2)F	Pvalue 4(2)F		
	EYATEVDVDFVR	0.37	0.0757		
	ITEYLCDPLQR+Carbamidomethyl(6)	0.30	0.0361		
	NANQDVFYFSAK	0.72	0.7541		
	NIDQVLLEFK	0.30	0.0761		
Glyma01g43680.1	Adaptin family protein	7	0.45	0.0413	cell
	Peptide Sequnce	Ratio 4(2)F	Pvalue 4(2)F		
	AYIYWR	0.54	0.4080		
	EYATEVDVDFVR	0.37	0.0757		
	ITEYLCDPLQR+Carbamidomethyl(6)	0.30	0.0361		
	LAASNMFFIAK	0.35	0.4129		

	LYDINAELVEDR	0.45	0.1929		
	NANQDVFYFSAK	0.72	0.7541		
	NIDQVLLEFK	0.30	0.0761		
Glyma11g01800.1	Adaptin family protein	7	0.45	0.0413	cell
	Peptide Sequnce	Ratio 4(2)F	Pvalue 4(2)F		
	AYIYWR	0.54	0.4080		
	EYATEVDVDFVR	0.37	0.0757		
	ITEYLCDPLQR+Carbamidomethyl(6)	0.30	0.0361		
	LAASNMFFIAK	0.35	0.4129		
	LYDINAELVEDR	0.45	0.1929		
	NANQDVFYFSAK	0.72	0.7541		
	NIDQVLLEFK	0.30	0.0761		
Glyma14g17930.1	serine/arginine rich 22	2	0.45	0.0141	RNA
	Peptide Sequnce	Ratio 4(2)F	Pvalue 4(2)F		
	DLEDEFR	0.41	0.0062		
	RPPGYAFIDFDDR	0.60	0.3165		
Glyma17g29080.1	serine/arginine rich 22	2	0.45	0.0141	RNA
	Peptide Sequnce	Ratio 4(2)F	Pvalue 4(2)F		
	DLEDEFR	0.41	0.0062		
	RPPGYAFIDFDDR	0.60	0.3165		
Glyma19g30251.1	RNA binding (RRM/RBD/RNP motifs) family protein	5	0.45	0.0000	RNA
	Peptide Sequnce	Ratio 4(2)F	Pvalue 4(2)F		
	FGEIEEGPLGLDK	0.51	0.0119		
	GFCLFVYR+Carbamidomethyl(3)	0.34	0.0262		
	GYGFILFK	0.45	0.0251		
	IYVSNVGADLDPQK	0.39	0.0349		
	LLAFFSR	0.36	0.0236		
Glyma13g23790.1	formate dehydrogenase	6	0.45	0.0180	C1-metabolism
	Peptide Sequnce	Ratio 4(2)F	Pvalue 4(2)F		
	CDVIVINTPLTEQTR+Carbamidomethyl(1)	0.22	0.0182		
	EGQLASQYR	2.39	0.4242		
	FEEDLDAMLPK	0.28	0.1626		
	GEDFPEQNYIVK	0.26	0.0757		
	HIPDAHVIISTPFHPAYVTAER	0.26	0.1683		
	IVGVFYK	0.56	0.3175		

Glyma10g07820.1	monodehydroascorbate reductase 1	15	0.45	0.0040	redox
	Peptide Sequnce	Ratio 4(2)F	Pvalue 4(2)F		
	AYLFPESPAR	0.44	0.1511		
	FGTYWIK	0.44	0.2377		
	GTVAVGFTSNSDGEVK	0.46	0.3201		
	LFTAGIAEFYEGYYANK	0.51	0.4792		
	LLPEWYTEK	0.47	0.2623		
	LNNIDVTMVYPEPWCMPR+Carbamidomethyl(15)	0.03	0.1939		
	LPGFHVCVGSGGER+Carbamidomethyl(7)	0.59	0.3851		
	LTDFGVEGADAK	0.40	0.1885		
	NIFYLR	0.50	0.1696		
	QGVKPGELAIISK	0.57	0.1462		
	TNLSDVYAVGDVATFPLK	0.45	0.0330		
	TVEEYDYLPYFYSR	0.54	0.3125		
	VLEADIVVVGVGGRPQTVLVK	0.36	0.0854		
	VQPPVADVNQLAK	0.37	0.0173		
	VVGVFLEGGTPEENQAIAK	0.44	0.1827		
Glyma15g04140.1	Ubiquitin fusion degradation UFD1 family protein	2	0.45	0.0279	protein
	Peptide Sequnce	Ratio 4(2)F	Pvalue 4(2)F		
	AILETTLR	0.40	0.0138		
	DFLDISNPK	0.60	0.3150		
Glyma12g02440.1	Adaptin family protein	6	0.46	0.0313	cell
	Peptide Sequnce	Ratio 4(2)F	Pvalue 4(2)F		
	AYIYWR	0.54	0.4080		
	EYATEVDVDFVR	0.37	0.0757		
	ITEYLCDPLQR+Carbamidomethyl(6)	0.30	0.0361		
	LYDINAELVEDR	0.45	0.1929		
	NANQDVFYFSAK	0.72	0.7541		
	NIDQVLLEFK	0.30	0.0761		
Glyma02g32100.1	villin 2	3	0.46	0.0042	cell
	Peptide Sequnce	Ratio 4(2)F	Pvalue 4(2)F		
	ALEVIQFLK	0.46	0.5457		
	IYQFNGANSNIQER	0.62	0.3002		
	SDSSEVEEVAEAK	0.14	0.0005		
Glyma10g21350.2	villin 2	3	0.46	0.0042	cell

	Peptide Sequnce	Ratio 4(2)F	Pvalue 4(2)F		
	ALEVIQFLK	0.46	0.5457		
	IYQFNGANSNIQER	0.62	0.3002		
	SDSSEVEEVAEAK	0.14	0.0005		
Glyma04g40760.1	CTC interacting domain 11	4	0.46	0.0268	RNA
	Peptide Sequnce	Ratio 4(2)F	Pvalue 4(2)F		
	AALNLSGTMLGYYPLR	0.72	0.6070		
	DLEELLSK	0.37	0.1967		
	TAIAPVNPTFLPR	0.36	0.0091		
	VCGDPNSILR+Carbamidomethyl(2)	0.53	0.1588		
Glyma06g14030.1	CTC interacting domain 11	4	0.46	0.0268	RNA
	Peptide Sequnce	Ratio 4(2)F	Pvalue 4(2)F		
	AALNLSGTMLGYYPLR	0.72	0.6070		
	DLEELLSK	0.37	0.1967		
	TAIAPVNPTFLPR	0.36	0.0091		
	VCGDPNSILR+Carbamidomethyl(2)	0.53	0.1588		
Glyma04g35950.1	ATPase AAA type CDC48 protein	12	0.46	0.0002	cell
	Peptide Sequnce	Ratio 4(2)F	Pvalue 4(2)F		
	AIANECQANFISVK+Carbamidomethyl(6)	0.03	0.0603		
	DTHGYVGADLAALCTEAALQCIR+Carbamidomethyl(14)Carbamidomethyl(21)	0.02	0.0541		
	EIDIGVPDEVGR	0.06	0.0576		
	ELQETVQYPVEHPEK	0.07	0.2833		
	ELVELPLR	0.02	0.0982		
	GILLYGPPGSGK	0.07	0.0858		
	GVLFYGPPGCGK+Carbamidomethyl(10)	0.04	0.0658		
	LAGESESNLR	2.10	0.1645		
	LGDVVSVHQCPDVK+Carbamidomethyl(10)	0.55	0.1658		
	LNDVGYDDVGGVR	0.20	0.0222		
	TVFIIGATNRPDIIDPALLRPGR	0.01	0.3223		
	VLNQLLTEMDGMTAK	0.69	0.1243		
Glyma08g11520.1	Chalcone and stilbene synthase family protein	5	0.46	0.0244	secondary metabolism
	Peptide Sequnce	Ratio 4(2)F	Pvalue 4(2)F		
	EVGLTFHLLK	0.57	0.3228		
	ITHLIFCTTSGVDMPGADYQLTK+Carbamidomethyl(7)	0.46	0.1027		
	VLVVCSEITAVTFR+Carbamidomethyl(5)	0.39	0.0939		

	YMMYQQGCFAGGTVLR+Carbamidomethyl(8)	0.41	0.0889		
	YMYLNEEILK	0.48	0.1236		
Glyma05g10840.2	Cobalamin independent synthase family protein	7	0.46	0.0139	amino acid metabolism
	Peptide Sequnce	Ratio 4(2)F	Pvalue 4(2)F		
	AGITVIQIDEAALR	0.55	0.3846		
	FALESFWEGK	0.09	0.0385		
	IVEVNALAK	0.34	0.0406		
	MLAVLEK	0.19	0.1251		
	NILWVNPDCGLK+Carbamidomethyl(9)	0.51	0.1395		
	SWLAFAAQK	0.45	0.2477		
	YGAGIGPGVYDIHSPR	0.56	0.3091		
Glyma04g08131.1	oligouridylate binding protein 1B	3	0.46	0.0176	RNA
	Peptide Sequnce	Ratio 4(2)F	Pvalue 4(2)F		
	DHQDAQSAINDMTGK	0.58	0.0893		
	GYGFVSFR	0.39	0.0799		
	LLAEVFQSAGPLAGCK+Carbamidomethyl(15)	0.41	0.0647		
Glyma06g08200.1	oligouridylate binding protein 1B	3	0.46	0.0176	RNA
	Peptide Sequnce	Ratio 4(2)F	Pvalue 4(2)F		
	DHQDAQSAINDMTGK	0.58	0.0893		
	GYGFVSFR	0.39	0.0799		
	LLAEVFQSAGPLAGCK+Carbamidomethyl(15)	0.41	0.0647		
Glyma08g40380.1	Thiamin diphosphate binding fold (THDP binding) superfamily protein	5	0.46	0.0023	TCA / org transformation
	Peptide Sequnce	Ratio 4(2)F	Pvalue 4(2)F		
	ESQMPEPSDLFTNVYVK	0.27	0.0172		
	LLLTHEIATEK	0.28	0.0074		
	MEIAADSLYK	0.21	0.1311		
	NGPIILEMDTYR	0.48	0.2085		
	VDGMDALAVK	0.75	0.3337		
Glyma11g10790.1	nucleolin like 2	10	0.46	0.0000	protein
	Peptide Sequnce	Ratio 4(2)F	Pvalue 4(2)F		
	ADVEGFFK	0.57	0.0145		
	DAGEVVDVR	0.43	0.0096		
	DVEMVDAALSEK	0.17	0.0036		
	GAYTPNSSNWNNSSQK	0.81	0.9856		
	GFAYVDFGDADSMGK	0.25	0.0576		

	GFDTSLGEDEIR	0.31	0.1104		
	GSLQEHFGSCGDITR+Carbamidomethyl(10)	0.46	0.0269		
	LPTLPVAPAK	0.17	0.0119		
	TLFVGNLPFSVER	0.52	0.0141		
	VDAAPAVVPPSK	0.03	0.0050		
Glyma20g16070.1	heat shock protein 70 (Hsp 70) family protein	6	0.46	0.0355	stress
	Peptide Sequnce	Ratio 4(2)F	Pvalue 4(2)F		
	IETLEEFEK	0.40	0.0852		
	LLGEEAAGLAAR	0.42	0.0734		
	NNLEGYIYTTK	0.56	0.1943		
	SGILSLDR	0.32	0.1154		
	SPALVSFHDGDR	0.48	0.2086		
	SVSVNQFQVK	0.57	0.5200		
Glyma15g42140.2	ATP citrate lyase A 1	6	0.46	0.0146	TCA / org transformation
	Peptide Sequnce	Ratio 4(2)F	Pvalue 4(2)F		
	ALGEEIGIPIEVYGPEATMTGICK+Carbamidomethyl(23)	0.01	0.0246		
	EDEVLQYAR	0.31	0.0567		
	FTVLNPMGR	0.03	0.1091		
	LVVKPDMLFGK	0.27	0.0432		
	SAQVTESTNFSELAEK	0.86	0.5535		
	VVIDCATSNPDGQK+Carbamidomethyl(5)	0.00	1.0000		
Glyma13g31200.1	Eukaryotic translation initiation factor 3 subunit 7 (eIF 3)	7	0.46	0.0005	protein
	Peptide Sequnce	Ratio 4(2)F	Pvalue 4(2)F		
	DGSQLDLLSVHETSQEPLPESK	0.34	0.0001		
	LDDDMYLVAR	0.36	0.1931		
	SFLTLNALNEFDPK	0.80	0.5563		
	SIVDLCMK+Carbamidomethyl(6)	0.24	0.1271		
	SSVDIQPEWNMHDQIPFSTFSK	1.64	0.4815		
	VTFDEANPFANEGEEVASVAYR	0.33	0.0086		
	WTAQALLASADTMK	0.75	0.6737		
Glyma15g08150.1	Eukaryotic translation initiation factor 3 subunit 7 (eIF 3)	7	0.46	0.0005	protein
	Peptide Sequnce	Ratio 4(2)F	Pvalue 4(2)F		
	DGSQLDLLSVHETSQEPLPESK	0.34	0.0001		
	LDDDMYLVAR	0.36	0.1931		
	SFLTLNALNEFDPK	0.80	0.5563		

	SIVDLCMK+Carbamidomethyl(6)	0.24	0.1271		
	SSVDIQPEWNMHDQIPFSTFSK	1.64	0.4815		
	VTFDEANPFANEGEEVASVAYR	0.33	0.0086		
	WTAQALLASADTMK	0.75	0.6737		
Glyma10g35750.1	ATP synthase D chain mitochondrial	5	0.46	0.0153	mitochondrial electron transport
	Peptide Sequnce	Ratio 4(2)F	Pvalue 4(2)F		
	AFDEVNSQLETK	0.46	0.0839		
	FDALLIELK	0.62	0.3536		
	FSQEPEPIDWDYYR	0.76	0.5514		
	FVDTVTPQYK	0.38	0.0011		
	LSTMTADEYFEK	0.92	0.9137		
Glyma02g38921.1	Histone superfamily protein	4	0.46	0.0012	DNA
	Peptide Sequnce	Ratio 4(2)F	Pvalue 4(2)F		
	DNIQGITKPAIR	0.47	0.1003		
	IFLENVIR	0.13	0.0553		
	ISGLIYEETR	0.62	0.0851		
	TVTAMDVVYALK	0.20	0.0054		
Glyma13g21050.2	Histone superfamily protein	4	0.46	0.0012	DNA
	Peptide Sequnce	Ratio 4(2)F	Pvalue 4(2)F		
	DNIQGITKPAIR	0.47	0.1003		
	IFLENVIR	0.13	0.0553		
	ISGLIYEETR	0.62	0.0851		
	TVTAMDVVYALK	0.20	0.0054		
Glyma14g36970.1	Histone superfamily protein	4	0.46	0.0012	DNA
	Peptide Sequnce	Ratio 4(2)F	Pvalue 4(2)F		
	DNIQGITKPAIR	0.47	0.1003		
	IFLENVIR	0.13	0.0553		
	ISGLIYEETR	0.62	0.0851		
	TVTAMDVVYALK	0.20	0.0054		
Glyma15g04805.1	Histone superfamily protein	4	0.46	0.0012	DNA
	Peptide Sequnce	Ratio 4(2)F	Pvalue 4(2)F		
	DNIQGITKPAIR	0.47	0.1003		
	IFLENVIR	0.13	0.0553		
	ISGLIYEETR	0.62	0.0851		
	TVTAMDVVYALK	0.20	0.0054		

Glyma18g07153.1	Histone superfamily protein	4	0.46	0.0012	DNA
	Peptide Sequnce	Ratio 4(2)F	Pvalue 4(2)F		
	DNIQGITKPAIR	0.47	0.1003		
	IFLENVIR	0.13	0.0553		
	ISGLIYEETR	0.62	0.0851		
	TVTAMDVVYALK	0.20	0.0054		
Glyma01g03610.1	Transducin/WD40 repeat like superfamily protein	6	0.47	0.0337	protein
	Peptide Sequnce	Ratio 4(2)F	Pvalue 4(2)F		
	DGDLLFSCAK+Carbamidomethyl(8)	0.39	0.0505		
	IANDPAEQTGESVLLIK	0.22	0.1352		
	ILQEEIGGVK	0.31	0.0578		
	LAVITTDPFMELPSAIHVK	0.89	0.7979		
	LHHFDPDYFNIK	0.44	0.3300		
	TIISAGEDAVIR	0.59	0.1344		
Glyma17g21490.1	SKU5 similar 17	2	0.47	0.0448	misc
	Peptide Sequnce	Ratio 4(2)F	Pvalue 4(2)F		
	QYLGQQLYLR	0.44	0.1062		
	TYNLVDALTR	0.48	0.0718		
Glyma20g30730.1	S18 ribosomal protein	4	0.47	0.0207	protein
	Peptide Sequnce	Ratio 4(2)F	Pvalue 4(2)F		
	AGELSAAELDSVMTVVANPR	0.73	0.1411		
	IMFALTSIK	0.00	1.0000		
	IPDWFLNR	0.05	0.0371		
	YSQVVSNALDMK	0.17	0.0227		
Glyma17g13361.1	RAB GTPase homolog E1B	2	0.47	0.0230	protein
	Peptide Sequnce	Ratio 4(2)F	Pvalue 4(2)F		
	FSAIVYVLK	0.51	0.2769		
	TVGAGVIQSIIE	0.46	0.0125		
Glyma10g44351.1	ENTH/VHS family protein	2	0.47	0.0108	not assigned
	Peptide Sequnce	Ratio 4(2)F	Pvalue 4(2)F		
	GLVNLNISGPK	0.52	0.0377		
	STVWADTLSR	0.39	0.0377		
Glyma20g39181.1	ENTH/VHS family protein	2	0.47	0.0108	not assigned
	Peptide Sequnce	Ratio 4(2)F	Pvalue 4(2)F		
	GLVNLNISGPK	0.52	0.0377		

	STVWADTLSR	0.39	0.0377		
Glyma09g01050.1	Phosphofructokinase family protein	14	0.47	0.0009	glycolysis
	Peptide Sequnce	Ratio 4(2)F	Pvalue 4(2)F		
	AMVELEGAPFK	0.55	0.3012		
	EVPTSFGFDTACK+Carbamidomethyl(12)	0.31	0.0799		
	EWTVGGTALTSMMDVER	0.16	0.0538		
	IYSEMIGNVMIDAR	0.54	0.1158		
	KLDLDGLVVIGGDDSNTNACLLAENFR+Carbamidomethyl(20)	0.33	0.1019		
	LASVYSEVQK	0.51	0.1213		
	LFPHLFGQPSAALAR	0.31	0.0949		
	MLIQMVETELEK	0.58	0.6000		
	MLIQMVETELEKR	1.48	0.6975		
	NQGGFDMICSGR+Carbamidomethyl(9)	0.28	0.0723		
	NVTDYIVDVVCK+Carbamidomethyl(11)	0.36	0.0739		
	TGLISSVGNLCAPVK+Carbamidomethyl(11)	0.24	0.0327		
	VDHALPLPSVLK	0.63	0.3973		
	YVELTSEYIYPYR	0.33	0.0785		
Glyma08g44210.1	FASCICLIN like arabinogalactan 1	6	0.47	0.0000	cell wall
	Peptide Sequnce	Ratio 4(2)F	Pvalue 4(2)F		
	AALLEFHAVPVYQSK	0.81	0.3955		
	FDFTVQNDGEDVTLK	0.50	0.0155		
	SNNGLQNTLATDGANK	0.66	0.4797		
	SNNGLQNTLATDGANKFDFTVQNDGEDVTLK	4.72	0.4475		
	TTITVCAVDNAAMSDLLSK+Carbamidomethyl(6)	0.49	0.1630		
	VAFAPENNDGTLSSTFVK	0.41	0.0000		
Glyma05g03320.1	purple acid phosphatase 27	3	0.47	0.0158	misc
	Peptide Sequnce	Ratio 4(2)F	Pvalue 4(2)F		
	DPGFIHTSFLK	0.47	0.1585		
	TCPIYQNQCVNDER+Carbamidomethyl(2)Carbamidomethyl(9)	0.47	0.0126		
	VIIFGDMGK	0.48	0.2003		
Glyma02g46650.1	RNA binding (RRM/RBD/RNP motifs) family protein	5	0.47	0.0071	RNA
	Peptide Sequnce	Ratio 4(2)F	Pvalue 4(2)F		
	GFGFITYDSEEAVDR	0.63	0.5641		
	GFGFVVFADPSAAER	0.19	0.0530		
	IFVGGLPSTITESDFK	0.40	0.0149		

	LFIGGISWDTDDER	0.27	0.0400		
	SPLIGYNYGLNR	0.68	0.3119		
Glyma14g02020.3	RNA binding (RRM/RBD/RNP motifs) family protein	5	0.47	0.0071	RNA
	Peptide Sequnce	Ratio 4(2)F	Pvalue 4(2)F		
	GFGFITYDSEEAVDR	0.63	0.5641		
	GFGFVVFADPSAAER	0.19	0.0530		
	IFVGGLPSTITESDFK	0.40	0.0149		
	LFIGGISWDTDDER	0.27	0.0400		
	SPLIGYNYGLNR	0.68	0.3119		
Glyma08g38420.1	PLAT/LH2 domain containing lipoxygenase family protein	8	0.47	0.0031	hormone metabolism
	Peptide Sequnce	Ratio 4(2)F	Pvalue 4(2)F		
	DGGDYWTSDAGPLEAFK	0.05	0.1130		
	DGGDYWTSDAGPLEAFKR	0.24	0.0743		
	DPELQAWWK	0.05	0.1426		
	ELVEVGHGDLK	0.07	0.2031		
	GIPNSISI	0.11	0.0765		
	HASDEFYLGQR	0.43	0.0187		
	MPYTLLYPSSEEGLTFR	0.06	0.1356		
	MPYTLLYPSSEEGLTFR+Oxidation(1)	2.04	0.3557		
Glyma03g38730.2	aspartic proteinase A1	3	0.47	0.0154	protein
	Peptide Sequnce	Ratio 4(2)F	Pvalue 4(2)F		
	DGGDYWTSDAGPLEAFK	0.05	0.1130		
	ISYGTGSISGFFSQDNVK	0.43	0.0085		
	VFSFWLNGDPNAK	0.44	0.1474		
Glyma11g14360.1	RAB GTPase homolog A2B	4	0.47	0.0014	signalling
	Peptide Sequnce	Ratio 4(2)F	Pvalue 4(2)F		
	AQIWDTAGQER	0.71	0.4462		
	EGLSFLETSALEATNIEK	0.11	0.0001		
	IVLIGDSGVGK	0.68	0.0770		
	STIGVEFATR	0.83	0.7359		
Glyma12g06280.2	RAB GTPase homolog A2B	4	0.47	0.0014	signalling
	Peptide Sequnce	Ratio 4(2)F	Pvalue 4(2)F		
	AQIWDTAGQER	0.71	0.4462		
	EGLSFLETSALEATNIEK	0.11	0.0001		
	IVLIGDSGVGK	0.68	0.0770		

	STIGVEFATR	0.83	0.7359		
Glyma16g04560.1	glutamate dehydrogenase 1	8	0.47	0.0004	N-metabolism
	Peptide Sequnce	Ratio 4(2)F	Pvalue 4(2)F		
	DDGTLQSYVGFR	0.60	0.2397		
	FIVEAANHPTDPEADEILK	0.62	0.0654		
	GGIGCDPAELSISELER+Carbamidomethyl(5)	0.51	0.0523		
	GVLFATEALLNEYGK	1.66	0.3137		
	MGAFTLAVNR	0.76	0.2201		
	SLLIPFR	0.14	0.0166		
	TAVANIPYGGAK	0.43	0.0498		
	VVAVSDITGAIK	0.33	0.0164		
Glyma19g28770.2	glutamate dehydrogenase 1	8	0.47	0.0004	N-metabolism
	Peptide Sequnce	Ratio 4(2)F	Pvalue 4(2)F		
	DDGTLQSYVGFR	0.60	0.2397		
	FIVEAANHPTDPEADEILK	0.62	0.0654		
	GGIGCDPAELSISELER+Carbamidomethyl(5)	0.51	0.0523		
	GVLFATEALLNEYGK	1.66	0.3137		
	MGAFTLAVNR	0.76	0.2201		
	SLLIPFR	0.14	0.0166		
	TAVANIPYGGAK	0.43	0.0498		
	VVAVSDITGAIK	0.33	0.0164		
Glyma04g40750.2	CTC interacting domain 11	4	0.47	0.0241	RNA
	Peptide Sequnce	Ratio 4(2)F	Pvalue 4(2)F		
	AALSLSGTMLGYYPLR	0.74	0.5191		
	DLEELLSK	0.37	0.1967		
	TAIAPVNPTFLPR	0.36	0.0091		
	VCGDPNSILR+Carbamidomethyl(2)	0.53	0.1588		
Glyma06g14050.3	CTC interacting domain 11	4	0.47	0.0241	RNA
	Peptide Sequnce	Ratio 4(2)F	Pvalue 4(2)F		
	AALSLSGTMLGYYPLR	0.74	0.5191		
	DLEELLSK	0.37	0.1967		
	TAIAPVNPTFLPR	0.36	0.0091		
	VCGDPNSILR+Carbamidomethyl(2)	0.53	0.1588		
Glyma01g43470.1	AMP dependent synthetase and ligase family protein	3	0.48	0.0435	lipid metabolism
	Peptide Sequnce	Ratio 4(2)F	Pvalue 4(2)F		

	FIIEVEK	0.14	0.1584		
	LILSGAAPLSAHVEGYLR	0.16	0.0754		
	LLESVNEQLTEK	0.55	0.1280		
Glyma04g39390.1	carbamoyl phosphate synthetase A	3	0.48	0.0422	nucleotide metabolism
	Peptide Sequnce	Ratio 4(2)F	Pvalue 4(2)F		
	ESLHQLSAAQALDA	0.59	0.2830		
	LVLEDGSIWR	0.38	0.0416		
	NIMGIYDVDTR	0.49	0.1241		
Glyma06g07140.1	epsin N terminal homology (ENTH) domain containing protein	6	0.48	0.0139	not assigned
	Peptide Sequnce	Ratio 4(2)F	Pvalue 4(2)F		
	FFDMEYADCVK+Carbamidomethyl(9)	0.28	0.3632		
	LLETLEEFVR	0.28	0.0232		
	LLNMSDFR	0.77	0.7627		
	MVLIALYPVVK	0.35	0.1150		
	TYALYLDQR	0.29	0.0445		
	VEEEPAPDMNEIK	0.52	0.1027		
Glyma19g43490.1	ankyrin repeat family protein	3	0.48	0.0375	cell
	Peptide Sequnce	Ratio 4(2)F	Pvalue 4(2)F		
	AVEILFPLTSK	0.44	0.3716		
	LALQLDEGK	0.53	0.1123		
	VQLFLNAAR	0.45	0.0299		
Glyma13g43630.1	heat shock protein 91	24	0.48	0.0006	stress
	Peptide Sequnce	Ratio 4(2)F	Pvalue 4(2)F		
	ALAEAGLTVENVHMVEVVGSGSR	0.30	0.2398		
	ATANAPGAENGTPEAGDKPVQMDTDTK	0.31	0.0711		
	AVLDAATIAGLHPLR	0.47	0.2935		
	DEFEQLSLPILER	0.88	0.5565		
	DFDEVLFNHFAAK	0.40	0.1635		
	EFEMALQDR	0.85	0.8442		
	ETPAIVCFGDK+Carbamidomethyl(7)	0.64	0.3207		
	FLGTAGAASTMMNPK	0.60	0.4884		
	GCALQCAILSPTFK+Carbamidomethyl(2)Carbamidomethyl(6)	0.40	0.1334		
	GIDVVLNDESKR	0.44	0.0934		
	GTVIDQLAYCINSYR+Carbamidomethyl(10)	1.13	0.7391		
	IDVFQNAR	0.56	0.2052		

	ISTYTIGPFQSTK	0.38	0.1455		
	LFHETTATALAYGIYK	0.43	0.2246		
	LQEVEDWLYEDGEDETK	0.43	0.0353		
	NAVEAYVYDMR	0.53	0.1443		
	QFADPELQQDIK	0.38	0.0579		
	RDEFEQLSLPILER	0.56	0.3404		
	SGTFSIDVQYDDVSGLQTPAK	0.65	0.5500		
	TFPFVVTEGPDGYPLIHAR	0.20	0.0137		
	VLNECVEAENWLR+Carbamidomethyl(5)	0.38	0.0681		
	VLSANPEAPLNIECLMDEK+Carbamidomethyl(14)	0.39	0.0929		
	YATPVLLSADVR	0.41	0.0614		
	YQEFVIDSER	0.43	0.1513		
Glyma01g12340.3	ARF GAP domain 8	4	0.48	0.0011	protein
	Peptide Sequnce	Ratio 4(2)F	Pvalue 4(2)F		
	DNNIDLTAGDLINR	0.38	0.0127		
	LSFQAQQDLSSLK	0.59	0.0817		
	LSSLASTLMTDLQDR	0.57	0.1542		
	STNLDSWSPEQLK	0.27	0.0150		
Glyma03g40000.1	D amino acid aminotransferase like PLP dependent enzymes	3	0.48	0.0251	misc
	Peptide Sequnce	Ratio 4(2)F	Pvalue 4(2)F		
	ALAFENVPTQDEIK	0.22	0.0764		
	IIGNGEVGPVTR	0.74	0.6448		
	NPLDILPSFDK	0.16	0.0149		
Glyma05g31490.1	aspartate aminotransferase	5	0.48	0.0116	amino acid metabolism
	Peptide Sequnce	Ratio 4(2)F	Pvalue 4(2)F		
	IQSQFTSGASSIAQK	0.37	0.0497		
	ISYAESLTTLQAAVER	0.50	0.0472		
	LAAGEPDFDTPAPIAEAGINAIR	0.49	0.0969		
	LLILCSPSNPTGSVYPK+Carbamidomethyl(5)	0.39	0.0976		
	YTPNAGTMELR	0.70	0.5137		
Glyma08g14720.1	aspartate aminotransferase	5	0.48	0.0116	amino acid metabolism
	Peptide Sequnce	Ratio 4(2)F	Pvalue 4(2)F		
	IQSQFTSGASSIAQK	0.37	0.0497		
	ISYAESLTTLQAAVER	0.50	0.0472		
	LAAGEPDFDTPAPIAEAGINAIR	0.49	0.0969		

	LLILCSPSNPTGSVYPK+Carbamidomethyl(5)	0.39	0.0976		
	YTPNAGTMELR	0.70	0.5137		
Glyma08g20610.1	Root hair defective 3 GTP binding protein (RHD3)	7	0.48	0.0029	development
	Peptide Sequnce	Ratio 4(2)F	Pvalue 4(2)F		
	FHHSIAPGGLAGDR	0.92	0.8721		
	FIPTIMNLMR	0.49	0.2824		
	ISELTSSYEEK	0.24	0.0191		
	LFQLVQPAFQSALGHIR	0.20	0.0397		
	STLLNNLFGTNFR	1.16	0.8171		
	TPLENLEPVLR	0.26	0.0250		
	TTLLFVIR	0.09	0.0179		
Glyma07g00540.1	D 3 phosphoglycerate dehydrogenase	7	0.48	0.0107	amino acid metabolism
	Peptide Sequnce	Ratio 4(2)F	Pvalue 4(2)F		
	AIMAIGVDEEPNK	0.21	0.0301		
	GGVIDEDALVR	0.86	0.5919		
	IFNDNTFAK	0.25	0.0287		
	LAVQLVCGGSGIK+Carbamidomethyl(7)	0.39	0.4193		
	LGEAGLQVLR	0.27	0.0531		
	VGNILGEQNVNVSFMSVGR	0.29	0.1640		
	YVGVSMVGK	0.52	0.2821		
Glyma08g23860.1	D 3 phosphoglycerate dehydrogenase	7	0.48	0.0107	amino acid metabolism
	Peptide Sequnce	Ratio 4(2)F	Pvalue 4(2)F		
	AIMAIGVDEEPNK	0.21	0.0301		
	GGVIDEDALVR	0.86	0.5919		
	IFNDNTFAK	0.25	0.0287		
	LAVQLVCGGSGIK+Carbamidomethyl(7)	0.39	0.4193		
	LGEAGLQVLR	0.27	0.0531		
	VGNILGEQNVNVSFMSVGR	0.29	0.1640		
	YVGVSMVGK	0.52	0.2821		
Glyma15g43200.3	sterol methyltransferase 1	4	0.48	0.0043	hormone metabolism
	Peptide Sequnce	Ratio 4(2)F	Pvalue 4(2)F		
	AEIEIGDGLPDIR	0.36	0.0292		
	FSLTSITGLNNNEYQITR	0.55	0.1050		
	QAGFEVIWEK	0.34	0.0863		
	VLDVGCGIGGPLR+Carbamidomethyl(6)	0.45	0.0529		

Glyma03g39890.1	mevalonate kinase	2	0.48	0.0275	secondary metabolism
	Peptide Sequnce	Ratio 4(2)F	Pvalue 4(2)F		
	LQETALEFSWPITR	0.40	0.0592		
	SPTPDELSVTEK	0.52	0.0718		
Glyma09g36760.2	nucleotide sensitive chloride conductance regulator (ICln) family protein	2	0.48	0.0279	transport
	Peptide Sequnce	Ratio 4(2)F	Pvalue 4(2)F		
	FEDAEEMEDNGDGTNR	1.20	0.7543		
	QVIWLSDLDK	0.31	0.0057		
Glyma01g45380.1	vaculolar sorting receptor 3	2	0.48	0.0021	protein
	Peptide Sequnce	Ratio 4(2)F	Pvalue 4(2)F		
	GDVTILPTLVVNNR	0.22	0.0023		
	VCECPLVDGVQFK+Carbamidomethyl(2)Carbamidomethyl(4)	0.57	0.0975		
Glyma04g07050.2	epsin N terminal homology (ENTH) domain containing protein	5	0.49	0.0239	not assigned
	Peptide Sequnce	Ratio 4(2)F	Pvalue 4(2)F		
	LDLMLFDR	0.06	0.1594		
	LLETLEEFVR	0.28	0.0232		
	LLNMSDFR	0.77	0.7627		
	MVLIALYPVVK	0.35	0.1150		
	VEEEPAPDMNEIK	0.52	0.1027		
Glyma04g11160.3	N.D. *	3	0.49	0.0002	not assigned
	Peptide Sequnce	Ratio 4(2)F	Pvalue 4(2)F		
	ELSGTLESEDSILK	0.57	0.0297		
	EMSGSNIFADGK	0.28	0.0031		
	FAELSGNDIFK	0.46	0.0162		
Glyma06g10930.2	N.D. *	3	0.49	0.0002	not assigned
	Peptide Sequnce	Ratio 4(2)F	Pvalue 4(2)F		
	ELSGTLESEDSILK	0.57	0.0297		
	EMSGSNIFADGK	0.28	0.0031		
	FAELSGNDIFK	0.46	0.0162		
Glyma20g28700.2	carbamoyl phosphate synthetase B	2	0.49	0.0478	nucleotide metabolism
	Peptide Sequnce	Ratio 4(2)F	Pvalue 4(2)F		
	LSLGVTPAYK	0.40	0.0280		
	STGEVMGIDPSYNIAFAK	0.54	0.2945		
Glyma05g35350.1	aldehyde dehydrogenase 2C4	9	0.49	0.0170	fermentation

	Peptide Sequnce	Ratio 4(2)F	Pvalue 4(2)F		
	AWVVGDPFDPK	0.39	0.0727		
	GYFIEPTIFSNIR	0.98	0.9705		
	ISEGDKEDIDIAVK	0.65	0.2668		
	NLEVPAAANTLR	0.57	0.4872		
	SPLIIFDDADIDK	0.29	0.0235		
	SVVTPLYNSPWL	0.55	0.1113		
	TFETIDPR	0.33	0.1923		
	VLVQEGIYDEFEK	0.50	0.1345		
	YGLAAGIVTK	0.40	0.1133		
Glyma20g12220.1	Plant L ascorbate oxidase	6	0.49	0.0042	redox
	Peptide Sequnce	Ratio 4(2)F	Pvalue 4(2)F		
	AEVGDILDIALTNK	0.53	0.0660		
	FDVEYMIR	0.43	0.1612		
	GGEECAPQILHVEPNK+Carbamidomethyl(5)	0.51	0.1065		
	LFTEGTVIHWHGIR	0.39	0.0689		
	NTAVIFPYGWTALR	0.59	0.1702		
	NYWLSIGVR	0.38	0.0419		
Glyma16g30190.1	tripeptidyl peptidase ii	12	0.49	0.0002	protein
	Peptide Sequnce	Ratio 4(2)F	Pvalue 4(2)F		
	AALDTQSLEDDPNCGK+Carbamidomethyl(14)	0.26	0.0690		
	ALENTAIPIGDLPEDK	0.24	0.0345		
	FFDAHPEYDGR	0.29	0.0362		
	FVDLVNEVVNK	0.24	0.0592		
	FYVDAVQLCPLR+Carbamidomethyl(9)	0.25	0.0676		
	GALIAIFDSGVDPAADGLQITSDGKPK	2.68	0.6665		
	GLALAEIESLK	0.08	0.0789		
	LANFMPLTNYR	0.26	0.1478		
	MLFQPGHIER	0.74	0.3772		
	SQGDLFEENFK	0.13	0.0668		
	VLDVIDCTGSGDIDTSK+Carbamidomethyl(7)	0.19	0.0467		
	VYSSGDVYPSSSNLPK	0.07	0.1267		
Glyma03g33830.1	Pyridoxal dependent decarboxylase family protein	6	0.49	0.0084	amino acid metabolism
	Peptide Sequnce	Ratio 4(2)F	Pvalue 4(2)F		
	DLIDTVR	0.51	0.3156		

	HGETFEDHIR	0.99	0.9848		
	INPDVDPQVHPYVATGNK	0.59	0.0987		
	LQWFLDAVK	0.28	0.0932		
	SEDGYLYCEGLK+Carbamidomethyl(8)	0.38	0.0336		
	SLIANTCCLVNR+Carbamidomethyl(7)Carbamidomethyl(8)	0.38	0.0163		
Glyma04g17050.1	decapping 5	3	0.49	0.0023	not assigned
	Peptide Sequnce	Ratio 4(2)F	Pvalue 4(2)F		
	FTEDFDFMAMNEK	1.73	0.4340		
	IDTETFGDFVR	0.47	0.0004		
	YEGILYNINTEESSIGLR	0.58	0.2091		
Glyma12g19265.1	RNA binding (RRM/RBD/RNP motifs) family protein	3	0.5	0.0045	RNA
	Peptide Sequnce	Ratio 4(2)F	Pvalue 4(2)F		
	GFALFVYK	0.51	0.0834		
	GLGWDTTTDGLR	0.53	0.0424		
	GYGFVTFR	0.04	0.6162		
Glyma05g31450.1	myo inositol 1 phosphate synthase 3	6	0.5	0.0494	minor CHO metabolism
	Peptide Sequnce	Ratio 4(2)F	Pvalue 4(2)F		
	ACVGLAPENNMILEYK+Carbamidomethyl(2)	0.72	0.6006		
	AMDEYTSEIFMGGK	0.27	0.2949		
	APLVPPGTPVVNALSK	0.64	0.5622		
	IQQANYFGSLTQASAIR	0.20	0.0507		
	SNVVDDMVNSNAILYEPGEHPDHVVVIK	0.45	0.3881		
	VVVLWTANTER	0.12	0.0136		
Glyma06g16410.1	Small nuclear ribonucleoprotein family protein	2	0.5	0.0253	RNA
	Peptide Sequnce	Ratio 4(2)F	Pvalue 4(2)F		
	IQIWLFEQK	0.31	0.0174		
	VMTQPINLIFR	0.66	0.2220		
Glyma13g01320.1	Small nuclear ribonucleoprotein family protein	2	0.5	0.0253	RNA
	Peptide Sequnce	Ratio 4(2)F	Pvalue 4(2)F		
	IQIWLFEQK	0.31	0.0174		
	VMTQPINLIFR	0.66	0.2220		
Glyma17g07460.1	Small nuclear ribonucleoprotein family protein	2	0.5	0.0253	RNA
	Peptide Sequnce	Ratio 4(2)F	Pvalue 4(2)F		
	IQIWLFEQK	0.31	0.0174		
	VMTQPINLIFR	0.66	0.2220		

Glyma07g02340.1	Pyridoxamine 5’ phosphate oxidase family protein	3	2.02	0.0180	not assigned
	Peptide Sequnce	Ratio 4(2)F	Pvalue 4(2)F		
	IAVDGGEQYLK	2.56	0.1080		
	ISFDEGQSVETLEEAK	2.43	0.1426		
	VTIFGDVYPLPEDQQEWAHK	1.83	0.0309		
Glyma08g23680.1	Pyridoxamine 5’ phosphate oxidase family protein	3	2.02	0.0180	not assigned
	Peptide Sequnce	Ratio 4(2)F	Pvalue 4(2)F		
	IAVDGGEQYLK	2.56	0.1080		
	ISFDEGQSVETLEEAK	2.43	0.1426		
	VTLFGDVYPLPEDQQEWAHK	1.83	0.0309		
Glyma09g27430.1	pfkB like carbohydrate kinase family protein	7	2.03	0.0163	major CHO metabolism
	Peptide Sequnce	Ratio 4(2)F	Pvalue 4(2)F		
	APGGAPANVAVGISR	3.78	0.0198		
	GIMDIWDQADVIK	1.16	0.8268		
	ISEDEITFLTGGDDPYDDNVVLK	15.45	0.1378		
	KAPGGAPANVAVGISR	5.08	0.0494		
	NPSADMLLQESELDK	6.80	0.1282		
	NSGCILSYDPNLR+Carbamidomethyl(4)	0.83	0.1104		
	TALAFVTLR	0.80	0.6552		
Glyma16g32530.1	pfkB like carbohydrate kinase family protein	7	2.03	0.0163	major CHO metabolism
	Peptide Sequnce	Ratio 4(2)F	Pvalue 4(2)F		
	APGGAPANVAVGISR	3.78	0.0198		
	GIMDIWDQADVIK	1.16	0.8268		
	ISEDEITFLTGGDDPYDDNVVLK	15.45	0.1378		
	KAPGGAPANVAVGISR	5.08	0.0494		
	NPSADMLLQESELDK	6.80	0.1282		
	NSGCILSYDPNLR+Carbamidomethyl(4)	0.83	0.1104		
	TALAFVTLR	0.80	0.6552		
Glyma06g16940.1	Transmembrane CLPTM1 family protein	2	2.04	0.0041	not assigned
	Peptide Sequnce	Ratio 4(2)F	Pvalue 4(2)F		
	APPTPAVEGAQAPQGR	2.17	0.0065		
	SLLGSSPDSSEAQVASK	0.28	0.0735		
Glyma15g30610.1	Calcium binding EF hand family protein	5	2.06	0.0187	signalling
	Peptide Sequnce	Ratio 4(2)F	Pvalue 4(2)F		
	GVVVIDGSTVR	1.56	0.2631		

	LYDSIFDK	1.83	0.0990		
	LYDSIFDKFDGDR	7.64	0.4015		
	MVLEDDPNSLLQK	2.43	0.0729		
	SVDEQFGVLDLNNDGVLSR	3.65	0.0301		
Glyma13g07220.1	glycosyl hydrolase family 81 protein	3	2.32	0.0149	stress
	Peptide Sequnce	Ratio 4(2)F	Pvalue 4(2)F		
	DSGDLLLLAHPLHVQLLR	9.94	0.0034		
	DTGLWFAPAEWK	3.76	0.2407		
	ELVEWTLPALDR	1.38	0.4493		
Glyma10g02730.1	Peroxidase superfamily protein	7	2.35	0.0093	stress
	Peptide Sequnce	Ratio 4(2)F	Pvalue 4(2)F		
	DSCPQAEDIIK+Carbamidomethyl(3)	2.00	0.2575		
	FFTEFAQSMK	1.41	0.1655		
	GLFQSDAALLTQEQSEDIAK	10.85	0.1456		
	MGAIEVLTGSAGEIR	11.79	0.0698		
	MHFHDCFVR+Carbamidomethyl(6)	1.51	0.4694		
	MHFHDCFVR+Carbamidomethyl(6)Oxidation(1)	3.34	0.0309		
	TVSCADILALAAR+Carbamidomethyl(4)	1.65	0.0666		
Glyma07g37240.2	MLP like protein 423	6	2.44	0.0470	stress
	Peptide Sequnce	Ratio 4(2)F	Pvalue 4(2)F		
	AIEAYLLAHPDYN	5.16	0.0071		
	ALVTDADNVIPK	2.08	0.5751		
	GVFTFEDEINSPVAPATLYK	5.69	0.0423		
	ITFLEDGETK	1.20	0.4891		
	KITFLEDGETK	1.58	0.3949		
	SVENVEGNGGPGTIK	0.96	0.7383		
Glyma01g44480.2	Nucleotide diphospho sugar transferases superfamily protein	7	2.49	0.0484	not assigned
	Peptide Sequnce	Ratio 4(2)F	Pvalue 4(2)F		
	EDLAYEVCR+Carbamidomethyl(8)	1.86	0.3468		
	GGPWFEAWK	1.59	0.3371		
	GQFESTEFSFSR	2.39	0.2068		
	MDGAVQTVYPR	1.80	0.2912		
	SSIPVEIIPIK	2.00	0.1882		
	TEAPLPSSNGGGAEIDNNEK	5.98	0.1300		
	VLTPDTVNTQTGAFLHR	5.82	0.0393		

Glyma11g03700.1	ATP binding	11	2.52	0.0194	protein
	Peptide Sequnce	Ratio 4(2)F	Pvalue 4(2)F		
	ALDVLNFTPLNNRPIR	1.12	0.5972		
	ALHDTFSTFGNILSCK+Carbamidomethyl(15)	1.05	0.9215		
	ELFSPFGTITSCK+Carbamidomethyl(12)	0.70	0.1566		
	GSGFVAFSTPDEASR	1.42	0.6285		
	GYGFVQFDNEESAQK	0.81	0.5468		
	LNGMLLNDK	0.80	0.7716		
	MVVSKPLYVTLAQR	5.76	0.4022		
	NLSESTTDDELK	0.37	0.0354		
	SLGYGYVNFSNPQDAAR	0.61	0.5016		
	VAEAMDVLR	0.92	0.7802		
	YQGANLYVK	0.89	0.8286		
Glyma13g05120.3	SPFH/Band 7/PHB domain containing membrane associated protein family	5	2.56	0.0152	not assigned
	Peptide Sequnce	Ratio 4(2)F	Pvalue 4(2)F		
	DSVLAFSENVPGTTAK	1.48	0.2204		
	EQIQSYVFDVIR	8.33	0.1263		
	LELDSVFEQK	3.23	0.0739		
	SSSVFIPHGPGAVK	3.13	0.1118		
	YLSGLGIAR	1.59	0.0732		
Glyma17g03365.1	MLP like protein 423	3	2.57	0.0283	stress
	Peptide Sequnce	Ratio 4(2)F	Pvalue 4(2)F		
	ALVTDADNIIPK	0.48	0.0386		
	GIFTFEDEITSPVAPATLYK	6.37	0.0301		
	SVENVEGNGGPGTIK	0.96	0.7383		
Glyma06g46190.2	aconitase 3	10	2.62	0.0359	TCA / org transformation
	Peptide Sequnce	Ratio 4(2)F	Pvalue 4(2)F		
	DAYCLLNFGDSITTDHISPAGSIHK+Carbamidomethyl(4)	1.03	0.9892		
	FDFHGQPAELK	0.56	0.3025		
	FVEFYGDGMGELSLADR	0.69	0.6808		
	FYSLPSLNDPR	0.45	0.0180		
	GPMLLGVK	0.61	0.4405		
	ILLESAIR	0.52	0.2610		
	LFVDYNEPQQDR	19.67	0.0351		
	LYVFDAATR	0.81	0.7220		

	TSLAPGSGVVTK	0.42	0.1029		
	YLLQSGLQK	0.21	0.0704		
Glyma12g10580.1	aconitase 3	10	2.62	0.0359	TCA / org transformation
	Peptide Sequnce	Ratio 4(2)F	Pvalue 4(2)F		
	DAYCLLNFGDSITTDHISPAGSIHK+Carbamidomethyl(4)	1.03	0.9892		
	FDFHGQPAELK	0.56	0.3025		
	FVEFYGDGMGELSLADR	0.69	0.6808		
	FYSLPSLNDPR	0.45	0.0180		
	GPMLLGVK	0.61	0.4405		
	ILLESAIR	0.52	0.2610		
	LFVDYNEPQQDR	19.67	0.0351		
	LYVFDAATR	0.81	0.7220		
	TSLAPGSGVVTK	0.42	0.1029		
	YLLQSGLQK	0.21	0.0704		
Glyma09g04337.1	beta xylosidase 1	3	2.66	0.0052	cell wall
	Peptide Sequnce	Ratio 4(2)F	Pvalue 4(2)F		
	AGLDLDCGPFLAIHTDSAIR+Carbamidomethyl(7)	0.48	0.0352		
	LGIQGYEWWSEALHGVSNVGPGTK	13.74	0.0599		
	LVVNNAIAVPR	0.35	0.0473		
Glyma15g15370.1	beta xylosidase 1	3	2.66	0.0052	cell wall
	Peptide Sequnce	Ratio 4(2)F	Pvalue 4(2)F		
	AGLDLDCGPFLAIHTDSAIR+Carbamidomethyl(7)	0.48	0.0352		
	LGIQGYEWWSEALHGVSNVGPGTK	13.74	0.0599		
	LVVNNAIAVPR	0.35	0.0473		
Glyma01g34410.2	AT hook motif nuclear localized protein 1	3	2.66	0.0435	RNA
	Peptide Sequnce	Ratio 4(2)F	Pvalue 4(2)F		
	FEILSLSGSFMPTDNQGTR	7.41	0.1071		
	TGGMSVSLASPDGR	1.39	0.4971		
	VISFSQQGPR	0.49	0.0286		
Glyma03g02670.5	AT hook motif nuclear localized protein 1	3	2.66	0.0435	RNA
	Peptide Sequnce	Ratio 4(2)F	Pvalue 4(2)F		
	FEILSLSGSFMPTDNQGTR	7.41	0.1071		
	TGGMSVSLASPDGR	1.39	0.4971		
	VISFSQQGPR	0.49	0.0286		
Glyma05g06420.1	glyceraldehyde 3 phosphate dehydrogenase C2	8	2.66	0.0216	glycolysis

	Peptide Sequnce	Ratio 4(2)F	Pvalue 4(2)F		
	DAPMFVVGVNEK	2.72	0.0217		
	GILGYTEDDVVSTDFVGDNR	1.84	0.1774		
	IGINGFGR	1.15	0.9258		
	LTGMSFR	2.75	0.3315		
	LTGMSFRVPTVDVSVVDLTVR	7.40	0.0080		
	VIISAPSK	23.97	0.1262		
	VPTVDVSVVDLTVR	0.95	0.9382		
	VPTVDVSVVDLTVRLEK	19.29	0.3757		
Glyma19g22780.1	glyceraldehyde 3 phosphate dehydrogenase C2	8	2.66	0.0216	glycolysis
	Peptide Sequnce	Ratio 4(2)F	Pvalue 4(2)F		
	DAPMFVVGVNEK	2.72	0.0217		
	GILGYTEDDVVSTDFVGDNR	1.84	0.1774		
	IGINGFGR	1.15	0.9258		
	LTGMSFR	2.75	0.3315		
	LTGMSFRVPTVDVSVVDLTVR	7.40	0.0080		
	VIISAPSK	23.97	0.1262		
	VPTVDVSVVDLTVR	0.95	0.9382		
	VPTVDVSVVDLTVRLEK	19.29	0.3757		
Glyma15g08840.1	xylem bark cysteine peptidase 3	2	2.78	0.0046	protein
	Peptide Sequnce	Ratio 4(2)F	Pvalue 4(2)F		
	ALDWVIGNR	2.245052637	0.007640609		
	GIASEIDYPYTAR	25.08755699	0.07122905		
Glyma11g02950.1	hydroxymethylglutaryl CoA synthase	6	2.79	0.0000	secondary metabolism
	Peptide Sequnce	Ratio 4(2)F	Pvalue 4(2)F		
	LEVGSETVIDK	0.46	0.0702		
	LGPFATLSGDESYQSR	0.32	0.0035		
	LVFNDFLMNSSSVDEVAK	0.40	0.1134		
	QFSLSDAEYFVFHSPYNK	28.53	0.0018		
	TFLMQIFEK	0.20	0.0722		
	VQPTTLIPK	0.25	0.0276		
Glyma17g03350.1	N.D. *	7	2.79	0.0226	not assigned
	Peptide Sequnce	Ratio 4(2)F	Pvalue 4(2)F		
	ALVTDADNVIPK	2.08	0.5751		
	AVEAYLLANPHYN	2.38	0.3344		

	GDAQPNPDDLK	20.25	0.1710		
	GIFTFEDETTSPVAPATLYK	2.69	0.0469		
	IESVDEANLGYSYSVVGGVGLPDTVEK	8.61	0.1509		
	ITFVEDGESK	1.47	0.2138		
	SVENLEGNGGPGTIK	3.15	0.0361		
Glyma09g40520.5	AT hook motif nuclear localized protein 1	2	2.89	0.0208	RNA
	Peptide Sequnce	Ratio 4(2)F	Pvalue 4(2)F		
	FEILSLSGSFMPTDNQGTR	7.41	0.1071		
	VISFSQQGPR	0.49	0.0286		
Glyma05g34320.1	26S proteasome regulatory complex non ATPase subcomplex	13	2.89	0.0194	protein
	Peptide Sequnce	Ratio 4(2)F	Pvalue 4(2)F		
	AIDEYASLK	0.50	0.1095		
	ANAEISSAPDSASVAPSGGK	6.56	0.0013		
	EEEADTLIEQMTR	0.55	0.2423		
	ENLDWLSR	0.34	0.1771		
	FSATAGLGVIHR	0.57	0.3849		
	IESLHEDEEFDQHQR	0.46	0.1874		
	IVSLLSESYNPHVR	0.69	0.6493		
	LPTAVLSTSAK	0.41	0.1597		
	MGAILASGILDAGGR	2.43	0.2474		
	QLAALLVSK	0.45	0.3577		
	SDLLILK	0.40	0.3484		
	TSEPEPSFEILTNPAR	2.62	0.4668		
	YGGMYALALAYR	0.73	0.6948		
Glyma15g13500.1	Peroxidase superfamily protein	4	2.98	0.0149	misc
	Peptide Sequnce	Ratio 4(2)F	Pvalue 4(2)F		
	GLDVVNDIK	2.59	0.1416		
	GLLQSDQELFSTPGADTIPIVNR	3.46	0.0372		
	MGNIGVLTGK	4.47	0.0159		
	VYFSNLQVK	1.08	0.9010		
Glyma07g11810.1	glutamine synthase clone R1	7	3.04	0.0441	N-metabolism
	Peptide Sequnce	Ratio 4(2)F	Pvalue 4(2)F		
	DIVDAHYK	2.28	0.5210		
	EHIAAYGEGNER	9.21	0.0183		
	GNNILVICDVYTPAGEPLPTNK+Carbamidomethyl(8)	1.39	0.2289		

	HETADINTFSWGVANR	1.56	0.2944		
	IIAEYIWVGGSGMDLR	1.38	0.0841		
	RGNNILVICDVYTPAGEPLPTNK+Carbamidomethyl(9)	1.90	0.1702		
	TLPGPVSDPAK	1.64	0.6246		
Glyma20g29270.1	pfkB like carbohydrate kinase family protein	6	3.1	0.0264	major CHO metabolism
	Peptide Sequnce	Ratio 4(2)F	Pvalue 4(2)F		
	APGGAPANVAVGISR	3.78	0.0198		
	ESGCILSYDPNLR+Carbamidomethyl(4)	0.93	0.8264		
	ISEDEITFLTGGDDPYDDNVVLK	15.45	0.1378		
	KAPGGAPANVAVGISR	5.08	0.0494		
	KAPGGAPANVAVGISR	5.08	0.0494		
	NPSADMLLQESELDK	6.80	0.1282		
	TALAFVTLR	0.80	0.6552		
Glyma10g28900.1	Adenine nucleotide alpha hydrolases like superfamily protein	4	3.13	0.0318	hormone metabolism
	Peptide Sequnce	Ratio 4(2)F	Pvalue 4(2)F		
	AFLGSVSNHCAQNVK+Carbamidomethyl(10)	0.61	0.1237		
	DVICQMVQK+Carbamidomethyl(4)	3.48	0.1838		
	LGADVLVMGSHGYGLIK	4.49	0.2343		
	YSQQVADCVLEK+Carbamidomethyl(8)	3.38	0.0414		
Glyma20g23090.1	Adenine nucleotide alpha hydrolases like superfamily protein	4	3.13	0.0318	hormone metabolism
	Peptide Sequnce	Ratio 4(2)F	Pvalue 4(2)F		
	AFLGSVSNHCAQNVK+Carbamidomethyl(10)	0.61	0.1237		
	DVICQMVQK+Carbamidomethyl(4)	3.48	0.1838		
	LGADVLVMGSHGYGLIK	4.49	0.2343		
	YSQQVADCVLEK+Carbamidomethyl(8)	3.38	0.0414		
Glyma19g02370.4	SPFH/Band 7/PHB domain containing membrane associated protein family	4	3.14	0.0151	not assigned
	Peptide Sequnce	Ratio 4(2)F	Pvalue 4(2)F		
	EQIQSYVFDVIR	8.33	0.1263		
	LELDSVFEQK	3.23	0.0739		
	SSSVFIPHGPGAVK	3.13	0.1118		
	YLSGLGIAR	1.59	0.0732		
Glyma03g26060.1	uclacyanin 1	4	3.47	0.0326	misc
	Peptide Sequnce	Ratio 4(2)F	Pvalue 4(2)F		
	IELTSPGK	11.87	0.0179		
	TFTVGDTLVFK	0.96	0.7939		

	YDSTHQVDEVDESGYNSCSSSNSIK+Carbamidomethyl(18)	0.86	0.1435		
	YFLCPISGHCAGGMK+Carbamidomethyl(4)Carbamidomethyl(10)	2.10	0.1120		
Glyma08g20490.2	Glycosyl hydrolases family 32 protein	2	3.53	0.0014	major CHO metabolism
	Peptide Sequnce	Ratio 4(2)F	Pvalue 4(2)F		
	DAFIPDEESNIFVLR	0.18	0.0638		
	TLIDHSVVESFGGEGR	17.74	0.0023		
Glyma08g08520.1	FAD binding Berberine family protein	7	3.6	0.0011	misc
	Peptide Sequnce	Ratio 4(2)F	Pvalue 4(2)F		
	DLDIGTNSFGK	1.86	0.1549		
	ESMGEDLFWAIR	6.73	0.0508		
	LVPVPEVVSVFR	11.02	0.2141		
	MSEVSSDATPFPHR	17.92	0.0101		
	NSYAEGAVYGVK	1.32	0.6399		
	TEVDPENFFR	2.04	0.1053		
	TGFVFNPYGGK	2.28	0.0134		
Glyma05g09290.4	actin 11	15	3.69	0.0237	cell
	Peptide Sequnce	Ratio 4(2)F	Pvalue 4(2)F		
	AEYDESGPSIVHR	0.67	0.3332		
	AGFAGDDAPR	126.52	0.0255		
	AVFPSIVGRPR	2.62	0.5911		
	DAYVGDEAQSK	28.55	0.1173		
	DLTDFLMK	0.57	0.3613		
	EISALAPSSMK	0.81	0.6994		
	GILTLKYPIEHGIVSNWDDMEK	11.94	0.1190		
	GYSFTTSAER	0.43	0.0423		
	HTGVMVGMGQK	16.58	0.0527		
	IWHHTFYNELR	0.55	0.2742		
	LAYIALDYEQELETSK	0.19	0.1122		
	SYELPDGQVITIGAER	0.72	0.0779		
	TTGIVLDSGDGVSHTVPIYEGYALPHAILR	0.80	0.6827		
	VAPEEHPVLLTEAPLNPK	1.16	0.8653		
	YPIEHGIVSNWDDMEK	1.06	0.9942		
Glyma19g00850.1	actin 11	15	3.69	0.0237	cell
	Peptide Sequnce	Ratio 4(2)F	Pvalue 4(2)F		
	AEYDESGPSIVHR	0.67	0.3332		

	AGFAGDDAPR	126.52	0.0255		
	AVFPSIVGRPR	2.62	0.5911		
	DAYVGDEAQSK	28.55	0.1173		
	DLTDFLMK	0.57	0.3613		
	EISALAPSSMK	0.81	0.6994		
	GILTLKYPIEHGIVSNWDDMEK	11.94	0.1190		
	GYSFTTSAER	0.43	0.0423		
	HTGVMVGMGQK	16.58	0.0527		
	IWHHTFYNELR	0.55	0.2742		
	LAYIALDYEQELETSK	0.19	0.1122		
	SYELPDGQVITIGAER	0.72	0.0779		
	TTGIVLDSGDGVSHTVPIYEGYALPHAILR	0.80	0.6827		
	VAPEEHPVLLTEAPLNPK	1.16	0.8653		
	YPIEHGIVSNWDDMEK	1.06	0.9942		
Glyma03g39440.1	Calcineurin like metallo phosphoesterase superfamily protein	2	3.82	0.0452	protein
	Peptide Sequnce	Ratio 4(2)F	Pvalue 4(2)F		
	QSLETICLLLAYK+Carbamidomethyl(7)	20.27	0.1186		
	QVQLSESEIR	0.41	0.0651		
Glyma19g42050.1	Calcineurin like metallo phosphoesterase superfamily protein	2	3.82	0.0452	protein
	Peptide Sequnce	Ratio 4(2)F	Pvalue 4(2)F		
	QSLETICLLLAYK+Carbamidomethyl(7)	20.27	0.1186		
	QVQLSESEIR	0.41	0.0651		
Glyma09g24410.1	heat shock protein 90.1	7	3.93	0.0026	stress
	Peptide Sequnce	Ratio 4(2)F	Pvalue 4(2)F		
	ADLVNNLGTIAR	0.18	0.0636		
	APFDLFDTR	0.02	0.1254		
	EDQLEYLEER	0.10	0.0539		
	ELISNASDALDK	34.23	0.1609		
	HFSVEGQLEFK	0.04	0.0916		
	RAPFDLFDTR	0.02	0.0870		
	SLTNDWEEHLAVK	0.28	0.1058		
Glyma16g29750.1	heat shock protein 90.1	7	3.93	0.0026	stress
	Peptide Sequnce	Ratio 4(2)F	Pvalue 4(2)F		
	ADLVNNLGTIAR	0.18	0.0636		
	APFDLFDTR	0.02	0.1254		

	EDQLEYLEER	0.10	0.0539		
	ELISNASDALDK	34.23	0.1609		
	HFSVEGQLEFK	0.04	0.0916		
	RAPFDLFDTR	0.02	0.0870		
	SLTNDWEEHLAVK	0.28	0.1058		
Glyma01g34770.1	xyloglucan endotransglucosylase/hydrolase 26	2	3.98	0.0446	cell wall
	Peptide Sequnce	Ratio 4(2)F	Pvalue 4(2)F		
	NYENEGIAYPNK	0.34	0.0557		
	VYTSLWNADDWATR	5.74	0.1361		
Glyma09g32630.1	xyloglucan endotransglucosylase/hydrolase 26	2	3.98	0.0446	cell wall
	Peptide Sequnce	Ratio 4(2)F	Pvalue 4(2)F		
	NYENEGIAYPNK	0.34	0.0557		
	VYTSLWNADDWATR	5.74	0.1361		
Glyma09g40830.1	Protein of unknown function (DUF3411)	4	4	0.0483	not assigned
	Peptide Sequnce	Ratio 4(2)F	Pvalue 4(2)F		
	ENEVALIAEK	0.83	0.3482		
	VAADPQFPFK	0.26	0.0790		
	VLNPLAFK	0.33	0.1042		
	VVESEREEGLQNNQSTAP	12.41	0.1422		
Glyma05g25540.1	FAD binding Berberine family protein	5	4.07	0.0004	misc
	Peptide Sequnce	Ratio 4(2)F	Pvalue 4(2)F		
	DLDIGTNSFGK	1.86	0.1549		
	ESMGEDLFWAIR	6.73	0.0508		
	MSEVSSDATPFPHR	17.92	0.0101		
	TEVDPENFFR	2.04	0.1053		
	TGFVFNPYGGK	2.28	0.0134		
Glyma13g34520.1	D mannose binding lectin protein	6	4.13	0.0153	misc
	Peptide Sequnce	Ratio 4(2)F	Pvalue 4(2)F		
	ENVDGPYSLVLEPK	4.95	0.0340		
	FENSGELGPYIVEYGADYR	3.06	0.1165		
	FYTYFLDVR	3.36	0.3023		
	GLALYYK	6.48	0.0442		
	LGIDGNIR	0.22	0.3618		
	WVWEANR	5.13	0.2046		
Glyma19g39270.2	Peroxidase superfamily protein	4	4.15	0.0051	misc

	Peptide Sequnce	Ratio 4(2)F	Pvalue 4(2)F		
	GDQDPSLNPTYANFLK	7.93	0.0182		
	GLFQSDAALLTTK	6.50	0.0669		
	MHFHDCFVR+Carbamidomethyl(6)	1.51	0.4694		
	MHFHDCFVR+Carbamidomethyl(6)Oxidation(1)	3.34	0.0309		
Glyma17g14150.1	hydroxymethylglutaryl CoA synthase /	5	4.22	0.0000	secondary metabolism
	Peptide Sequnce	Ratio 4(2)F	Pvalue 4(2)F		
	LEVGSETVIDK	0.46	0.0702		
	LVFNDFLK	0.20	0.0105		
	QFSISDAEYFVFHSPYNK	28.53	0.0018		
	TFIMQIFEK	0.20	0.0722		
	VQPTTLIPK	0.25	0.0276		
Glyma15g15200.1	Glycosyl hydrolase superfamily protein	4	4.41	0.0011	misc
	Peptide Sequnce	Ratio 4(2)F	Pvalue 4(2)F		
	HFGLFNPNK	2.24	0.2838		
	LYDPNQAALEALR	1.05	0.8569		
	NVLNFWPSVK	6.63	0.0004		
	VYLDNLVR	7.80	0.0243		
Glyma09g32080.1	Leucine rich repeat (LRR) family protein	2	4.57	0.0132	cell wall
	Peptide Sequnce	Ratio 4(2)F	Pvalue 4(2)F		
	FNEFEGTVPK	4.49	0.0131		
	FPDVVLR	4.74	0.1373		
Glyma13g00790.1	Peroxidase superfamily protein	9	4.59	0.0239	misc
	Peptide Sequnce	Ratio 4(2)F	Pvalue 4(2)F		
	DVVNLAGGPFYNVELGR	105.01	0.0477		
	FDNQYFK	5.51	0.1296		
	FQQTFVTAPATLR	2.55	0.1824		
	GLFTSDQVLFTDAR	1.87	0.2490		
	IAINMDPVTPQK	5.90	0.0891		
	IDPTLNLQYAFQLR	0.66	0.3571		
	LFFHDCFVR+Carbamidomethyl(6)	6.56	0.1561		
	NTCPNVEQLVR+Carbamidomethyl(3)	1.40	0.1966		
	VSCADILALATR+Carbamidomethyl(3)	1.40	0.4796		
Glyma17g06890.1	Peroxidase superfamily protein	9	4.59	0.0239	misc
	Peptide Sequnce	Ratio 4(2)F	Pvalue 4(2)F		

	DVVNLAGGPFYNVELGR	105.01	0.0477		
	FDNQYFK	5.51	0.1296		
	FQQTFVTAPATLR	2.55	0.1824		
	GLFTSDQVLFTDAR	1.87	0.2490		
	IAINMDPVTPQK	5.90	0.0891		
	IDPTLNLQYAFQLR	0.66	0.3571		
	LFFHDCFVR+Carbamidomethyl(6)	6.56	0.1561		
	NTCPNVEQLVR+Carbamidomethyl(3)	1.40	0.1966		
	VSCADILALATR+Carbamidomethyl(3)	1.40	0.4796		
Glyma02g03320.1	Matrixin family protein	4	4.62	0.0015	protein
	Peptide Sequnce	Ratio 4(2)F	Pvalue 4(2)F		
	FSNLPVTGVPNK	3.31	0.0524		
	LFGPALAK	2.48	0.0745		
	MEVYGGSLIFLQPDSSK	3.72	0.1351		
	QLIQQMLSLR	5.13	0.0070		
Glyma09g16690.1	Chaperone protein htpG family protein	4	5.03	0.0120	stress
	Peptide Sequnce	Ratio 4(2)F	Pvalue 4(2)F		
	ELISNASDALDK	34.23	0.1609		
	FEFQAEVSR	0.05	0.0784		
	FLSLTDK	0.05	0.0521		
	LMDIIINSLYSNK	0.04	0.0848		
Glyma01g04350.1	Matrixin family protein	3	5.89	0.0011	protein
	Peptide Sequnce	Ratio 4(2)F	Pvalue 4(2)F		
	GVILLDGTNK	3.25	0.0088		
	LWALPSENGR	2.11	0.1181		
	TYQQYFSLQPTGK	18.81	0.0138		
Glyma09g04191.1	Glycosyl hydrolase superfamily protein	2	6.08	0.0011	misc
	Peptide Sequnce	Ratio 4(2)F	Pvalue 4(2)F		
	HFGLFNPNK	2.24	0.2838		
	NVLNFWPSVK	6.63	0.0004		
Glyma02g00490.1	Ribosomal protein L7Ae/L30e/S12e/Gadd45 family protein	5	6.25	0.0033	protein
	Peptide Sequnce	Ratio 4(2)F	Pvalue 4(2)F		
	LIIIANNCPPLR+Carbamidomethyl(8)	0.19	0.0418		
	LIIIANNCPPLRK+Carbamidomethyl(8)	0.46	0.3327		
	SEIEYYAMLAK	0.31	0.1575		

	VCCLSIIDPGDSDIIK+Carbamidomethyl(2)Carbamidomethyl(3)	0.23	0.0303		
	VGVHHYNGNNVDLGTACGK+Carbamidomethyl(17)	10.42	0.0284		
Glyma08g28800.1	Ribosomal protein L7Ae/L30e/S12e/Gadd45 family protein	5	6.25	0.0033	protein
	Peptide Sequnce	Ratio 4(2)F	Pvalue 4(2)F		
	LIIIANNCPPLR+Carbamidomethyl(8)	0.19	0.0418		
	LIIIANNCPPLRK+Carbamidomethyl(8)	0.46	0.3327		
	SEIEYYAMLAK	0.31	0.1575		
	VCCLSIIDPGDSDIIK+Carbamidomethyl(2)Carbamidomethyl(3)	0.23	0.0303		
	VGVHHYNGNNVDLGTACGK+Carbamidomethyl(17)	10.42	0.0284		
Glyma18g51660.1	Ribosomal protein L7Ae/L30e/S12e/Gadd45 family protein	5	6.25	0.0033	protein
	Peptide Sequnce	Ratio 4(2)F	Pvalue 4(2)F		
	LIIIANNCPPLR+Carbamidomethyl(8)	0.19	0.0418		
	LIIIANNCPPLRK+Carbamidomethyl(8)	0.46	0.3327		
	SEIEYYAMLAK	0.31	0.1575		
	VCCLSIIDPGDSDIIK+Carbamidomethyl(2)Carbamidomethyl(3)	0.23	0.0303		
	VGVHHYNGNNVDLGTACGK+Carbamidomethyl(17)	10.42	0.0284		
Glyma11g12960.1	hemoglobin 1	2	6.37	0.0338	redox
	Peptide Sequnce	Ratio 4(2)F	Pvalue 4(2)F		
	EAVPEMWSPAMK	12.58	0.1003		
	GFTEEQEALVVK	3.80	0.0543		
Glyma15g07940.1	Rubber elongation factor protein (REF)	4	6.8	0.0051	not assigned
	Peptide Sequnce	Ratio 4(2)F	Pvalue 4(2)F		
	VAELDSHVPSNVK	5.338946828	0.188276783		
	VSAYLPLVPTEK	12.67294083	0.0313344		
	VSSQACSVVSEVR+Carbamidomethyl(6)	1.488782677	0.227700055		
	YEPTAEQCAVSAWR+Carbamidomethyl(8)	14.13130156	0.012921354		
Glyma18g43460.1	pyruvate decarboxylase 2	11	6.87	0.0265	fermentation
	Peptide Sequnce	Ratio 4(2)F	Pvalue 4(2)F		
	AIIVQPDR	5.10	0.3260		
	DSLCFIEVIVHK+Carbamidomethyl(4)	5.64	0.1986		
	DSLCFIEVIVHKDDTSK+Carbamidomethyl(4)	0.83	0.5819		
	ELLEWGSR	5.65	0.0374		
	ESKPVYISISCNLPGIPHPTFSR+Carbamidomethyl(11)	11.90	0.1693		
	IFVPDGHPLK	1.93	0.1622		
	ILHHTIGLPDFSQELR	12.65	0.1276		

	KDSLCFIEVIVHK+Carbamidomethyl(5)	5.47	0.3210		
	NWNYTGLIDAIHNGEGK	11.15	0.0959		
	VSAANSRPPNPQ	29.57	0.2477		
	VVIANGPAFGCVLMK+Carbamidomethyl(11)	6.08	0.3075		
Glyma09g28100.1	Enolase	17	8.18	0.0000	glycolysis
	Peptide Sequnce	Ratio 4(2)F	Pvalue 4(2)F		
	AAVPSGASTGVYEALELR	6.67	0.0226		
	ACNALLLK+Carbamidomethyl(2)	0.44	0.0603		
	AVENVNSIIAPALLGK	9.59	0.0373		
	DGGSDYLGK	42.89	0.2505		
	GNPTVEVDVILSDGSFHR	9.39	0.0000		
	IEEELGSAAVYAGAK	4.13	0.2064		
	LGANAILAVSLAVCK+Carbamidomethyl(14)	22.12	0.1125		
	LGANAILAVSLAVCKAGAAVK+Carbamidomethyl(14)	0.39	0.1826		
	LTAEVGQQVQIVGDDLLVTNPK	16.24	0.0214		
	MGVEVYHHLK	10.44	0.0969		
	TLVLPVPSFNVINGGSHAGNK	7.76	0.0855		
	TYDLNFK	1.06	0.8949		
	TYDLNFKEENNDGSQK	75.67	0.3227		
	VNQIGSVTESIEAVR	1.05	0.4198		
	VVIGMDVAASEFYDNK	18.86	0.0523		
	YGQDATNVGDEGGFAPNIQENQEGLELLK	11.06	0.0359		
	YNQLLR	10.83	0.1177		
Glyma10g05580.1	Ribosomal protein L4/L1 family	2	8.22	0.0339	protein
	Peptide Sequnce	Ratio 4(2)F	Pvalue 4(2)F		
	IINSDEVQSVVRPIK	0.24	0.2857		
	NVPGVEVANVER	13.98	0.0191		
Glyma13g19930.1	Ribosomal protein L4/L1 family	2	8.22	0.0339	protein
	Peptide Sequnce	Ratio 4(2)F	Pvalue 4(2)F		
	IINSDEVQSVVRPIK	0.24	0.2857		
	NVPGVEVANVER	13.98	0.0191		
Glyma03g36610.1	Peroxidase superfamily protein	3	8.25	0.0046	misc
	Peptide Sequnce	Ratio 4(2)F	Pvalue 4(2)F		
	GDQDPSLNPTYANFLK	7.93	0.0182		
	GLFQSDAALLTTK	6.50	0.0669		

	MGAIEVLTGSAGEIR	11.79	0.0698		
Glyma10g00920.1	Lactate/malate dehydrogenase family protein	12	8.54	0.0119	TCA / org transformation
	Peptide Sequnce	Ratio 4(2)F	Pvalue 4(2)F		
	ALGQISER	156.46	0.2209		
	EFAPSIPEK	0.80	0.6481		
	ELIADDAWLNGEFITTVQQR	6.09	0.4689		
	KLDLTAEELSEEK	0.81	1.2849		
	LDLTAEELSEEK	1.09	0.8921		
	LNIQVSDVK	0.49	0.0902		
	LSSALSAASAACDHIR+Carbamidomethyl(12)	0.66	0.5984		
	MELVDAAFPLLK	0.02	0.1280		
	MELVDAAFPLLK+Oxidation(1)	0.48	0.0289		
	NISCLTR+Carbamidomethyl(4)	24.02	0.0864		
	NVIIWGNHSSTQYPDVNHATVGEKPVR	7.08	0.0512		
	VLVVANPANTNALILK	0.52	0.0300		
Glyma07g18570.1	Thiamine pyrophosphate dependent pyruvate decarboxylase family protein	13	11.9	0.0027	fermentation
	Peptide Sequnce	Ratio 4(2)F	Pvalue 4(2)F		
	AIIVQPDR	5.10	0.3260		
	DPVPFSLSPR	17.39	0.0799		
	DSLCFIEVIVHK+Carbamidomethyl(4)	5.64	0.1986		
	DSLCFIEVIVHKDDTSK+Carbamidomethyl(4)	0.83	0.5819		
	ELLEWGSR	5.65	0.0374		
	ESKPVYISISCNLPGIPHPTFSR+Carbamidomethyl(11)	11.90	0.1693		
	IFVPEGHPLK	174.44	0.0959		
	ILHHTIGLPDFSQELR	12.65	0.1276		
	MLSGETAVIAETGDSWFNCQK+Carbamidomethyl(19)	16.93	0.0279		
	NWNYTGLIDAIHNGEGK	11.15	0.0959		
	VNVLFQHIQK	7.69	0.2225		
	VSAANSRPPNPQ	29.57	0.2477		
	VVIANGPAFGCVLMK+Carbamidomethyl(11)	6.08	0.3075		
Glyma08g03310.1	ACC oxidase 1	3	12.28	0.0218	hormone metabolism
	Peptide Sequnce	Ratio 4(2)F	Pvalue 4(2)F		
	LLYPSNFR	7.51	0.0569		
	LSELMSENLGLEK	5.09	0.2956		
	YPQCPRPELVR+Carbamidomethyl(4)	17.32	0.0362		

Glyma17g07250.1	xyloglucan endotransglycosylase 6	5	12.45	0.0211	cell wall
	Peptide Sequnce	Ratio 4(2)F	Pvalue 4(2)F		
	ILNNENLLTLSLDK	11.34	0.0979		
	IVFSVDGTPIR	0.38	0.1826		
	IYSSLWNADDWATR	17.21	0.0809		
	LVPGNSAGTVTAYYLSSK	8.83	0.3996		
	NLESIGVPFPK	32.43	0.0477		
Glyma13g31390.1	Rubber elongation factor protein (REF)	2	12.85	0.0036	not assigned
	Peptide Sequnce	Ratio 4(2)F	Pvalue 4(2)F		
	VSAYLPLVPTEK	12.67	0.0313		
	YEPTAEQCAVSAWR+Carbamidomethyl(8)	14.13	0.0129		
Glyma02g16710.1	Eukaryotic aspartyl protease family protein	7	13.7	0.0098	protein
	Peptide Sequnce	Ratio 4(2)F	Pvalue 4(2)F		
	DASTLQYITQIK	20.61	0.0333		
	IALPSQLASAFSFR	26.26	0.2615		
	IFGANSMVSVSDDK	7.70	0.2190		
	LGAAVPTIELVLQNQK	18.38	0.1548		
	LGFSSLLYGSR	11.23	0.1520		
	VASVAPFEVCFSR+Carbamidomethyl(10)	1.50	0.5554		
	VLCLGFVNGGENPR+Carbamidomethyl(3)	6.32	0.0183		
Glyma15g39370.2	glyoxalase II 3	4	13.71	0.0180	bodegradation of Xenobiotics
	Peptide Sequnce	Ratio 4(2)F	Pvalue 4(2)F		
	DLSLIEQLGLK	12.50	0.1350		
	GCVTYVTGDAPDQPQPR+Carbamidomethyl(2)	15.13	0.0217		
	MAFTGDTLLIR	10.77	0.0760		
	TVDRDLSLIEQLGLK	2.01	0.4398		
Glyma11g35600.1	Eukaryotic translation initiation factor 2 subunit 1	5	13.75	0.0010	protein
	Peptide Sequnce	Ratio 4(2)F	Pvalue 4(2)F		
	CFQFDGVLHIK+Carbamidomethyl(1)	0.44	0.1578		
	EQGILVLNNAIASCTEAIEQHK+Carbamidomethyl(14)	0.18	0.0695		
	IEPVMVLR	0.25	0.1323		
	IIVTDPDTVLSTLTR	0.25	0.0138		
	VSEEDIQACEER+Carbamidomethyl(9)	36.40	0.0179		
Glyma18g02820.1	Eukaryotic translation initiation factor 2 subunit 1	5	13.75	0.0010	protein
	Peptide Sequnce	Ratio 4(2)F	Pvalue 4(2)F		

	CFQFDGVLHIK+Carbamidomethyl(1)	0.44	0.1578		
	EQGILVLNNAIASCTEAIEQHK+Carbamidomethyl(14)	0.18	0.0695		
	IEPVMVLR	0.25	0.1323		
	IIVTDPDTVLSTLTR	0.25	0.0138		
	VSEEDIQACEER+Carbamidomethyl(9)	36.40	0.0179		
Glyma05g28310.2	xyloglucan endotransglucosylase/hydrolase 16	4	13.98	0.0188	cell wall
	Peptide Sequnce	Ratio 4(2)F	Pvalue 4(2)F		
	APFTAYYR	7.13	0.1040		
	EQQFYLWFDPTR	5.46	0.4612		
	IYSSLWNADDWATR	17.21	0.0809		
	NAESLGVPFPK	26.18	0.0268		
Glyma05g36310.1	ACC oxidase 1	6	17.65	0.0022	hormone metabolism
	Peptide Sequnce	Ratio 4(2)F	Pvalue 4(2)F		
	AFSGNGEGPAVGTK	11.49	0.3867		
	LLYPSNFR	7.51	0.0569		
	LSELMSENLGLEK	5.09	0.2956		
	QLINAYYEENLK	29.86	0.0484		
	WGCFMVENHEIDTQLMGK+Carbamidomethyl(3)	120.04	0.0189		
	YPQCPRPELVR+Carbamidomethyl(4)	17.32	0.0362		
Glyma08g08360.1	polygalacturonase inhibiting protein 1	6	17.68	0.0016	cell wall
	Peptide Sequnce	Ratio 4(2)F	Pvalue 4(2)F		
	CLCGSPLPK+Carbamidomethyl(1)Carbamidomethyl(3)	8.24	0.0181		
	EALLQIK	24.25	0.3134		
	ISGTIPDSFGSFSDSLK	18.71	0.0130		
	LTGEIPATLAK	16.16	0.0457		
	LYGALPEGLTSLK	64.97	0.1856		
	TLLILYLSHNR	5.97	0.2253		
Glyma04g09670.1	Rhamnogalacturonate lyase family protein	6	17.72	0.0069	cell wall
	Peptide Sequnce	Ratio 4(2)F	Pvalue 4(2)F		
	DGPTLWEIGIPDR	42.23	0.1432		
	DWFFAQVTR	16.25	0.1894		
	GAYVGLAPPGDVGSWQR	7.17	0.0469		
	GNGPFQAIMYDYIR	102.20	0.0959		
	SAAEFYVPDPNPK	24.54	0.0217		
	YINDDYVSAK	0.58	0.4414		

Glyma16g33760.1	Kunitz family trypsin and protease inhibitor protein	3	24.95	0.0327	stress
	Peptide Sequnce	Ratio 4(2)F	Pvalue 4(2)F		
	LALSSEPYR	47.21	0.0261		
	LVYCPSVCK+Carbamidomethyl(4)Carbamidomethyl(8)	0.71	0.5363		
	VSTDLNIYFPIDTSCPLTK+Carbamidomethyl(15)	13.90	0.0742		
Glyma12g23150.1	Aluminium induced protein with YGL and LRDR motifs	6	26.96	0.0066	hormone metabolism
	Peptide Sequnce	Ratio 4(2)F	Pvalue 4(2)F		
	DLDGSFGFVVYDSK	35.06	0.1606		
	DRGPYPADQVVK	1.13	0.8622		
	GTNEAMFVIEAYK	49.44	0.0133		
	IDSEGAICGANFK+Carbamidomethyl(8)	6.24	0.0556		
	VGSQANWMEWTQH	173.25	0.0287		
	VGSVFAALGSDGGIK	4.30	0.3725		
Glyma19g30210.4	translocon at the outer envelope membrane of chloroplasts 34	4	31.53	0.0258	protein
	Peptide Sequnce	Ratio 4(2)F	Pvalue 4(2)F		
	EWSGINTFAPATQTK	0.34	0.1139		
	LLELLGNLK	0.33	0.0748		
	NLIEGPNPNQR	83.68	0.0681		
	TIDVLLYVDR	0.45	0.2802		
Glyma11g08260.1	Rhodanese/Cell cycle control phosphatase superfamily protein	3	42.21	0.0001	protein
	Peptide Sequnce	Ratio 4(2)F	Pvalue 4(2)F		
	DMGGGYVDWVK	38.48	0.0003		
	SLIQTGSIYLDVR	28.08	0.1322		
	SLYATADLLSDGFK	69.19	0.0208		
Glyma11g20940.2	dehydration induced protein (ERD15)	3	49.39	0.0437	stress
	Peptide Sequnce	Ratio 4(2)F	Pvalue 4(2)F		
	AFLINLTPK	64.46	0.0827		
	QVEDFSPQWWDLVK	52.27	0.0788		
	SSALNPNAPMFIPAALR	11.51	0.2358		
Glyma09g29330.1	Kunitz family trypsin and protease inhibitor protein	6	121.7	0.0037	stress
	Peptide Sequnce	Ratio 4(2)F	Pvalue 4(2)F		
	DVGVFVDENGYR	4.09	0.0547		
	IVYCPSVCPSSK+Carbamidomethyl(4)Carbamidomethyl(8)	66.20	0.0274		
	LALSDVPFK	471.73	0.0473		

	TSYSCAEYSPVWK+Carbamidomethyl(5)	44.72	0.1652		
	VSTDLNIMFCTDR+Carbamidomethyl(10)	117.24	0.1813		
	WFVTTGGSMGNPSWETIR	287.56	0.2176		
Glyma09g27700.1	Concanavalin A like lectin protein kinase family protein	5	461.87	0.0001	signalling
	Peptide Sequnce	Ratio 4(2)F	Pvalue 4(2)F		
	LDDSGKPEFGSVGR	444.25	0.0002		
	NVLQLTK	20.15	0.1420		
	SSQLVSTFETTFTFK	64.08	0.0614		
	TVTAQISYNSASK	590.86	0.2145		
	VLYFAPVHLWK	901.51	0.0237		

Protein ID, according to the Phytozome database; M.P., number of matched peptide; Ratio, relative abundance of protein; Function, functional classification by MapMan bin code; *N.D., Not Description in Phytozome database.

Supplemental Table 4. List of identified root proteins that were differentially changed in soybeans exposed to 2-day flooding stress with calcium compared to 2-day-old untreated soybeans.

Protein ID	Description	M.P.	Ratio 4(2)F+Ca/2(0)	Pvalue 4(2)F+Ca/2(0)	Function
Glyma06g03050.1	Papain family cysteine protease	2	0.05	0.0014	protein
	Peptide Sequnce	Ratio 4(2)F+Ca	Pvalue 4(2)F+Ca		
	APILPTNDLPTDFDWR	0.05	0.0031		
	NSWGESWGEEGYYK	0.04	0.0474		
Glyma05g20930.1	Granulin repeat cysteine protease family protein	2	0.10	0.0044	protein
	Peptide Sequnce	Ratio 4(2)F+Ca	Pvalue 4(2)F+Ca		
	FVSLSEQELVDCDR+Carbamidomethyl(12)	0.11	0.0100		
	NSWGTGWGEDGYFK	0.07	0.0515		
Glyma16g16290.1	Granulin repeat cysteine protease family protein	2	0.10	0.0044	protein
	Peptide Sequnce	Ratio 4(2)F+Ca	Pvalue 4(2)F+Ca		
	FVSLSEQELVDCDR+Carbamidomethyl(12)	0.11	0.0100		
	NSWGTGWGEDGYFK	0.07	0.0515		
Glyma17g18440.1	Granulin repeat cysteine protease family protein	2	0.10	0.0044	protein
	Peptide Sequnce	Ratio 4(2)F+Ca	Pvalue 4(2)F+Ca		
	FVSLSEQELVDCDR+Carbamidomethyl(12)	0.11	0.0100		
	NSWGTGWGEDGYFK	0.07	0.0515		
Glyma02g47330.1	seed imbibition 1	3	0.12	0.0011	minor CHO metabolism
	Peptide Sequnce	Ratio 4(2)F+Ca	Pvalue 4(2)F+Ca		
	DCLFTDPAR+Carbamidomethyl(2)	0.17	0.0521		
	LALPDGSILR	0.06	0.0548		
	VDVQNILETLGAGHGGR	0.21	0.0052		
Glyma08g46090.2	rotamase FKBP 1	2	0.12	0.0003	protein
	Peptide Sequnce	Ratio 4(2)F+Ca	Pvalue 4(2)F+Ca		
	EGEGYERPNEGAIVK	0.11	0.0002		
	VLDLESTNVK	0.19	0.1322		
Glyma18g32830.1	rotamase FKBP 1	2	0.12	0.0003	protein
	Peptide Sequnce	Ratio 4(2)F+Ca	Pvalue 4(2)F+Ca		
	EGEGYERPNEGAIVK	0.11	0.0002		
	VLDLESTNVK	0.19	0.1322		
Glyma06g02290.1	Pyridoxal dependent decarboxylase family protein	11	0.13	0.0018	amino acid metabolism
	Peptide Sequnce	Ratio 4(2)F+Ca	Pvalue 4(2)F+Ca		
	AEIESVLSLGVSPDR	0.00	1.0000		
	IIYANPCK+Carbamidomethyl(7)	0.41	0.3637		

	LPTVQPFYAVK	0.13	0.1061		
	NCHPTCELLLR+Carbamidomethyl(2)Carbamidomethyl(6)	0.01	0.0111		
	NVFEMASGLGLPR	0.15	0.0113		
	PSLVAEAFEAK	0.17	0.1022		
	STFLAYSSPEHSMF	0.00	1.0000		
	TYPSTVFGPTCDSIDTVLR+Carbamidomethyl(11)	0.44	0.8024		
	VLDIGGGFTSGPPFEAAALK	0.02	0.0250		
	VTGVSFHIGSGGADTR	0.37	0.1351		
	YFAETAFTLATR	0.06	0.0657		
Glyma09g15620.2	Cellulose synthase family protein	4	0.13	0.0165	cell wall
	Peptide Sequnce	Ratio 4(2)F+Ca	Pvalue 4(2)F+Ca		
	APEWYFAQK	0.35	0.1715		
	GLDGIQGPVYVGTGCVFNR+Carbamidomethyl(15)	0.15	0.3395		
	NVCYVQFPQR+Carbamidomethyl(3)	0.11	0.0757		
	VGDPGLGNVAWK	0.09	0.0196		
Glyma15g43040.1	Cellulose synthase family protein	4	0.13	0.0165	cell wall
	Peptide Sequnce	Ratio 4(2)F+Ca	Pvalue 4(2)F+Ca		
	APEWYFAQK	0.35	0.1715		
	GLDGIQGPVYVGTGCVFNR+Carbamidomethyl(15)	0.15	0.3395		
	NVCYVQFPQR+Carbamidomethyl(3)	0.11	0.0757		
	VGDPGLGNVAWK	0.09	0.0196		
Glyma09g08120.1	Subtilase family protein	2	0.13	0.0001	protein
	Peptide Sequnce	Ratio 4(2)F+Ca	Pvalue 4(2)F+Ca		
	ALSPGLVYDATPSDYIK	0.10	0.0006		
	DFPAYASLGNK	0.21	0.0199		
Glyma17g05340.2	histone deacetylase 2C	3	0.14	0.0001	RNA
	Peptide Sequnce	Ratio 4(2)F+Ca	Pvalue 4(2)F+Ca		
	FVLGTLSR	0.176098835	0.0070		
	IPQISLELVLEK	0.14	0.0538		
	VDPAEFEACIHLSQAALGEAK+Carbamidomethyl(9)	0.14	0.0014		
Glyma11g17930.1	DNAJ homologue 2	3	0.15	0.0087	stress
	Peptide Sequnce	Ratio 4(2)F+Ca	Pvalue 4(2)F+Ca		
	EIYDQYGEDALK	0.20	0.0940		
	VSLEDLYLGTSK	0.10	0.0251		
	YYEILGVSK	0.05	0.0793		
Glyma12g10150.1	DNAJ homologue 2	3	0.15	0.0087	stress

	Peptide Sequnce	Ratio 4(2)F+Ca	Pvalue 4(2)F+Ca		
	EIYDQYGEDALK	0.20	0.0940		
	VSLEDLYLGTSK	0.10	0.0251		
	YYEILGVSK	0.05	0.0793		
Glyma09g37850.1	Hyaluronan / mRNA binding family	4	0.15	0.0003	RNA
	Peptide Sequnce	Ratio 4(2)F+Ca	Pvalue 4(2)F+Ca		
	EFESMQALSSK	0.20	0.0103		
	EMTLEEYEK	0.18	0.0126		
	GNWGAQTDELAQVTDEVANETEK	0.00	1.0000		
	NLGDEKPAVEEDVADGNK	0.16	0.0030		
Glyma14g01430.1	seed imbibition 1	4	0.16	0.0002	minor CHO metabolism
	Peptide Sequnce	Ratio 4(2)F+Ca	Pvalue 4(2)F+Ca		
	DCLFTDPAR+Carbamidomethyl(2)	0.17	0.0521		
	LALPDGSILR	0.06	0.0548		
	VDVQNILETLGAGHGGR	0.21	0.0052		
	VEDPALGLR	0.28	0.0152		
Glyma12g30600.1	histone deacetylase 2C	3	0.17	0.0000	RNA
	Peptide Sequnce	Ratio 4(2)F+Ca	Pvalue 4(2)F+Ca		
	FVLGTLSR	0.18	0.0070		
	SNEPVVLYLK	0.29	0.0033		
	VDPAEFEACIHLSQAALGEAK+Carbamidomethyl(9)	0.14	0.0014		
Glyma02g46450.3	microtubule associated proteins 651	2	0.17	0.0009	cell
	Peptide Sequnce	Ratio 4(2)F+Ca	Pvalue 4(2)F+Ca		
	IPALVDTLVAK	0.15	0.0116		
	LSAPVNYVAISK	0.18	0.0075		
Glyma14g02180.1	microtubule associated proteins 651	2	0.17	0.0009	cell
	Peptide Sequnce	Ratio 4(2)F+Ca	Pvalue 4(2)F+Ca		
	IPALVDTLVAK	0.15	0.0116		
	LSAPVNYVAISK	0.18	0.0075		
Glyma14g02200.2	microtubule associated proteins 651	2	0.17	0.0009	cell
	Peptide Sequnce	Ratio 4(2)F+Ca	Pvalue 4(2)F+Ca		
	IPALVDTLVAK	0.15	0.0116		
	LSAPVNYVAISK	0.18	0.0075		
Glyma18g12660.1	rhamnose biosynthesis 1	10	0.17	0.0001	cell wall
	Peptide Sequnce	Ratio 4(2)F+Ca	Pvalue 4(2)F+Ca		

	AYSTDAFYFR	0.06	0.0097		
	EYENVCTLR+Carbamidomethyl(6)	0.17	0.0857		
	FLLLAMK	0.00	1.0000		
	FVENRPFNDQR	0.22	0.0164		
	GNNVYGPNQFPEK	0.66	0.5522		
	NPDWWGDVSGALLPHPR	0.01	0.0071		
	SQILFDFR	0.16	0.0167		
	SYGLPVITTR	0.24	0.0637		
	TMDWYVK	0.05	0.1774		
	YYDGSDNVTGTASNGDVNHSNQNR	0.25	0.3441		
Glyma07g00620.1	O fucosyltransferase family protein	7	0.17	0.0000	not assigned
	Peptide Sequnce	Ratio 4(2)F+Ca	Pvalue 4(2)F+Ca		
	NFEDIYDVDVFMK	0.00	1.0000		
	SCFNAQEVAVFLR+Carbamidomethyl(2)	0.24	0.0001		
	SLGATLVIPDIR	0.14	0.0145		
	SQILVPANIPDSSASASSFLSHYVSK	0.16	0.0422		
	VTEDYIAQHVEPIYR	0.11	0.0639		
	WDESLDSLK	0.06	0.0396		
	YLDSEDSELEK	0.09	0.0421		
Glyma04g03020.1	Papain family cysteine protease	2	0.17	0.0131	protein
	Peptide Sequnce	Ratio 4(2)F+Ca	Pvalue 4(2)F+Ca		
	APILPTSDLPTDFDWR	0.19	0.0378		
	NSWGESWGEEGYYK	0.04	0.0474		
Glyma18g48620.1	Hyaluronan / mRNA binding family	6	0.18	0.0000	RNA
	Peptide Sequnce	Ratio 4(2)F+Ca	Pvalue 4(2)F+Ca		
	APYNQGPFEEGDAGK	0.21	0.0178		
	EFESMQALSSK	0.20	0.0103		
	EMTLEEYEK	0.18	0.0126		
	GSSTNNAPAPSIEDPGHFPNLGAK	0.08	0.1449		
	KAPAQQNKPAQLPTKPPPPAQAVR	0.09	0.1028		
	NLGDEKPAVEEDVADGNK	0.16	0.0030		
Glyma11g15040.3	RNA binding (RRM/RBD/RNP motifs) family protein	3	0.18	0.0000	RNA
	Peptide Sequnce	Ratio 4(2)F+Ca	Pvalue 4(2)F+Ca		
	IEIVGTNISTPGVAPAR	0.04	0.0009		

	LYISNLDYGVSSDDIK	0.15	0.0023		
	VSADDLDADLEK	0.27	0.0400		
Glyma08g45425.1	eukaryotic translation initiation factor 4G	2	0.18	0.0097	protein
	Peptide Sequnce	Ratio 4(2)F+Ca	Pvalue 4(2)F+Ca		
	DTSVADQSNTGETYTGTR	0.04	0.1269		
	VNPTPVNSTESNSTYAAR	0.22	0.0100		
Glyma05g37150.1	vacuolar H+ ATPase subunit E isoform 3	2	0.19	0.0422	transport
	Peptide Sequnce	Ratio 4(2)F+Ca	Pvalue 4(2)F+Ca		
	DLIVQCLLR+Carbamidomethyl(6)	0.15	0.0342		
	IVCENTLDAR+Carbamidomethyl(3)	0.34	0.2072		
Glyma05g37160.1	vacuolar H+ ATPase subunit E isoform 3	2	0.19	0.0422	transport
	Peptide Sequnce	Ratio 4(2)F+Ca	Pvalue 4(2)F+Ca		
	DLIVQCLLR+Carbamidomethyl(6)	0.15	0.0342		
	IVCENTLDAR+Carbamidomethyl(3)	0.34	0.2072		
Glyma05g37190.1	vacuolar H+ ATPase subunit E isoform 3	2	0.19	0.0422	transport
	Peptide Sequnce	Ratio 4(2)F+Ca	Pvalue 4(2)F+Ca		
	DLIVQCLLR+Carbamidomethyl(6)	0.15	0.0342		
	IVCENTLDAR+Carbamidomethyl(3)	0.34	0.2072		
Glyma08g02390.1	vacuolar ATP synthase subunit E1	2	0.19	0.0422	transport
	Peptide Sequnce	Ratio 4(2)F+Ca	Pvalue 4(2)F+Ca		
	DLIVQCLLR+Carbamidomethyl(6)	0.15	0.0342		
	IVCENTLDAR+Carbamidomethyl(3)	0.34	0.2072		
Glyma19g27036.1	heat shock protein 81-2	3	0.19	0.0079	stress
	Peptide Sequnce	Ratio 4(2)F+Ca	Pvalue 4(2)F+Ca		
	EGQNDIYYITGESK	0.17	0.0198		
	GIVDSEDLPLNISR	0.21	0.0774		
	HFSVEGQLEFK	0.20	0.1085		
Glyma04g05460.1	N.D. *	2	0.20	0.0088	not assigned
	Peptide Sequnce	Ratio 4(2)F+Ca	Pvalue 4(2)F+Ca		
	AVLSGSQDLDTDSLTSLR	0.20	0.0296		
	GFPVEIQMDTK	0.18	0.0381		
Glyma06g05490.1	N.D. *	2	0.20	0.0088	not assigned
	Peptide Sequnce	Ratio 4(2)F+Ca	Pvalue 4(2)F+Ca		
	AVLSGSQDLDTDSLTSLR	0.20	0.0296		
	GFPVEIQMDTK	0.18	0.0381		

Glyma04g01130.1	cold regulated 47	3	0.20	0.0139	stress
	Peptide Sequnce	Ratio 4(2)F+Ca	Pvalue 4(2)F+Ca		
	GVFDFLGK	0.09	0.0092		
	VEVVETAHAEEK	0.28	0.0453		
	YESSEVEVQDR	0.83	0.8137		
Glyma04g02230.1	Pyridoxal-dependent decarboxylase family protein	10	0.20	0.0361	amino acid metabolism
	Peptide Sequnce	Ratio 4(2)F+Ca	Pvalue 4(2)F+Ca		
	AEIESVLSLGVSPDR	0.00	1.0000		
	IIYANPCK+Carbamidomethyl(7)	0.41	0.3637		
	INAAIEGSFGK	0.12	0.0385		
	LPTVQPFYAVK	0.13	0.1061		
	MGVLDIGGGFTSGPSFEAAALK	0.00	1.0000		
	NGLTDFIQR	0.13	0.0074		
	SIFLACSSPEHTMF+Carbamidomethyl(6)	0.00	1.0000		
	TYPSTVFGPTCDSIDTVLR+Carbamidomethyl(11)	0.44	0.8024		
	VTGVSFHIGSGGADTR	0.37	0.1351		
	YFAETAFTLATR	0.06	0.0657		
Glyma19g09960.1	O fucosyltransferase family protein	3	0.22	0.0000	not assigned
	Peptide Sequnce	Ratio 4(2)F+Ca	Pvalue 4(2)F+Ca		
	SCFNAQEVAVFLR+Carbamidomethyl(2)	0.24	0.0001		
	VTEDYIAQHVEPIYR	0.11	0.0639		
	WDESLDSLK	0.06	0.0396		
Glyma02g01170.1	S adenosylmethionine synthetase 2	4	0.22	0.0000	amino acid metabolism
	Peptide Sequnce	Ratio 4(2)F+Ca	Pvalue 4(2)F+Ca		
	EILQLVK	0.12	0.0367		
	FVIGGPHGDAGLTGR	0.29	0.0000		
	TIFHLNPSGR	0.28	0.0101		
	VLVNIEQQSPDIAQGVHGHFTK	0.14	0.0000		
Glyma03g25621.1	RNA binding (RRM/RBD/RNP motifs) family protein	2	0.22	0.0223	RNA
	Peptide Sequnce	Ratio 4(2)F+Ca	Pvalue 4(2)F+Ca		
	GFAFLEFPSR	0.14	0.0123		
	VSFADSFIDPGDEIMAQVK	0.37	0.2699		
Glyma07g13211.1	RNA binding (RRM/RBD/RNP motifs) family protein	2	0.22	0.0223	RNA
	Peptide Sequnce	Ratio 4(2)F+Ca	Pvalue 4(2)F+Ca		
	GFAFLEFPSR	0.14	0.0123		
	VSFADSFIDPGDEIMAQVK	0.37	0.2699		

Glyma19g21200.1	ATPase AAA type CDC48 protein	3	0.23	0.0100	cell
	Peptide Sequnce	Ratio 4(2)F+Ca	Pvalue 4(2)F+Ca		
	AIANECQANFISVK+Carbamidomethyl(6)	0.30	0.0897		
	EIDIGVPDEVGR	0.22	0.0704		
	ETVVEVPNVSWEDIGGLENVK	0.20	0.0354		
Glyma17g09120.1	tRNA synthetase beta subunit family protein	3	0.23	0.0111	protein
	Peptide Sequnce	Ratio 4(2)F+Ca	Pvalue 4(2)F+Ca		
	FLHIIEDSPVFPVIYDSK	0.21	0.0364		
	FVIEPVEVISSDGK	0.43	0.3003		
	NVFIECTATDLTK+Carbamidomethyl(6)	0.17	0.0235		
Glyma04g07220.1	cellulose synthase 1	3	0.23	0.0302	cell wall
	Peptide Sequnce	Ratio 4(2)F+Ca	Pvalue 4(2)F+Ca		
	APEFYFAQK	0.46	0.1529		
	DLNSYGLGNVDWK	0.13	0.0391		
	NIVFFDINMK	0.17	0.1565		
Glyma06g07320.1	cellulose synthase 1	3	0.23	0.0302	cell wall
	Peptide Sequnce	Ratio 4(2)F+Ca	Pvalue 4(2)F+Ca		
	APEFYFAQK	0.46	0.1529		
	DLNSYGLGNVDWK	0.13	0.0391		
	NIVFFDINMK	0.17	0.1565		
Glyma04g02271.1	sterol methyltransferase 2	3	0.24	0.0120	hormone metabolism
	Peptide Sequnce	Ratio 4(2)F+Ca	Pvalue 4(2)F+Ca		
	ANVVGITINEYQVNR	0.30	0.1008		
	LEEVYAEIFR	0.16	0.0716		
	QYWSFFR	0.14	0.0390		
Glyma18g14826.1	ATPase AAAtype CDC48 protein	2	0.24	0.0366	cell
	Peptide Sequnce	Ratio 4(2)F+Ca	Pvalue 4(2)F+Ca		
	DIALAEIEK	1.55956231	0.5946		
	GVLFYGPPGCGK+Carbamidomethyl(10)	0.263216625	0.0989		
Glyma20g35120.5	S adenosyl Lmethionine dependent methyltransferases superfamily protein	3	0.24	0.0192	stress
	Peptide Sequnce	Ratio 4(2)F+Ca	Pvalue 4(2)F+Ca		
	DGILLLELDR	0.29	0.1079		
	GIPAYLGVLGTK	0.25	0.0865		
	MWLTSESFR	0.19	0.0552		

Glyma12g08410.2	ATPase AAA type CDC48 protein	8	0.25	0.0009	cell
	Peptide Sequnce	Ratio 4(2)F+Ca	Pvalue 4(2)F+Ca		
	AIANECQANFISVK+Carbamidomethyl(6)	0.30	0.0897		
	DTHGYVGADLAAICTEAALQCIR+Carbamidomethyl(14)Carbamidomethyl(21)	0.21	0.0604		
	EIDIGVPDEVGR	0.22	0.0704		
	ELQETVQYPVEHPEK	0.33	0.0992		
	ELVELPLR	0.26	0.1564		
	ETVVEVPNVSWEDIGGLENVK	0.20	0.0354		
	GILLYGPPGSGK	0.25	0.1162		
	GVLFYGPPGCGK+Carbamidomethyl(10)	0.26	0.0989		
Glyma07g36150.1	S adenosylmethionine synthetase family protein	12	0.25	0.0000	metal handling
	Peptide Sequnce	Ratio 4(2)F+Ca	Pvalue 4(2)F+Ca		
	EHVIKPVIPEK	0.33	0.1128		
	ENFDFRPGMISINLDLK	0.43	0.4068		
	FVIGGPHGDAGLTGR	0.29	0.0000		
	IIIDTYGGWGAHGGGAFSGK	0.54	0.0079		
	LCDQISDAVLDACLEQDPDSK+Carbamidomethyl(2)Carbamidomethyl(13)	0.15	0.0006		
	NGTCPWLRPDGK+Carbamidomethyl(4)	1.28	0.8291		
	NIGFVSNDVGLDADNCK+Carbamidomethyl(16)	0.17	0.0001		
	TIFHLNPSGR	0.28	0.0101		
	TNLVMVFGEITTK	0.21	0.0899		
	TQVTVEYYNDNGAR	0.20	0.0833		
	VHTVLISTQHDETVTNDEIAADLK	0.19	0.0007		
	VLVNIEQQSPDIAQGVHGHLTK	0.24	0.0000		
Glyma17g04330.1	S adenosylmethionine synthetase family protein	12	0.25	0.0000	metal handling
	Peptide Sequnce	Ratio 4(2)F+Ca	Pvalue 4(2)F+Ca		
	EHVIKPVIPEK	0.33	0.1128		
	ENFDFRPGMISINLDLK	0.43	0.4068		
	FVIGGPHGDAGLTGR	0.29	0.0000		
	IIIDTYGGWGAHGGGAFSGK	0.54	0.0079		
	LCDQISDAVLDACLEQDPDSK+Carbamidomethyl(2)Carbamidomethyl(13)	0.15	0.0006		
	NGTCPWLRPDGK+Carbamidomethyl(4)	1.28	0.8291		
	NIGFVSNDVGLDADNCK+Carbamidomethyl(16)	0.17	0.0001		
	TIFHLNPSGR	0.28	0.0101		
	TNLVMVFGEITTK	0.21	0.0899		

	TQVTVEYYNDNGAR	0.20	0.0833		
	VHTVLISTQHDETVTNDEIAADLK	0.19	0.0007		
	VLVNIEQQSPDIAQGVHGHLTK	0.24	0.0000		
Glyma17g04340.1	S adenosylmethionine synthetase family protein	12	0.25	0.0000	metal handling
	Peptide Sequnce	Ratio 4(2)F+Ca	Pvalue 4(2)F+Ca		
	EHVIKPVIPEK	0.33	0.1128		
	ENFDFRPGMISINLDLK	0.43	0.4068		
	FVIGGPHGDAGLTGR	0.29	0.0000		
	IIIDTYGGWGAHGGGAFSGK	0.54	0.0079		
	LCDQISDAVLDACLEQDPDSK+Carbamidomethyl(2)Carbamidomethyl(13)	0.15	0.0006		
	NGTCPWLRPDGK+Carbamidomethyl(4)	1.28	0.8291		
	NIGFVSNDVGLDADNCK+Carbamidomethyl(16)	0.17	0.0001		
	TIFHLNPSGR	0.28	0.0101		
	TNLVMVFGEITTK	0.21	0.0899		
	TQVTVEYYNDNGAR	0.20	0.0833		
	VHTVLISTQHDETVTNDEIAADLK	0.19	0.0007		
	VLVNIEQQSPDIAQGVHGHLTK	0.24	0.0000		
Glyma12g31620.1	DNAJ homologue 2	5	0.25	0.0392	stress
	Peptide Sequnce	Ratio 4(2)F+Ca	Pvalue 4(2)F+Ca		
	AINDEGMPNYQR	0.32	0.0688		
	EIYDTYGEDALK	0.00	1.0000		
	QQAQQEAYEEDEDMHGGAQR	4.36	0.5243		
	VSLEDLYLGTSK	0.10	0.0251		
	YYEILGVSK	0.05	0.0793		
Glyma10g28500.1	S-adenosylmethionine synthetase 2	7	0.26	0.0000	amino acid metabolism
	Peptide Sequnce	Ratio 4(2)F+Ca	Pvalue 4(2)F+Ca		
	DDPDFTWEVVKPLK	0.48	0.0731		
	EHVIKPVIPEK	0.33	0.1128		
	FVIGGPHGDAGLTGR	0.29	0.0000		
	LCDQISDAVLDACLEQDPESK+Carbamidomethyl(2)Carbamidomethyl(13)	0.12	0.1254		
	RPEDIGAGDQGHMFGYATDETPELMPLSHVLATK+Oxidation(13)	0.25	0.1236		
	TNLVMVFGEITTK	0.21	0.0899		
	VHTVLISTQHDETVTNDEIAADLK	0.19	0.0007		
Glyma08g22380.1	Protein of unknown function DUF642	2	0.26	0.0268	not assigned

	Peptide Sequnce	Ratio 4(2)F+Ca	Pvalue 4(2)F+Ca		
	VEIVIHNPGVDEDPACGPLIDSVALK+Carbamidomethyl(16)	0.43	0.1955		
	YIDSDHFAVPEGK	0.25	0.0211		
Glyma05g31830.1	copper ion binding;cobalt ion binding;zinc ion binding	5	0.26	0.0005	not assigned
	Peptide Sequnce	Ratio 4(2)F+Ca	Pvalue 4(2)F+Ca		
	DALEAMETQK	0.22	0.0555		
	FETAIGILK	0.40	0.0804		
	GMDLLLAEFDK	0.27	0.0488		
	TYLLTLK	0.23	0.0339		
	YEEELELK	0.24	0.0191		
Glyma08g15100.1	copper ion binding;cobalt ion binding;zinc ion binding	5	0.26	0.0005	not assigned
	Peptide Sequnce	Ratio 4(2)F+Ca	Pvalue 4(2)F+Ca		
	DALEAMETQK	0.22	0.0555		
	FETAIGILK	0.40	0.0804		
	GMDLLLAEFDK	0.27	0.0488		
	TYLLTLK	0.23	0.0339		
	YEEELELK	0.24	0.0191		
Glyma14g34990.1	copper ion binding;cobalt ion binding;zinc ion binding	5	0.26	0.0005	not assigned
	Peptide Sequnce	Ratio 4(2)F+Ca	Pvalue 4(2)F+Ca		
	DALEAMETQK	0.22	0.0555		
	FETAIGILK	0.40	0.0804		
	GMDLLLAEFDK	0.27	0.0488		
	TYLLTLK	0.23	0.0339		
	YEEELELK	0.24	0.0191		
Glyma14g38600.1	eukaryotic translation initiation factor 2 beta subunit	5	0.26	0.0060	protein
	Peptide Sequnce	Ratio 4(2)F+Ca	Pvalue 4(2)F+Ca		
	EEVPEIVPFDPTK	0.47	0.1641		
	ITIVDPADEPVEK	0.30	0.1283		
	NFEGILR	0.17	0.0464		
	NYEYEELLGR	0.07	0.0420		
	TVFVNFMDLCK+Carbamidomethyl(10)	0.16	0.1068		
Glyma01g00740.3	Ribosomal protein L31e family protein	2	0.26	0.0017	protein
	Peptide Sequnce	Ratio 4(2)F+Ca	Pvalue 4(2)F+Ca		
	EELYSLVTVVEIPK	0.13	0.0022		
	FVWSQGIR	0.33	0.0810		

Glyma02g40310.1	eukaryotic translation initiation factor 2 beta subunit	5	0.26	0.0001	protein
	Peptide Sequnce	Ratio 4(2)F+Ca	Pvalue 4(2)F+Ca		
	EEVPEIVPFDPTK	0.47	0.1641		
	ITIVDPADDPVEK	0.12	0.0004		
	NFEGILR	0.17	0.0464		
	TENLSVSEGVETAFAGLK	0.27	0.0447		
	TVFVNFMDLCK+Carbamidomethyl(10)	0.16	0.1068		
Glyma1337s00200.1	S-adenosylmethionine synthetase 2	11	0.27	0.0000	amino acid metabolism
	Peptide Sequnce	Ratio 4(2)F+Ca	Pvalue 4(2)F+Ca		
	EHVIKPVIPEK	0.33	0.1128		
	ENFDFRPGMISINLDLK	0.43	0.4068		
	FVIGGPHGDAGLTGR	0.29	0.0000		
	IIIDTYGGWGAHGGGAFSGK	0.54	0.0079		
	LCDQISDAVLDACLEQDPDSK+Carbamidomethyl(2)Carbamidomethyl(13)	0.15	0.0006		
	NGTCPWLRPDGK+Carbamidomethyl(4)	1.28	0.8291		
	TIFHLNPSGR	0.28	0.0101		
	TNLVMVFGEITTK	0.21	0.0899		
	TQVTVEYYNDNGAR	0.20	0.0833		
	VHTVLISTQHDETVTNDEIAADLK	0.19	0.0007		
	VLVNIEQQSPDIAQGVHGHLTK	0.24	0.0000		
Glyma03g37240.4	RNA binding (RRM/RBD/RNP motifs) family protein	2	0.27	0.0185	RNA
	Peptide Sequnce	Ratio 4(2)F+Ca	Pvalue 4(2)F+Ca		
	AGDVCFSQVFHDGR+Carbamidomethyl(5)	0.28	0.0207		
	GTTGIVDYTNYDDMK	0.25	0.1293		
Glyma12g10420.1	SKU5 similar 5	6	0.28	0.0000	not assigned
	Peptide Sequnce	Ratio 4(2)F+Ca	Pvalue 4(2)F+Ca		
	DEYPIPSNAIR	0.32	0.0136		
	DQIGSYFYYPSLAFHK	0.29	0.0014		
	GSNAYTFTVDQGK	0.33	0.0030		
	SENWVHQYLGQQFYLR	0.26	0.0916		
	VYSPANSWR	0.25	0.0122		
	YAVNSVSFIPADTPLK	0.19	0.0027		
Glyma05g04220.1	stress inducible protein putative	3	0.28	0.0330	stress
	Peptide Sequnce	Ratio 4(2)F+Ca	Pvalue 4(2)F+Ca		
	ALELDDEDISYLTNR	0.29	0.0885		
	ELEQQEYFDPK	0.34	0.1704		

	LGAMPEGLK	0.26	0.0697		
Glyma17g14660.1	stress inducible protein putative	3	0.28	0.0330	stress
	Peptide Sequnce	Ratio 4(2)F+Ca	Pvalue 4(2)F+Ca		
	ALELDDEDISYLTNR	0.29	0.0885		
	ELEQQEYFDPK	0.34	0.1704		
	LGAMPEGLK	0.26	0.0697		
Glyma06g46350.1	SKU5 similar 5	7	0.28	0.0000	not assigned
	Peptide Sequnce	Ratio 4(2)F+Ca	Pvalue 4(2)F+Ca		
	DEYPIPSNAIR	0.32	0.0136		
	DQIGSYFYYPSLAFHK	0.29	0.0014		
	GSNAYTFTVDQGK	0.33	0.0030		
	ISNVGLTTSINFR	0.44	0.1155		
	SENWVHQYLGQQFYLR	0.26	0.0916		
	VYSPANSWR	0.25	0.0122		
	YAVNSVSFIPADTPLK	0.19	0.0027		
Glyma03g37280.1	NAD(P) binding Rossmann fold superfamily protein	2	0.29	0.0163	cell wall
	Peptide Sequnce	Ratio 4(2)F+Ca	Pvalue 4(2)F+Ca		
	ETYWGNVNPIGVR	0.24	0.1906		
	VVSNFVAQALR	0.29	0.0121		
Glyma10g06480.1	ATPase AAA type CDC48 protein	20	0.29	0.0000	cell
	Peptide Sequnce	Ratio 4(2)F+Ca	Pvalue 4(2)F+Ca		
	AIANECQANFISVK+Carbamidomethyl(6)	0.30	0.0897		
	DFSTAILER	0.32	0.1343		
	DTHGYVGADLAALCTEAALQCIR+Carbamidomethyl(14)Carbamidomethyl(21)	0.21	0.0604		
	DTICIALADETCEEPK+Carbamidomethyl(4)Carbamidomethyl(12)	0.25	0.1309		
	EDENRLDEVGYDDVGGVR	0.31	0.0891		
	EIDIGVPDEVGR	0.22	0.0704		
	ELQETVQYPVEHPEK	0.33	0.0992		
	ELVELPLR	0.26	0.1564		
	ETVVEVPNVSWEDIGGLENVK	0.20	0.0354		
	GILLYGPPGSGK	0.25	0.1162		
	GVLFYGPPGCGK+Carbamidomethyl(10)	0.26	0.0989		
	LAEDVDLER	0.13	0.0990		
	LAGESESNLR	1.96	0.5215		
	LDEVGYDDVGGVR	0.36	0.0318		
	LDQLIYIPLPDEDSR	0.29	0.0746		

	LVVDEAVNDDNSVVALHPNTMEK	0.00	1.0000		
	TVFIIGATNRPDIIDPALLRPGR	0.31	0.1523		
	VLNQLLTEMDGMSAK	0.29	0.2509		
	YQAFAQTLQQSR	0.30	0.1399		
	YTQGFSGADITEICQR+Carbamidomethyl(14)	0.25	0.0675		
Glyma10g32471.1	S adenosyl L methionine dependent methyltransferases superfamily protein	4	0.30	0.0154	stress
	Peptide Sequnce	Ratio 4(2)F+Ca	Pvalue 4(2)F+Ca		
	DGLLLLELDR	0.29	0.1079		
	GIPAYLGVLGTK	0.25	0.0865		
	LADFGYSNDMFEK	0.32	0.1525		
	MWLTSESFR	0.19	0.0552		
Glyma16g04950.1	xyloglucan endotransglucosylase/hydrolase 5	10	0.30	0.0000	cell wall
	Peptide Sequnce	Ratio 4(2)F+Ca	Pvalue 4(2)F+Ca		
	APFIAAYK	0.29	0.0669		
	DLDAAQWR	0.10	0.0198		
	FPFDQPMK	0.10	0.1217		
	GFHIDGCEASVNAK+Carbamidomethyl(7)	0.06	0.0013		
	IYLWFDPTK	0.07	0.0279		
	IYNSLWNADDWATR	0.07	0.0069		
	NYVPTWAFDHIK	0.74	0.0002		
	TGQPYILQTNVFTGGK	0.50	0.0038		
	WWDQPEFR	0.04	0.0669		
	YTIYNYCTDTK+Carbamidomethyl(7)	0.10	0.0005		
Glyma05g07640.3	tRNA synthetase beta subunit family protein	4	0.30	0.0109	protein
	Peptide Sequnce	Ratio 4(2)F+Ca	Pvalue 4(2)F+Ca		
	FLHIIEDSPVFPVIYDSK	0.21	0.0364		
	FVIEPVEVISSDGK	0.43	0.3003		
	NVFIECTATDLTK+Carbamidomethyl(6)	0.17	0.0235		
	YNSFIDLQDK	0.46	0.1915		
Glyma08g23770.1	O fucosyltransferase family protein	5	0.31	0.0000	not assigned
	Peptide Sequnce	Ratio 4(2)F+Ca	Pvalue 4(2)F+Ca		
	SCFNAQEVAVFLR+Carbamidomethyl(2)	0.24	0.0001		
	SLGATLVIPDIR	0.14	0.0145		
	SQILVPANIPDSSASASSFLSHYVSK	0.16	0.0422		
	VTEEYIAQHVEPIYR	0.78	0.4133		
	WDESLDSLK	0.06	0.0396		

Glyma09g16553.1	Ribosomal L22e protein family	2	0.31	0.0126	protein
	Peptide Sequnce	Ratio 4(2)F+Ca	Pvalue 4(2)F+Ca		
	AGALGDSITVTR	0.32	0.0438		
	IIVTSDSNFSK	0.31	0.0391		
Glyma13g20680.1	ATPase AAA type CDC48 protein	20	0.32	0.0000	cell
	Peptide Sequnce	Ratio 4(2)F+Ca	Pvalue 4(2)F+Ca		
	AIANECQANFISVK+Carbamidomethyl(6)	0.30	0.0897		
	DFSTAILER	0.32	0.1343		
	DTHGYVGADLAALCTEAALQCIR+Carbamidomethyl(14)Carbamidomethyl(21)	0.21	0.0604		
	DTICIALADETCEEPK+Carbamidomethyl(4)Carbamidomethyl(12)	0.25	0.1309		
	EDENRLDEVGYDDVGGVR	0.31	0.0891		
	EIDIGVPDEVGR	0.22	0.0704		
	ELQETVQYPVEHPEK	0.33	0.0992		
	ELVELPLR	0.26	0.1564		
	ETVVEVPNVSWEDIGGLENVK	0.20	0.0354		
	GILLYGPPGSGK	0.25	0.1162		
	GVLFYGPPGCGK+Carbamidomethyl(10)	0.26	0.0989		
	LAEDVDLER	0.13	0.0990		
	LAGESESNLR	1.96	0.5215		
	LDEVGYDDVGGVR	0.36	0.0318		
	LDQLIYIPLPDEDSR	0.29	0.0746		
	LVVDEAVNDDNSVVVLHPDTMEK	0.08	0.0455		
	TVFIIGATNRPDIIDPALLRPGR	0.31	0.1523		
	VLNQLLTEMDGMSAK	0.29	0.2509		
	YQAFAQTLQQSR	0.30	0.1399		
	YTQGFSGADITEICQR+Carbamidomethyl(14)	0.25	0.0675		
Glyma01g29420.1	metallopeptidase M24 family protein	5	0.32	0.0015	protein
	Peptide Sequnce	Ratio 4(2)F+Ca	Pvalue 4(2)F+Ca		
	AWLALGTK	0.19	0.0889		
	ELDLTSPEVVTK	0.27	0.0829		
	FIFSEISQK	0.52	0.3084		
	IVEGVLSHQMK	0.22	0.0042		
	VVLSVSNPDTR	0.21	0.0666		
Glyma19g28220.1	xyloglucan endotransglucosylase/hydrolase 5	9	0.32	0.0000	cell wall

	Peptide Sequnce	Ratio 4(2)F+Ca	Pvalue 4(2)F+Ca		
	APFIAAYK	0.29	0.0669		
	FPFDQPMK	0.10	0.1217		
	GFHIDGCEASVNAK+Carbamidomethyl(7)	0.06	0.0013		
	IYLWFDPTK	0.07	0.0279		
	IYNSLWNADDWATR	0.07	0.0069		
	NYVPTWAFDHIK	0.74	0.0002		
	SWWDQPEFR	0.01	0.0340		
	TGQPYILQTNVFTGGK	0.50	0.0038		
	YTIYNYCTDR+Carbamidomethyl(7)	0.25	0.0573		
Glyma13g05520.4	RNA binding KH domain containing protein	2	0.32	0.0097	RNA
	Peptide Sequnce	Ratio 4(2)F+Ca	Pvalue 4(2)F+Ca		
	LLVPSDQIGCVIGK+Carbamidomethyl(10)	0.33	0.0253		
	LVCPTGNIGGVIGK+Carbamidomethyl(3)	0.30	0.0497		
Glyma19g02840.2	RNA binding KH domain containing protein	2	0.32	0.0097	RNA
	Peptide Sequnce	Ratio 4(2)F+Ca	Pvalue 4(2)F+Ca		
	LLVPSDQIGCVIGK+Carbamidomethyl(10)	0.332596373	0.0253		
	LVCPTGNIGGVIGK+Carbamidomethyl(3)	0.298454283	0.0497		
Glyma05g03140.1	adenylate kinase 1	6	0.32	0.0000	nucleotide metabolism
	Peptide Sequnce	Ratio 4(2)F+Ca	Pvalue 4(2)F+Ca		
	GFILDGFPR	0.36	0.0210		
	GLVANLHAEKPPK	0.97	0.9787		
	LILIGPPGSGK	0.25	0.0109		
	QTEPVIDYYSK	0.54	0.1428		
	VLGVDDVTGEPLIQR	0.25	0.0001		
	VLNFAIDDAILEER	0.33	0.0000		
Glyma17g13760.1	adenylate kinase 1	7	0.33	0.0000	nucleotide metabolism
	Peptide Sequnce	Ratio 4(2)F+Ca	Pvalue 4(2)F+Ca		
	GFILDGFPR	0.36	0.0210		
	GLVANLHAEKPPK	0.97	0.9787		
	LDEMLQNQGVK	0.38	0.0470		
	LILIGPPGSGK	0.25	0.0109		
	QTEPVIDYYSK	0.54	0.1428		
	VLGVDDVTGEPLIQR	0.25	0.0001		
	VLNFAIDDAILEER	0.33	0.0000		

Glyma04g01244.1	thymidylate synthase 1	2	0.33	0.0086	C1-metabolism
	Peptide Sequnce	Ratio 4(2)F+Ca	Pvalue 4(2)F+Ca		
	IILSAWNPSDLK	0.55	0.4181		
	VFVIGGGQIFR	0.23	0.0026		
Glyma10g33350.2	ARABIDOPSIS THALIANA PEROXYGENASE 2	2	0.33	0.0302	development
	Peptide Sequnce	Ratio 4(2)F+Ca	Pvalue 4(2)F+Ca		
	ESLITEAPNAPVTAQR	0.16	0.0278		
	YVPANIENIFSK	0.42	0.1708		
Glyma20g03060.1	Nuclear transport factor 2 (NTF2) family protein	7	0.33	0.0000	protein
	Peptide Sequnce	Ratio 4(2)F+Ca	Pvalue 4(2)F+Ca		
	EVSQPLENGNVSVTEK	0.20	0.0591		
	FTQSFFLAPQDK	0.50	0.0217		
	GYFVLNDVFR	0.41	0.0198		
	ILSLDYTSFR	0.29	0.0038		
	SFASIVNALK	0.34	0.0002		
	VEILSADAQPSFK	0.19	0.0001		
	VSSIPAPEAPAPSIESPPEK	0.09	0.0001		
Glyma20g21431.1	Ribosomal protein L14p/L23e family protein	2	0.33	0.0116	protein
	Peptide Sequnce	Ratio 4(2)F+Ca	Pvalue 4(2)F+Ca		
	ECADLWPR+Carbamidomethyl(2)	0.34	0.0341		
	VLPAVIVR	0.32	0.0454		
Glyma02g02170.1	NAD(P) binding Rossmann fold superfamily protein	3	0.33	0.0291	cell wall
	Peptide Sequnce	Ratio 4(2)F+Ca	Pvalue 4(2)F+Ca		
	ETYWGNVNPIGVR	0.24	0.1906		
	GDSVIVVDNFFTGR	0.61	0.3866		
	VVSNFVAQALR	0.29	0.0121		
Glyma19g39870.1	NAD(P) binding Rossmann fold superfamily protein	3	0.33	0.0291	cell wall
	Peptide Sequnce	Ratio 4(2)F+Ca	Pvalue 4(2)F+Ca		
	ETYWGNVNPIGVR	0.24	0.1906		
	GDSVIVVDNFFTGR	0.61	0.3866		
	VVSNFVAQALR	0.29	0.0121		
Glyma19g36740.1	ATPase AAA type CDC48 protein	21	0.33	0.0000	cell
	Peptide Sequnce	Ratio 4(2)F+Ca	Pvalue 4(2)F+Ca		
	AIANECQANFISVK+Carbamidomethyl(6)	0.30	0.0897		

	DFSTAILER	0.32	0.1343		
	DNPEAMEEDDVEDEIAEIK	0.43	0.2941		
	DTHGYVGADLAALCTEAALQCIR+Carbamidomethyl(14)Carbamidomethyl(21)	0.21	0.0604		
	EDENRLDEVGYDDVGGVR	0.31	0.0891		
	EIDIGVPDEVGR	0.22	0.0704		
	ELQETVQYPVEHPEK	0.33	0.0992		
	ELVELPLR	0.26	0.1564		
	ETVVEVPNVSWEDIGGLENVK	0.20	0.0354		
	GILLYGPPGSGK	0.25	0.1162		
	GVLFYGPPGCGK+Carbamidomethyl(10)	0.26	0.0989		
	LAEDVDLER	0.13	0.0990		
	LAGESESNLR	1.96	0.5215		
	LDEVGYDDVGGVR	0.36	0.0318		
	LDQLIYIPLPDEDSR	0.29	0.0746		
	LGDVVSVHQCPDVK+Carbamidomethyl(10)	0.35	0.1444		
	LVVDEAVNDDNSVVALHPDTMEK	0.30	0.1392		
	TVFIIGATNRPDIIDPALLRPGR	0.31	0.1523		
	VLNQLLTEMDGMSAK	0.29	0.2509		
	YQAFAQTLQQSR	0.30	0.1399		
	YTQGFSGADITEICQR+Carbamidomethyl(14)	0.25	0.0675		
Glyma02g47460.1	ADP glucose pyrophosphorylase 1	3	0.33	0.0087	major CHO metabolism
	Peptide Sequnce	Ratio 4(2)F+Ca	Pvalue 4(2)F+Ca		
	LIDIPVSNCLNSNVSK+Carbamidomethyl(9)	0.03	0.0102		
	NVMLDLLR	0.23	0.1986		
	VDTTILGLDDER	0.37	0.0928		
Glyma14g01290.1	ADP glucose pyrophosphorylase 1	3	0.33	0.0087	major CHO metabolism
	Peptide Sequnce	Ratio 4(2)F+Ca	Pvalue 4(2)F+Ca		
	LIDIPVSNCLNSNVSK+Carbamidomethyl(9)	0.03	0.0102		
	NVMLDLLR	0.23	0.1986		
	VDTTILGLDDER	0.37	0.0928		
Glyma08g46520.1	cytochrome P450 family 93 subfamily D polypeptide 1	2	0.33	0.0334	misc
	Peptide Sequnce	Ratio 4(2)F+Ca	Pvalue 4(2)F+Ca		
	GQYYQLLPFGSGR	0.25	0.0091		
	LPPGPPISIPLLGHAPYLR	1.65	0.5910		

Glyma03g33990.1	ATPase AAA type CDC48 protein	21	0.33	0.0000	cell
	Peptide Sequnce	Ratio 4(2)F+Ca	Pvalue 4(2)F+Ca		
	AIANECQANFISVK+Carbamidomethyl(6)	0.30	0.0897		
	DFSTAILER	0.32	0.1343		
	DNPEAMEEDDVEDEIAEIK	0.43	0.2941		
	DTHGYVGADLAALCTEAALQCIR+Carbamidomethyl(14)Carbamidomethyl(21)	0.21	0.0604		
	DTICIALADETCEEPK+Carbamidomethyl(4)Carbamidomethyl(12)	0.25	0.1309		
	EDENRLDEVGYDDVGGVR	0.31	0.0891		
	EIDIGVPDEVGR	0.22	0.0704		
	ELQETVQYPVEHPEK	0.33	0.0992		
	ELVELPLR	0.26	0.1564		
	ETVVEVPNVSWEDIGGLENVK	0.20	0.0354		
	GILLYGPPGSGK	0.25	0.1162		
	GVLFYGPPGCGK+Carbamidomethyl(10)	0.26	0.0989		
	LAGESESNLR	1.96	0.5215		
	LDEVGYDDVGGVR	0.36	0.0318		
	LDQLIYIPLPDEDSR	0.29	0.0746		
	LGDVVSVHQCPDVK+Carbamidomethyl(10)	0.35	0.1444		
	LVVDEAVNDDNSVVALHPDTMEK	0.30	0.1392		
	TVFIIGATNRPDIIDPALLRPGR	0.31	0.1523		
	VLNQLLTEMDGMSAK	0.29	0.2509		
	YQAFAQTLQQSR	0.30	0.1399		
	YTQGFSGADITEICQR+Carbamidomethyl(14)	0.25	0.0675		
Glyma08g11650.1	Chalcone and stilbene synthase family protein	4	0.33	0.0004	secondary metabolism
	Peptide Sequnce	Ratio 4(2)F+Ca	Pvalue 4(2)F+Ca		
	ITHLIFCTTSGVDMPGADYQLTK+Carbamidomethyl(7)	0.26	0.0638		
	VLVVCSEITAVTFR+Carbamidomethyl(5)	0.40	0.0110		
	YMMYQQGCFAGGTVLR+Carbamidomethyl(8)	0.14	0.0091		
	YMYLNEEILK	0.48	0.1203		
Glyma17g03030.1	NAD(P) binding Rossmann fold superfamily protein	2	0.34	0.0297	cell wall
	Peptide Sequnce	Ratio 4(2)F+Ca	Pvalue 4(2)F+Ca		
	GDSVIVVDNFFTGR	0.61	0.3866		
	VVSNFVAQALR	0.29	0.0121		
Glyma14g11760.1	early nodulin like protein 15	2	0.34	0.0012	misc
	Peptide Sequnce	Ratio 4(2)F+Ca	Pvalue 4(2)F+Ca		
	FQVGDYLVWK	0.39	0.0249		

	LVVVVLTPR	0.18	0.0050		
Glyma17g34040.1	early nodulin like protein 15	2	0.34	0.0012	misc
	Peptide Sequnce	Ratio 4(2)F+Ca	Pvalue 4(2)F+Ca		
	FQVGDYLVWK	0.39	0.0249		
	LVVVVLTPR	0.18	0.0050		
Glyma16g26630.1	xyloglucan endotransglucosylase/hydrolase 5	4	0.34	0.0000	cell wall
	Peptide Sequnce	Ratio 4(2)F+Ca	Pvalue 4(2)F+Ca		
	GFHIDGCEASVNAK+Carbamidomethyl(7)	0.06	0.0013		
	IYNSLWNADDWATR	0.07	0.0069		
	TGQPYILQTNVFTGGK	0.50	0.0038		
	WWDQPEFR	0.04	0.0669		
Glyma07g40090.1	glycosyl hydrolase 9A1	3	0.34	0.0024	cell wall
	Peptide Sequnce	Ratio 4(2)F+Ca	Pvalue 4(2)F+Ca		
	ALLFFNAQK	0.40	0.1435		
	DLVGGYYDAGDAIK	0.08	0.0094		
	WGTDYLLK	0.45	0.0288		
Glyma19g36580.1	Pyridoxal-dependent decarboxylase family protein	5	0.34	0.0009	amino acid metabolism
	Peptide Sequnce	Ratio 4(2)F+Ca	Pvalue 4(2)F+Ca		
	DLIDTVR	0.77	0.5937		
	HGETFEDHIR	0.06	0.0592		
	INPDVDPQVHPYVATGNK	0.51	0.0636		
	SEDGYLYCEGLK+Carbamidomethyl(8)	0.34	0.0173		
	SLIANTCCLVNR+Carbamidomethyl(7)Carbamidomethyl(8)	0.25	0.0080		
Glyma18g52860.1	O Glycosyl hydrolases family 17 protein	4	0.34	0.0065	misc
	Peptide Sequnce	Ratio 4(2)F+Ca	Pvalue 4(2)F+Ca		
	AFAGSGISVTVTAPNGDIAALTK	0.34	0.3254		
	IYDVNPDILR	0.32	0.0673		
	TLHSALLAEGITDIK	0.35	0.0017		
	TPLMVNPYPYFGYNGK	0.65	0.6612		
Glyma13g39830.1	ATPase AAA type CDC48 protein	16	0.34	0.0001	cell
	Peptide Sequnce	Ratio 4(2)F+Ca	Pvalue 4(2)F+Ca		
	AIANECQANFISVK+Carbamidomethyl(6)	0.30	0.0897		
	DFSTAILER	0.32	0.1343		
	DTHGYVGADLAALCTEAALQCIR+Carbamidomethyl(14)Carbamidomethyl(21)	0.21	0.0604		
	EIDIGVPDEVGR	0.22	0.0704		

	ELQETVQYPVEHPEK	0.33	0.0992		
	ELVELPLR	0.26	0.1564		
	ETVVEVPNVSWEDIGGLENVK	0.20	0.0354		
	GILLYGPPGSGK	0.25	0.1162		
	GVLFYGPPGCGK+Carbamidomethyl(10)	0.26	0.0989		
	LAGESESNLR	1.96	0.5215		
	LDEVGYDDVGGVR	0.36	0.0318		
	LDQLIYIPLPDEDSR	0.29	0.0746		
	LGDVVSVHQCPDVK+Carbamidomethyl(10)	0.35	0.1444		
	TVFIIGATNRPDIIDPALLRPGR	0.31	0.1523		
	VLNQLLTEMDGMSAK	0.29	0.2509		
	YQAFAQTLQQSR	0.30	0.1399		
Glyma09g23330.1	UDP glucosyl transferase 88A1	2	0.35	0.0468	misc
	Peptide Sequnce	Ratio 4(2)F+Ca	Pvalue 4(2)F+Ca		
	FLWVVR	0.24	0.0833		
	ILNSISQTSNLK	0.36	0.0965		
Glyma09g23600.1	UDP glucosyl transferase 88A1	2	0.35	0.0468	misc
	Peptide Sequnce	Ratio 4(2)F+Ca	Pvalue 4(2)F+Ca		
	FLWVVR	0.24	0.0833		
	ILNSISQTSNLK	0.36	0.0965		
Glyma16g29340.1	UDP glucosyl transferase 88A1	2	0.35	0.0468	misc
	Peptide Sequnce	Ratio 4(2)F+Ca	Pvalue 4(2)F+Ca		
	FLWVVR	0.24	0.0833		
	ILNSISQTSNLK	0.36	0.0965		
Glyma16g29370.1	UDP glucosyl transferase 88A1	2	0.35	0.0468	misc
	Peptide Sequnce	Ratio 4(2)F+Ca	Pvalue 4(2)F+Ca		
	FLWVVR	0.24	0.0833		
	ILNSISQTSNLK	0.36	0.0965		
Glyma03g33830.1	Pyridoxal-dependent decarboxylase family protein	6	0.35	0.0009	amino acid metabolism
	Peptide Sequnce	Ratio 4(2)F+Ca	Pvalue 4(2)F+Ca		
	DLIDTVR	0.77	0.5937		
	HGETFEDHIR	0.06	0.0592		
	INPDVDPQVHPYVATGNK	0.51	0.0636		
	LQWFLDAVK	0.48	0.1861		

	SEDGYLYCEGLK+Carbamidomethyl(8)	0.34	0.0173		
	SLIANTCCLVNR+Carbamidomethyl(7)Carbamidomethyl(8)	0.25	0.0080		
Glyma13g33960.1	rhamnose biosynthesis 1	14	0.35	0.0001	cell wall
	Peptide Sequnce	Ratio 4(2)F+Ca	Pvalue 4(2)F+Ca		
	EYDNVCTLR+Carbamidomethyl(6)	0.16	0.1033		
	FVENRPFNDQR	0.22	0.0164		
	GIWNFTNPGAVSHNEILEMYR	0.31	0.1097		
	GNNVYGPNQFPEK	0.66	0.5522		
	ILGWSER	0.17	0.0802		
	LKNEFPELLSIK	0.39	0.4083		
	MPISSDLSNPR	0.25	0.1662		
	NEFPELLSIK	0.27	0.0889		
	NILITGAAGFIASHVANR	0.21	0.0388		
	QGIPYEYGK	0.74	0.6270		
	SYGLPVITTR	0.24	0.0637		
	TGWIGGLLGK	0.45	0.0500		
	TNVAGTLTLADVSR	0.15	0.0655		
	WANFNLEEQAK	0.21	0.0357		
Glyma03g07420.4	metallopeptidase M24 family protein	5	0.35	0.0066	protein
	Peptide Sequnce	Ratio 4(2)F+Ca	Pvalue 4(2)F+Ca		
	AWLALGTK	0.19	0.0889		
	ELDLTSPEVVTK	0.27	0.0829		
	FIFSEISQK	0.52	0.3084		
	IVEGVLSHQMK	0.22	0.0042		
	KGAEAEPMDATNDATPQEQD	0.57	0.5241		
Glyma11g20060.2	ATPase AAA type CDC48 protein	12	0.35	0.0002	cell
	Peptide Sequnce	Ratio 4(2)F+Ca	Pvalue 4(2)F+Ca		
	AIANECQANFISVK+Carbamidomethyl(6)	0.30	0.0897		
	DFSTAILER	0.32	0.1343		
	DTHGYVGADLAALCTEAALQCIR+Carbamidomethyl(14)Carbamidomethyl(21)	0.21	0.0604		
	EIDIGVPDEVGR	0.22	0.0704		
	ELQETVQYPVEHPEK	0.33	0.0992		
	ELVELPLR	0.26	0.1564		
	ETVVEVPNVSWEDIGGLENVK	0.20	0.0354		
	GILLYGPPGSGK	0.25	0.1162		

	GVLFYGPPGCGK+Carbamidomethyl(10)	0.26	0.0989		
	LAGESESNLR	1.96	0.5215		
	LDEVGYDDVGGVR	0.36	0.0318		
	YQAFAQTLQQSR	0.30	0.1399		
Glyma16g19311.1	metallopeptidase M24 family protein	3	0.35	0.0189	protein
	Peptide Sequnce	Ratio 4(2)F+Ca	Pvalue 4(2)F+Ca		
	FIFSEISQK	0.52	0.3084		
	IVEGVLSHQMK	0.22	0.0042		
	VDEAEFEENEVYAIDIVTSTGDGKPK	0.13	0.3885		
Glyma11g11410.1	subtilisin like serine protease 2	4	0.35	0.0273	protein
	Peptide Sequnce	Ratio 4(2)F+Ca	Pvalue 4(2)F+Ca		
	MYQLVYPGK	0.38	0.0650		
	SAMMTTATVLDNR	0.18	0.1652		
	SFSDLNLGPIPR	0.44	0.0737		
	SPQFLGLR	0.08	0.2237		
Glyma10g35381.1	phenylalanine ammonia lyase 2	4	0.35	0.0003	secondary metabolism
	Peptide Sequnce	Ratio 4(2)F+Ca	Pvalue 4(2)F+Ca		
	ALHGGNFQGTPIGVSMDNTR	0.54	0.2223		
	EINSVNDNPLIDVSR	0.22	0.0004		
	INTLLQGYSGIR	0.04	0.0225		
	NPSLDYGFK	0.68	0.2468		
Glyma20g32135.1	phenylalanine ammonia lyase 2	4	0.35	0.0003	secondary metabolism
	Peptide Sequnce	Ratio 4(2)F+Ca	Pvalue 4(2)F+Ca		
	ALHGGNFQGTPIGVSMDNTR	0.54	0.2223		
	EINSVNDNPLIDVSR	0.22	0.0004		
	INTLLQGYSGIR	0.04	0.0225		
	NPSLDYGFK	0.68	0.2468		
Glyma03g21690.1	rotamase FKBP 1	5	0.35	0.0040	protein
	Peptide Sequnce	Ratio 4(2)F+Ca	Pvalue 4(2)F+Ca		
	EGEGYERPNEGAIVK	0.11	0.0002		
	GTPFSFTLGQGQVIK	0.49	0.7364		
	GWDQGIITMK	0.65	0.4984		
	SDGVEFTVNDGYFCPALSK+Carbamidomethyl(14)	0.48	0.3058		
	VLDLESTNVK	0.19	0.1322		
Glyma03g01550.1	Remorin family protein	2	0.35	0.0492	RNA

	Peptide Sequnce	Ratio 4(2)F+Ca	Pvalue 4(2)F+Ca		
	DIALAEIEK	1.56	0.5946		
	IAALEAQLR	0.19	0.0143		
Glyma19g39850.1	RNA binding (RRM/RBD/RNP motifs) family protein	3	0.35	0.0047	RNA
	Peptide Sequnce	Ratio 4(2)F+Ca	Pvalue 4(2)F+Ca		
	AGDVCFSQVFHDGR+Carbamidomethyl(5)	0.28	0.0207		
	GTTGIVDYTNYDDMK	1.03	0.9791		
	VLVTGLPSSASWQDLK	0.42	0.0326		
Glyma03g02240.1	SIN3 associated polypeptide P18	2	0.36	0.0171	RNA
	Peptide Sequnce	Ratio 4(2)F+Ca	Pvalue 4(2)F+Ca		
	IGSHHSMEDFAVR	0.52	0.4944		
	LSFAFVFPDK	0.35	0.0049		
Glyma02g24818.1	Adenylate kinase family protein	2	0.36	0.0078	nucleotide metabolism
	Peptide Sequnce	Ratio 4(2)F+Ca	Pvalue 4(2)F+Ca		
	GFILDGFPR	0.36	0.0210		
	LDEMLQNQGVK	0.38	0.0470		
Glyma10g06170.1	methionine adenosyltransferase 3	5	0.36	0.0000	amino acid metabolism
	Peptide Sequnce	Ratio 4(2)F+Ca	Pvalue 4(2)F+Ca		
	ETFLFTSESVNEGHPDK	0.18	0.1743		
	FVIGGPHGDAGLTGR	0.29	0.0000		
	IIIDTYGGWGAHGGGAFSGK	0.54	0.0079		
	TIFHLNPSGR	0.28	0.0101		
	TNMVMVFGEITTK	0.20	0.1355		
Glyma05g32670.3	S adenosyl L methionine dependent methyltransferases superfamily protein	2	0.37	0.0497	stress
	Peptide Sequnce	Ratio 4(2)F+Ca	Pvalue 4(2)F+Ca		
	LPEDVEIWNEMK	0.39	0.0776		
	VTGEYLTFPGGGTQFK	0.07	0.1116		
Glyma08g00320.1	S adenosyl L methionine dependent methyltransferases superfamily protein	2	0.37	0.0497	stress
	Peptide Sequnce	Ratio 4(2)F+Ca	Pvalue 4(2)F+Ca		
	LPEDVEIWNEMK	0.39	0.0776		
	VTGEYLTFPGGGTQFK	0.07	0.1116		
Glyma13g43970.1	Protein of unknown function DUF642	8	0.37	0.0000	not assigned
	Peptide Sequnce	Ratio 4(2)F+Ca	Pvalue 4(2)F+Ca		
	ADFPEAEIVIHNPGKEEDPACGPLIDSVALK+Carbamidomethyl(21)	0.33	0.0012		
	EEDPACGPLIDSVALK+Carbamidomethyl(6)	0.31	0.0045		

	ESALAQVVITTIGK	0.72	0.0092		
	FLSTFYTMK	0.14	0.1111		
	GSFYSITFSAAR	0.34	0.0336		
	QGDMLLVVPEGDYAVR	0.29	0.0832		
	SDNSGSLCGPVIDDVK+Carbamidomethyl(8)	0.34	0.0095		
	YIDSDHFAVPEGK	0.25	0.0211		
Glyma13g20480.1	methionine adenosyltransferase 3	5	0.37	0.0000	amino acid metabolism
	Peptide Sequnce	Ratio 4(2)F+Ca	Pvalue 4(2)F+Ca		
	ETFLFTSESVNEGHPDK	0.18	0.1743		
	FVIGGPHGDAGLTGR	0.29	0.0000		
	ICDQVSDAILDACLEQDPESK+Carbamidomethyl(2)Carbamidomethyl(13)	0.12	0.1254		
	IIIDTYGGWGAHGGGAFSGK	0.54	0.0079		
Glyma07g35200.1	Nuclear transport factor 2 (NTF2) family protein	4	0.37	0.0000	protein
	Peptide Sequnce	Ratio 4(2)F+Ca	Pvalue 4(2)F+Ca		
	FTQSFFLAPQDK	0.50	0.0217		
	GYFVLNDVFR	0.41	0.0198		
	ILSLDYTSFR	0.29	0.0038		
	SFASIVNALK	0.34	0.0002		
Glyma15g01370.1	Protein of unknown function DUF642	7	0.38	0.0000	not assigned
	Peptide Sequnce	Ratio 4(2)F+Ca	Pvalue 4(2)F+Ca		
	EEDPACGPLIDSVALK+Carbamidomethyl(6)	0.31	0.0045		
	ESALAQVVITTIGK	0.72	0.0092		
	FLSTFYTMK	0.14	0.1111		
	GSFYSITFSAAR	0.34	0.0336		
	QGDMLLVVPEGDYAVR	0.29	0.0832		
	SDNSGSLCGPVIDDVK+Carbamidomethyl(8)	0.34	0.0095		
	YIDSDHFAVPEGK	0.25	0.0211		
Glyma12g30060.1	ATPase AAA type CDC48 protein	15	0.38	0.0002	cell
	Peptide Sequnce	Ratio 4(2)F+Ca	Pvalue 4(2)F+Ca		
	AIANECQANFISVK+Carbamidomethyl(6)	0.30	0.0897		
	DFSTAILER	0.32	0.1343		
	DTHGYVGADLAALCTEAALQCIR+Carbamidomethyl(14)Carbamidomethyl(21)	0.21	0.0604		
	EIDIGVPDEVGR	0.22	0.0704		
	ELQETVQYPVEHPEK	0.33	0.0992		

	ELVELPLR	0.26	0.1564		
	GILLYGPPGSGK	0.25	0.1162		
	GVLFYGPPGCGK+Carbamidomethyl(10)	0.26	0.0989		
	LAGESESNLR	1.96	0.5215		
	LDEVGYDDVGGVR	0.36	0.0318		
	LDQLIYIPLPDEDSR	0.29	0.0746		
	LGDVVSVHQCPDVK+Carbamidomethyl(10)	0.35	0.1444		
	TVFIIGATNRPDIIDPALLRPGR	0.31	0.1523		
	VLNQLLTEMDGMSAK	0.29	0.2509		
	YQAFAQTLQQSR	0.30	0.1399		
Glyma15g27510.3	rhamnose biosynthesis 1	9	0.38	0.0018	cell wall
	Peptide Sequnce	Ratio 4(2)F+Ca	Pvalue 4(2)F+Ca		
	EYDNVCTLR+Carbamidomethyl(6)	0.16	0.1033		
	FILLAMQGK	0.20	0.1294		
	FVENRPFNDQR	0.22	0.0164		
	GNNVYGPNQFPEK	0.66	0.5522		
	MPISSDLSNPR	0.25	0.1662		
	NILITGAAGFIASHVANR	0.21	0.0388		
	QGIPYEYGK	0.74	0.6270		
	SYGLPVITTR	0.24	0.0637		
	TGWIGGLLGK	0.45	0.0500		
Glyma09g37070.3	RNA binding KH domain containing protein	3	0.38	0.0189	RNA
	Peptide Sequnce	Ratio 4(2)F+Ca	Pvalue 4(2)F+Ca		
	ALFQIAAQIR	0.30	0.0323		
	IGDALPGCDER+Carbamidomethyl(8)	0.62	0.6181		
	LLVPSDQIGCVIGK+Carbamidomethyl(10)	0.33	0.0253		
Glyma18g49600.2	RNA binding KH domain containing protein	3	0.38	0.0189	RNA
	Peptide Sequnce	Ratio 4(2)F+Ca	Pvalue 4(2)F+Ca		
	ALFQIAAQIR	0.30	0.0323		
	IGDALPGCDER+Carbamidomethyl(8)	0.62	0.6181		
	LLVPSDQIGCVIGK+Carbamidomethyl(10)	0.33	0.0253		
Glyma17g00710.1	glycosyl hydrolase 9A1	4	0.38	0.0027	cell wall
	Peptide Sequnce	Ratio 4(2)F+Ca	Pvalue 4(2)F+Ca		
	ALLFFNAQK	0.40	0.1435		
	DLVGGYYDAGDAIK	0.08	0.0094		

	WGTDYLLK	0.45	0.0288		
	YVDLGCIIVSR+Carbamidomethyl(6)	0.51	0.1950		
Glyma12g03570.1	subtilisin like serine protease 2	6	0.38	0.0143	protein
	Peptide Sequnce	Ratio 4(2)F+Ca	Pvalue 4(2)F+Ca		
	MYQLVYPGK	0.38	0.0650		
	SAMMTTATVLDNR	0.18	0.1652		
	SFSDLNLGPIPR	0.44	0.0737		
	SPQFLGLR	0.08	0.2237		
	TVTNVGPANSVYR	0.28	0.0216		
	YISSSTNPTATLDFK	1.27	0.9232		
Glyma02g06400.1	succinate dehydrogenase 11	3	0.39	0.0034	TCA / org transformation
	Peptide Sequnce	Ratio 4(2)F+Ca	Pvalue 4(2)F+Ca		
	AVIELENYGLPFSR	0.40	0.0511		
	GSDWLGDQDAIQYMCR+Carbamidomethyl(15)	0.50	0.2670		
	TQETLEEGCQLIDK+Carbamidomethyl(9)	0.38	0.0042		
Glyma15g03761.1	Leucine rich repeat (LRR) family protein	2	0.39	0.0326	not assigned
	Peptide Sequnce	Ratio 4(2)F+Ca	Pvalue 4(2)F+Ca		
	CITTLPFLR+Carbamidomethyl(1)	0.46	0.0656		
	TGYMTGYISPAICK+Carbamidomethyl(13)	0.10	0.0794		
Glyma02g07610.1	xyloglucan endotransglucosylase/hydrolase 5	5	0.39	0.0000	cell wall
	Peptide Sequnce	Ratio 4(2)F+Ca	Pvalue 4(2)F+Ca		
	GFHIDGCEASVNAK+Carbamidomethyl(7)	0.06	0.0013		
	IYNSLWNADDWATR	0.07	0.0069		
	NYVPTWAFDHIK	0.74	0.0002		
	TGQPYILQTNVFTGGK	0.50	0.0038		
	WWDQPEFR	0.04	0.0669		
Glyma19g42090.2	Ribosomal L22e protein family	4	0.39	0.0031	protein
	Peptide Sequnce	Ratio 4(2)F+Ca	Pvalue 4(2)F+Ca		
	AGALGDSITVTR	0.32	0.0438		
	IIVTSDSNFSK	0.31	0.0391		
	IMDIASLEK	0.36	0.0924		
	YFNIAENEGEEED	0.48	0.0587		
Glyma06g10180.1	P450 reductase 1	2	0.39	0.0483	misc

	Peptide Sequnce	Ratio 4(2)F+Ca	Pvalue 4(2)F+Ca		
	LLGQNLDLLFSLHTDK	0.57	0.3247		
	TLHTIVQQQENVDSTK	0.35	0.0257		
Glyma20g04830.1	Ribonuclease T2 family protein	3	0.39	0.0380	RNA
	Peptide Sequnce	Ratio 4(2)F+Ca	Pvalue 4(2)F+Ca		
	ITDLIPR	0.09	0.0338		
	QLNEIYLCADK+Carbamidomethyl(8)	0.08	0.0795		
	YASSFIECPILPSR+Carbamidomethyl(8)	0.72	0.4231		
Glyma06g01280.2	thymidylate synthase 2	2	0.39	0.0075	nucleotide metabolism
	Peptide Sequnce	Ratio 4(2)F+Ca	Pvalue 4(2)F+Ca		
	IILSAWNPVDLK	0.65	0.3550		
	VFVIGGGQIFR	0.23	0.0026		
Glyma08g15680.1	rhamnose biosynthesis 1	8	0.40	0.0029	cell wall
	Peptide Sequnce	Ratio 4(2)F+Ca	Pvalue 4(2)F+Ca		
	FILLAMQGK	0.20	0.1294		
	FVENRPFNDQR	0.22	0.0164		
	GNNVYGPNQFPEK	0.66	0.5522		
	MPISSDLSNPR	0.25	0.1662		
	NILITGAAGFIASHVANR	0.21	0.0388		
	QGIPYEYGK	0.74	0.6270		
	SYGLPVITTR	0.24	0.0637		
	TGWIGGLLGK	0.45	0.0500		
Glyma08g08970.1	urease accessory protein G	5	0.40	0.0262	amino acid metabolism
	Peptide Sequnce	Ratio 4(2)F+Ca	Pvalue 4(2)F+Ca		
	ADILLCESGGDNLAANFSR+Carbamidomethyl(6)	0.28	0.1153		
	AFTVGIGGPVGTGK	0.20	0.0259		
	ENYSLAAVTNDIFTK	0.37	0.1114		
	GGPGITQADLLVINK	0.37	0.1536		
	TDLAPAIGADLAVMQR	0.78	0.7482		
Glyma18g53470.1	Rad23 UV excision repair protein family	3	0.40	0.0296	DNA
	Peptide Sequnce	Ratio 4(2)F+Ca	Pvalue 4(2)F+Ca		
	NEDLAANYLLDHPDEFMPTD	1.05	0.8561		
	VLNDATTLEENK	1.40	0.6346		
	VVENNFVVIMLSK	0.05	0.0017		
Glyma20g38080.1	Ribosomal L22e protein family	4	0.40	0.0060	protein

	Peptide Sequnce	Ratio 4(2)F+Ca	Pvalue 4(2)F+Ca		
	AGALADTITVTR	0.38	0.1020		
	IIVTSDSNFSK	0.31	0.0391		
	IMDIASLEK	0.36	0.0924		
	YFNIAENEGEEED	0.48	0.0587		
Glyma05g31950.2	Small nuclear ribonucleoprotein family protein	3	0.40	0.0268	RNA
	Peptide Sequnce	Ratio 4(2)F+Ca	Pvalue 4(2)F+Ca		
	FMVIPDMLK	0.41	0.1382		
	LLHEASGHVVTVELK	0.39	0.1008		
	TSQLEHVFIR	0.41	0.0572		
Glyma08g15260.1	Small nuclear ribonucleoprotein family protein	3	0.40	0.0268	RNA
	Peptide Sequnce	Ratio 4(2)F+Ca	Pvalue 4(2)F+Ca		
	FMVIPDMLK	0.41	0.1382		
	LLHEASGHVVTVELK	0.39	0.1008		
	TSQLEHVFIR	0.41	0.0572		
Glyma04g08540.1	La protein 1	2	0.41	0.0051	RNA
	Peptide Sequnce	Ratio 4(2)F+Ca	Pvalue 4(2)F+Ca		
	NYIAILDPVTGEAEK	0.65	0.2830		
	TLAVSPFEYDLK	0.25	0.0022		
Glyma02g14450.1	Chalcone and stilbene synthase family protein	5	0.41	0.0004	secondary metabolism
	Peptide Sequnce	Ratio 4(2)F+Ca	Pvalue 4(2)F+Ca		
	ITHLIFCTTSGVDMPGADYQLTK+Carbamidomethyl(7)	0.26	0.0638		
	QDMVVVEVPK	0.65	0.1572		
	VLVVCSEITAVTFR+Carbamidomethyl(5)	0.40	0.0110		
	YMMYQQGCFAGGTVLR+Carbamidomethyl(8)	0.14	0.0091		
	YMYLNEEILK	0.48	0.1203		
Glyma03g33880.1	PHE ammonia lyase 1	5	0.41	0.0012	secondary metabolism
	Peptide Sequnce	Ratio 4(2)F+Ca	Pvalue 4(2)F+Ca		
	ALHGGNFQGTPIGVSMDNTR	0.54	0.2223		
	EINSVNDNPLIDVSR	0.22	0.0004		
	FEILEAITK	0.96	0.9160		
	INTLLQGYSGIR	0.04	0.0225		
	NPSLDYGFK	0.68	0.2468		
Glyma03g33890.1	PHE ammonia lyase 1	5	0.41	0.0012	secondary metabolism
	Peptide Sequnce	Ratio 4(2)F+Ca	Pvalue 4(2)F+Ca		

	ALHGGNFQGTPIGVSMDNTR	0.54	0.2223		
	EINSVNDNPLIDVSR	0.22	0.0004		
	FEILEAITK	0.96	0.9160		
	INTLLQGYSGIR	0.04	0.0225		
	NPSLDYGFK	0.68	0.2468		
Glyma04g40760.1	CTC interacting domain 11	4	0.42	0.0003	RNA
	Peptide Sequnce	Ratio 4(2)F+Ca	Pvalue 4(2)F+Ca		
	AALNLSGTMLGYYPLR	0.70	0.5736		
	DLEELLSK	0.16	0.1190		
	TAIAPVNPTFLPR	0.27	0.0065		
	VCGDPNSILR+Carbamidomethyl(2)	0.49	0.0011		
Glyma06g14030.1	CTC interacting domain 11	4	0.42	0.0003	RNA
	Peptide Sequnce	Ratio 4(2)F+Ca	Pvalue 4(2)F+Ca		
	AALNLSGTMLGYYPLR	0.70	0.5736		
	DLEELLSK	0.16	0.1190		
	TAIAPVNPTFLPR	0.27	0.0065		
	VCGDPNSILR+Carbamidomethyl(2)	0.49	0.0011		
Glyma04g40750.2	CTC interacting domain 11	4	0.42	0.0004	RNA
	Peptide Sequnce	Ratio 4(2)F+Ca	Pvalue 4(2)F+Ca		
	AALSLSGTMLGYYPLR	0.87	0.8078		
	DLEELLSK	0.16	0.1190		
	TAIAPVNPTFLPR	0.27	0.0065		
	VCGDPNSILR+Carbamidomethyl(2)	0.49	0.0011		
Glyma06g14050.3	CTC interacting domain 11	4	0.42	0.0004	RNA.
	Peptide Sequnce	Ratio 4(2)F+Ca	Pvalue 4(2)F+Ca		
	AALSLSGTMLGYYPLR	0.87	0.8078		
	DLEELLSK	0.16	0.1190		
	TAIAPVNPTFLPR	0.27	0.0065		
	VCGDPNSILR+Carbamidomethyl(2)	0.49	0.0011		
Glyma01g42010.3	stress inducible protein putative	9	0.42	0.0002	stress
	Peptide Sequnce	Ratio 4(2)F+Ca	Pvalue 4(2)F+Ca		
	ALELDDEDISYLTNR	0.29	0.0885		
	DYESAIETYQK	0.77	0.4731		
	ELEQQEYFDPK	0.34	0.1704		
	GLEIDPHNEPLK	0.35	0.0386		
	IMQALGVLLNVK	0.18	0.0328		

	LGAMPEGLK	0.26	0.0697		
	LVSAGIVQMK	0.21	0.0449		
	QVLIDFQENPK	0.37	0.0364		
	YDSNNQELLEGIR	0.27	0.0509		
Glyma07g13900.1	Hyaluronan / mRNA binding family	4	0.42	0.0002	RNA
	Peptide Sequnce	Ratio 4(2)F+Ca	Pvalue 4(2)F+Ca		
	DFSNDDNSSAPANQGSFEGDSGNHSER	0.13	0.0017		
	EFASMQPLSNK	0.37	0.0582		
	EMTLEEYEK	0.18	0.0126		
	ENDEIFIK	0.66	0.2207		
Glyma19g36620.1	PHE ammonia lyase 1	6	0.42	0.0017	secondary metabolism
	Peptide Sequnce	Ratio 4(2)F+Ca	Pvalue 4(2)F+Ca		
	ALHGGNFQGTPIGVSMDNTR	0.54	0.2223		
	EINSVNDNPLIDVSR	0.22	0.0004		
	FEILEAITK	0.96	0.9160		
	GSDPLNWGAAAEAMK	0.60	0.3005		
	INTLLQGYSGIR	0.04	0.0225		
	NPSLDYGFK	0.68	0.2468		
Glyma01g38320.1	Tyrosyl tRNA synthetase class Ib bacterial/mitochondrial	2	0.43	0.0354	protein
	Peptide Sequnce	Ratio 4(2)F+Ca	Pvalue 4(2)F+Ca		
	TVEGNPCLEYIK+Carbamidomethyl(7)	0.41	0.0240		
	YLIEIWK	0.54	0.2395		
Glyma11g07250.1	succinate dehydrogenase 11	6	0.43	0.0004	TCA / org transformation
	Peptide Sequnce	Ratio 4(2)F+Ca	Pvalue 4(2)F+Ca		
	AASTILATGGYGR	0.26	0.0755		
	AFGGQSLNYGK	0.52	0.0366		
	AVIELENYGLPFSR	0.40	0.0511		
	GSDWLGDQDAIQYMCR+Carbamidomethyl(15)	0.50	0.2670		
	TQETLEEGCQLIDK+Carbamidomethyl(9)	0.38	0.0042		
	TWESFHDVQVK	0.57	0.1237		
Glyma07g00540.1	D-3-phosphoglycerate dehydrogenase	7	0.43	0.0122	amino acid metabolism
	Peptide Sequnce	Ratio 4(2)F+Ca	Pvalue 4(2)F+Ca		
	AASTILATGGYGR	0.26	0.0755		
	GGVIDEDALVR	0.65	0.1572		

	IFNDNTFAK	0.25	0.0436		
	LAVQLVCGGSGIK+Carbamidomethyl(7)	0.06	0.2555		
	LGEAGLQVLR	0.25	0.0508		
	VGNILGEQNVNVSFMSVGR	0.69	0.4542		
	YVGVSMVGK	0.63	0.3705		
Glyma08g23860.1	D-3-phosphoglycerate dehydrogenase	7	0.43	0.0122	amino acid metabolism
	Peptide Sequnce	Ratio 4(2)F+Ca	Pvalue 4(2)F+Ca		
	AIMAIGVDEEPNK	0.25	0.0430		
	GGVIDEDALVR	0.65	0.1572		
	IFNDNTFAK	0.25	0.0436		
	LAVQLVCGGSGIK+Carbamidomethyl(7)	0.06	0.2555		
	LGEAGLQVLR	0.25	0.0508		
	VGNILGEQNVNVSFMSVGR	0.69	0.4542		
	YVGVSMVGK	0.63	0.3705		
Glyma01g38200.1	succinate dehydrogenase 11	5	0.43	0.0008	TCA / org transformation
	Peptide Sequnce	Ratio 4(2)F+Ca	Pvalue 4(2)F+Ca		
	AFGGQSLNYGK	0.52	0.0366		
	AVIELENYGLPFSR	0.40	0.0511		
	GSDWLGDQDAIQYMCR+Carbamidomethyl(15)	0.50	0.2670		
	TQETLEEGCQLIDK+Carbamidomethyl(9)	0.38	0.0042		
	TWESFHDVQVK	0.57	0.1237		
Glyma06g04100.2	RNA binding protein 47C	2	0.43	0.0190	RNA
	Peptide Sequnce	Ratio 4(2)F+Ca	Pvalue 4(2)F+Ca		
	QPFSQYGEIVSVK	0.42	0.0031		
	VLQNYAGILMPNTEQPFR	0.78	0.8877		
Glyma17g17850.1	Subtilase family protein	9	0.43	0.0000	protein
	Peptide Sequnce	Ratio 4(2)F+Ca	Pvalue 4(2)F+Ca		
	AVGPTGLPVDNR	0.24	0.0273		
	GGCFSSDILAAIER+Carbamidomethyl(3)	0.42	0.0633		
	HLVGSPISVNWG	0.44	0.0076		
	ISVEPQVLSFK	0.35	0.0527		
	SALMTTAYTVYK	0.54	0.0011		
	SFDDTGLGPVPSTWK	0.29	0.0007		
	TFTVTFSSSGSPQHTENAFGR	0.91	0.6622		

	TPLFLGLDK	0.29	0.0029		
	VDFNIISGTSMSCPHVSGLAALIK+Carbamidomethyl(13)	1.82	0.3654		
Glyma04g42460.1	2 oxoglutarate (2OG) and Fe(II) dependent oxygenase superfamily protein	4	0.44	0.0202	hormone metabolism
	Peptide Sequnce	Ratio 4(2)F+Ca	Pvalue 4(2)F+Ca		
	ATICPAPQLVEK+Carbamidomethyl(4)	0.33	0.0670		
	FVFGDYMSVYAEQK	0.65	0.5333		
	SIASFYNPSFK	0.40	0.0153		
	VGGLQMLK	0.45	0.2101		
Glyma07g12190.1	hexokinase 1	4	0.44	0.0313	major CHO metabolism
	Peptide Sequnce	Ratio 4(2)F+Ca	Pvalue 4(2)F+Ca		
	GLFYALDLGGTNFR	0.75	0.2584		
	IVVELCDIVATR+Carbamidomethyl(6)	0.37	0.0020		
	MLITYVDNLPSGDEK	0.56	0.4817		
	TPDMSAMHHDTSSDLK	0.90	0.8678		
Glyma12g03070.1	nucleolin like 2	11	0.44	0.0000	protein
	Peptide Sequnce	Ratio 4(2)F+Ca	Pvalue 4(2)F+Ca		
	ADVEDFFK	0.38	0.0000		
	ALGLNGQQLFNR	0.57	0.0994		
	DAGEVVDVR	0.73	0.3935		
	GAYTPNSSNWNNSSQK	1.20	0.9852		
	GFAYVDFSDVDSMGK	0.36	0.0471		
	GFDTSLGEDEIR	0.34	0.1237		
	GFGHVEFATAAAAQK	0.29	0.0746		
	GSLQEHFGSCGDITR+Carbamidomethyl(10)	0.40	0.0164		
	LPALSVAPALK	0.35	0.0284		
	TLFVGNLPFSVER	0.67	0.0186		
	VDAVPAVVPPSK	0.35	0.0100		
Glyma08g11520.1	Chalcone and stilbene synthase family protein	5	0.45	0.0013	secondary metabolism
	Peptide Sequnce	Ratio 4(2)F+Ca	Pvalue 4(2)F+Ca		
	EVGLTFHLLK	0.91	0.6916		
	ITHLIFCTTSGVDMPGADYQLTK+Carbamidomethyl(7)	0.26	0.0638		
	VLVVCSEITAVTFR+Carbamidomethyl(5)	0.40	0.0110		
	YMMYQQGCFAGGTVLR+Carbamidomethyl(8)	0.14	0.0091		
	YMYLNEEILK	0.48	0.1203		
Glyma14g17930.1	serine/argininerich 22	2	0.45	0.0107	RNA

	Peptide Sequnce	Ratio 4(2)F+Ca	Pvalue 4(2)F+Ca		
	DLEDEFR	0.46	0.0098		
	RPPGYAFIDFDDR	0.32	0.1440		
Glyma17g29080.1	serine/argininerich 22	2	0.45	0.0107	RNA
	Peptide Sequnce	Ratio 4(2)F+Ca	Pvalue 4(2)F+Ca		
	DLEDEFR	0.46	0.0098		
	RPPGYAFIDFDDR	0.32	0.1440		
Glyma17g37270.2	beta galactosidase 5	4	0.46	0.0023	misc
	Peptide Sequnce	Ratio 4(2)F+Ca	Pvalue 4(2)F+Ca		
	ITASGLLEQISTTR	0.41	0.0032		
	LLSWETYDEDVSSLAESSK	0.65	0.3588		
	STPEMWEDLIGK	0.26	0.0710		
	WSYQIGLR	0.17	0.0753		
Glyma15g06020.1	3 deoxy d arabino heptulosonate 7 phosphate synthase	2	0.46	0.0142	amino acid metabolism
	Peptide Sequnce	Ratio 4(2)F+Ca	Pvalue 4(2)F+Ca		
	AFATGGYAAMQR	0.34	0.2138		
	GVANPLGIK	0.46	0.0092		
Glyma01g41990.1	Glycosyl hydrolases family 32 protein	8	0.46	0.0014	major CHO metabolism
	Peptide Sequnce	Ratio 4(2)F+Ca	Pvalue 4(2)F+Ca		
	DFRDPTTAWITSEGK	1.66	0.4382		
	DPTTAWITSEGK	0.67	0.0484		
	IFGSFVPVLK	0.18	0.0001		
	ILVDHSIVESFAQGGR	0.78	0.5573		
	NWMNDPNGPMYYK	0.76	0.6627		
	VLWGWIGESDSEYADVAK	0.46	0.0340		
	VSLDDDRHDYYALGTYDEK	2.06	0.5355		
	YDYGIFYASK	0.77	0.4456		
Glyma05g33860.1	Pathogenesis related thaumatin superfamily protein	2	0.46	0.0081	stress
	Peptide Sequnce	Ratio 4(2)F+Ca	Pvalue 4(2)F+Ca		
	AYSLQLPALWSGR	0.48	0.0334		
	AYSYAYDDPTSIATCTK+Carbamidomethyl(15)	0.24	0.0306		
Glyma08g05820.1	Pathogenesis related thaumatin superfamily protein	2	0.46	0.0081	stress
	Peptide Sequnce	Ratio 4(2)F+Ca	Pvalue 4(2)F+Ca		
	AYSLQLPALWSGR	0.48	0.0334		

	AYSYAYDDPTSIATCTK+Carbamidomethyl(15)	0.24	0.0306		
Glyma05g27840.1	urease	8	0.46	0.0019	amino acid metabolism
	Peptide Sequnce	Ratio 4(2)F+Ca	Pvalue 4(2)F+Ca		
	DFALYGDECVFGGGK+Carbamidomethyl(9)	0.29	0.0311		
	DGLIVSIGK	0.66	0.4981		
	GPLQPGESDNDNFR	0.45	0.1418		
	GSSSKPDELHDIIK	0.52	0.0255		
	LGLHNAGYLAQK	0.43	0.1018		
	LMLQSTDDLPLNFGFTGK	0.52	0.3779		
	LNIAAGTAVR	0.46	0.0996		
	TIHTYHSEGAGGGHAPDIIK	0.36	0.0362		
Glyma18g49189.1	Hyaluronan / mRNA binding family	3	0.47	0.0078	RNA
	Peptide Sequnce	Ratio 4(2)F+Ca	Pvalue 4(2)F+Ca		
	EFASMQPLSNK	0.37	0.0582		
	EMTLEEYEK	0.18	0.0126		
	ENDEIFIK	0.66	0.2207		
Glyma05g22060.1	Subtilase family protein	7	0.47	0.0000	protein
	Peptide Sequnce	Ratio 4(2)F+Ca	Pvalue 4(2)F+Ca		
	AVGPTGLPVDNR	0.24	0.0273		
	GGCFSSDILAAIER+Carbamidomethyl(3)	0.42	0.0633		
	HVVGTPISINWG	0.81	0.7993		
	ISVEPQVLSFK	0.35	0.0527		
	SALMTTAYTVYK	0.54	0.0011		
	SFDDTGLGPVPSTWK	0.29	0.0007		
	VDFNIISGTSMSCPHVSGLAALIK+Carbamidomethyl(13)	1.82	0.3654		
Glyma14g07700.1	beta galactosidase 5	2	0.47	0.0090	misc
	Peptide Sequnce	Ratio 4(2)F+Ca	Pvalue 4(2)F+Ca		
	ITASGLLEQISTTR	0.41	0.0032		
	LLSWETYDEDVSSLAESSK	0.65	0.3588		
Glyma08g03730.1	hexokinase 1	8	0.48	0.0181	major CHO metabolism
	Peptide Sequnce	Ratio 4(2)F+Ca	Pvalue 4(2)F+Ca		
	ELGFTFSFPVR	0.56	0.0349		
	IVVELCDIVATR+Carbamidomethyl(6)	0.37	0.0020		
	LAAAGILGILK	0.62	0.1646		

	LISGMYLGEVVR	1.16	0.6360		
	SGDMVINMEWGNFR	0.83	0.8279		
	SVIALDGGLFEHYTK	0.59	0.2341		
	TPDMSAMHHDTSSDLK	0.90	0.8678		
	VAALVNDTIGTLAGGR	0.67	0.2520		
Glyma13g43630.1	heat shock protein 91	24	0.48	0.0000	stress
	Peptide Sequnce	Ratio 4(2)F+Ca	Pvalue 4(2)F+Ca		
	ALAEAGLTVENVHMVEVVGSGSR	0.36	0.3001		
	ATANAPGAENGTPEAGDKPVQMDTDTK	0.24	0.0533		
	AVLDAATIAGLHPLR	0.52	0.1778		
	DEFEQLSLPILER	0.75	0.6858		
	DFDEVLFNHFAAK	0.37	0.0963		
	EFEMALQDR	0.08	0.0256		
	ETPAIVCFGDK+Carbamidomethyl(7)	0.58	0.2104		
	FLGTAGAASTMMNPK	0.64	0.3904		
	GCALQCAILSPTFK+Carbamidomethyl(2)Carbamidomethyl(6)	0.28	0.0034		
	GIDVVLNDESKR	0.32	0.0609		
	GTVIDQLAYCINSYR+Carbamidomethyl(10)	0.34	0.0496		
	IDVFQNAR	0.85	0.6871		
	ISTYTIGPFQSTK	0.39	0.1552		
	LFHETTATALAYGIYK	0.39	0.0467		
	LQEVEDWLYEDGEDETK	0.43	0.0424		
	NAVEAYVYDMR	0.50	0.1403		
	QFADPELQQDIK	0.36	0.0423		
	RDEFEQLSLPILER	0.42	0.1585		
	SGTFSIDVQYDDVSGLQTPAK	0.53	0.2018		
	TFPFVVTEGPDGYPLIHAR	0.44	0.0303		
	VLNECVEAENWLR+Carbamidomethyl(5)	0.25	0.0214		
	VLSANPEAPLNIECLMDEK+Carbamidomethyl(14)	0.40	0.1376		
	YATPVLLSADVR	0.45	0.0065		
	YQEFVIDSER	0.44	0.0847		
Glyma18g33150.1	Nuclear transport factor 2 (NTF2) family protein	3	0.48	0.0424	protein
	Peptide Sequnce	Ratio 4(2)F+Ca	Pvalue 4(2)F+Ca		

	EGAAPSSTVTPVSVK	0.52	0.0864		
	GLPPTATPAVLENEFK	0.84	0.8620		
	GYFVLNDVFR	0.41	0.0198		
Glyma11g33880.1	DegP protease 7	5	0.48	0.0027	protein
	Peptide Sequnce	Ratio 4(2)F+Ca	Pvalue 4(2)F+Ca		
	ALGFLPEEGHGVYVAR	0.34	0.0377		
	FGCSTSEDHQFVR+Carbamidomethyl(3)	0.32	0.0651		
	GIPIYAISQVLDK	0.12	0.0581		
	ILEVELYPTLLSK	0.32	0.0280		
	VSILAGTLAR	0.75	0.3531		
Glyma18g04400.1	DegP protease 7	5	0.48	0.0027	protein
	Peptide Sequnce	Ratio 4(2)F+Ca	Pvalue 4(2)F+Ca		
	ALGFLPEEGHGVYVAR	0.34	0.0377		
	FGCSTSEDHQFVR+Carbamidomethyl(3)	0.32	0.0651		
	GIPIYAISQVLDK	0.12	0.0581		
	ILEVELYPTLLSK	0.32	0.0280		
	VSILAGTLAR	0.75	0.3531		
Glyma09g02160.1	glycosyl hydrolase 9A1	5	0.48	0.0043	cell wall
	Peptide Sequnce	Ratio 4(2)F+Ca	Pvalue 4(2)F+Ca		
	DLVGGYYDAGDAIK	0.08	0.0094		
	LFLSPGYPYEEILR	0.02	0.0153		
	SQIDYILGNNPR	1.16	0.6163		
	WGTDYFLK	0.00	0.1577		
	YVDLGCIIVSR+Carbamidomethyl(6)	0.51	0.1950		
Glyma09g36870.1	Transducin/WD40 repeat like superfamily protein	4	0.49	0.0167	protein
	Peptide Sequnce	Ratio 4(2)F+Ca	Pvalue 4(2)F+Ca		
	DGDLLFSCAK+Carbamidomethyl(8)	0.54	0.1044		
	IADDPTEQSGESLLLIK	0.38	0.0271		
	ILQEEIGGVK	0.51	0.1471		
	LHHFDPDYFNIK	0.52	0.2116		
Glyma04g11160.3	unknown protein	3	0.49	0.0402	not assigned
	Peptide Sequnce	Ratio 4(2)F+Ca	Pvalue 4(2)F+Ca		
	ELSGTLESEDSILK	0.38	0.0353		
	EMSGSNIFADGK	0.64	0.3001		

	FAELSGNDIFK	0.58	0.1292		
Glyma06g10930.2	unknown protein	3	0.49	0.0402	not assigned
	Peptide Sequnce	Ratio 4(2)F+Ca	Pvalue 4(2)F+Ca		
	ELSGTLESEDSILK	0.38	0.0353		
	EMSGSNIFADGK	0.64	0.3001		
	FAELSGNDIFK	0.58	0.1292		
Glyma02g02140.1	Ribosomal L22e protein family	5	0.49	0.0216	protein
	Peptide Sequnce	Ratio 4(2)F+Ca	Pvalue 4(2)F+Ca		
	AGALGDSITVTR	0.32	0.0438		
	GASFVIDCAKPVEDK+Carbamidomethyl(8)	0.14	0.1269		
	IMDIASLEK	0.36	0.0924		
	ITVTSDSNFSK	0.96	0.9464		
	YFNIAENEGEEED	0.48	0.0587		
Glyma10g02270.1	Ribosomal L22e protein family	5	0.49	0.0216	protein
	Peptide Sequnce	Ratio 4(2)F+Ca	Pvalue 4(2)F+Ca		
	AGALGDSITVTR	0.32	0.0438		
	GASFVIDCAKPVEDK+Carbamidomethyl(8)	0.14	0.1269		
	IMDIASLEK	0.36	0.0924		
	ITVTSDSNFSK	0.96	0.9464		
	YFNIAENEGEEED	0.48	0.0587		
Glyma01g22880.1	Chalcone and stilbene synthase family protein	6	0.49	0.0011	secondary metabolism
	Peptide Sequnce	Ratio 4(2)F+Ca	Pvalue 4(2)F+Ca		
	EVGLTFHLLK	0.91	0.6916		
	ITHLIFCTTSGVDMPGADYQLTK+Carbamidomethyl(7)	0.26	0.0638		
	QDMVVVEVPK	0.65	0.1572		
	VLVVCSEITAVTFR+Carbamidomethyl(5)	0.40	0.0110		
	YMMYQQGCFAGGTVLR+Carbamidomethyl(8)	0.14	0.0091		
	YMYLNEEILK	0.48	0.1203		
Glyma05g28610.1	Chalcone and stilbene synthase family protein	6	0.49	0.0011	secondary metabolism
	Peptide Sequnce	Ratio 4(2)F+Ca	Pvalue 4(2)F+Ca		
	EVGLTFHLLK	0.91	0.6916		
	ITHLIFCTTSGVDMPGADYQLTK+Carbamidomethyl(7)	0.26	0.0638		
	QDMVVVEVPK	0.65	0.1572		
	VLVVCSEITAVTFR+Carbamidomethyl(5)	0.40	0.0110		
	YMMYQQGCFAGGTVLR+Carbamidomethyl(8)	0.14	0.0091		

	YMYLNEEILK	0.48	0.1203		
Glyma08g11530.1	Chalcone and stilbene synthase family protein	6	0.49	0.0011	secondary metabolism
	Peptide Sequnce	Ratio 4(2)F+Ca	Pvalue 4(2)F+Ca		
	EVGLTFHLLK	0.91	0.6916		
	ITHLIFCTTSGVDMPGADYQLTK+Carbamidomethyl(7)	0.26	0.0638		
	QDMVVVEVPK	0.65	0.1572		
	VLVVCSEITAVTFR+Carbamidomethyl(5)	0.40	0.0110		
	YMMYQQGCFAGGTVLR+Carbamidomethyl(8)	0.14	0.0091		
	YMYLNEEILK	0.48	0.1203		
Glyma08g11620.1	Chalcone and stilbene synthase family protein	6	0.49	0.0011	secondary metabolism
	Peptide Sequnce	Ratio 4(2)F+Ca	Pvalue 4(2)F+Ca		
	EVGLTFHLLK	0.91	0.6916		
	ITHLIFCTTSGVDMPGADYQLTK+Carbamidomethyl(7)	0.26	0.0638		
	QDMVVVEVPK	0.65	0.1572		
	VLVVCSEITAVTFR+Carbamidomethyl(5)	0.40	0.0110		
	YMMYQQGCFAGGTVLR+Carbamidomethyl(8)	0.14	0.0091		
	YMYLNEEILK	0.48	0.1203		
Glyma09g08780.1	Chalcone and stilbene synthase family protein	6	0.49	0.0011	secondary metabolism
	Peptide Sequnce	Ratio 4(2)F+Ca	Pvalue 4(2)F+Ca		
	EVGLTFHLLK	0.91	0.6916		
	ITHLIFCTTSGVDMPGADYQLTK+Carbamidomethyl(7)	0.26	0.0638		
	QDMVVVEVPK	0.65	0.1572		
	VLVVCSEITAVTFR+Carbamidomethyl(5)	0.40	0.0110		
	YMMYQQGCFAGGTVLR+Carbamidomethyl(8)	0.14	0.0091		
	YMYLNEEILK	0.48	0.1203		
Glyma11g01350.1	Chalcone and stilbene synthase family protein	6	0.49	0.0011	secondary metabolism
	Peptide Sequnce	Ratio 4(2)F+Ca	Pvalue 4(2)F+Ca		
	EVGLTFHLLK	0.91	0.6916		
	ITHLIFCTTSGVDMPGADYQLTK+Carbamidomethyl(7)	0.26	0.0638		
	QDMVVVEVPK	0.65	0.1572		
	VLVVCSEITAVTFR+Carbamidomethyl(5)	0.40	0.0110		
	YMMYQQGCFAGGTVLR+Carbamidomethyl(8)	0.14	0.0091		
	YMYLNEEILK	0.48	0.1203		

Glyma08g11610.1	Chalcone and stilbene synthase family protein	5	0.49	0.0016	secondary metabolism
	Peptide Sequnce	Ratio 4(2)F+Ca	Pvalue 4(2)F+Ca		
	EVGLTFHLLK	0.91	0.6916		
	ITHLIFCTTSGVDMPGADYQLTK+Carbamidomethyl(7)	0.26	0.0638		
	QDMVVVEVPK	0.65	0.1572		
	VLVVCSEITAVTFR+Carbamidomethyl(5)	0.40	0.0110		
	YMMYQQGCFAGGTVLR+Carbamidomethyl(8)	0.14	0.0091		
Glyma01g41310.1	glutamate dehydrogenase 2	2	0.49	0.0048	N-metabolism
	Peptide Sequnce	Ratio 4(2)F+Ca	Pvalue 4(2)F+Ca		
	LENSLLIPFR	0.50	0.0040		
	TAVADIPYGGAK	0.48	0.1424		
Glyma02g35640.1	CTC interacting domain 11	2	0.49	0.0052	RNA
	Peptide Sequnce	Ratio 4(2)F+Ca	Pvalue 4(2)F+Ca		
	AALNLSGTMLGYYPLR	0.70	0.5736		
	VCGDPNSILR+Carbamidomethyl(2)	0.49	0.0011		
Glyma16g34120.1	Translation initiation factor eIF3 subunit	5	0.50	0.0028	protein.synthesis
	Peptide Sequnce	Ratio 4(2)F+Ca	Pvalue 4(2)F+Ca		
	DIASSVTAIANEK	0.56	0.0553		
	ESWEDEDEPAPAPAAPAVK	0.35	0.0789		
	SESDFLEYAELISHK	0.49	0.2214		
	SNWDDEDVDENDVK	0.05	0.0211		
	TVEPIKEEPLDPVAEK	0.34	0.0735		
Glyma19g31590.1	beta-1, 3-glucanase 1	4	2.05	0.0403	misc
	Peptide Sequnce	Ratio 4(2)F+Ca	Pvalue 4(2)F+Ca		
	AGGGSLNIVVSESGWPSSGGTATSLDNAR	1.56	0.2175		
	DISLDYALFR	1.54	0.4757		
	SPSVVVQDGSLGYR	1.96	0.3495		
	TYNTNLVR	6.67	0.0086		
Glyma09g00711.1	Glycine cleavage Tprotein family	3	2.06	0.0390	photosynthesis
	Peptide Sequnce	Ratio 4(2)F+Ca	Pvalue 4(2)F+Ca		
	AEGGFLGADVILK	2.19	0.0243		
	MPFVPTK	0.39	0.4342		
	TGYTGEDGFEISIPSESAVDLAK	0.36	0.1250		

Glyma11g14950.1	heat shock protein 70	25	2.22	0.0017	stress
	Peptide Sequnce	Ratio 4(2)F+Ca	Pvalue 4(2)F+Ca		
	ARFEELNMDLFR	0.99	0.9893		
	ATAGDTHLGGEDFDNR	0.74	0.8082		
	DAGVISGLNVMR	0.52	0.1271		
	ELESICNPIIAK+Carbamidomethyl(6)	0.46	0.1300		
	EQVFSTYSDNQPGVLIQVYEGER	0.66	0.1781		
	FEELNMDLFR	0.55	0.1527		
	FEELNMDLFRK	0.20	0.2309		
	FELSGIPPAPR	0.34	0.0793		
	FSDASVQGDMK	0.88	0.8496		
	GEGPAIGIDLGTTYSCVGVWQHDR+Carbamidomethyl(16)	0.56	0.0755		
	IINEPTAAAIAYGLDK	0.78	0.0046		
	IINEPTAAAIAYGLDKK	0.64	0.2851		
	ITITNDK	14.56	0.0519		
	MKELESICNPIIAK+Carbamidomethyl(8)	0.77	0.4170		
	MVNHFVQEFK	0.77	0.3969		
	MYQGAGGDAGGAMDEDGPAAGSGSGAGPK	0.60	0.5099		
	NALENYAYNMR	0.80	0.2343		
	NALENYAYNMR+Oxidation(10)	0.88	0.6590		
	NAVVTVPAYFNDSQR	0.71	0.0508		
	NQVAMNPTNTVFDAK	0.60	0.1333		
	QFSAEEISSMVLMK	0.56	0.2360		
	TTPSYVAFTDTER	0.60	0.0582		
	VEIIANDQGNR	96.07	0.1311		
	VQQLLQDFFNGK	0.72	0.2363		
	VQQLLQDFFNGKELCK+Carbamidomethyl(15)	0.50	0.4267		
Glyma08g36700.1	ARFGAP domain 8	6	2.26	0.0012	protein
	Peptide Sequnce	Ratio 4(2)F+Ca	Pvalue 4(2)F+Ca		
	DNNIDLTAGDLINR	0.50	0.0238		
	LSFQAQQDLSSLK	0.65	0.1209		
	LSSLASTLMTDLQDR	0.87	0.2464		
	SISSSQYFGDQNK	5.12	0.0066		

	SSSFFADFGMDSGFPK	0.74	0.6022		
	STNLDSWSPEQLK	0.49	0.0312		
Glyma17g14950.1	Lactate/malate dehydrogenase family protein	4	2.28	0.0009	fermentation
	Peptide Sequnce	Ratio 4(2)F+Ca	Pvalue 4(2)F+Ca		
	AIIPPLVR	1.78	0.0675		
	GEMLDLQHAAAFLPR	2.47	0.0383		
	GYTSWAIGYSVANLAR	1.59	0.0563		
	VIGSGTNLDSSR	8.63	0.0130		
Glyma03g02760.1	N.D.*	6	2.29	0.0000	not assigned
	Peptide Sequnce	Ratio 4(2)F+Ca	Pvalue 4(2)F+Ca		
	ILLGYSAIK	3.12	0.0198		
	IVGEYDYYDSK	1.79	0.2161		
	LLSLAVSDAGK	2.34	0.0402		
	LNCDASGLPVK+Carbamidomethyl(3)	1.73	0.0334		
	VFVSIGGDDPFK	2.03	0.0136		
	WDTDNVTPQLIEQFK	2.96	0.0008		
Glyma15g07850.3	Aluminium induced protein with YGL and LRDR motifs	2	2.30	0.0347	hormone metabolism
	Peptide Sequnce	Ratio 4(2)F+Ca	Pvalue 4(2)F+Ca		
	GPYPADQVLK	2.73	0.1057		
	GTNEAMFITEAYR	2.11	0.0531		
Glyma15g30610.1	Calcium-binding EF-hand family protein	5	2.30	0.0000	signalling
	Peptide Sequnce	Ratio 4(2)F+Ca	Pvalue 4(2)F+Ca		
	GVVVIDGSTVR	2.19	0.0005		
	LYDSIFDK	2.63	0.0396		
	LYDSIFDKFDGDR	4.88	0.1869		
	MVLEDDPNSLLQK	2.72	0.0865		
	SVDEQFGVLDLNNDGVLSR	4.38	0.0136		
Glyma13g07220.1	glycosyl hydrolase family 81 protein	3	2.30	0.0000	stress
	Peptide Sequnce	Ratio 4(2)F+Ca	Pvalue 4(2)F+Ca		
	DSGDLLLLAHPLHVQLLR	11.59	0.0489		
	DTGLWFAPAEWK	4.15	0.0081		
	ELVEWTLPALDR	2.23	0.0004		
Glyma18g52650.2	heat shock cognate protein 701	22	2.31	0.0000	stress

	Peptide Sequnce	Ratio 4(2)F+Ca	Pvalue 4(2)F+Ca		
	ARFEELNMDLFR	0.99	0.9893		
	ATAGDTHLGGEDFDNR	0.74	0.8082		
	DAGVIAGLNVMR	0.66	0.2075		
	EIAEAYLGSTVK	0.73	0.1558		
	ELEGICNPIIAK+Carbamidomethyl(6)	0.17	0.0009		
	EQVFSTYSDNQPGVLIQVYEGER	0.66	0.1781		
	FEELNMDLFR	0.55	0.1527		
	FEELNMDLFRK	0.20	0.2309		
	FELSGIPPAPR	0.34	0.0793		
	IINEPTAAAIAYGLDK	0.78	0.0046		
	IINEPTAAAIAYGLDKK	0.64	0.2851		
	ITITNDK	14.56	0.0519		
	MVNHFVQEFK	0.77	0.3969		
	NALENYAYNMR	0.80	0.2343		
	NALENYAYNMR+Oxidation(10)	0.88	0.6590		
	NAVVTVPAYFNDSQR	0.71	0.0508		
	NQVAMNPINTVFDAK	0.66	0.2136		
	NQVAMNPINTVFDAK+Oxidation(5)	1.47	0.0352		
	NQVAMNPINTVFDAKR	0.02	0.3462		
	TTPSYVAFTDTER	0.60	0.0582		
	VEIIANDQGNR	96.07	0.1311		
	VQQLLQDFFNGK	0.72	0.2363		
Glyma18g52610.1	heat shock cognate protein 701	25	2.31	0.0046	stress
	Peptide Sequnce	Ratio 4(2)F+Ca	Pvalue 4(2)F+Ca		
	ARFEELNMDLFR	0.99	0.9893		
	ATAGDTHLGGEDFDNR	0.74	0.8082		
	DAGVIAGLNVMR	0.66	0.2075		
	EIAEAYLGSTVK	0.73	0.1558		
	ELESICNPIIAK+Carbamidomethyl(6)	0.46	0.1300		
	EQVFSTYSDNQPGVLIQVYEGER	0.66	0.1781		
	FEELNMDLFR	0.55	0.1527		
	FEELNMDLFRK	1.02	0.7897		

	FELSGIPPAPR	0.34	0.0793		
	FSDASVQSDMK	1.66	0.6807		
	IINEPTAAAIAYGLDK	0.78	0.0046		
	IINEPTAAAIAYGLDKK	0.64	0.2851		
	ITITNDK	14.56	0.0519		
	MKELESICNPIIAK+Carbamidomethyl(8)	0.77	0.4170		
	MVNHFVQEFK	0.77	0.3969		
	NALENYAYNMR	0.80	0.2343		
	NALENYAYNMR+Oxidation(10)	0.88	0.6590		
	NAVVTVPAYFNDSQR	0.71	0.0508		
	NQVAMNPVNTVFDAK	0.72	0.3015		
	QFSAEEISSMVLMK	0.56	0.2360		
	TTPSYVAFTDSER	0.56	0.0703		
	VEIIANDQGNR	96.07	0.1311		
	VIPGPADKPMIVVNYK	0.70	0.6770		
	VQQLLQDFFNGK	0.72	0.2363		
	VQQLLQDFFNGKELCK+Carbamidomethyl(15)	0.50	0.4267		
Glyma13g19331.1	heat shock cognate protein 701	26	2.32	0.0002	stress
	Peptide Sequnce	Ratio 4(2)F+Ca	Pvalue 4(2)F+Ca		
	ARFEELNMDLFR	0.99	0.9893		
	ATAGDTHLGGEDFDNR	0.74	0.8082		
	DAGVIAGLNVMR	0.66	0.2075		
	EIAEAYLGSSIK	0.73	0.1558		
	ELESICNPIIAK+Carbamidomethyl(6)	0.46	0.1300		
	EQVFSTYSDNQPGVLIQVYEGER	0.66	0.1781		
	FEELNMDLFR	0.55	0.1527		
	FEELNMDLFRK	0.20	0.2309		
	FELSGIPPAPR	0.34	0.0793		
	FSDASVQSDIK	0.65	0.0978		
	GEGPAIGIDLGTTYSCVGVWQHDR+Carbamidomethyl(16)	0.56	0.0755		
	IINEPTAAAIAYGLDK	0.78	0.0046		
	IINEPTAAAIAYGLDKK	0.64	0.2851		
	ITITNDK	14.56	0.0519		

	MKELESICNPIIAK+Carbamidomethyl(8)	0.77	0.4170		
	MVNHFVQEFK	0.77	0.3969		
	MYQGGAGPDVGGAMDDDVPAAGSGAGPK	0.28	0.2720		
	NALENYSYNMR	0.64	0.1238		
	NAVVTVPAYFNDSQR	0.71	0.0508		
	NQVAMNPINTVFDAK	0.66	0.2136		
	NQVAMNPINTVFDAK+Oxidation(5)	1.47	0.0352		
	NQVAMNPINTVFDAKR	0.02	0.3462		
	QFAAEEISSMVLMK	0.60	0.2970		
	TTPSYVGFTDTER	0.66	0.0370		
	VEIIANDQGNR	96.07	0.1311		
	VLSGPAEKPMIQVSYK	0.96	0.9764		
Glyma08g25006.1	Calcium-binding EF-hand family protein	6	2.32	0.0000	signalling
	Peptide Sequnce	Ratio 4(2)F+Ca	Pvalue 4(2)F+Ca		
	DFVNDETAFTK	2.53	0.0088		
	GVVVIDGSTVR	2.19	0.0005		
	LIETHFGIDVSTPPEQLAK	2.46	0.0136		
	LYDSIFDK	2.63	0.0396		
	LYDSIFDKFDGDR	4.88	0.1869		
	MVLEDDPNSLLQK	2.72	0.0865		
Glyma02g41890.3	type one serine/threonine protein phosphatase 4	3	2.33	0.0039	protein
	Peptide Sequnce	Ratio 4(2)F+Ca	Pvalue 4(2)F+Ca		
	IYGFYDECK+Carbamidomethyl(8)	0.77	0.5761		
	QSLETICLLLAYK+Carbamidomethyl(7)	18.85	0.0003		
	YPENFFLLR	0.76	0.3536		
Glyma14g07080.3	type one serine/threonine protein phosphatase 4	3	2.33	0.0039	protein
	Peptide Sequnce	Ratio 4(2)F+Ca	Pvalue 4(2)F+Ca		
	IYGFYDECK+Carbamidomethyl(8)	0.77	0.5761		
	QSLETICLLLAYK+Carbamidomethyl(7)	18.85	0.0003		
	YPENFFLLR	0.76	0.3536		
Glyma01g41920.2	Lactate/malate dehydrogenase family protein	3	2.33	0.0019	fermentation
	Peptide Sequnce	Ratio 4(2)F+Ca	Pvalue 4(2)F+Ca		
	GEMLDLQHAAAFLPR	2.47	0.0383		

	GYTSWAIGYSVANLAR	1.59	0.0563		
	VIGSGTNLDSSR	8.63	0.0130		
Glyma13g40890.1	histone H2A 12	2	2.33	0.0002	DNA
	Peptide Sequnce	Ratio 4(2)F+Ca	Pvalue 4(2)F+Ca		
	AGLQFPVGR	2.42	0.0006		
	LLAGVTIAHGGVLPNINPVLLPK	0.21	0.0342		
Glyma13g40900.1	histone H2A 12	2	2.33	0.0002	DNA
	Peptide Sequnce	Ratio 4(2)F+Ca	Pvalue 4(2)F+Ca		
	AGLQFPVGR	2.42	0.0006		
	LLAGVTIAHGGVLPNINPVLLPK	0.21	0.0342		
Glyma13g40940.1	histone H2A 12	2	2.33	0.0002	DNA
	Peptide Sequnce	Ratio 4(2)F+Ca	Pvalue 4(2)F+Ca		
	AGLQFPVGR	2.42	0.0006		
	LLAGVTIAHGGVLPNINPVLLPK	0.21	0.0342		
Glyma15g04530.1	histone H2A 12	2	2.33	0.0002	DNA
	Peptide Sequnce	Ratio 4(2)F+Ca	Pvalue 4(2)F+Ca		
	AGLQFPVGR	2.42	0.0006		
	LLAGVTIAHGGVLPNINPVLLPK	0.21	0.0342		
Glyma15g04540.1	histone H2A 12	2	2.33	0.0002	DNA
	Peptide Sequnce	Ratio 4(2)F+Ca	Pvalue 4(2)F+Ca		
	AGLQFPVGR	2.42	0.0006		
	LLAGVTIAHGGVLPNINPVLLPK	0.21	0.0342		
Glyma03g02852.1	N.D. *	2	2.39	0.0025	not assigned
	Peptide Sequnce	Ratio 4(2)F+Ca	Pvalue 4(2)F+Ca		
	ILLGYSAIK	3.12	0.0198		
	VFVSIGGDDPFK	2.03	0.0136		
Glyma01g34770.1	xyloglucan endotransglucosylase/hydrolase 26	2	2.42	0.0004	cell wall
	Peptide Sequnce	Ratio 4(2)F+Ca	Pvalue 4(2)F+Ca		
	NYENEGIAYPNK	0.28	0.0414		
	VYTSLWNADDWATR	2.54	0.0010		
Glyma09g32630.1	xyloglucan endotransglucosylase/hydrolase 26	2	2.42	0.0004	cell wall
	Peptide Sequnce	Ratio 4(2)F+Ca	Pvalue 4(2)F+Ca		
	NYENEGIAYPNK	0.28	0.0414		

	VYTSLWNADDWATR	2.54	0.0010		
Glyma03g34830.1	Enolase	19	2.46	0.0016	glycolysis
	Peptide Sequnce	Ratio 4(2)F+Ca	Pvalue 4(2)F+Ca		
	AAVPSGASTGIYEALELR	1.32	0.3532		
	ACNALLLK+Carbamidomethyl(2)	1.40	0.4865		
	AVDNVNTIIAPALVGK	1.08	0.2353		
	DGGSDYLGK	50.34	0.0891		
	EGLELLK	1.50	0.1728		
	GNPTVEVDLTCSDGTFAR+Carbamidomethyl(11)	1.08	0.8660		
	IEEELGAEAVYAGANFR	1.32	0.0419		
	KYGQDAVNVGDEGGFAPNIQENK	1.12	0.8152		
	LGANAILAVSLAVCK+Carbamidomethyl(14)	8.19	0.0273		
	LTAEVGTNVQIVGDDLLVTNPK	1.80	0.0693		
	MGVEVYHNLK	1.72	0.1793		
	TYDLNFK	1.87	0.0384		
	TYDLNFKEDNNDGSQK	0.17	0.1057		
	VNQIGSVTESIEAVR	1.59	0.0639		
	VVIGMDVAASEFYK	1.15	0.9655		
	VVIGMDVAASEFYKEDK	1.03	0.9660		
	YGQDAVNVGDEGGFAPNIQENK	0.85	0.1866		
	YGQDAVNVGDEGGFAPNIQENKEGLELLK	3.48	0.0362		
	YNQLLR	24.29	0.1031		
Glyma02g04800.1	Calcium dependent phosphotriesterase superfamily protein	2	2.58	0.0371	secondary metabolism
	Peptide Sequnce	Ratio 4(2)F+Ca	Pvalue 4(2)F+Ca		
	FFSYNPATK	0.94	0.8885		
	VVYTGCEDGWIK+Carbamidomethyl(6)	3.10	0.0068		
Glyma16g22650.1	Calcium dependent phosphotriesterase superfamily protein	2	2.58	0.0371	secondary metabolism
	Peptide Sequnce	Ratio 4(2)F+Ca	Pvalue 4(2)F+Ca		
	FFSYNPATK	0.94	0.8885		
	VVYTGCEDGWIK+Carbamidomethyl(6)	3.10	0.0068		
Glyma17g29320.1	Peroxidase family protein	2	2.63	0.0199	misc
	Peptide Sequnce	Ratio 4(2)F+Ca	Pvalue 4(2)F+Ca		
	LFFHDCFVR+Carbamidomethyl(6)	5.86	0.1201		

	VSCADILALATR+Carbamidomethyl(3)	2.19	0.0243		
Glyma09g06350.1	Peroxidase superfamily protein	5	2.65	0.0034	misc
	Peptide Sequnce	Ratio 4(2)F+Ca	Pvalue 4(2)F+Ca		
	FDNQYFK	12.31	0.1038		
	FQQTFVTAPATLR	3.76	0.0441		
	LFFHDCFVR+Carbamidomethyl(6)	5.86	0.1201		
	NTCPNVEQLVR+Carbamidomethyl(3)	1.40	0.1469		
	VSCADILALATR+Carbamidomethyl(3)	2.19	0.0243		
Glyma19g42760.1	gamma histone variant H2AX	3	2.72	0.0000	DNA
	Peptide Sequnce	Ratio 4(2)F+Ca	Pvalue 4(2)F+Ca		
	AGLQFPVGR	2.42	0.0006		
	HIQLAVR	6.94	0.0028		
	LLGSVTIANGGVLPNIHQTLLPK	0.25	0.0337		
Glyma20g28460.1	cupin family protein	2	2.72	0.0006	development
	Peptide Sequnce	Ratio 4(2)F+Ca	Pvalue 4(2)F+Ca		
	ESYFVDAQPQQK	3.93	0.0440		
	TISSEDEPFNLR	2.24	0.0013		
Glyma20g28640.1	cupin family protein	2	2.72	0.0006	development
	Peptide Sequnce	Ratio 4(2)F+Ca	Pvalue 4(2)F+Ca		
	ESYFVDAQPQQK	3.93	0.0440		
	TISSEDEPFNLR	2.24	0.0013		
Glyma04g38590.1	beta galactosidase 10	6	2.77	0.0011	misc
	Peptide Sequnce	Ratio 4(2)F+Ca	Pvalue 4(2)F+Ca		
	FASFGTPSGSCGSYLK+Carbamidomethyl(11)	2.75	0.0841		
	FTTYIVNLMK	0.67	0.2695		
	GLAWLNGEEIGR	0.44	0.0593		
	IGVQGEYLR	7.55	0.1855		
	IWTENWPGWFK	17.77	0.0010		
	SVPAMWPGLVQTAK	0.72	0.3348		
Glyma06g16420.2	beta galactosidase 10	5	2.78	0.0020	misc
	Peptide Sequnce	Ratio 4(2)F+Ca	Pvalue 4(2)F+Ca		
	FTTYIVNLMK	0.67	0.2695		
	GLAWLNGEEIGR	0.44	0.0593		

	IGVQGEYLR	7.55	0.1855		
	IWTENWPGWFK	17.77	0.0010		
	SVPAMWPGLVQTAK	0.72	0.3348		
Glyma02g00850.3	type one serine/threonine protein phosphatase 4	2	2.79	0.0012	protein
	Peptide Sequnce	Ratio 4(2)F+Ca	Pvalue 4(2)F+Ca		
	QSLETICLLLAYK+Carbamidomethyl(7)	18.85	0.0003		
	YPENFFLLR	0.76	0.3536		
Glyma13g05120.3	SPFH/Band 7/PHB domain containing membrane associated protein family	5	2.84	0.0002	not assigned
	Peptide Sequnce	Ratio 4(2)F+Ca	Pvalue 4(2)F+Ca		
	DSVLAFSENVPGTTAK	1.85	0.0023		
	EQIQSYVFDVIR	13.95	0.0366		
	LELDSVFEQK	4.67	0.0364		
	SSSVFIPHGPGAVK	7.29	0.2004		
	YLSGLGIAR	1.89	0.0539		
Glyma05g33410.2	Aldolasetype TIM barrel family protein	2	2.84	0.0077	OPP
	Peptide Sequnce	Ratio 4(2)F+Ca	Pvalue 4(2)F+Ca		
	AISSSNAYDDQLR	3.08	0.0046		
	DIESAYWELVVK	1.87	0.2089		
Glyma19g34780.1	RmlC like cupins superfamily protein	3	2.91	0.0287	development
	Peptide Sequnce	Ratio 4(2)F+Ca	Pvalue 4(2)F+Ca		
	ALVQVVNCNGER+Carbamidomethyl(8)	1.82	0.0450		
	LSAQFGSLR	0.72	0.4918		
	SQSDNFEYVSFK	16.77	0.0395		
Glyma19g32690.1	Ribosomal protein S10p/S20e family protein	4	2.95	0.0335	protein
	Peptide Sequnce	Ratio 4(2)F+Ca	Pvalue 4(2)F+Ca		
	SPCGEGTNTWDR+Carbamidomethyl(3)	0.90	0.8005		
	VCADLVR+Carbamidomethyl(2)	7.50	0.0713		
	VIDLYSSPDVVK	0.27	0.0169		
	VLLITTR	0.46	0.2469		
Glyma03g30440.1	Histone superfamily protein	2	2.99	0.0000	DNA
	Peptide Sequnce	Ratio 4(2)F+Ca	Pvalue 4(2)F+Ca		
	AGLQFPVGR	2.42	0.0006		
	HIQLAVR	6.94	0.0028		

Glyma12g34360.1	histone H2A 10	2	2.99	0.0000	DNA
	Peptide Sequnce	Ratio 4(2)F+Ca	Pvalue 4(2)F+Ca		
	AGLQFPVGR	2.42	0.0006		
	HIQLAVR	6.94	0.0028		
Glyma12g34370.2	histone H2A 2	2	2.99	0.0000	DNA
	Peptide Sequnce	Ratio 4(2)F+Ca	Pvalue 4(2)F+Ca		
	AGLQFPVGR	2.42	0.0006		
	HIQLAVR	6.94	0.0028		
Glyma13g36180.1	histone H2A 10	2	2.99	0.0000	DNA
	Peptide Sequnce	Ratio 4(2)F+Ca	Pvalue 4(2)F+Ca		
	AGLQFPVGR	2.42	0.0006		
	HIQLAVR	6.94	0.0028		
Glyma13g36190.1	histone H2A 10	2	2.99	0.0000	DNA
	Peptide Sequnce	Ratio 4(2)F+Ca	Pvalue 4(2)F+Ca		
	AGLQFPVGR	2.42	0.0006		
	HIQLAVR	6.94	0.0028		
Glyma19g33360.1	gamma histone variant H2AX	2	2.99	0.0000	DNA
	Peptide Sequnce	Ratio 4(2)F+Ca	Pvalue 4(2)F+Ca		
	AGLQFPVGR	2.42	0.0006		
	HIQLAVR	6.94	0.0028		
Glyma17g03360.1	N.D. *	7	3.03	0.0000	not assigned
	Peptide Sequnce	Ratio 4(2)F+Ca	Pvalue 4(2)F+Ca		
	ALVTDADNVIPK	1.73	0.0296		
	AVEAYLLANPHYN	3.00	0.0680		
	GDAQPNPDDLK	80.47	0.0158		
	GVFTFEDETTSPVAPATLYK	1.38	0.0254		
	IESVDEANLGYSYSVVGGVGLPDTVEK	20.95	0.0334		
	ITFVEDGESK	7.00	0.1998		
	SVENLEGNGGPGTIK	4.05	0.0206		
Glyma02g47210.1	HEAT SHOCK PROTEIN 81.4	16	3.11	0.0008	stress
	Peptide Sequnce	Ratio 4(2)F+Ca	Pvalue 4(2)F+Ca		
	ADLVNNLGTIAR	0.29	0.0788		

	APFDLFDTK	0.14	0.1622		
	AVENSPFLEK	1.03	0.9293		
	EDQLEYLEER	0.33	0.0950		
	EGQNDIYYITGESK	0.17	0.0198		
	ELISNASDALDK	41.39	0.0374		
	FEGLCHVIK+Carbamidomethyl(5)	0.96	0.9253		
	GIVDSEDLPLNISR	0.21	0.0774		
	HFSVEGQLEFK	0.20	0.1085		
	HSEFISYPISLWIEK	0.78	0.2681		
	LDAQPELFIHIIPDK	0.30	0.0869		
	SKLDAQPELFIHIIPDK	0.40	0.2797		
	SLTNDWEEHLAVK	0.79	0.1367		
	TMEINPENPIMDELRK	0.65	0.3627		
	TNNTLSIIDSGIGMTK	0.55	0.1401		
	VVDSPCCLVTGEYGWTANMER+Carbamidomethyl(6)Carbamidomethyl(7)	0.21	0.0673		
Glyma03g32380.2	Ribosomal protein L14p/L23e family protein	6	3.14	0.0025	protein
	Peptide Sequnce	Ratio 4(2)F+Ca	Pvalue 4(2)F+Ca		
	ECADLWPR+Carbamidomethyl(2)	0.34	0.0341		
	GSAITGPIGK	9.78	0.0933		
	LPSACVGDMVMATVK+Carbamidomethyl(5)	0.43	0.1925		
	MSLGLPVAATVNCADNTGAK+Carbamidomethyl(13)	0.41	0.1977		
	NLYIISVK	0.34	0.0465		
	VLPAVIVR	0.32	0.0454		
Glyma13g18830.1	Ribosomal protein L14p/L23e family protein	6	3.14	0.0025	protein
	Peptide Sequnce	Ratio 4(2)F+Ca	Pvalue 4(2)F+Ca		
	ECADLWPR+Carbamidomethyl(2)	0.34	0.0341		
	GSAITGPIGK	9.78	0.0933		
	LPSACVGDMVMATVK+Carbamidomethyl(5)	0.43	0.1925		
	MSLGLPVAATVNCADNTGAK+Carbamidomethyl(13)	0.41	0.1977		
	NLYIISVK	0.34	0.0465		
	VLPAVIVR	0.32	0.0454		
Glyma14g01530.1	HEAT SHOCK PROTEIN 81.4	17	3.25	0.0010	stress
	Peptide Sequnce	Ratio 4(2)F+Ca	Pvalue 4(2)F+Ca		

	ADLVNNLGTIAR	0.29	0.0788		
	APFDLFDTR	0.17	0.1521		
	AVENSPFLEK	1.03	0.9293		
	EDQLEYLEER	0.33	0.0950		
	EGQNDIYYITGESK	0.17	0.0198		
	ELISNASDALDK	41.39	0.0374		
	FEGLCHVIK+Carbamidomethyl(5)	0.96	0.9253		
	GIVDSEDLPLNISR	0.21	0.0774		
	HFSVEGQLEFK	0.20	0.1085		
	HSEFISYPISLWIEK	0.78	0.2681		
	LDAQPELFIHIIPDK	0.30	0.0869		
	RAPFDLFDTR	0.79	0.8178		
	SKLDAQPELFIHIIPDK	0.40	0.2797		
	SLTNDWEEHLAVK	0.79	0.1367		
	TMEINPENPIMEELR	0.28	0.1569		
	TNNTLSIIDSGIGMTK	0.55	0.1401		
	VVDSPCCLVTGEYGWTANMER+Carbamidomethyl(6)Carbamidomethyl(7)	0.21	0.0673		
Glyma14g04840.1	Ribosomal protein S10p/S20e family protein	3	3.34	0.0309	protein
	Peptide Sequnce	Ratio 4(2)F+Ca	Pvalue 4(2)F+Ca		
	SPCGEGTNTWDR+Carbamidomethyl(3)	0.90	0.8005		
	VCADLVR+Carbamidomethyl(2)	7.50	0.0713		
	VIDLYSSPDVVK	0.27	0.0169		
Glyma19g32680.1	Ribosomal protein S10p/S20e family protein	3	3.34	0.0309	protein
	Peptide Sequnce	Ratio 4(2)F+Ca	Pvalue 4(2)F+Ca		
	SPCGEGTNTWDR+Carbamidomethyl(3)	0.90	0.8005		
	VCADLVR+Carbamidomethyl(2)	7.50	0.0713		
	VIDLYSSPDVVK	0.27	0.0169		
Glyma02g00490.1	Ribosomal protein L7Ae/L30e/S12e/Gadd45 family protein	5	3.54	0.0369	protein
	Peptide Sequnce	Ratio 4(2)F+Ca	Pvalue 4(2)F+Ca		
	LIIIANNCPPLR+Carbamidomethyl(8)	0.33	0.0728		
	LIIIANNCPPLRK+Carbamidomethyl(8)	0.75	0.7672		
	SEIEYYAMLAK	0.60	0.4668		
	VCCLSIIDPGDSDIIK+Carbamidomethyl(2)Carbamidomethyl(3)	0.22	0.0296		

	VGVHHYNGNNVDLGTACGK+Carbamidomethyl(17)	6.31	0.0842		
Glyma08g28800.1	Ribosomal protein L7Ae/L30e/S12e/Gadd45 family protein	5	3.54	0.0369	protein
	Peptide Sequnce	Ratio 4(2)F+Ca	Pvalue 4(2)F+Ca		
	LIIIANNCPPLR+Carbamidomethyl(8)	0.33	0.0728		
	LIIIANNCPPLRK+Carbamidomethyl(8)	0.75	0.7672		
	SEIEYYAMLAK	0.60	0.4668		
	VCCLSIIDPGDSDIIK+Carbamidomethyl(2)Carbamidomethyl(3)	0.22	0.0296		
	VGVHHYNGNNVDLGTACGK+Carbamidomethyl(17)	6.31	0.0842		
Glyma18g51660.1	Ribosomal protein L7Ae/L30e/S12e/Gadd45 family protein	5	3.54	0.0369	protein
	Peptide Sequnce	Ratio 4(2)F+Ca	Pvalue 4(2)F+Ca		
	LIIIANNCPPLR+Carbamidomethyl(8)	0.33	0.0728		
	LIIIANNCPPLRK+Carbamidomethyl(8)	0.75	0.7672		
	SEIEYYAMLAK	0.60	0.4668		
	VCCLSIIDPGDSDIIK+Carbamidomethyl(2)Carbamidomethyl(3)	0.22	0.0296		
	VGVHHYNGNNVDLGTACGK+Carbamidomethyl(17)	6.31	0.0842		
Glyma03g39440.1	Calcineurin like metallophosphoesterase superfamily protein	2	3.55	0.0021	protein
	Peptide Sequnce	Ratio 4(2)F+Ca	Pvalue 4(2)F+Ca		
	QSLETICLLLAYK+Carbamidomethyl(7)	18.85	0.0003		
	QVQLSESEIR	0.88	0.6520		
Glyma19g42050.1	Calcineurin like metallophosphoesterase superfamily protein	2	3.55	0.0021	protein
	Peptide Sequnce	Ratio 4(2)F+Ca	Pvalue 4(2)F+Ca		
	QSLETICLLLAYK+Carbamidomethyl(7)	18.85	0.0003		
	QVQLSESEIR	0.88	0.6520		
Glyma17g03350.1	N.D. *	7	3.62	0.0000	not assigned
	Peptide Sequnce	Ratio 4(2)F+Ca	Pvalue 4(2)F+Ca		
	ALVTDADNVIPK	1.73	0.0296		
	AVEAYLLANPHYN	3.00	0.0680		
	GDAQPNPDDLK	80.47	0.0158		
	GIFTFEDETTSPVAPATLYK	3.71	0.0000		
	IESVDEANLGYSYSVVGGVGLPDTVEK	20.95	0.0334		
	ITFVEDGESK	7.00	0.1998		
	SVENLEGNGGPGTIK	4.05	0.0206		
Glyma10g28900.1	Adenine nucleotide alpha hydrolases like superfamily protein	4	3.67	0.0079	hormone metabolism
	Peptide Sequnce	Ratio 4(2)F+Ca	Pvalue 4(2)F+Ca		

	AFLGSVSNHCAQNVK+Carbamidomethyl(10)	0.97	0.2379		
	DVICQMVQK+Carbamidomethyl(4)	4.15	0.1403		
	LGADVLVMGSHGYGLIK	4.24	0.0214		
	YSQQVADCVLEK+Carbamidomethyl(8)	3.93	0.0440		
Glyma20g23090.1	Adenine nucleotide alpha hydrolases like superfamily protein	4	3.67	0.0079	hormone metabolism
	Peptide Sequnce	Ratio 4(2)F+Ca	Pvalue 4(2)F+Ca		
	AFLGSVSNHCAQNVK+Carbamidomethyl(10)	0.97	0.2379		
	DVICQMVQK+Carbamidomethyl(4)	4.15	0.1403		
	LGADVLVMGSHGYGLIK	4.24	0.0214		
	YSQQVADCVLEK+Carbamidomethyl(8)	3.93	0.0440		
Glyma08g44590.1	HEAT SHOCK PROTEIN 81.4	16	3.71	0.0014	stress
	Peptide Sequnce	Ratio 4(2)F+Ca	Pvalue 4(2)F+Ca		
	ADLVNNLGTIAR	0.29	0.0788		
	APFDLFDTR	0.17	0.1521		
	AVENSPFLEK	1.03	0.9293		
	EDQLEYLEER	0.33	0.0950		
	EGQSDIYYITGESK	0.21	0.0484		
	ELISNASDALDK	41.39	0.0374		
	GIVDSEDLPLNISR	0.21	0.0774		
	HFSVEGQLEFK	0.20	0.1085		
	HSEFISYPISLWIEK	0.78	0.2681		
	LDAQPELFIHIIPDK	0.30	0.0869		
	RAPFDLFDTR	0.79	0.8178		
	SKLDAQPELFIHIIPDK	0.40	0.2797		
	SLTNDWEEHLAVK	0.79	0.1367		
	TMEINPENPIMEELR	0.28	0.1569		
	TNNTLTIVDSGIGMTK	0.58	0.4259		
	VVDSPCCLVTGEYGWTANMER+Carbamidomethyl(6)Carbamidomethyl(7)	0.21	0.0673		
Glyma03g28870.1	beta-1,3-glucanase 1	3	3.87	0.0415	misc
	Peptide Sequnce	Ratio 4(2)F+Ca	Pvalue 4(2)F+Ca		
	DISLDYALFR	1.54	0.4757		
	SPSVVVQDGSLGYR	1.96	0.3495		
	TYNTNLVR	6.67	0.0086		

Glyma13g01140.1	Xyloglucan endotransglucosylase/hydrolase family protein	6	3.94	0.0005	cell wall
	Peptide Sequnce	Ratio 4(2)F+Ca	Pvalue 4(2)F+Ca		
	FAQGLPLECTHS+Carbamidomethyl(9)	1.31	0.6094		
	IVFSVDGTPIR	0.24	0.0305		
	IYSSLWNADDWATR	4.42	0.0002		
	LVPGNSAGTVTAYYLSSK	0.00	1.0000		
	NFNANACVWNR+Carbamidomethyl(7)	0.87	0.8600		
	NLESIGVPFPK	10.94	0.0089		
Glyma05g25540.1	FAD binding Berberine family protein	5	3.95	0.0002	misc
	Peptide Sequnce	Ratio 4(2)F+Ca	Pvalue 4(2)F+Ca		
	DLDIGTNSFGK	1.76	0.2969		
	ESMGEDLFWAIR	4.15	0.0476		
	MSEVSSDATPFPHR	16.76	0.0528		
	TEVDPENFFR	2.58	0.0094		
	TGFVFNPYGGK	3.36	0.0078		
Glyma08g08520.1	FAD binding Berberine family protein	7	3.95	0.0000	misc
	Peptide Sequnce	Ratio 4(2)F+Ca	Pvalue 4(2)F+Ca		
	DLDIGTNSFGK	1.76	0.2969		
	ESMGEDLFWAIR	4.15	0.0476		
	LVPVPEVVSVFR	24.89	0.0025		
	MSEVSSDATPFPHR	16.76	0.0528		
	NSYAEGAVYGVK	1.48	0.3975		
	TEVDPENFFR	2.58	0.0094		
	TGFVFNPYGGK	3.36	0.0078		
Glyma18g08220.1	HEAT SHOCK PROTEIN 81.4	16	4.02	0.0001	stress
	Peptide Sequnce	Ratio 4(2)F+Ca	Pvalue 4(2)F+Ca		
	ADLVNNLGTIAR	0.29	0.0788		
	APFDLFDTR	0.17	0.1521		
	AVENSPFLEK	1.03	0.9293		
	EDQLEYLEER	0.33	0.0950		
	EGQSDIYYITGESK	0.21	0.0484		
	ELISNASDALDK	41.39	0.0374		
	GIVDSEDLPLNISR	0.21	0.0774		
	HFSVEGQLEFK	0.20	0.1085		

	HSEFISYPISLWVEK	0.19	0.0497		
	LDAQPELFIHIIPDK	0.30	0.0869		
	RAPFDLFDTR	0.79	0.8178		
	SKLDAQPELFIHIIPDK	0.40	0.2797		
	SLTNDWEEHLAVK	0.79	0.1367		
	TMEINPENPIMEELR	0.28	0.1569		
	TNNSLTIVDSGIGMTK	0.30	0.0299		
	VVDSPCCLVTGEYGWTANMER+Carbamidomethyl(6)Carbamidomethyl(7)	0.21	0.0673		
Glyma08g11300.2	xyloglucan endotransglucosylase/hydrolase 16	3	4.05	0.0003	cell wall
	Peptide Sequnce	Ratio 4(2)F+Ca	Pvalue 4(2)F+Ca		
	APFTAYYR	2.95	0.0441		
	EQQFYLWFDPTR	2.48	0.2982		
	IYSSLWNADDWATR	4.42	0.0002		
Glyma11g36730.2	xyloglucan endotransglucosylase/hydrolase 16	3	4.05	0.0003	cell wall
	Peptide Sequnce	Ratio 4(2)F+Ca	Pvalue 4(2)F+Ca		
	APFTAYYR	2.95	0.0441		
	EQQFYLWFDPTR	2.48	0.2982		
	IYSSLWNADDWATR	4.42	0.0002		
Glyma18g00630.2	xyloglucan endotransglucosylase/hydrolase 16	3	4.05	0.0003	cell wall
	Peptide Sequnce	Ratio 4(2)F+Ca	Pvalue 4(2)F+Ca		
	APFTAYYR	2.95	0.0441		
	EQQFYLWFDPTR	2.48	0.2982		
	IYSSLWNADDWATR	4.42	0.0002		
Glyma17g07250.1	xyloglucan endotransglycosylase 6	5	4.09	0.0001	cell wall
	Peptide Sequnce	Ratio 4(2)F+Ca	Pvalue 4(2)F+Ca		
	ILNNENLLTLSLDK	4.84	0.3400		
	IVFSVDGTPIR	0.24	0.0305		
	IYSSLWNADDWATR	4.42	0.0002		
	LVPGNSAGTVTAYYLSSK	0.00	1.0000		
	NLESIGVPFPK	10.94	0.0089		
Glyma02g19380.1	copper chaperone	4	4.12	0.0331	metal handling
	Peptide Sequnce	Ratio 4(2)F+Ca	Pvalue 4(2)F+Ca		
	GNVEPDEVLQAVSK	2.08	0.1701		

	MEGVESFDIDLK	2.32	0.1566		
	TAFWVDEAPQSK	1.72	0.3528		
	VGMSCQGCAGAVNR+Carbamidomethyl(5)Carbamidomethyl(8)	12.92	0.0248		
Glyma15g17620.1	Peroxidase superfamily protein	5	4.14	0.0012	misc
	Peptide Sequnce	Ratio 4(2)F+Ca	Pvalue 4(2)F+Ca		
	FDNQYFK	12.31	0.1038		
	FQQTFVTAPATLR	3.76	0.0441		
	IAINMDPVTPQK	7.72	0.0364		
	LFFHDCFVR+Carbamidomethyl(6)	5.86	0.1201		
	VSCADILALATR+Carbamidomethyl(3)	2.19	0.0243		
Glyma13g01120.1	xyloglucan endotransglycosylase 6	3	4.14	0.0027	cell wall
	Peptide Sequnce	Ratio 4(2)F+Ca	Pvalue 4(2)F+Ca		
	IVFSVDGSPIR	2.85	0.2109		
	IYSSLWNADDWATR	4.42	0.0002		
	LVPGNSAGTVTAYYLSSK	0.00	1.0000		
Glyma17g07240.1	xyloglucan endotransglycosylase 6	3	4.14	0.0027	cell wall
	Peptide Sequnce	Ratio 4(2)F+Ca	Pvalue 4(2)F+Ca		
	IVFSVDGSPIR	2.85	0.2109		
	IYSSLWNADDWATR	4.42	0.0002		
	LVPGNSAGTVTAYYLSSK	0.00	1.0000		
Glyma05g28310.2	xyloglucan endotransglucosylase/hydrolase 16	4	4.18	0.0001	cell wall
	Peptide Sequnce	Ratio 4(2)F+Ca	Pvalue 4(2)F+Ca		
	APFTAYYR	2.95	0.0441		
	EQQFYLWFDPTR	2.48	0.2982		
	IYSSLWNADDWATR	4.42	0.0002		
	NAESLGVPFPK	7.26	0.0514		
Glyma13g01150.1	xyloglucan endotransglucosylase/hydrolase family protein	3	4.18	0.0045	cell wall
	Peptide Sequnce	Ratio 4(2)F+Ca	Pvalue 4(2)F+Ca		
	IYSSLWNADDWATR	4.42	0.0002		
	LVPGNSAGTVTAYYLSSK	0.00	1.0000		
	NYMIYNYCTDTK+Carbamidomethyl(8)	0.07	0.3869		
Glyma17g07270.1	xyloglucan endotransglycosylase 6	3	4.18	0.0045	cell wall
	Peptide Sequnce	Ratio 4(2)F+Ca	Pvalue 4(2)F+Ca		

	IYSSLWNADDWATR	4.42	0.0002		
	LVPGNSAGTVTAYYLSSK	0.00	1.0000		
	NYMIYNYCTDTK+Carbamidomethyl(8)	0.07	0.3869		
Glyma01g04350.1	Matrixin family protein	3	4.52	0.0067	protein
	Peptide Sequnce	Ratio 4(2)F+Ca	Pvalue 4(2)F+Ca		
	GVILLDGTNK	3.65	0.0379		
	LWALPSENGR	1.42	0.1925		
	TYQQYFSLQPTGK	14.86	0.0184		
Glyma19g02370.4	SPFH/Band 7/PHB domain containing membrane associated protein family	4	4.57	0.0044	not assigned
	Peptide Sequnce	Ratio 4(2)F+Ca	Pvalue 4(2)F+Ca		
	EQIQSYVFDVIR	13.95	0.0366		
	LELDSVFEQK	4.67	0.0364		
	SSSVFIPHGPGAVK	7.29	0.2004		
	YLSGLGIAR	1.89	0.0539		
Glyma13g00790.1	Peroxidase superfamily protein	9	4.60	0.0003	misc
	Peptide Sequnce	Ratio 4(2)F+Ca	Pvalue 4(2)F+Ca		
	DVVNLAGGPFYNVELGR	128.00	0.0412		
	FDNQYFK	12.31	0.1038		
	FQQTFVTAPATLR	3.76	0.0441		
	GLFTSDQVLFTDAR	2.49	0.0866		
	IAINMDPVTPQK	7.72	0.0364		
	IDPTLNLQYAFQLR	1.87	0.4169		
	LFFHDCFVR+Carbamidomethyl(6)	5.86	0.1201		
	NTCPNVEQLVR+Carbamidomethyl(3)	1.40	0.1469		
	VSCADILALATR+Carbamidomethyl(3)	2.19	0.0243		
Glyma17g06890.1	Peroxidase superfamily protein	9	4.60	0.0003	misc
	Peptide Sequnce	Ratio 4(2)F+Ca	Pvalue 4(2)F+Ca		
	DVVNLAGGPFYNVELGR	128.00	0.0412		
	FDNQYFK	12.31	0.1038		
	FQQTFVTAPATLR	3.76	0.0441		
	GLFTSDQVLFTDAR	2.49	0.0866		
	IAINMDPVTPQK	7.72	0.0364		
	IDPTLNLQYAFQLR	1.87	0.4169		
	LFFHDCFVR+Carbamidomethyl(6)	5.86	0.1201		

	NTCPNVEQLVR+Carbamidomethyl(3)	1.40	0.1469		
	VSCADILALATR+Carbamidomethyl(3)	2.19	0.0243		
Glyma19g31580.1	beta-1,3-glucanase 1	2	4.82	0.0205	misc
	Peptide Sequnce	Ratio 4(2)F+Ca	Pvalue 4(2)F+Ca		
	SPSVVVQDGSLGYR	1.96	0.3495		
	TYNTNLVR	6.67	0.0086		
Glyma10g14110.1	copper chaperone	3	5.00	0.0369	metal handling
	Peptide Sequnce	Ratio 4(2)F+Ca	Pvalue 4(2)F+Ca		
	GNVQPDEVLQAVSK	1.500	0.3138		
	MEGVESFDIDLK	2.321	0.1566		
	VGMSCQGCAGAVNR+Carbamidomethyl(5)Carbamidomethyl(8)	12.923	0.0248		
Glyma07g11810.1	glutamine synthase clone R1	7	5.10	0.0022	N-metabolism
	Peptide Sequnce	Ratio 4(2)F+Ca	Pvalue 4(2)F+Ca		
	DIVDAHYK	6.06	0.0889		
	EHIAAYGEGNER	63.29	0.0000		
	GNNILVICDVYTPAGEPLPTNK+Carbamidomethyl(8)	1.11	0.9130		
	HETADINTFSWGVANR	2.00	0.2487		
	IIAEYIWVGGSGMDLR	1.24	0.3600		
	RGNNILVICDVYTPAGEPLPTNK+Carbamidomethyl(9)	0.89	0.9520		
	TLPGPVSDPAK	6.88	0.4356		
Glyma09g04337.1	beta xylosidase 1	3	5.11	0.0000	cell wall
	Peptide Sequnce	Ratio 4(2)F+Ca	Pvalue 4(2)F+Ca		
	AGLDLDCGPFLAIHTDSAIR+Carbamidomethyl(7)	0.41	0.0122		
	LGIQGYEWWSEALHGVSNVGPGTK	27.96	0.0000		
	LVVNNAIAVPR	0.44	0.0263		
Glyma15g15370.1	beta xylosidase 1	3	5.11	0.0000	cell wall
	Peptide Sequnce	Ratio 4(2)F+Ca	Pvalue 4(2)F+Ca		
	AGLDLDCGPFLAIHTDSAIR+Carbamidomethyl(7)	0.41	0.0122		
	LGIQGYEWWSEALHGVSNVGPGTK	27.96	0.0000		
	LVVNNAIAVPR	0.44	0.0263		
Glyma13g34540.1	D mannose binding lectin protein	3	5.24	0.0291	misc
	Peptide Sequnce	Ratio 4(2)F+Ca	Pvalue 4(2)F+Ca		
	ENVDGPYSLVLEPK	4.62	0.0349		
	LGIDGNIR	1.05	0.9554		

	WVWEANR	6.73	0.0267		
Glyma19g39270.2	Peroxidase superfamily protein	4	5.27	0.0186	misc
	Peptide Sequnce	Ratio 4(2)F+Ca	Pvalue 4(2)F+Ca		
	GDQDPSLNPTYANFLK	10.78	0.0171		
	GLFQSDAALLTTK	6.62	0.0303		
	MHFHDCFVR+Carbamidomethyl(6)	1.64	0.4737		
	MHFHDCFVR+Carbamidomethyl(6)Oxidation(1)	2.29	0.4158		
Glyma03g32030.1	RmlC like cupins superfamily protein	5	5.35	0.0160	development
	Peptide Sequnce	Ratio 4(2)F+Ca	Pvalue 4(2)F+Ca		
	FYLAGNQEQEFLK	9.07	0.0705		
	LSAEFGSLR	2.06	0.5206		
	SQSDNFEYVSFK	16.77	0.0395		
	VFDGELQEGR	2.92	0.2108		
	VLIVPQNFVVAAR	7.32	0.0596		
Glyma02g03320.1	Matrixin family protein	4	5.35	0.0070	protein
	Peptide Sequnce	Ratio 4(2)F+Ca	Pvalue 4(2)F+Ca		
	FSNLPVTGVPNK	4.861448288	0.1311		
	LFGPALAK	4.983986075	0.1085		
	MEVYGGSLIFLQPDSSK	4.047945732	0.0169		
	QLIQQMLSLR	6.320277037	0.1113		
Glyma01g34410.2	AThook motif nuclearlocalized protein 1	3	5.69	0.0421	RNA
	Peptide Sequnce	Ratio 4(2)F+Ca	Pvalue 4(2)F+Ca		
	FEILSLSGSFMPTDNQGTR	6.44	0.0073		
	TGGMSVSLASPDGR	1.81	0.2816		
	VISFSQQGPR	1.30	0.7062		
Glyma03g02670.5	AThook motif nuclearlocalized protein 1	3	5.69	0.0421	RNA
	Peptide Sequnce	Ratio 4(2)F+Ca	Pvalue 4(2)F+Ca		
	FEILSLSGSFMPTDNQGTR	6.44	0.0073		
	TGGMSVSLASPDGR	1.81	0.2816		
	VISFSQQGPR	1.30	0.7062		
Glyma15g08840.1	xylem bark cysteine peptidase 3	2	5.80	0.0149	protein
	Peptide Sequnce	Ratio 4(2)F+Ca	Pvalue 4(2)F+Ca		
	ALDWVIGNR	4.83	0.0190		
	GIASEIDYPYTAR	26.68	0.1094		

Glyma07g02720.1	Ribosomal protein L7Ae/L30e/S12e/Gadd45 family protein	7	6.05	0.0034	protein
	Peptide Sequnce	Ratio 4(2)F+Ca	Pvalue 4(2)F+Ca		
	ALCAEHNVSLLTVPSAK+Carbamidomethyl(3)	0.96	0.2507		
	DFGEEHEAYNVVLQHVK	1.13	0.3050		
	HAAQLCVLAEDCDQPDYVK+Carbamidomethyl(6)Carbamidomethyl(12)	0.69	0.2906		
	KVTGCSCVVVK+Carbamidomethyl(5)Carbamidomethyl(7)	5.05	0.0657		
	SLAYGGLSR	2.05	0.4976		
	TLGEWAGLCK+Carbamidomethyl(9)	0.69	0.1608		
	VTGCSCVVVK+Carbamidomethyl(4)Carbamidomethyl(6)	151.23	0.0008		
Glyma08g23260.3	Ribosomal protein L7Ae/L30e/S12e/Gadd45 family protein	7	6.06	0.0033	protein
	Peptide Sequnce	Ratio 4(2)F+Ca	Pvalue 4(2)F+Ca		
	ALCAEHNVSLLTVPSAK+Carbamidomethyl(3)	0.96	0.2507		
	DFGEEHEAYNVVLQHVK	1.13	0.3050		
	HAAQLCVLAEDCDQPDYVK+Carbamidomethyl(6)Carbamidomethyl(12)	0.69	0.2906		
	KVTGCSCVVVK+Carbamidomethyl(5)Carbamidomethyl(7)	5.05	0.0657		
	SLAYGGLAR	2.96	0.4746		
	TLGEWAGLCK+Carbamidomethyl(9)	0.69	0.1608		
	VTGCSCVVVK+Carbamidomethyl(4)Carbamidomethyl(6)	151.23	0.0008		
Glyma13g44690.1	Ribosomal protein L7Ae/L30e/S12e/Gadd45 family protein	7	6.06	0.0033	protein
	Peptide Sequnce	Ratio 4(2)F+Ca	Pvalue 4(2)F+Ca		
	ALCAEHNVSLLTVPSAK+Carbamidomethyl(3)	0.96	0.2507		
	DFGEEHEAYNVVLQHVK	1.13	0.3050		
	HAAQLCVLAEDCDQPDYVK+Carbamidomethyl(6)Carbamidomethyl(12)	0.69	0.2906		
	KVTGCSCVVVK+Carbamidomethyl(5)Carbamidomethyl(7)	5.05	0.0657		
	SLAYGGLAR	2.96	0.4746		
	TLGEWAGLCK+Carbamidomethyl(9)	0.69	0.1608		
	VTGCSCVVVK+Carbamidomethyl(4)Carbamidomethyl(6)	151.23	0.0008		
Glyma15g00610.1	Ribosomal protein L7Ae/L30e/S12e/Gadd45 family protein	7	6.06	0.0033	protein
	Peptide Sequnce	Ratio 4(2)F+Ca	Pvalue 4(2)F+Ca		
	ALCAEHNVSLLTVPSAK+Carbamidomethyl(3)	0.96	0.2507		
	DFGEEHEAYNVVLQHVK	1.13	0.3050		
	HAAQLCVLAEDCDQPDYVK+Carbamidomethyl(6)Carbamidomethyl(12)	0.69	0.2906		
	KVTGCSCVVVK+Carbamidomethyl(5)Carbamidomethyl(7)	5.05	0.0657		

	SLAYGGLAR	2.96	0.4746		
	TLGEWAGLCK+Carbamidomethyl(9)	0.69	0.1608		
	VTGCSCVVVK+Carbamidomethyl(4)Carbamidomethyl(6)	151.23	0.0008		
Glyma09g40520.5	AThook motif nuclearlocalized protein 1	2	6.08	0.0324	RNA
	Peptide Sequnce	Ratio 4(2)F+Ca	Pvalue 4(2)F+Ca		
	FEILSLSGSFMPTDNQGTR	6.44	0.0073		
	VISFSQQGPR	1.30	0.7062		
Glyma13g32300.1	Quinone reductase family protein	6	6.14	0.0133	lipid metabolism
	Peptide Sequnce	Ratio 4(2)F+Ca	Pvalue 4(2)F+Ca		
	AFFDATGGLWR	1.40	0.3714		
	FGSMAAQFK	1.70	0.4394		
	GGSPYGAGTYAGDGSR	22.83	0.0373		
	LWQVPETLPEEVLAK	1.45	0.1471		
	QPSELELAQAFHQGK	1.36	0.2536		
	VYIVYYSTYGHVEK	3.07	0.0139		
Glyma15g07040.1	Quinone reductase family protein	6	6.14	0.0133	lipid metabolism
	Peptide Sequnce	Ratio 4(2)F+Ca	Pvalue 4(2)F+Ca		
	AFFDATGGLWR	1.40	0.3714		
	FGSMAAQFK	1.70	0.4394		
	GGSPYGAGTYAGDGSR	22.83	0.0373		
	LWQVPETLPEEVLAK	1.45	0.1471		
	QPSELELAQAFHQGK	1.36	0.2536		
	VYIVYYSTYGHVEK	3.07	0.0139		
Glyma18g43460.1	pyruvate decarboxylase2	11	6.28	0.0000	fermentation
	Peptide Sequnce	Ratio 4(2)F+Ca	Pvalue 4(2)F+Ca		
	AIIVQPDR	7.61	0.0831		
	DSLCFIEVIVHK+Carbamidomethyl(4)	3.95	0.0332		
	DSLCFIEVIVHKDDTSK+Carbamidomethyl(4)	0.35	0.0201		
	ELLEWGSR	6.10	0.0001		
	ESKPVYISISCNLPGIPHPTFSR+Carbamidomethyl(11)	7.10	0.0035		
	IFVPDGHPLK	1.87	0.1916		
	ILHHTIGLPDFSQELR	9.47	0.0214		
	KDSLCFIEVIVHK+Carbamidomethyl(5)	5.49	0.1119		

	NWNYTGLIDAIHNGEGK	9.53	0.0203		
	VSAANSRPPNPQ	15.33	0.1288		
	VVIANGPAFGCVLMK+Carbamidomethyl(11)	3.97	0.2441		
Glyma09g28100.1	Enolase	17	6.30	0.0000	glycolysis
	Peptide Sequnce	Ratio 4(2)F+Ca	Pvalue 4(2)F+Ca		
	AAVPSGASTGVYEALELR	5.53	0.0021		
	ACNALLLK+Carbamidomethyl(2)	1.40	0.4865		
	AVENVNSIIAPALLGK	7.90	0.0005		
	DGGSDYLGK	50.34	0.0891		
	GNPTVEVDVILSDGSFHR	10.39	0.0001		
	IEEELGSAAVYAGAK	4.27	0.1500		
	LGANAILAVSLAVCK+Carbamidomethyl(14)	8.19	0.0273		
	LGANAILAVSLAVCKAGAAVK+Carbamidomethyl(14)	0.84	0.7668		
	LTAEVGQQVQIVGDDLLVTNPK	15.65	0.0068		
	MGVEVYHHLK	13.88	0.1093		
	TLVLPVPSFNVINGGSHAGNK	5.42	0.1478		
	TYDLNFK	1.87	0.0384		
	TYDLNFKEENNDGSQK	9.17	0.2207		
	VNQIGSVTESIEAVR	1.59	0.0639		
	VVIGMDVAASEFYDNK	17.61	0.0753		
	YGQDATNVGDEGGFAPNIQENQEGLELLK	11.54	0.0181		
	YNQLLR	24.29	0.1031		
Glyma08g45510.1	Kunitz family trypsin and protease inhibitor protein	3	6.65	0.0161	stress
	Peptide Sequnce	Ratio 4(2)F+Ca	Pvalue 4(2)F+Ca		
	GLPEGLAVK	2.60	0.3777		
	NGGTYYILPVIR	4.14	0.0244		
	VSLEFNDYK	18.57	0.0445		
Glyma13g34520.1	D mannose binding lectin protein	6	6.98	0.0000	misc
	Peptide Sequnce	Ratio 4(2)F+Ca	Pvalue 4(2)F+Ca		
	ENVDGPYSLVLEPK	4.62	0.0349		
	FENSGELGPYIVEYGADYR	9.68	0.0004		
	FYTYFLDVR	5.13	0.0778		
	GLALYYK	7.34	0.0334		
	LGIDGNIR	1.05	0.9554		
	WVWEANR	6.73	0.0267		

Glyma15g15200.1	Glycosyl hydrolase superfamily protein	4	7.25	0.0039	misc
	Peptide Sequnce	Ratio 4(2)F+Ca	Pvalue 4(2)F+Ca		
	HFGLFNPNK	4.14	0.1096		
	LYDPNQAALEALR	2.59	0.0347		
	NVLNFWPSVK	20.53	0.0533		
	VYLDNLVR	23.83	0.0615		
Glyma04g39930.1	manganese superoxide dismutase 1	4	7.27	0.0490	redox
	Peptide Sequnce	Ratio 4(2)F+Ca	Pvalue 4(2)F+Ca		
	ALEQLQDAVAK	0.58	0.1597		
	HHQTYITNFNK	16.34	0.0164		
	LVVETTANQDPLVTK	0.64	0.4612		
	VNAEGAALQGSGWVWLGLDK	2.22	0.3449		
Glyma09g30370.1	glutamine synthase clone R1	8	7.86	0.0038	N-metabolism
	Peptide Sequnce	Ratio 4(2)F+Ca	Pvalue 4(2)F+Ca		
	DIVDAHYK	6.06	0.0889		
	EHIAAYGEGNER	63.29	0.0000		
	GNNILVICDVYTPAGEPLPTNK+Carbamidomethyl(8)	1.11	0.9130		
	HETADINTFSWGVANR	2.00	0.2487		
	IVAEYIWVGGSGMDLR	1.37	0.3026		
	RGNNILVICDVYTPAGEPLPTNK+Carbamidomethyl(9)	0.89	0.9520		
	SMRDEGGYEVIK	1.39	0.6226		
	TLPGPVSDPAK	6.88	0.4356		
Glyma03g36610.1	Peroxidase superfamily protein	3	8.03	0.0015	misc
	Peptide Sequnce	Ratio 4(2)F+Ca	Pvalue 4(2)F+Ca		
	GDQDPSLNPTYANFLK	10.78	0.0171		
	GLFQSDAALLTTK	6.62	0.0303		
	MGAIEVLTGSAGEIR	8.86	0.0412		
Glyma07g18570.1	Thiamine pyrophosphate dependent pyruvate decarboxylase family protein	13	8.10	0.0000	fermentation
	Peptide Sequnce	Ratio 4(2)F+Ca	Pvalue 4(2)F+Ca		
	AIIVQPDR	7.61	0.0831		
	DPVPFSLSPR	11.82	0.0427		
	DSLCFIEVIVHK+Carbamidomethyl(4)	3.95	0.0332		
	DSLCFIEVIVHKDDTSK+Carbamidomethyl(4)	0.35	0.0201		
	ELLEWGSR	6.10	0.0001		

	ESKPVYISISCNLPGIPHPTFSR+Carbamidomethyl(11)	7.10	0.0035		
	IFVPEGHPLK	145.48	0.0102		
	ILHHTIGLPDFSQELR	9.47	0.0214		
	MLSGETAVIAETGDSWFNCQK+Carbamidomethyl(19)	9.36	0.0097		
	NWNYTGLIDAIHNGEGK	9.53	0.0203		
	VNVLFQHIQK	5.83	0.0330		
	VSAANSRPPNPQ	15.33	0.1288		
	VVIANGPAFGCVLMK+Carbamidomethyl(11)	3.97	0.2441		
Glyma09g24410.1	heat shock protein 90.1	7	8.46	0.0106	stress
	Peptide Sequnce	Ratio 4(2)F+Ca	Pvalue 4(2)F+Ca		
	ADLVNNLGTIAR	0.29	0.0788		
	APFDLFDTR	0.17	0.1521		
	EDQLEYLEER	0.33	0.0950		
	ELISNASDALDK	41.39	0.0374		
	HFSVEGQLEFK	0.20	0.1085		
	RAPFDLFDTR	0.79	0.8178		
	SLTNDWEEHLAVK	0.79	0.1367		
Glyma16g29750.1	heat shock protein 90.1	7	8.46	0.0106	stress
	Peptide Sequnce	Ratio 4(2)F+Ca	Pvalue 4(2)F+Ca		
	ADLVNNLGTIAR	0.29	0.0788		
	APFDLFDTR	0.17	0.1521		
	EDQLEYLEER	0.33	0.0950		
	ELISNASDALDK	41.39	0.0374		
	HFSVEGQLEFK	0.20	0.1085		
	RAPFDLFDTR	0.79	0.8178		
	SLTNDWEEHLAVK	0.79	0.1367		
Glyma08g08360.1	polygalacturonase inhibiting protein 1	6	9.61	0.0000	cell wall
	Peptide Sequnce	Ratio 4(2)F+Ca	Pvalue 4(2)F+Ca		
	CLCGSPLPK+Carbamidomethyl(1)Carbamidomethyl(3)	6.49	0.0920		
	EALLQIK	19.66	0.2709		
	ISGTIPDSFGSFSDSLK	10.20	0.0000		
	LTGEIPATLAK	8.61	0.0020		
	LYGALPEGLTSLK	45.12	0.0288		
	TLLILYLSHNR	3.89	0.0235		
Glyma10g03091.1	Eukaryotic aspartyl protease family protein	2	9.67	0.0000	protein

	Peptide Sequnce	Ratio 4(2)F+Ca	Pvalue 4(2)F+Ca		
	LGAAVPTIELVLQNQK	9.22	0.0001		
	LGFSSLLYGSR	10.13	0.0074		
Glyma08g03310.1	ACC oxidase 1	3	9.78	0.0008	hormone metabolism
	Peptide Sequnce	Ratio 4(2)F+Ca	Pvalue 4(2)F+Ca		
	LLYPSNFR	7.55	0.0397		
	LSELMSENLGLEK	5.96	0.0613		
	YPQCPRPELVR+Carbamidomethyl(4)	12.21	0.0042		
Glyma15g07940.1	Rubber elongation factor protein (REF)	4	10.10	0.0093	not assigned
	Peptide Sequnce	Ratio 4(2)F+Ca	Pvalue 4(2)F+Ca		
	VAELDSHVPSNVK	10.96	0.1594		
	VSAYLPLVPTEK	18.82	0.0602		
	VSSQACSVVSEVR+Carbamidomethyl(6)	1.77	0.1701		
	YEPTAEQCAVSAWR+Carbamidomethyl(8)	21.32	0.0240		
Glyma02g16710.1	Eukaryotic aspartyl protease family protein	7	10.91	0.0000	protein
	Peptide Sequnce	Ratio 4(2)F+Ca	Pvalue 4(2)F+Ca		
	DASTLQYITQIK	16.65	0.0031		
	IALPSQLASAFSFR	1.19	0.8087		
	IFGANSMVSVSDDK	6.06	0.0772		
	LGAAVPTIELVLQNQK	9.22	0.0001		
	LGFSSLLYGSR	10.13	0.0074		
	VASVAPFEVCFSR+Carbamidomethyl(10)	1.28	0.4804		
	VLCLGFVNGGENPR+Carbamidomethyl(3)	4.84	0.0813		
Glyma03g22260.1	Auxin responsive family protein	2	11.51	0.0081	hormone metabolism
	Peptide Sequnce	Ratio 4(2)F+Ca	Pvalue 4(2)F+Ca		
	GAEAFLAFPSSASASAITLGR	23.88	0.0015		
	SLSVAFSAEPPSR	0.94	0.6911		
Glyma05g36310.1	ACC oxidase 1	6	11.72	0.0000	hormone metabolism
	Peptide Sequnce	Ratio 4(2)F+Ca	Pvalue 4(2)F+Ca		
	AFSGNGEGPAVGTK	9.88	0.1301		
	LLYPSNFR	7.55	0.0397		
	LSELMSENLGLEK	5.96	0.0613		
	QLINAYYEENLK	34.59	0.0125		
	WGCFMVENHEIDTQLMGK+Carbamidomethyl(3)	54.82	0.0330		
	YPQCPRPELVR+Carbamidomethyl(4)	12.21	0.0042		

Glyma16g33760.1	Kunitz family trypsin and protease inhibitor protein	3	12.27	0.0240	stress
	Peptide Sequnce	Ratio 4(2)F+Ca	Pvalue 4(2)F+Ca		
	LALSSEPYR	24.53	0.0632		
	LVYCPSVCK+Carbamidomethyl(4)Carbamidomethyl(8)	0.60	0.4222		
	VSTDLNIYFPIDTSCPLTK+Carbamidomethyl(15)	7.62	0.0259		
Glyma12g23150.1	Aluminium induced protein with YGL and LRDR motifs	6	14.12	0.0006	hormone metabolism
	Peptide Sequnce	Ratio 4(2)F+Ca	Pvalue 4(2)F+Ca		
	DLDGSFGFVVYDSK	50.73	0.0102		
	DRGPYPADQVVK	2.59	0.4946		
	GTNEAMFVIEAYK	35.52	0.1282		
	IDSEGAICGANFK+Carbamidomethyl(8)	8.53	0.0407		
	VGSQANWMEWTQH	112.25	0.0851		
	VGSVFAALGSDGGIK	7.10	0.0175		
Glyma08g06570.1	flavodoxinlike quinone reductase 1	2	14.54	0.0044	lipid metabolism
	Peptide Sequnce	Ratio 4(2)F+Ca	Pvalue 4(2)F+Ca		
	GGSPYGAGTYAGDGSR	22.83	0.0373		
	VYIVYYSTYGHVEK	3.07	0.0139		
Glyma04g37140.1	SNF1related protein kinase regulatory subunit gamma 1	2	14.59	0.0493	cell wall
	Peptide Sequnce	Ratio 4(2)F+Ca	Pvalue 4(2)F+Ca		
	VYEDEPVLQAFK	11.85	0.1120		
	WAPFLALER	17.56	0.0764		
Glyma04g09670.1	Rhamnogalacturonate lyase family protein	6	15.56	0.0002	cell wall
	Peptide Sequnce	Ratio 4(2)F+Ca	Pvalue 4(2)F+Ca		
	DGPTLWEIGIPDR	39.48	0.0242		
	DWFFAQVTR	14.14	0.0277		
	GAYVGLAPPGDVGSWQR	5.70	0.0148		
	GNGPFQAIMYDYIR	62.07	0.2211		
	SAAEFYVPDPNPK	13.51	0.0101		
	YINDDYVSAK	3.14	0.4400		
Glyma09g04191.1	Glycosyl hydrolase superfamily protein	2	17.27	0.0359	misc
	Peptide Sequnce	Ratio 4(2)F+Ca	Pvalue 4(2)F+Ca		
	HFGLFNPNK	4.14	0.1096		
	NVLNFWPSVK	20.53	0.0533		
Glyma11g35600.1	Eukaryotic translation initiation factor 2 subunit 1	5	17.46	0.0029	protein
	Peptide Sequnce	Ratio 4(2)F+Ca	Pvalue 4(2)F+Ca		

	CFQFDGVLHIK+Carbamidomethyl(1)	0.30	0.0422		
	EQGILVLNNAIASCTEAIEQHK+Carbamidomethyl(14)	0.28	0.0947		
	IEPVMVLR	0.44	0.2503		
	IIVTDPDTVLSTLTR	0.38	0.0900		
	VSEEDIQACEER+Carbamidomethyl(9)	40.91	0.0176		
Glyma18g02820.1	Eukaryotic translation initiation factor 2 subunit 1	5	17.46	0.0029	protein
	Peptide Sequnce	Ratio 4(2)F+Ca	Pvalue 4(2)F+Ca		
	CFQFDGVLHIK+Carbamidomethyl(1)	0.30	0.0422		
	EQGILVLNNAIASCTEAIEQHK+Carbamidomethyl(14)	0.28	0.0947		
	IEPVMVLR	0.44	0.2503		
	IIVTDPDTVLSTLTR	0.38	0.0900		
	VSEEDIQACEER+Carbamidomethyl(9)	40.91	0.0176		
Glyma09g16690.1	Chaperone protein htpG family protein	4	18.18	0.0071	stress
	Peptide Sequnce	Ratio 4(2)F+Ca	Pvalue 4(2)F+Ca		
	ELISNASDALDK	41.39	0.0374		
	FEFQAEVSR	0.15	0.0782		
	FLSLTDK	0.38	0.0937		
	LMDIIINSLYSNK	0.20	0.0993		
Glyma15g39370.2	glyoxalase II 3	4	19.00	0.0001	biodegradation of Xenobiotics
	Peptide Sequnce	Ratio 4(2)F+Ca	Pvalue 4(2)F+Ca		
	DLSLIEQLGLK	20.10	0.0027		
	GCVTYVTGDAPDQPQPR+Carbamidomethyl(2)	20.20	0.0056		
	MAFTGDTLLIR	13.24	0.0634		
	TVDRDLSLIEQLGLK	1.92	0.1413		
Glyma13g31390.1	Rubber elongation factor protein (REF)	2	19.21	0.0109	not assigned
	Peptide Sequnce	Ratio 4(2)F+Ca	Pvalue 4(2)F+Ca		
	VSAYLPLVPTEK	18.82	0.0602		
	YEPTAEQCAVSAWR+Carbamidomethyl(8)	21.32	0.0240		
Glyma09g29300.1	Kunitz family trypsin and protease inhibitor protein	7	22.58	0.0124	stress
	Peptide Sequnce	Ratio 4(2)F+Ca	Pvalue 4(2)F+Ca		
	EGVNYNILISMPYTSCR+Carbamidomethyl(16)	23.56	0.2045		
	ETIGNWFK	3.96	0.2534		
	IGNSCPLDVVVVDINHRLPLR+Carbamidomethyl(5)	1.15	0.8588		
	LALSDVPYQFR	25.73	0.1111		

	LVTTGGVVGYPGR	59.31	0.0218		
	LVYCPSVCPSCK+Carbamidomethyl(4)Carbamidomethyl(8)Carbamidomethyl(11)	43.81	0.0932		
	VATDLNIMFPDR	0.40	0.0659		
Glyma11g08260.1	Rhodanese/Cell cycle control phosphatase superfamily protein	3	31.05	0.0340	protein
	Peptide Sequnce	Ratio 4(2)F+Ca	Pvalue 4(2)F+Ca		
	DMGGGYVDWVK	34.73	0.0953		
	SLIQTGSIYLDVR	13.27	0.1542		
	SLYATADLLSDGFK	35.43	0.0726		
Glyma09g29330.1	Kunitz family trypsin and protease inhibitor protein	6	46.15	0.0002	stress
	Peptide Sequnce	Ratio 4(2)F+Ca	Pvalue 4(2)F+Ca		
	DVGVFVDENGYR	1.77	0.0719		
	IVYCPSVCPSSK+Carbamidomethyl(4)Carbamidomethyl(8)	57.24	0.1140		
	LALSDVPFK	173.69	0.0152		
	TSYSCAEYSPVWK+Carbamidomethyl(5)	16.31	0.0132		
	VSTDLNIMFCTDR+Carbamidomethyl(10)	29.73	0.0403		
	WFVTTGGSMGNPSWETIR	137.47	0.1194		
Glyma09g27700.1	Concanavalin A like lectin protein kinase family protein	5	406.84	0.0000	signalling
	Peptide Sequnce	Ratio 4(2)F+Ca	Pvalue 4(2)F+Ca		
	LDDSGKPEFGSVGR	423.09	0.0002		
	NVLQLTK	23.62	0.0228		
	SSQLVSTFETTFTFK	160.79	0.0040		
	TVTAQISYNSASK	667.27	0.1613		
	VLYFAPVHLWK	673.92	0.0066		

Protein ID, according to the Phytozome database; M.P., number of matched peptide; Ratio, relative abundance of protein; Function, functional classification by MapMan bin code; *N.D. Not Description in Phytozome database.
